# The impact of perceived school climate on exercise behavior engagement among obese adolescents: a dual mediation effect test of exercise benefits and perseverance qualities

**DOI:** 10.3389/fpsyg.2023.1220362

**Published:** 2023-10-03

**Authors:** Yao Yin, Chong Zhang, Zhibo Chen, Yufei Qi, Cheng Qiu

**Affiliations:** ^1^Progression School of Upper Secondary, Beijing College of Finance and Commerce, Beijing, China; ^2^Department of Physical Education, Shanghai Dianji University, Shanghai, China; ^3^Department of Physical Education and Research, Central South University, Changsha, China; ^4^Police Sports and Warfare Training Academy, People’s Public Security University of China, Beijing, China

**Keywords:** obese adolescents, physical activity, sports participation, dual mediation effect, individual perception

## Abstract

**Introduction:**

This study explores the relationship between perceived school climate and exercise behavior among obese adolescents, as well as the multiple mediating effects of perseverance qualities and exercise benefits.

**Methods:**

A survey was conducted on 586 obese adolescents in Beijing, with an age range of 13–18 years old and an average age of 15.40 ± 1.824, among who 337 were male, 249 were female, 303 were high school students and 238 were middle school students. A standard scale was used to evaluate perceived school climate, exercise benefits, perseverance qualities, and exercise behaviors. The data was analyzed by independent samples *t*-test, bivariate correlation analysis, descriptive statistical analysis, and structural equation model (SEM).

**Results:**

(1) Perceived school climate among obese adolescents positively predicted exercise behavior (*Z* = 2.870, *p* < 0.01), perseverance qualities (*Z* = 3.107, *p* < 0.01) and exercise benefits (*Z* = 4.290, *p* < 0.001); perseverance qualities positively predicted exercise behavior in obese adolescents (*Z* = 4.431, *p* < 0.001); exercise benefits positively predicted the obese adolescents’ exercise behavior (*Z* = 4.267, *p* < 0.001). (2) Perseverance qualities (*Z* = 2.282, 95% CI [0.032, 0.191], [0.028, 0.179]) and exercise benefits (*Z* = 2.518, 95% CI [0.060, 0.287], [0.053, 0.271]) play a mediating role in the obese adolescents’ perceived school climate and exercise behavior, respectively. These two factors have parallel multiple mediating effects between obese adolescents’ perceived school climate and exercise behavior, with mediating effects accounting for 16 and 25%, respectively. The mediating effect of exercise benefits is greater than that of perseverance qualities. (3) There is no difference in the specific indirect effects of perseverance qualities and exercise benefits (*Z* = −0.800, 95% CI [− 0.198, 0.064], [−0.190, 0.068]).

**Conclusion:**

Obese adolescents’ perception of school climate can effectively enhance their motivation to participate in exercise behavior and indirectly influence exercise behavior through exercise benefits and perseverance qualities, cultivate good physical exercise behavior among obese adolescents, and effectively prevent and intervene in the occurrence of obesity.

## Introduction

The increasing prevalence of obesity among adolescents is a serious public health issue. Obesity is a chronic disease, mainly caused by an energy imbalance between excessive calorie intake from food, calories consumption or insufficient physical activity, resulting in the enlargement or increase of fat cells (Bray, 2004; Camacho and Ruppel, 2017; Ibrahim et al., 2021).

*The Report on the Nutrition and Chronic Disease Status of Chinese Residents (2020)* shows that the overweight and obesity rate of children and adolescents aged 6–17 has risen to 19%. *The China Children’s Obesity Report* points out that the overweight and obesity rate of children and adolescents in China is also constantly rising, and if without intervention, the number of overweight and obese children aged 7 and above will rise to nearly 50 million by 2030 (Zhang and Ma, 2017). The focus of *Implementation Plan of Obesity Prevention and Control in Children and Adolescents* is to promote the balance between food intake and physical activity among children and adolescents, strengthen school responsibilities, maintain a healthy weight for children and adolescents, ensure physical activity time at school, strengthen physical education classes and extracurricular exercise (China, M. o. E. o. t. P. s. R. o, 2020), and thus to control obesity weight through physical exercise (Xu et al., 2021). The high prevalence of overweight and obesity is mainly due to a decrease in physical activity levels (Tremblay et al., 2011; Tabacchi et al., 2016; Marsigliante et al., 2022), and regular physical exercise can effectively intervene in obesity and is an important factor in preventing overweight (Strong et al., 2005; Richardson et al., 2013; Wyszyńska et al., 2020; Marsigliante et al., 2022).

The World Health Organization defines physical activity as any physical movement performed by skeletal muscles that requires energy consumption. Physical activity refers to all activities, including leisure time activities, traveling between different locations, or as part of a person’s job. Both moderate and high intensity physical activity can improve health (World Health Organization, 2021). And activity behavior refers to the conscious and active participation in physical exercise by individuals under the influence of a variety of internal and external stimuli, excluding passive or forced participation (Pan et al., 2021). The Theory of Planned Behavior points out that the factors that affect behavior are indirectly influenced by behavioral intention (weight control), and physical exercise can effectively reduce BMI, body fat percentage, and other factors in obese individuals (Marteau et al., 2012).

Due to spending most of their adolescence in school, the school sports environment is a setting that provides opportunities for teenagers to participate in behaviors that have a positive impact on them. Research has shown that schools provide an appropriate environment for implementing positive and healthy lifestyle interventions (Ip et al., 2017), and learning correct and healthy lifestyles is an effective way to intervene in obese students (Lirola et al., 2021). Therefore, in order to continue improving the predictive explanatory abilities of obese adolescents’ behavior, this study investigated the transition process from perceived school climate to exercise behavior among obese adolescents, and it also investigated whether exercise benefits and perseverance qualities mediate the relationship between perceived school climate and exercise behavior among obese adolescents, in order to determine other key influencing factors of perceived school climate and exercise behavior.

### Perceived school climate and exercise behavior

Perceived school climate refers to the humanistic environmental characteristics that are perceived or experienced by school members as having the significant psychological and behavioral impact that are enduring and stable. Human behavior is not only driven by thoughtful considerations (e.g., knowledge, attitudes, and beliefs), but also influenced by environmental stimuli (Ge and Yu, 2006). For teenagers, school is an important place for them to learn and exercise, and the school environment climate is a unique cultural attribute formed by it, which is an internal psychological characteristic shared by school members (Lv, 2021). Previous studies have shown that the school sports environment not only has a direct effect on physical exercise behavior, but also has a specific indirect effect on physical exercise behavior through mediating variables (Ma et al., 2022), and the school sports venue environment can enhance the adolescents’ physical activity level, thus effectively reduce the risk of obesity (Duckworth et al., 2007). Accordingly, the research hypothesis is proposed. H1: There is a significant positive correlation between perceived school climate and physical exercise behavior among obese adolescents.

### The mediating role of perseverance qualities

Perseverance qualities refer to an individual’s tendency to strive to achieve long-term goals and maintain passion and effort, especially in situations of challenges and setbacks, which can help individuals better adapt to the environment (Hu et al., 2022). Current research on perseverance qualities mainly focuses on the cultivation level (Song, 2019a,b) and the relationship between academic perseverance and academic performance (Wang, 2021). Persistence is a form of perseverance qualities, and the perseverance character of exercise behavior can be effectively control body weight (Zhang and Chen, 2010), achieving the effect of reducing fat. The level of teacher support in the school environment climate is significantly positively correlated with perseverance qualities (Li, 2020), and teacher support can effectively enhance students’ perseverance qualities. Accordingly, the research hypotheses were proposed: H2: perceived school climate is an important factor influencing the perseverance qualities of obese adolescents, and perceived school climate positively influences adolescents’ perseverance qualities. Hypothesis H3: perseverance quality is an important factor influencing adolescents’ adherence to exercise, and it positively predicts exercise behavior. Based on the hypotheses of H2 and H3, this study aims to verify the mediating effect of perseverance qualities on perceived school climate and exercise behavior.

### The mediating effect of exercise benefits

Exercise benefits perception refers to the obese adolescents’ perception of various benefits of physical activity, and physical exercise benefits refer to an individual’s intuitive evaluation and perception of the results of accompanying physical exercise behavior (Guo and Xu, 2011). A reasonable cognitive concept and correct understanding of the benefits of physical exercise will lead to reasonable physical exercise behavior (Wang, 2021). Students’ perception of the teaching and learning environment will fluctuate with environmental perception factors, thereby affecting their cognitive engagement (exercise benefits) (Lu et al., 2021). Thus, it can been seen that perception of the school learning environment is an important factor for individuals to perceive the exercise benefits, and exercise benefits are important predictive factors for promoting individuals’ physical exercise behaviors. Accordingly, a hypothesis model H4 is proposed: obese adolescents perceive school climate as a positive predictor of individual cognition of exercise benefits; hypothesis H5: the exercise benefits positively predict physical exercise behavior. Based on hypotheses H4 and H5, it is verified that the mediating effect of exercise benefits on obese adolescents’ perceived school climate and physical exercise behavior.

**Figure 1:**
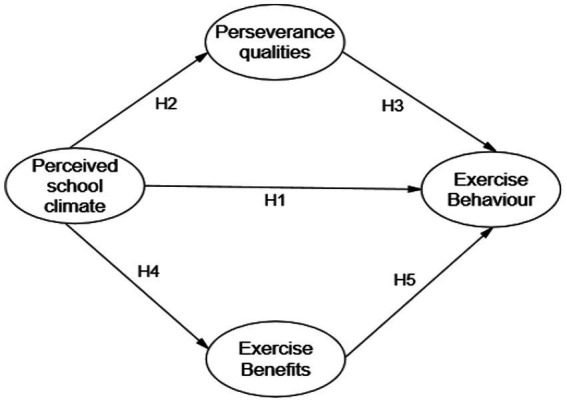
A hypothetical model for perceived school climate and physical exercise behavior.

In summary, there is currently limited empirical research in the field of physical education on the impact of perceived school climate on students’ psychological aspects of exercise behavior among obese adolescents. From the perspective of the psychology of exercise behavior, this study constructs a path model for the influence of exercise behavior intention among obese adolescents with dual mediating effects of exercise benefits and perseverance qualities, and analyses the mechanism of action to enrich the theory of exercise behavior among obese adolescents, and ultimately, it encourages them to consciously form the practical significance of adherence to physical exercise. Based on the above theoretical assumptions, a theoretical model of the relationship between perceived school climate and physical exercise behavior was constructed (see Figure 1).

### Research objects and methods

#### Survey objects

The Body Mass Index (BMI) method was adopted as the standard for selecting obese adolescents, and the calculation formula is: BMI = weight[kg] ÷ (height[m])^2^, and BMI ≥ 28 can be determined as obesity. Sixty eight teaching classes from 10 ordinary high schools in Beijing were selected, and 600 obese adolescents were screened through the results of the physical fitness test and physical education teacher sampling to conduct a questionnaire survey on the relationship between perceived school climate and exercise behavior. After removing invalid questionnaires, 586 valid questionnaires were obtained, with a valid questionnaire rate of 98%. The age range of the subjects was 13–18 years old, with an average age of 15.40 ± 1.824, including 93 senior 1 students, 119 senior 2 students, 91 senior 3 students, 142 junior 1 students, 85 junior 2 students, and 56 junior 3 students; 337 male students (57.5%) and 249 female students (42.5%), 303 were senior high school students, and 238 were junior high school students.

This study was approved by the Ethics Committee of Xiangya School of Public Health, Central South University (Project No. XYGW-2022-44; 20 July 2022). and conducted in accordance with the guidelines of *Declaration of Helsinki*. Students were informed prior to the survey that the questionnaires were answered anonymously, emphasizing voluntary completion. The content is strictly confidential and the results are only used for scientific research. All questionnaires were collected on the spot. All subjects were informed of the purpose and characteristics of the study and signed an informed consent form.

### Survey tools

#### Physical Activity Rating Scale

The Physical Activity Rating Scale (PARS-3) revised by Liang (1994) was used, which examines the amount of exercise from three items: intensity, time and frequency of participation in physical activity. The 5-point Likert scale was used, with the total score representing the level of physical activity, and the higher the score, the higher the level of physical activity. The study has proved that the Cronbach’s a coefficient of the scale in physical activity test for college students was 0.820. The Cronbach’s alpha coefficient of the questionnaire in this study was 0.710.

#### Perceived School Climate Scale

The Perceived School Climate Scale developed by Jia et al. (2009) was used, which consists of 25 items, including three dimensions: teacher support (7 items), peer support (13 items), and autonomy opportunities (5 items), and is scored on a 4-point scale of “1 (never) to 4 (always).” The higher the score, the better the perceived school climate, and the higher the exercise level of the participants’ perceived school climate. The study has confirmed that the scale has high reliability among Chinese and American adolescents. Cronbach’s alpha coefficients ranges from 0.69 to 0.86. The Cronbach’s alpha coefficient for the total scale in this study is 0.960, with dimensions ranging from 0.89 to 0.98.

#### Chinese version of perseverance qualities scale (12-Item Grit Scale, 12-IGS)

Xie et al. (2017) translated and revised the Grit Scale, which consists of 12 items, including 2 variables of unremitting effort and enduring enthusiasm. Each variable contains 6 items, and is scored on a 5-point scale. The higher the score, the higher the level of perseverance qualities. This study has proved that the Chinese version of the scale has been preliminarily revised in the adult population in China, with a Cronbach’s a coefficient of 0.729. The Cronbach’s alpha coefficient of the scale is 0.941 in this study.

#### Exercise benefits scale

Adopting the top 5 items in the study by Wang et al. (2022) that ranked the highest proportion of agreement and strong agreement on the benefits of exercise, the item “Exercise can prevent heart attacks” was revised to “Exercise can prevent obesity.” Each item used the Likert 5-level scoring method, and the higher score, the better the benefits from exercise. The study has proved that the Cronbach’s alpha coefficient of this scale is 0.820 in the exercise behavior of elderly patients with chronic obstructive pulmonary disease (COPD), and the Cronbach’s alpha coefficient of this scale is 0.702 in this study.

#### Mathematics statistics

Firstly, SPSS 26.0 (SPSS Inc., Chicago, IL, United States) was used to input and organize the collected data. Independent samples *t*-test was used to analyze the differences in direct quantitative data between two different clusters. The obtained data was tested using the Harman’s single-factor method. Then, bivariate correlation analysis was used to analyze the correlation between various dimensions and measure the degree of correlation between the two variable factors. The statistical description is expressed in terms of mean and standard deviation (M ± SD).

Structural Equation Model (SEM) was then used to estimate the correlations between physical exercise, potential influencing factors and pathways. The AMOS 24.0 (SPSS Inc., Chicago, IL, United States) maximum likelihood estimation method was used for SEM, and the applicability of the model was determined through a variety of indices, including the ratio of the minimum difference to degree of freedom (CMIN/DF), the Goodness of Fit Index (GFI), Adjusted Goodness of Fit Index (AGFI), Comparative Fit Index (CFI) and Standardized Root Mean Square Residual (SRMR) (Jackson et al., 2009; Whittaker, 2011). Direct effect analysis focuses on non-standardized path coefficients, with significance levels set at *p* < 0.05 and Z > 1.96.

Finally, a two factor mediating effect analysis was conducted to determine whether the relationship between perceived school climate and exercise behavior was indirectly influenced by perseverance qualities and exercise benefits. 5,000 Bootstrap multiple mediating effects tests were conducted using the syntax in AMOS 24.0, including direct effects, indirect effects, and specific indirect effects to compare differences. The test results are mainly determined through the bias-corrected 95% and Percentile 95% estimation methods to determine whether the confidence interval obtains contain 0.

## Results and analysis

### Basic statistical data

The study used independent sample *t*-tests to determine whether there were differences between different genders in 4 dimensions of perceived school climate, perseverance qualities, exercise benefits and exercise behavior. The results showed that there were no significant differences between different genders and these 4 dimensions at the 0.05 level, as detailed in Table 1.

**Table 1:** Independent samples *t*-test of different genders and dimensions.

Dimension	Mean equivalence *t*-test				Gender	Number of cases	*M*	*SD*
	T	DF	*P*	Mean difference				
Perceive school climate	−1.330	584	0.184	−0.055	Male	337	3.574	0.523
					Female	249	3.630	0.460
Perseverance qualities	−0.804	584	0.421	−0.063	Male	337	3.951	0.917
					Female	249	4.014	0.964
Exercise behavior	0.326	584	0.745	0.025	Male	337	3.820	0.921
					Female	249	3.795	0.895
Exercise benefits	−0.222	584	0.824	−0.009	Male	337	3.326	0.496
					Female	249	3.335	0.484

Independent sample *t*-tests were used to determine whether there are differences between different grades in 4 dimensions of perceived school climate, perseverance qualities, exercise benefits, and exercise behavior. The results show that there is no significant difference between different grades and these 4 dimensions at the 0.05 level, as shown in Table 2.

**Table 2:** Independent sample *t*-test of different grades and dimensions.

Item	Mean equivalence *t*-test				Grade	Number of cases	*M*	*SD*
	T	DF	*P*	Mean difference				
Perceive school climate	−1.419	584	0.156	−0.058	Senior high school	303	3.570	0.537
					Junior high school	283	3.628	0.451
Perseverance qualities	−0.421	584	0.674	−0.033	Senior high school	303	3.962	0.954
					Junior high school	283	3.995	0.920
Exercise behavior	−1.540	584	0.124	−0.116	Senior high school	303	3.754	0.883
					Junior high school	283	3.869	0.935
Exercise benefits	−0.319	584	0.750	−0.013	Senior high school	303	3.323	0.499
					Junior high school	283	3.336	0.483

### Common method control and test

Common method biases refer to the artificial covariation between predictor and criterion variables due to the same data source or rater, measurement environment, project context, and project characteristics. In order to prevent the possibility of common method bias in the collected data, corresponding control measures have been taken during the testing process. The Harman single factor method was used to test for covariance. The test criteria were: there were more than one factor with a characteristic root greater than 1 and the explanatory degree of the maximum factor’s variance was less than 40%. The results of this study show that there are a total of 7 factors with eigenvalues greater than 1 and the variance explained by the first factor was 24.17%, which is less than the critical criterion of 40%. The extraction amount of common variance is higher than 70% (74.02), which indicates that there is no common method bias (Zhou, 2004).

### Correlation analysis of various dimensions

In this study, the AVE method was used to test the discriminant validity of each dimension. The AVE method was proposed by Fornell and Larcher, which means that the average variance extraction amount of each dimension must be greater than the square value of the correlation coefficients between each dimension. However, since the AVE is a square value, it must first be converted to the same square unit if it is to be compared with Pearson correlation between dimensions. Therefore, the AVE value was opened and rooted before comparison could be made. Therefore, the AVE value was marked with a root before it could be compared. If it is higher than the Pearson correlation value between dimensions, it can be declared that the dimension has differential validity. In this study, except for the exercise behavior dimension of 0.458, all other dimensions were greater than 0.5, which is in line with the criteria proposed by Hair et al. (2009).

The correlational analysis of obese adolescents’ perceived school climate (three subscales of teacher support, peer support, and autonomy opportunity), perseverance qualities (two subscales of unremitting effort and persistent enthusiasm), exercise benefits, and exercise behavior (Table 3) shows that teacher support is significantly and positively correlated with peer support, autonomy opportunity, unremitting effort, persistent enthusiasm, exercise behavior, and exercise benefits (*r* = 0.098–0.438, *p* < 0.01 or *p* < 0.05). Peer support is significantly and positively correlated with autonomy opportunity, exercise behavior and exercise benefits (*r* = 0.168–0.540, *p* < 0.01). Autonomous opportunity is significantly positively correlated with unremitting effort, persistent enthusiasm, exercise behavior and exercise benefits (*r* = 0.141–0.185, *p* < 0.01). Unremitting effort is significantly positively correlated with persistent enthusiasm (*r* = 0.645, *p* < 0.01) and exercise behavior (*r* = 0.210, *p* < 0.01). Persistent enthusiasm is significantly positively correlated with exercise behavior (*r* = 0.211, *p* < 0.01) and exercise benefits (*r* = 0.095, *p* < 0.05). Exercise behavior is significantly positively correlated with exercise benefits (*r* = 0.249, *p* < 0.01).

**Table 3:** Statistics for correlation analysis of various variables.

Variables	Teacher support	Peer support	Autonomy opportunity	Unremitting efforts	Persistent enthusiasm	Exercise behavior	Exercise benefits	*M*	*SD*
Teacher support	**0.844**							3.602	0.680
Peer support	0.358**	**0.817**						3.638	0.596
Autonomy opportunity	0.438**	0.540**	**0.708**					3.552	0.607
Unremitting efforts	0.098*	−0.015	0.141**	**0.725**				4.009	1.000
Persistent enthusiasm	0.103*	−0.003	0.152**	0.645**	**0.754**			3.947	1.066
Exercise behavior	0.170**	0.175**	0.185**	0.210**	0.211**	**0.458**		3.809	0.910
Exercise benefits	0.134**	0.168**	0.158**	0.071	0.095*	0.249**	**0.634**	3.330	0.491

**At the 0.01 level (two-tailed). *Correlations significant at 0.05 level (two-tailed), diagonal bold text is open root value of AVE, lower triangle is Pearson correlation of dimensions.

#### Model goodness of fit test

**Figure 2:**
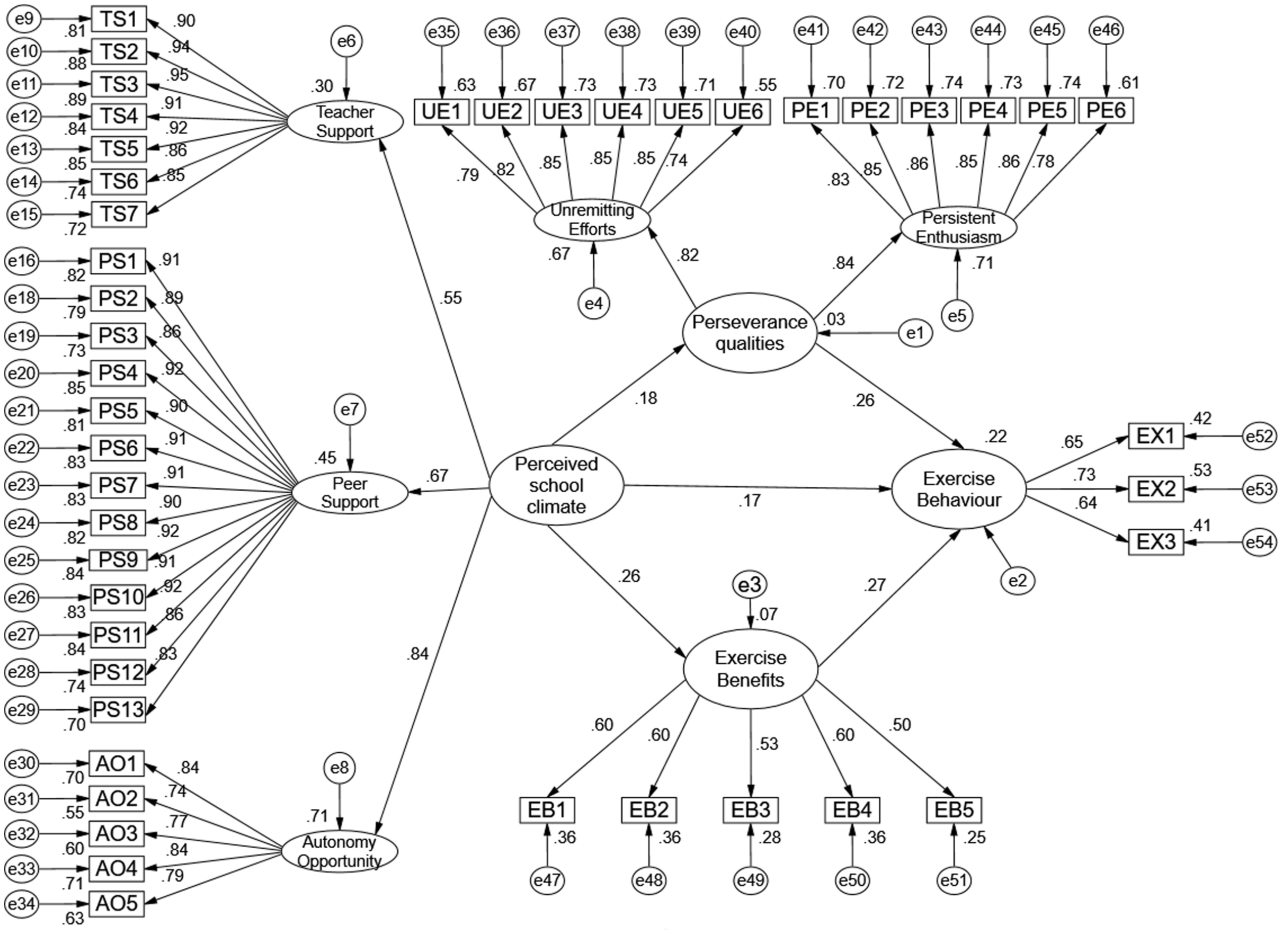
Model fitting of the relationship between perceived school climate and exercise behavior among obese adolescents.

The model fitting test result of the relationship model between perceived school climate and exercise behavior among obese adolescents constructed in this study are good. CMIN/DF = 1.78 < 3; GFI = 0.887 > 0.8 acceptable, AGFI = 0.875 > 0.8 acceptable, IFI = 0.970, CFI = 0.970, TLI = 0.968, RMSEA = 0.036 < 0.08, SRMR = 0.036 < 0.05, and above empirical path analysis goodness-of-fit indicators all meet the empirical rule of thumb criteria suggested by general scholars (Fornell and Larcker, 1981). It can be used for later statistical data analysis to indicate the effectiveness of the equation model of the relationship between perceived school climate and exercise behavior among obese adolescents. The model is shown in Figure 2.

### Model hypothesis testing

#### Direct effect path test

The direct path results of the relationship between perceived school climate and exercise behavior among obese adolescents (see Table 4) show that there are five direct impact paths between the four latent variables of perceived school climate, perseverance qualities, exercise benefits and exercise behavior. The non-standardized coefficient results mainly evaluate whether the results are significant or not. This study shows that each impact path is of significant and valid, with *p* < 0.05 and Z > 1.96.

**Table 4:** Path analysis results.

Direct path impact	Non-standardized path coefficient	Standard error	Z	C.R.	*P*	Standardized path coefficient
Perceived school climate - > Perseverance qualities	0.348	0.112	3.107	3.108	0.002	0.176
Perceived school climate - > Exercise benefits	0.276	0.064	4.313	4.290	0.000	0.265
Perceived school climate - > Exercise behavior	0.330	0.115	2.870	2.876	0.004	0.169
Exercise benefits - > Exercise behavior	0.512	0.120	4.267	4.275	0.000	0.274
Perseverance qualities - > Exercise behavior	0.257	0.058	4.431	4.434	0.000	0.261

The standardized path coefficient mainly evaluate the significance of each path coefficient. The results showed that among the path coefficients of exercise behavior as the dependent variable, the absolute value path coefficient of exercise benefits was the highest, with a standardized absolute value path coefficient of 0.274, accounting for 23.9% of the total effect. The path coefficient of perseverance qualities was 0.261, accounting for 22.8% of the total effect. The minimum path coefficient of perceived school climate was the smallest, 0.169, accounting for 14.8% of the total effect. In the direct influence path coefficient of perceived school climate as an independent variable, the maximum path of exercise benefits in the standardized coefficient is the highest, 0.265, accounting for 23.1% of the total effect. The path coefficient of perseverance qualities is 0.176, accounting for 15.4% of the total effect. The above data shows that the proportion of coefficient effects and total effects of each path is different, with H5 > H4 > H3 > H2 > H1.

#### Two factor mediation effect test

The causal path test and the Sobel *Z-*test are also statistical methods for testing the existence of mediating effects. In the causal path test (Baron and Kenny, 1986) a and b statistics are significantly representative of the existence of mediation effect. Sobel *Z-*test is a coefficient product test, and Sobel t has a major flaw that requires the indirect effect of the sample to be normal, but a*b is basically asymmetric (not normal), with skewness and kurtosis not zero (Hayes, 2009).

This study used Bootstrap 5,000 to test the mediating effect and whether there were differences in specific indirect effects. 5,000 Bootstrap samples were extracted and put back in the original data to form an approximate sampling distribution, and indirect effects, direct effects and specific indirect effects were tested for differences. The test results are as follows (see Table 5): Perceived school climate has a direct effect on exercise behavior (*Z* = 2.481, 95% CI [0.107, 0.639], [0.101, 0.632]), with a *Z*-value greater than 1.96 and a 95% CI confidence interval that does not include 0, suggesting that perceived school climate is a significant factor in predicting exercise behavior. The mediating effect of perseverance qualities on perceived school climate and exercise behavior (*Z* = 2.282, 95% CI [0.032, 0.191], [0.028, 0.179]), and the indirect effect of exercise benefits on perceived school climate and exercise behavior also exists (*Z* = 2.518, 95% CI [0.060, 0.287], [0.053, 0.271]). Among them, the perceived school climate has the greatest direct effect on exercise behavior, with an effect value of 0.330, accounting for 59%. In the indirect effect, the perceived school climate - > exercise benefits - > exercise behavior path effect value is 0.141, accounting for 25%. The perceived school climate - > perseverance qualities - > exercise behavior path effect value is 0.089, accounting for 16%. There was no significant difference in the mediating effect of exercise benefits compared to perseverance qualities (*Z* = −0.800, 95% CI [−0.198, 0.064], [−0.190, 0.068]). The *Z*-value was less than 1.96 and the 95% CI confidence interval include 0. However, the mediating factor of exercise benefits was still more important than the mediating factor of perseverance qualities (0.141 > 0.089).

**Table 5:** Results of the two factor mediation test for perseverance qualities and exercise benefits.

Variable path	Point estimation value	Coefficient product		Bootstrapping			
				bias-corrected 95% CI		Percentile 95% CI	
		SE	*Z*	Lower	Upper	Lower	Upper
Indirect effect							
Perceived school climate - > Perseverance qualities- > Exercise behavior	0.089	0.039	2.282	0.032	0.191	0.028	0.179
Perceived school climate - > Exercise benefits - > Exercise behavior	0.141	0.056	2.518	0.060	0.287	0.053	0.271
Direct effect							
Perceived school climate - > Exercise behavior	0.330	0.133	2.481	0.107	0.639	0.101	0.632
Total effect	0.560	0.150	3.733	0.312	0.898	0.311	0.896
Comparison of mediation effect differences							
Perseverance qualities vs. Exercise benefits	−0.052	0.065	−0.800	−0.198	0.064	−0.190	0.068

Bootstrap 5,000 self-sampling was used.

## Discussion

### The relationship between perceived school climate, exercise benefits, perseverance qualities and exercise behavior among obese adolescents

The results of this study found that obese adolescents have a significant positive correlation between their perception of school climate and exercise behavior. This may be due to the fact that school is an important place for adolescents to learn and live. Obese individuals’ perceptions of school climate, including teacher-student relationships, peer relationships, and independent opportunity choices, directly affects their emotional experience in school life, and further influences their cognition and engagement in exercise behavior. School based social environment factors (e.g., teacher behavior) are associated with physical exercise among adolescents. In one study, students, teachers, principals and parents recognized that the overall school climate indeed promotes children’s participation in physical activity (Macquarrie et al., 2015). The climate created by teachers in the classroom is associated with more activities both inside and outside of physical education, and students believe that they receive rewards for learning and improving the latter by outperforming others. The teacher’s “independent support” in physical education classes also shows a consistent association with physical exercise (Morton et al., 2016). The importance of teachers providing encouragement (Zhang and Ma, 2017) and supporting sports activities (Kubik et al., 2005; Robbins et al., 2010) show a positive correlation. Role models (Zhang and Ma, 2017) are also emphasized as important behaviors for teachers to encourage students. In a quantitative study of 93 papers with varying quality, intervention in environmental factors can increase the participation in physical exercise among adolescents (Morton et al., 2016), and the research has demonstrated that the school environment positively influences outdoor physical exercise (Liu et al., 2021) (*β* = 0.97, *p* < 0.001), this study perceived that the school climate environment positively predicted exercise behavior (*Z* = 2.481, 95% CI [0.107, 0.639], [0.101, 0.632]). The lack of school climate is not effective in intervening in physical exercise among adolescents (Doak et al., 2006). Perceived exercise benefits among obese adolescents significantly predict exercise behavior (Xue-liu and Mu, 2021), and physical exercise can promote physical health through different exercise methods. Conversely, perceived exercise benefits among obese adolescents can also influence their exercise behavior (Callaghan et al., 2002). There is a significant positive correlation between individual perseverance qualities and exercise behavior. Obese adolescents’ enthusiasm and persistence in perceiving the school sports environment climate during school may enhance their sense of individual cognition. Perseverance qualities emphasize the maintenance of sustained enthusiasm and hard work when pursuing long-term goals (weight loss) (Von Culin et al., 2014), perseverance quality, exercise commitment, and athletic performance were significantly and positively associated with exercise performance (Zhai et al., 2019) (*β* = 0.284, *p* < 0.01). Obese adolescents perceive that school climate has a positive contributor to exercise behavior, and intervention in the environment can effectively increase their participation in physical exercise. The research has pointed out that school based physical exercise interventions mainly focus on students’ motivation, basic psychological needs, goal orientation, enjoyment and stimulating teaching atmosphere in physical education (Demetriou et al., 2019). Exercise benefits and perseverance qualities are individual psychological activity behaviors in this study. It was found that these two factors are also important psychological indicators for intervening in adolescents’ participation in school based physical exercise, and good psychological perceptions can promote better engagement in exercise behavior among obese individuals.

### The mediating effect of perseverance qualities between perceived school climate and exercise behavior among obese adolescents

The results of this study show that the perseverance qualities of obese adolescents plays a mediating role in their perceived school climate and exercise behavior. On the one hand, it indicates that obese adolescents’ perceived school climate directly influences exercise behavior. The better the perceived school climate among obese adolescents, the more beneficial it is for their participation in exercise behavior, which is basically consistent with the existing research results (Guo, 2019). The explanatory power of school factors on the total amount of physical exercise among college students is 20.2%, and the explanatory power of this study reaches 59%. On the other hand, obese adolescents’ perceived school climate influences exercise behavior engagement through the mediating effect of perseverance qualities (unremitting effort and persistent enthusiasm). Perceived school climate positively affects individual perseverance qualities, and perceived teacher behavior in school climate positively predicts students’ perseverance qualities (Reed, 2014). Teacher support significantly and positively predicts perseverance qualities (=0.174, 0.182, *t* = 5.092). Teachers are able to help students establish correct beliefs and behaviors by constructing supportive relationships (Li, 2020), so it is hypothesized that perceived school climate can positively predict perseverance qualities among obese adolescents. Perseverance quality is a significant predictor of individual exercise behavior (Reed, 2014). Psychological studies have shown that perseverance qualities are positively correlated with academic performance (Liu and Gao, 2020). Both interest conformity and persistent effort significantly and positively predict academic performance in T1, β interest conformity = 0.26, *p* < 0.001 and β persistent effort = 0.08, *p* < 0.001. Perseverance qualities significantly predicted exercise behavior in this study, with *Z* = 4.431, *p* < 0.001. Persistent effort can also increase the occurrence of exercise behavior among obese adolescents. Therefore, existing relevant studies provide supporting evidence for the results of this study. This study verified the mediating effect of perseverance qualities on the relationship between perceived school climate and exercise behavior among obese adolescents, and clarified the relationship between latent variables such as perceived school climate, perseverance qualities and exercise behavior.

### The mediating effect of exercise benefits between perceived school climate and exercise behavior among obese adolescents

The results of this study suggest that the perceived exercise benefits among obese adolescents play a mediating role between perceived school climate and exercise behavior. The results also indicate that obese adolescents’ perception of school climate can not only directly predict exercise behavior, but also indirectly influence exercise behavior through the “bridge” of exercise benefits. The cultural environment of schools can influence students’ cognition and identification (Yang, 2022), and cognition can affect behavior, and perceived school climate can promote both behavior and cognition (Jiang, 2019), perceived school climate has a significant correlation with adolescent externalization behavior (*β* = −0.112, SE = 0.037, 95% CI [−0.185, −0.039]) (Pan et al., 2023), consistent with the results of this study (*β* = −0.330, SE = 0.133,95% CI [0.107, 0.639], [0.101, 0.632]), and some studies have shown a certain relationship between family environment and perceived exercise benefits (Guo et al., 2017). This study also verified that perceived school climate significantly predicts exercise benefits. Perceived exercise benefits positively influence exercise behavior (Xue-liu and Mu, 2021), with an effect size of 0.234. The perceived exercise benefits among obese individuals can enhance their cognitive level, thereby enhancing the occurrence of exercise behavior. This study validated the mediating effect of exercise benefits on the relationship between perceived school climate and exercise behavior among obese adolescents, and clarified the relationship between latent variables such as perceived school climate, exercise benefits and exercise behavior.

This is a cross-sectional study that is consistent with the research methods of Ip et al. (2017). They used robust Poisson regression to verify that school students with at least 11 sports friendly environmental factors (7.0%) have a much lower risk of obesity than students without environmental factors. A physical activity friendly school environment is associated with a lower risk of obesity. We collected exercise behavior data on school physical activity environment, teacher support and peer support. The analysis verified that perceived school climate can directly affect exercise behavior (Rodrigues et al., 2018; Dong, 2021), and that three factors in the school environment, namely “peer support,” “teacher support” and “autonomy opportunity,” influence students’ participation in physical exercise (Guo, 2019). The two factors of perseverance qualities and exercise benefits play a mediating role between perceived school climate and exercise behavior, suggesting that perceived school climate can also indirectly influence exercise behavior through perseverance qualities and exercise benefits.

Insufficient physical activity is not only common in high-income countries, but also in low-income and middle-income countries (LMICs). According to reports, the physical activity levels of Asian school-age children and adolescents are also low (Dan et al., 2011; Shokrvash et al., 2013). Diversified intervention measures (combining education with physical activity courses, educational materials, school environments and family education) may be promising strategies for increasing physical activity among Asian children and adolescents (Barbosa Filho et al., 2016; Ahmed et al., 2021). Comprehensive multi-component interventions in sports, such as participating in health education courses, participating in physical exercise after class or after school, and involving family members together, are more effective than a single strategy interventions (Salmon et al., 2007; van Sluijs et al., 2008; Meyer et al., 2011; Barbosa Filho et al., 2016). Previous studies have shown that focusing on changes in multiple health behaviors is more effective than a single strategy (De Meester et al., 2009; Crutzen, 2010).

School policies can also reduce unhealthy dietary behaviors and obesity risk among children in mega cities in China (Ding et al., 2023). Schools with prolonged physical activity may reduce the likelihood of obesity among students, and adequate physical activity may help prevent childhood obesity (Sprengeler et al., 2017). School management policies mainly include: written guidelines for the school, annual work plans for the school’s sports and health departments, monitoring of students’ physical health status, student obesity prevention meetings, and involvement of class administrators. School health education: health education (such as professional teachers and curriculum arrangements), obesity related lectures, student courses and activities, advice for overweight/obese students, and school obesity prevention reports for parents (Cheng et al., 2020). School environmental factors are the result of multiple levels of interaction, and strategies should not only focus on children and adolescents at the interpersonal level, but also on friends, teachers, parents, and school administrators, and school based interventions may increase the participation rate of adolescents in physical activity (Hu et al., 2021).

### Innovation and limitations

This study focuses on the impact of perceived school climate on exercise behavior, advancing relevant research in the fields of perceived school climate and exercise behavior. By constructing a dual mediation model and starting from the perspective of exercise psychology, it explores the relationship between obese adolescents’ perceived school climate and exercise behavior, which has practical significance in promoting their participation in physical exercise. Firstly, the impact of school climate on exercise behavior among obese adolescents was confirmed, and the relationship between perceived school climate and exercise behavior among obese adolescents was interpreted from the perspectives of perseverance qualities and exercise benefits. Secondly, the differences in mediating effects and specific indirect effects were examined.

There are also limitations in this study. Firstly, self-reporting may raise the correlation between variables and lead to common method biases. Therefore, the study tests for common method bias to avoid this limitation as much as possible, but there are still some uncontrollable factors that affect the research results, such as: self-expectation and participants’ response emotions. In the future, longitudinal tracking or experimental intervention designs can be applied to more effectively explain the impact of perceived school climate on exercise behavior among obese adolescents. Secondly, this study only examined the mediating effect of perseverance qualities and exercise benefits. Future studies will continue to examine variables closely related to exercise behavior, such as self-efficacy, sensory seeking and psychological resilience, in order to comprehensively reveal the relationship model between perceived school climate and physical activity behavior among obese adolescents.

## Conclusion

Obese adolescents’ perceived school climate positively predicts exercise behavior, perseverance qualities and exercise benefits. Perseverance qualities and exercise benefits positively predict exercise behavior among obese adolescents. Perseverance qualities and exercise benefits play a mediating role between perceived school climate and exercise behavior among obese adolescents, respectively. These two factors have parallel multiple mediating effects between obese adolescents’ perceived school climate and exercise behavior, with mediating effects accounting for 16 and 25%, respectively. The mediating effect of exercise benefits is greater than that of perseverance qualities. There is no difference in the test results of specific indirect effect between perseverance qualities and exercise benefits. Therefore, in the process of improving the exercise behavior among obese adolescents, it is recommended to start by strengthening their perception of the school climate, focusing on enhancing their perception of the exercise benefits as well as their willpower to persist in exercise. By improving their cognitive level of willpower and exercise benefits, it is recommended to promote the improvement of their exercise behavior, cultivate lifelong exercise awareness, and develop a healthy lifestyle.

## Data availability statement

The raw data supporting the conclusions of this article will be made available by the authors, without undue reservation.

## Ethics statement

The studies involving humans were approved by the Ethics Committee of Xiangya School of Public Health, Central South University. The studies were conducted in accordance with the local legislation and institutional requirements. Written informed consent for participation in this study was provided by the participants’ legal guardians/next of kin.

## Author contributions

YY contributed to the evaluation and interpretation of data, writing the first drafts, final version of the manuscript, and scrub data and maintain research data for initial use and later re-use. YQ contributed to the statistical analysis of data and development of the review concept, critically reviewed the manuscript, and participated in the interpretation and synthesis of data. CZ contributed to application of statistical to analyze and synthesize study data. CQ and ZC contributed to verification of the overall replication of results and other research outputs. All authors have read and agreed to the published version of the manuscript.
