# Supplementary material for: The impact of perceived school climate on exercise behavior engagement among obese adolescents: a dual mediation effect test of exercise benefits and perseverance qualities
Source: Front Psychol. 2023 Oct 3;14:1220362. doi: 10.3389/fpsyg.2023.1220362 (PMC10579602; doi:10.3389/fpsyg.2023.1220362)
Supplement: Supplementary file 1 [file Table_1.DOCX]

#### Informed Consent Form

Dear Student.

Hello! In order to promote your healthy development, we would like to invite you to participate in a Teaching Research Project of the People's Public Security University of China. Project name: The effect of perceived school climate on exercise behavioural engagement in obese adolescents: A dual mediated test of the benefits of exercise and the quality of perseverance.

Before you decide whether to participate in this study, please read the following as carefully as possible. It will help you to understand the study and why it is being conducted, the procedures, the benefits, risks and discomforts you may experience as a result of participating in the study.

**I. Purpose of the study**

This study examines the multiple mediating roles of perseverance qualities and exercise benefits between perceived school climate and exercise behavioural engagement in obese adolescents.

**II. What do you need to do if you participate in the study?**

1. You will be required to complete 3 paper version of the questionnaires.

**III. Benefits of participating in the trial**

1. We will understanding the relationship between obesity and physical activity.

2. We will learn how to improve health literacy.

**IV. Possible risks and discomforts**

During the study period, you will be required to complete the questionnaire entries, which may take up a small amount of your time and may cause you inconvenience.

**V. Confidentiality of personal information**

All information for this study is collected anonymously. All data collected will only be used for scientific research and will not be disclosed in any way to any entity or individual. Every effort will be made to protect the security of your personal data to the extent permitted by law.

If you have any questions about this study, you can ask any member of staff at the survey site and we will answer them as soon as possible. You can voluntarily choose whether or not to participate in this study, or to withdraw from the study at any time during the course of the study, without any loss to you.

I have read the above, fully understand the possible risks and benefits of participating in this study, and agree to participate in this study.

This informed consent has been reviewed by the Ethics Committee of Xiangya School of Public Health, Central South University (No.: _YGW-2022-44__, date of ethical review passed _ 20 July 2022__, effective date: __25 August 2022_).

□ Consent to participate in this study □ Refusal to participate in this study

Date： _______ ______ _____
