# Supplementary material for: The impact of perceived school climate on exercise behavior engagement among obese adolescents: a dual mediation effect test of exercise benefits and perseverance qualities
Source: Front Psychol. 2023 Oct 3;14:1220362. doi: 10.3389/fpsyg.2023.1220362 (PMC10579602; doi:10.3389/fpsyg.2023.1220362)
Supplement: Supplementary file 3 [file Table_3.DOCX]

**Questionnaire items on the impact of perceived school climate on exercise behavioural engagement among obese adolescents**

| **Title item** |  | **Never** | **Occasionally** | **Frequently** | **Always** |
| --- | --- | --- | --- | --- | --- |
| Teacher Support | | | | | |
| TS1 | I will discuss with the teachers at my school the problems that have occurred in my own. | □ | □ | □ | □ |
| TS2 | The teachers at my school care for me. | □ | □ | □ | □ |
| TS3 | The teachers at my school help students who have problems. | □ | □ | □ | □ |
| TS4 | The teachers at my school help students with school problems. | □ | □ | □ | □ |
| TS5 | My school's believe I can get things done. | □ | □ | □ | □ |
| TS6 | The teachers at my school worked hard to make sure I did well in my exams. | □ | □ | □ | □ |
| TS7 | The teachers at my school make me feel good about myself. | □ | □ | □ | □ |
| Classmate Support | | | | | |
| CS1 | The students at my school don't shout at each other. | □ | □ | □ | □ |
| CS2 | The students at my school don't get mean to each other. | □ | □ | □ | □ |
| CS3 | The students at my school will respect each other. | □ | □ | □ | □ |
| CS4 | The students at my school help each other out. | □ | □ | □ | □ |
| CS5 | No one at my school will beat me up or hurt me. | □ | □ | □ | □ |
| CS6 | The students at my school will like each other. | □ | □ | □ | □ |
| CS7 | Some students at my school will pick on other students. | □ | □ | □ | □ |
| CS8 | The students at my school care for each other. | □ | □ | □ | □ |
| CS9 | The students at my school have difficulty getting along with each other. | □ | □ | □ | □ |
| CS10 | I would worry that some of my classmates would hurt or bother me when I was at school. | □ | □ | □ | □ |
| CS11 | The students in my school did well. | □ | □ | □ | □ |
| CS12 | There are frequent fights between students in my school. | □ | □ | □ | □ |
| CS13 | The students at my school trust each other. | □ | □ | □ | □ |
| Opportunities for autonomy | | | | | |
| OA1 | Students from our school can assist with some of the rules involved. | □ | □ | □ | □ |
| 0A2 | Students from my school can assist with how class time is allocated. | □ | □ | □ | □ |
| OA3 | Students in our school are given the opportunity to make decisions in the classroom. | □ | □ | □ | □ |
| 0A4 | The teachers at my school ask the students what they want to learn. | □ | □ | □ | □ |
| OA5 | The students at my school have a say in how things work. | □ | □ | □ | □ |

| **Title item** |  | Strongly disagree | Disagree | Not necessarily | Agree | Couldn't agree more |
| --- | --- | --- | --- | --- | --- | --- |
| Exercise Benefits | | | | | | |
| EB1 | Exercise makes me feel clear-headed | □ | □ | □ | □ | □ |
| EB2 | Exercise will make me live longer | □ | □ | □ | □ | □ |
| EB3 | Exercise improves the overall function of my body | □ | □ | □ | □ | □ |
| EB4 | Exercise can prevent the onset of obesity | □ | □ | □ | □ | □ |
| EB5 | Exercise helps improve mental health | □ | □ | □ | □ | □ |
| Unrelenting Efforts | | | | | | |
| UE1 | I rarely feel depressed about losing weight | □ | □ | □ | □ | □ |
| UE2 | I will persevere to achieve my weight loss goal | □ | □ | □ | □ | □ |
| UE3 | I am very persistent and will not give up losing weight even when it is difficult | □ | □ | □ | □ | □ |
| UE4 | I will work hard to achieve my weight loss goal | □ | □ | □ | □ | □ |
| UE5 | I will persevere in the pursuit of my goals, even if it takes a long time to achieve them | □ | □ | □ | □ | □ |
| UE6 | I will give up other fun things for my weight loss goal | □ | □ | □ | □ | □ |
| Enduring Enthusiasm | | | | | | |
| EE1 | I will persevere in the pursuit of my weight loss goal, even in the face of challenges and difficulties | □ | □ | □ | □ | □ |
| EE2 | I will keep improving myself for my weight loss goal | □ | □ | □ | □ | □ |
| EE3 | I will suffer pain and discomfort for my weight loss goal | □ | □ | □ | □ | □ |
| EE4 | I will persevere in the face of setbacks | □ | □ | □ | □ | □ |
| EE5 | I will put a lot of time and effort into my weight loss goals | □ | □ | □ | □ | □ |
| EE6 | I will continue to find new ways and strategies for my weight loss goals | □ | □ | □ | □ | □ |
| **Exercise Behaviour** | | | | | | |
| EX1 How intensely do you do physical activity?  ① Light exercise (e.g. walking, doing gymnastics, playing gateball, etc.)  ②Low-intensity, less stressful exercise (e.g. recreational volleyball, table tennis, jogging, tai chi, etc.)  (iii) more vigorous and sustained exercise of moderate intensity (e.g. cycling, running, table tennis, etc.)  ④ Heavy, but not sustained, exercise with shortness of breath and a lot of sweating (e.g. playing badminton, basketball, tennis, football, etc.)  ⑤ Heavy, sustained exercise with shortness of breath and a lot of sweating (e.g. running, sets of aerobics exercises, swimming, etc.)  EX2 How many minutes at a time do you perform the above intensity physical activity?  ① Under 10 minutes  ②11 to 20 minutes  ③ 21 to 30 minutes  ④31 to 59 minutes  ⑤60 minutes or more  EX3 How many times a month do you do the above physical activities?  ①less than 1 time a month  ②3 to 5 times a week  ③ 2 to 3 times a month  ④ approximately 1 time per day  ⑤ 1 or 2 times a week  **Note: Intensity, frequency and time are graded from 1 to 5, with 1 to 5 points respectively.** | | | | | | |
