# Supplementary material for: The impact of perceived school climate on exercise behavior engagement among obese adolescents: a dual mediation effect test of exercise benefits and perseverance qualities
Source: Front Psychol. 2023 Oct 3;14:1220362. doi: 10.3389/fpsyg.2023.1220362 (PMC10579602; doi:10.3389/fpsyg.2023.1220362)
Supplement: Supplementary file 4 [file Data_Sheet_1.ZIP › Data analysis/Multi-mediator model construction results output.AmosOutput]

模型构建.amw


#### C:\Users\daboluo\Desktop\编辑部要求提交材料\中介模型构建\模型构建.amw

##### Analysis Summary

##### Date and Time

Date: 2023年5月16日

Time: 20:05:49

##### Title

模型构建: 2023年5月16日 20:05

##### Groups

##### Group number 1 (Group number 1)

##### Notes for Group (Group number 1)

The model is recursive.

Sample size = 586

##### Variable Summary (Group number 1)

##### Your model contains the following variables (Group number 1)

Observed, endogenous variables

EX1

EX2

EX3

EB5

EB4

EB3

EB2

EB1

TS7

TS6

TS5

TS4

TS3

TS2

TS1

CS13

CS12

CS11

CS10

CS9

CS8

CS7

CS6

CS5

CS4

CS3

CS2

CS1

OA5

OA4

OA3

OA2

OA1

UE1

UE2

UE3

UE4

UE5

UE6

EE1

EE2

EE3

EE4

EE5

EE6

Unobserved, endogenous variables

Exercise\_Behaviour

Classmate\_Support

Teacher\_Support

Exercise\_Benefits

Perseverance

Opportunities\_forAutonomy

Unrelenting\_Efforts

Enduring\_Enthusiasm

Unobserved, exogenous variables

Perceive\_SchoolClimate

e52

e53

e54

e51

e50

e49

e48

e47

e3

e1

e15

e14

e13

e12

e11

e10

e9

e29

e28

e27

e26

e25

e24

e23

e22

e21

e20

e19

e18

e16

e34

e33

e32

e31

e30

e35

e36

e37

e38

e39

e40

e41

e42

e43

e44

e45

e46

e6

e7

e8

e4

e5

e2

##### Variable counts (Group number 1)

|  |  |
| --- | --- |
| Number of variables in your model: | 107 |
| Number of observed variables: | 45 |
| Number of unobserved variables: | 62 |
| Number of exogenous variables: | 54 |
| Number of endogenous variables: | 53 |

##### Parameter Summary (Group number 1)

|  | Weights | Covariances | Variances | Means | Intercepts | Total |
| --- | --- | --- | --- | --- | --- | --- |
| Fixed | 62 | 0 | 0 | 0 | 0 | 62 |
| Labeled | 5 | 0 | 0 | 0 | 0 | 5 |
| Unlabeled | 41 | 0 | 54 | 0 | 0 | 95 |
| Total | 108 | 0 | 54 | 0 | 0 | 162 |

##### Models

##### Default model (Default model)

##### Notes for Model (Default model)

##### Computation of degrees of freedom (Default model)

|  |  |
| --- | --- |
| Number of distinct sample moments: | 1035 |
| Number of distinct parameters to be estimated: | 100 |
| Degrees of freedom (1035 - 100): | 935 |

##### Result (Default model)

Minimum was achieved

Chi-square = 1663.637

Degrees of freedom = 935

Probability level = .000

##### Group number 1 (Group number 1 - Default model)

##### Estimates (Group number 1 - Default model)

##### Scalar Estimates (Group number 1 - Default model)

##### Maximum Likelihood Estimates

##### Regression Weights: (Group number 1 - Default model)

|  |  |  | Estimate | S.E. | C.R. | P | Label |
| --- | --- | --- | --- | --- | --- | --- | --- |
| Perseverance | <--- | Perceive\_SchoolClimate | .348 | .112 | 3.108 | .002 | a |
| Exercise\_Benefits | <--- | Perceive\_SchoolClimate | .276 | .064 | 4.290 | \*\*\* | e |
| Exercise\_Behaviour | <--- | Perceive\_SchoolClimate | .330 | .115 | 2.876 | .004 | f |
| Classmate\_Support | <--- | Perceive\_SchoolClimate | 1.000 |  |
| Teacher\_Support | <--- | Perceive\_SchoolClimate | 1.118 | .114 | 9.788 | \*\*\* |  |
| Opportunities\_forAutonomy | <--- | Perceive\_SchoolClimate | 1.241 | .126 | 9.848 | \*\*\* |  |
| Unrelenting\_Efforts | <--- | Perseverance | 1.000 |  |
| Exercise\_Behaviour | <--- | Exercise\_Benefits | .512 | .120 | 4.275 | \*\*\* | c |
| Exercise\_Behaviour | <--- | Perseverance | .257 | .058 | 4.434 | \*\*\* | d |
| Enduring\_Enthusiasm | <--- | Perseverance | 1.178 | .190 | 6.203 | \*\*\* |  |
| EX1 | <--- | Exercise\_Behaviour | 1.000 |  |
| EX2 | <--- | Exercise\_Behaviour | 1.114 | .101 | 11.066 | \*\*\* |  |
| EX3 | <--- | Exercise\_Behaviour | 1.052 | .097 | 10.879 | \*\*\* |  |
| EB5 | <--- | Exercise\_Benefits | 1.000 |  |
| EB4 | <--- | Exercise\_Benefits | 1.103 | .125 | 8.801 | \*\*\* |  |
| EB3 | <--- | Exercise\_Benefits | .972 | .117 | 8.294 | \*\*\* |  |
| EB2 | <--- | Exercise\_Benefits | 1.085 | .123 | 8.807 | \*\*\* |  |
| EB1 | <--- | Exercise\_Benefits | 1.138 | .129 | 8.846 | \*\*\* |  |
| TS7 | <--- | Teacher\_Support | 1.000 |  |
| TS6 | <--- | Teacher\_Support | .787 | .028 | 27.923 | \*\*\* |  |
| TS5 | <--- | Teacher\_Support | .900 | .028 | 31.608 | \*\*\* |  |
| TS4 | <--- | Teacher\_Support | .882 | .028 | 31.259 | \*\*\* |  |
| TS3 | <--- | Teacher\_Support | .895 | .027 | 33.548 | \*\*\* |  |
| TS2 | <--- | Teacher\_Support | .904 | .027 | 32.906 | \*\*\* |  |
| TS1 | <--- | Teacher\_Support | .813 | .027 | 30.251 | \*\*\* |  |
| CS11 | <--- | Classmate\_Support | 1.054 | .028 | 37.825 | \*\*\* |  |
| CS10 | <--- | Classmate\_Support | 1.040 | .028 | 37.307 | \*\*\* |  |
| CS9 | <--- | Classmate\_Support | 1.079 | .028 | 38.161 | \*\*\* |  |
| CS8 | <--- | Classmate\_Support | 1.023 | .028 | 36.567 | \*\*\* |  |
| CS7 | <--- | Classmate\_Support | 1.000 |  |
| CS6 | <--- | Classmate\_Support | 1.036 | .028 | 37.518 | \*\*\* |  |
| CS5 | <--- | Classmate\_Support | 1.025 | .028 | 36.056 | \*\*\* |  |
| CS4 | <--- | Classmate\_Support | 1.019 | .026 | 38.771 | \*\*\* |  |
| CS3 | <--- | Classmate\_Support | 1.028 | .032 | 31.676 | \*\*\* |  |
| OA5 | <--- | Opportunities\_forAutonomy | 1.302 | .065 | 20.060 | \*\*\* |  |
| OA4 | <--- | Opportunities\_forAutonomy | .974 | .045 | 21.476 | \*\*\* |  |
| OA3 | <--- | Opportunities\_forAutonomy | 1.000 |  |
| OA2 | <--- | Opportunities\_forAutonomy | .953 | .051 | 18.609 | \*\*\* |  |
| OA1 | <--- | Opportunities\_forAutonomy | 1.009 | .047 | 21.341 | \*\*\* |  |
| UE1 | <--- | Unrelenting\_Efforts | 1.000 |  |
| UE2 | <--- | Unrelenting\_Efforts | 1.004 | .045 | 22.158 | \*\*\* |  |
| UE3 | <--- | Unrelenting\_Efforts | 1.124 | .048 | 23.505 | \*\*\* |  |
| UE4 | <--- | Unrelenting\_Efforts | 1.125 | .048 | 23.464 | \*\*\* |  |
| UE5 | <--- | Unrelenting\_Efforts | 1.061 | .046 | 23.146 | \*\*\* |  |
| UE6 | <--- | Unrelenting\_Efforts | 1.087 | .056 | 19.484 | \*\*\* |  |
| EE1 | <--- | Enduring\_Enthusiasm | 1.000 |  |
| EE2 | <--- | Enduring\_Enthusiasm | .978 | .039 | 25.397 | \*\*\* |  |
| EE3 | <--- | Enduring\_Enthusiasm | 1.036 | .040 | 26.218 | \*\*\* |  |
| EE4 | <--- | Enduring\_Enthusiasm | .995 | .039 | 25.757 | \*\*\* |  |
| EE5 | <--- | Enduring\_Enthusiasm | 1.020 | .039 | 26.042 | \*\*\* |  |
| EE6 | <--- | Enduring\_Enthusiasm | .961 | .043 | 22.430 | \*\*\* |  |
| CS1 | <--- | Classmate\_Support | 1.017 | .028 | 36.628 | \*\*\* |  |
| CS13 | <--- | Classmate\_Support | 1.204 | .040 | 29.842 | \*\*\* |  |
| CS12 | <--- | Classmate\_Support | 1.284 | .040 | 32.149 | \*\*\* |  |
| CS2 | <--- | Classmate\_Support | .991 | .029 | 34.716 | \*\*\* |  |

##### Standardized Regression Weights: (Group number 1 - Default model)

|  |  |  | Estimate |
| --- | --- | --- | --- |
| Perseverance | <--- | Perceive\_SchoolClimate | .176 |
| Exercise\_Benefits | <--- | Perceive\_SchoolClimate | .265 |
| Exercise\_Behaviour | <--- | Perceive\_SchoolClimate | .169 |
| Classmate\_Support | <--- | Perceive\_SchoolClimate | .671 |
| Teacher\_Support | <--- | Perceive\_SchoolClimate | .550 |
| Opportunities\_forAutonomy | <--- | Perceive\_SchoolClimate | .845 |
| Unrelenting\_Efforts | <--- | Perseverance | .820 |
| Exercise\_Behaviour | <--- | Exercise\_Benefits | .274 |
| Exercise\_Behaviour | <--- | Perseverance | .261 |
| Enduring\_Enthusiasm | <--- | Perseverance | .842 |
| EX1 | <--- | Exercise\_Behaviour | .645 |
| EX2 | <--- | Exercise\_Behaviour | .727 |
| EX3 | <--- | Exercise\_Behaviour | .643 |
| EB5 | <--- | Exercise\_Benefits | .504 |
| EB4 | <--- | Exercise\_Benefits | .598 |
| EB3 | <--- | Exercise\_Benefits | .531 |
| EB2 | <--- | Exercise\_Benefits | .599 |
| EB1 | <--- | Exercise\_Benefits | .605 |
| TS7 | <--- | Teacher\_Support | .850 |
| TS6 | <--- | Teacher\_Support | .863 |
| TS5 | <--- | Teacher\_Support | .920 |
| TS4 | <--- | Teacher\_Support | .915 |
| TS3 | <--- | Teacher\_Support | .946 |
| TS2 | <--- | Teacher\_Support | .937 |
| TS1 | <--- | Teacher\_Support | .900 |
| CS11 | <--- | Classmate\_Support | .915 |
| CS10 | <--- | Classmate\_Support | .911 |
| CS9 | <--- | Classmate\_Support | .918 |
| CS8 | <--- | Classmate\_Support | .905 |
| CS7 | <--- | Classmate\_Support | .911 |
| CS6 | <--- | Classmate\_Support | .913 |
| CS5 | <--- | Classmate\_Support | .900 |
| CS4 | <--- | Classmate\_Support | .923 |
| CS3 | <--- | Classmate\_Support | .856 |
| OA5 | <--- | Opportunities\_forAutonomy | .793 |
| OA4 | <--- | Opportunities\_forAutonomy | .841 |
| OA3 | <--- | Opportunities\_forAutonomy | .773 |
| OA2 | <--- | Opportunities\_forAutonomy | .744 |
| OA1 | <--- | Opportunities\_forAutonomy | .836 |
| UE1 | <--- | Unrelenting\_Efforts | .793 |
| UE2 | <--- | Unrelenting\_Efforts | .818 |
| UE3 | <--- | Unrelenting\_Efforts | .855 |
| UE4 | <--- | Unrelenting\_Efforts | .854 |
| UE5 | <--- | Unrelenting\_Efforts | .845 |
| UE6 | <--- | Unrelenting\_Efforts | .741 |
| EE1 | <--- | Enduring\_Enthusiasm | .835 |
| EE2 | <--- | Enduring\_Enthusiasm | .846 |
| EE3 | <--- | Enduring\_Enthusiasm | .863 |
| EE4 | <--- | Enduring\_Enthusiasm | .854 |
| EE5 | <--- | Enduring\_Enthusiasm | .860 |
| EE6 | <--- | Enduring\_Enthusiasm | .781 |
| CS1 | <--- | Classmate\_Support | .905 |
| CS13 | <--- | Classmate\_Support | .834 |
| CS12 | <--- | Classmate\_Support | .861 |
| CS2 | <--- | Classmate\_Support | .888 |

##### Variances: (Group number 1 - Default model)

|  |  |  | Estimate | S.E. | C.R. | P | Label |
| --- | --- | --- | --- | --- | --- | --- | --- |
| Perceive\_SchoolClimate |  |  | .139 | .020 | 6.998 | \*\*\* |  |
| e3 |  |  | .140 | .026 | 5.413 | \*\*\* |  |
| e1 |  |  | .528 | .098 | 5.391 | \*\*\* |  |
| e6 |  |  | .399 | .035 | 11.411 | \*\*\* |  |
| e7 |  |  | .170 | .017 | 10.166 | \*\*\* |  |
| e8 |  |  | .086 | .020 | 4.336 | \*\*\* |  |
| e4 |  |  | .265 | .086 | 3.066 | .002 |  |
| e5 |  |  | .309 | .118 | 2.623 | .009 |  |
| e2 |  |  | .411 | .060 | 6.858 | \*\*\* |  |
| e52 |  |  | .737 | .060 | 12.256 | \*\*\* |  |
| e53 |  |  | .581 | .061 | 9.569 | \*\*\* |  |
| e54 |  |  | .828 | .067 | 12.335 | \*\*\* |  |
| e51 |  |  | .443 | .030 | 14.785 | \*\*\* |  |
| e50 |  |  | .329 | .025 | 13.268 | \*\*\* |  |
| e49 |  |  | .362 | .025 | 14.414 | \*\*\* |  |
| e48 |  |  | .317 | .024 | 13.248 | \*\*\* |  |
| e47 |  |  | .338 | .026 | 13.122 | \*\*\* |  |
| e15 |  |  | .220 | .014 | 15.865 | \*\*\* |  |
| e14 |  |  | .122 | .008 | 15.720 | \*\*\* |  |
| e13 |  |  | .085 | .006 | 14.484 | \*\*\* |  |
| e12 |  |  | .087 | .006 | 14.658 | \*\*\* |  |
| e11 |  |  | .054 | .004 | 13.038 | \*\*\* |  |
| e10 |  |  | .065 | .005 | 13.632 | \*\*\* |  |
| e9 |  |  | .089 | .006 | 15.075 | \*\*\* |  |
| e29 |  |  | .196 | .012 | 16.407 | \*\*\* |  |
| e28 |  |  | .177 | .011 | 16.229 | \*\*\* |  |
| e27 |  |  | .067 | .004 | 15.531 | \*\*\* |  |
| e26 |  |  | .069 | .004 | 15.617 | \*\*\* |  |
| e25 |  |  | .067 | .004 | 15.471 | \*\*\* |  |
| e24 |  |  | .072 | .005 | 15.730 | \*\*\* |  |
| e23 |  |  | .064 | .004 | 15.623 | \*\*\* |  |
| e22 |  |  | .066 | .004 | 15.583 | \*\*\* |  |
| e21 |  |  | .076 | .005 | 15.802 | \*\*\* |  |
| e20 |  |  | .056 | .004 | 15.356 | \*\*\* |  |
| e19 |  |  | .119 | .007 | 16.269 | \*\*\* |  |
| e18 |  |  | .082 | .005 | 15.971 | \*\*\* |  |
| e16 |  |  | .071 | .004 | 15.721 | \*\*\* |  |
| e34 |  |  | .300 | .021 | 14.088 | \*\*\* |  |
| e33 |  |  | .118 | .009 | 12.789 | \*\*\* |  |
| e32 |  |  | .202 | .014 | 14.473 | \*\*\* |  |
| e31 |  |  | .220 | .015 | 14.913 | \*\*\* |  |
| e30 |  |  | .131 | .010 | 12.946 | \*\*\* |  |
| e35 |  |  | .476 | .032 | 14.951 | \*\*\* |  |
| e36 |  |  | .403 | .028 | 14.532 | \*\*\* |  |
| e37 |  |  | .376 | .028 | 13.642 | \*\*\* |  |
| e38 |  |  | .381 | .028 | 13.675 | \*\*\* |  |
| e39 |  |  | .364 | .026 | 13.918 | \*\*\* |  |
| e40 |  |  | .785 | .050 | 15.567 | \*\*\* |  |
| e41 |  |  | .463 | .032 | 14.551 | \*\*\* |  |
| e42 |  |  | .404 | .028 | 14.299 | \*\*\* |  |
| e43 |  |  | .392 | .028 | 13.857 | \*\*\* |  |
| e44 |  |  | .392 | .028 | 14.116 | \*\*\* |  |
| e45 |  |  | .392 | .028 | 13.959 | \*\*\* |  |
| e46 |  |  | .627 | .041 | 15.373 | \*\*\* |  |

##### Squared Multiple Correlations: (Group number 1 - Default model)

|  |  |  | Estimate |
| --- | --- | --- | --- |
| Perseverance |  |  | .031 |
| Exercise\_Benefits |  |  | .070 |
| Enduring\_Enthusiasm |  |  | .710 |
| Unrelenting\_Efforts |  |  | .673 |
| Opportunities\_forAutonomy |  |  | .713 |
| Teacher\_Support |  |  | .303 |
| Classmate\_Support |  |  | .450 |
| Exercise\_Behaviour |  |  | .218 |
| EE6 |  |  | .610 |
| EE5 |  |  | .739 |
| EE4 |  |  | .729 |
| EE3 |  |  | .745 |
| EE2 |  |  | .716 |
| EE1 |  |  | .697 |
| UE6 |  |  | .549 |
| UE5 |  |  | .714 |
| UE4 |  |  | .729 |
| UE3 |  |  | .731 |
| UE2 |  |  | .669 |
| UE1 |  |  | .629 |
| OA1 |  |  | .699 |
| OA2 |  |  | .553 |
| OA3 |  |  | .597 |
| OA4 |  |  | .707 |
| OA5 |  |  | .629 |
| CS1 |  |  | .819 |
| CS2 |  |  | .788 |
| CS3 |  |  | .732 |
| CS4 |  |  | .851 |
| CS5 |  |  | .810 |
| CS6 |  |  | .833 |
| CS7 |  |  | .829 |
| CS8 |  |  | .818 |
| CS9 |  |  | .842 |
| CS10 |  |  | .830 |
| CS11 |  |  | .837 |
| CS12 |  |  | .742 |
| CS13 |  |  | .695 |
| TS1 |  |  | .810 |
| TS2 |  |  | .878 |
| TS3 |  |  | .894 |
| TS4 |  |  | .836 |
| TS5 |  |  | .845 |
| TS6 |  |  | .744 |
| TS7 |  |  | .723 |
| EB1 |  |  | .366 |
| EB2 |  |  | .359 |
| EB3 |  |  | .282 |
| EB4 |  |  | .358 |
| EB5 |  |  | .254 |
| EX3 |  |  | .413 |
| EX2 |  |  | .529 |
| EX1 |  |  | .416 |

##### User-defined estimands: (Group number 1 - Default model)

|  |  |  |  |
| --- | --- | --- | --- |
| Estimand 1 |  |  | .089 |
| Estimand 2 |  |  | .141 |
| Estimand 3 |  |  | .330 |
| Estimand 4 |  |  | -.052 |
| Estimand 5 |  |  | .560 |

##### Matrices (Group number 1 - Default model)

##### Total Effects (Group number 1 - Default model)

|  | Perceive\_SchoolClimate | Perseverance | Exercise\_Benefits | Enduring\_Enthusiasm | Unrelenting\_Efforts | Opportunities\_forAutonomy | Teacher\_Support | Classmate\_Support | Exercise\_Behaviour |
| --- | --- | --- | --- | --- | --- | --- | --- | --- | --- |
| Perseverance | .348 | .000 | .000 | .000 | .000 | .000 | .000 | .000 | .000 |
| Exercise\_Benefits | .276 | .000 | .000 | .000 | .000 | .000 | .000 | .000 | .000 |
| Enduring\_Enthusiasm | .410 | 1.178 | .000 | .000 | .000 | .000 | .000 | .000 | .000 |
| Unrelenting\_Efforts | .348 | 1.000 | .000 | .000 | .000 | .000 | .000 | .000 | .000 |
| Opportunities\_forAutonomy | 1.241 | .000 | .000 | .000 | .000 | .000 | .000 | .000 | .000 |
| Teacher\_Support | 1.118 | .000 | .000 | .000 | .000 | .000 | .000 | .000 | .000 |
| Classmate\_Support | 1.000 | .000 | .000 | .000 | .000 | .000 | .000 | .000 | .000 |
| Exercise\_Behaviour | .560 | .257 | .512 | .000 | .000 | .000 | .000 | .000 | .000 |
| EE6 | .394 | 1.132 | .000 | .961 | .000 | .000 | .000 | .000 | .000 |
| EE5 | .419 | 1.202 | .000 | 1.020 | .000 | .000 | .000 | .000 | .000 |
| EE4 | .408 | 1.172 | .000 | .995 | .000 | .000 | .000 | .000 | .000 |
| EE3 | .425 | 1.221 | .000 | 1.036 | .000 | .000 | .000 | .000 | .000 |
| EE2 | .401 | 1.153 | .000 | .978 | .000 | .000 | .000 | .000 | .000 |
| EE1 | .410 | 1.178 | .000 | 1.000 | .000 | .000 | .000 | .000 | .000 |
| UE6 | .378 | 1.087 | .000 | .000 | 1.087 | .000 | .000 | .000 | .000 |
| UE5 | .370 | 1.061 | .000 | .000 | 1.061 | .000 | .000 | .000 | .000 |
| UE4 | .392 | 1.125 | .000 | .000 | 1.125 | .000 | .000 | .000 | .000 |
| UE3 | .391 | 1.124 | .000 | .000 | 1.124 | .000 | .000 | .000 | .000 |
| UE2 | .349 | 1.004 | .000 | .000 | 1.004 | .000 | .000 | .000 | .000 |
| UE1 | .348 | 1.000 | .000 | .000 | 1.000 | .000 | .000 | .000 | .000 |
| OA1 | 1.252 | .000 | .000 | .000 | .000 | 1.009 | .000 | .000 | .000 |
| OA2 | 1.183 | .000 | .000 | .000 | .000 | .953 | .000 | .000 | .000 |
| OA3 | 1.241 | .000 | .000 | .000 | .000 | 1.000 | .000 | .000 | .000 |
| OA4 | 1.209 | .000 | .000 | .000 | .000 | .974 | .000 | .000 | .000 |
| OA5 | 1.616 | .000 | .000 | .000 | .000 | 1.302 | .000 | .000 | .000 |
| CS1 | 1.017 | .000 | .000 | .000 | .000 | .000 | .000 | 1.017 | .000 |
| CS2 | .991 | .000 | .000 | .000 | .000 | .000 | .000 | .991 | .000 |
| CS3 | 1.028 | .000 | .000 | .000 | .000 | .000 | .000 | 1.028 | .000 |
| CS4 | 1.019 | .000 | .000 | .000 | .000 | .000 | .000 | 1.019 | .000 |
| CS5 | 1.025 | .000 | .000 | .000 | .000 | .000 | .000 | 1.025 | .000 |
| CS6 | 1.036 | .000 | .000 | .000 | .000 | .000 | .000 | 1.036 | .000 |
| CS7 | 1.000 | .000 | .000 | .000 | .000 | .000 | .000 | 1.000 | .000 |
| CS8 | 1.023 | .000 | .000 | .000 | .000 | .000 | .000 | 1.023 | .000 |
| CS9 | 1.079 | .000 | .000 | .000 | .000 | .000 | .000 | 1.079 | .000 |
| CS10 | 1.040 | .000 | .000 | .000 | .000 | .000 | .000 | 1.040 | .000 |
| CS11 | 1.054 | .000 | .000 | .000 | .000 | .000 | .000 | 1.054 | .000 |
| CS12 | 1.284 | .000 | .000 | .000 | .000 | .000 | .000 | 1.284 | .000 |
| CS13 | 1.204 | .000 | .000 | .000 | .000 | .000 | .000 | 1.204 | .000 |
| TS1 | .909 | .000 | .000 | .000 | .000 | .000 | .813 | .000 | .000 |
| TS2 | 1.011 | .000 | .000 | .000 | .000 | .000 | .904 | .000 | .000 |
| TS3 | 1.000 | .000 | .000 | .000 | .000 | .000 | .895 | .000 | .000 |
| TS4 | .986 | .000 | .000 | .000 | .000 | .000 | .882 | .000 | .000 |
| TS5 | 1.007 | .000 | .000 | .000 | .000 | .000 | .900 | .000 | .000 |
| TS6 | .879 | .000 | .000 | .000 | .000 | .000 | .787 | .000 | .000 |
| TS7 | 1.118 | .000 | .000 | .000 | .000 | .000 | 1.000 | .000 | .000 |
| EB1 | .314 | .000 | 1.138 | .000 | .000 | .000 | .000 | .000 | .000 |
| EB2 | .299 | .000 | 1.085 | .000 | .000 | .000 | .000 | .000 | .000 |
| EB3 | .268 | .000 | .972 | .000 | .000 | .000 | .000 | .000 | .000 |
| EB4 | .304 | .000 | 1.103 | .000 | .000 | .000 | .000 | .000 | .000 |
| EB5 | .276 | .000 | 1.000 | .000 | .000 | .000 | .000 | .000 | .000 |
| EX3 | .589 | .270 | .538 | .000 | .000 | .000 | .000 | .000 | 1.052 |
| EX2 | .624 | .286 | .570 | .000 | .000 | .000 | .000 | .000 | 1.114 |
| EX1 | .560 | .257 | .512 | .000 | .000 | .000 | .000 | .000 | 1.000 |

##### Standardized Total Effects (Group number 1 - Default model)

|  | Perceive\_SchoolClimate | Perseverance | Exercise\_Benefits | Enduring\_Enthusiasm | Unrelenting\_Efforts | Opportunities\_forAutonomy | Teacher\_Support | Classmate\_Support | Exercise\_Behaviour |
| --- | --- | --- | --- | --- | --- | --- | --- | --- | --- |
| Perseverance | .176 | .000 | .000 | .000 | .000 | .000 | .000 | .000 | .000 |
| Exercise\_Benefits | .265 | .000 | .000 | .000 | .000 | .000 | .000 | .000 | .000 |
| Enduring\_Enthusiasm | .148 | .842 | .000 | .000 | .000 | .000 | .000 | .000 | .000 |
| Unrelenting\_Efforts | .144 | .820 | .000 | .000 | .000 | .000 | .000 | .000 | .000 |
| Opportunities\_forAutonomy | .845 | .000 | .000 | .000 | .000 | .000 | .000 | .000 | .000 |
| Teacher\_Support | .550 | .000 | .000 | .000 | .000 | .000 | .000 | .000 | .000 |
| Classmate\_Support | .671 | .000 | .000 | .000 | .000 | .000 | .000 | .000 | .000 |
| Exercise\_Behaviour | .288 | .261 | .274 | .000 | .000 | .000 | .000 | .000 | .000 |
| EE6 | .116 | .658 | .000 | .781 | .000 | .000 | .000 | .000 | .000 |
| EE5 | .127 | .724 | .000 | .860 | .000 | .000 | .000 | .000 | .000 |
| EE4 | .126 | .719 | .000 | .854 | .000 | .000 | .000 | .000 | .000 |
| EE3 | .128 | .727 | .000 | .863 | .000 | .000 | .000 | .000 | .000 |
| EE2 | .125 | .713 | .000 | .846 | .000 | .000 | .000 | .000 | .000 |
| EE1 | .124 | .703 | .000 | .835 | .000 | .000 | .000 | .000 | .000 |
| UE6 | .107 | .608 | .000 | .000 | .741 | .000 | .000 | .000 | .000 |
| UE5 | .122 | .693 | .000 | .000 | .845 | .000 | .000 | .000 | .000 |
| UE4 | .123 | .700 | .000 | .000 | .854 | .000 | .000 | .000 | .000 |
| UE3 | .123 | .701 | .000 | .000 | .855 | .000 | .000 | .000 | .000 |
| UE2 | .118 | .671 | .000 | .000 | .818 | .000 | .000 | .000 | .000 |
| UE1 | .114 | .651 | .000 | .000 | .793 | .000 | .000 | .000 | .000 |
| OA1 | .706 | .000 | .000 | .000 | .000 | .836 | .000 | .000 | .000 |
| OA2 | .628 | .000 | .000 | .000 | .000 | .744 | .000 | .000 | .000 |
| OA3 | .653 | .000 | .000 | .000 | .000 | .773 | .000 | .000 | .000 |
| OA4 | .710 | .000 | .000 | .000 | .000 | .841 | .000 | .000 | .000 |
| OA5 | .670 | .000 | .000 | .000 | .000 | .793 | .000 | .000 | .000 |
| CS1 | .607 | .000 | .000 | .000 | .000 | .000 | .000 | .905 | .000 |
| CS2 | .595 | .000 | .000 | .000 | .000 | .000 | .000 | .888 | .000 |
| CS3 | .574 | .000 | .000 | .000 | .000 | .000 | .000 | .856 | .000 |
| CS4 | .619 | .000 | .000 | .000 | .000 | .000 | .000 | .923 | .000 |
| CS5 | .604 | .000 | .000 | .000 | .000 | .000 | .000 | .900 | .000 |
| CS6 | .612 | .000 | .000 | .000 | .000 | .000 | .000 | .913 | .000 |
| CS7 | .611 | .000 | .000 | .000 | .000 | .000 | .000 | .911 | .000 |
| CS8 | .607 | .000 | .000 | .000 | .000 | .000 | .000 | .905 | .000 |
| CS9 | .616 | .000 | .000 | .000 | .000 | .000 | .000 | .918 | .000 |
| CS10 | .611 | .000 | .000 | .000 | .000 | .000 | .000 | .911 | .000 |
| CS11 | .614 | .000 | .000 | .000 | .000 | .000 | .000 | .915 | .000 |
| CS12 | .578 | .000 | .000 | .000 | .000 | .000 | .000 | .861 | .000 |
| CS13 | .559 | .000 | .000 | .000 | .000 | .000 | .000 | .834 | .000 |
| TS1 | .495 | .000 | .000 | .000 | .000 | .000 | .900 | .000 | .000 |
| TS2 | .516 | .000 | .000 | .000 | .000 | .000 | .937 | .000 | .000 |
| TS3 | .520 | .000 | .000 | .000 | .000 | .000 | .946 | .000 | .000 |
| TS4 | .503 | .000 | .000 | .000 | .000 | .000 | .915 | .000 | .000 |
| TS5 | .506 | .000 | .000 | .000 | .000 | .000 | .920 | .000 | .000 |
| TS6 | .475 | .000 | .000 | .000 | .000 | .000 | .863 | .000 | .000 |
| TS7 | .468 | .000 | .000 | .000 | .000 | .000 | .850 | .000 | .000 |
| EB1 | .160 | .000 | .605 | .000 | .000 | .000 | .000 | .000 | .000 |
| EB2 | .159 | .000 | .599 | .000 | .000 | .000 | .000 | .000 | .000 |
| EB3 | .141 | .000 | .531 | .000 | .000 | .000 | .000 | .000 | .000 |
| EB4 | .158 | .000 | .598 | .000 | .000 | .000 | .000 | .000 | .000 |
| EB5 | .133 | .000 | .504 | .000 | .000 | .000 | .000 | .000 | .000 |
| EX3 | .185 | .168 | .176 | .000 | .000 | .000 | .000 | .000 | .643 |
| EX2 | .209 | .190 | .199 | .000 | .000 | .000 | .000 | .000 | .727 |
| EX1 | .186 | .169 | .177 | .000 | .000 | .000 | .000 | .000 | .645 |

##### Direct Effects (Group number 1 - Default model)

|  | Perceive\_SchoolClimate | Perseverance | Exercise\_Benefits | Enduring\_Enthusiasm | Unrelenting\_Efforts | Opportunities\_forAutonomy | Teacher\_Support | Classmate\_Support | Exercise\_Behaviour |
| --- | --- | --- | --- | --- | --- | --- | --- | --- | --- |
| Perseverance | .348 | .000 | .000 | .000 | .000 | .000 | .000 | .000 | .000 |
| Exercise\_Benefits | .276 | .000 | .000 | .000 | .000 | .000 | .000 | .000 | .000 |
| Enduring\_Enthusiasm | .000 | 1.178 | .000 | .000 | .000 | .000 | .000 | .000 | .000 |
| Unrelenting\_Efforts | .000 | 1.000 | .000 | .000 | .000 | .000 | .000 | .000 | .000 |
| Opportunities\_forAutonomy | 1.241 | .000 | .000 | .000 | .000 | .000 | .000 | .000 | .000 |
| Teacher\_Support | 1.118 | .000 | .000 | .000 | .000 | .000 | .000 | .000 | .000 |
| Classmate\_Support | 1.000 | .000 | .000 | .000 | .000 | .000 | .000 | .000 | .000 |
| Exercise\_Behaviour | .330 | .257 | .512 | .000 | .000 | .000 | .000 | .000 | .000 |
| EE6 | .000 | .000 | .000 | .961 | .000 | .000 | .000 | .000 | .000 |
| EE5 | .000 | .000 | .000 | 1.020 | .000 | .000 | .000 | .000 | .000 |
| EE4 | .000 | .000 | .000 | .995 | .000 | .000 | .000 | .000 | .000 |
| EE3 | .000 | .000 | .000 | 1.036 | .000 | .000 | .000 | .000 | .000 |
| EE2 | .000 | .000 | .000 | .978 | .000 | .000 | .000 | .000 | .000 |
| EE1 | .000 | .000 | .000 | 1.000 | .000 | .000 | .000 | .000 | .000 |
| UE6 | .000 | .000 | .000 | .000 | 1.087 | .000 | .000 | .000 | .000 |
| UE5 | .000 | .000 | .000 | .000 | 1.061 | .000 | .000 | .000 | .000 |
| UE4 | .000 | .000 | .000 | .000 | 1.125 | .000 | .000 | .000 | .000 |
| UE3 | .000 | .000 | .000 | .000 | 1.124 | .000 | .000 | .000 | .000 |
| UE2 | .000 | .000 | .000 | .000 | 1.004 | .000 | .000 | .000 | .000 |
| UE1 | .000 | .000 | .000 | .000 | 1.000 | .000 | .000 | .000 | .000 |
| OA1 | .000 | .000 | .000 | .000 | .000 | 1.009 | .000 | .000 | .000 |
| OA2 | .000 | .000 | .000 | .000 | .000 | .953 | .000 | .000 | .000 |
| OA3 | .000 | .000 | .000 | .000 | .000 | 1.000 | .000 | .000 | .000 |
| OA4 | .000 | .000 | .000 | .000 | .000 | .974 | .000 | .000 | .000 |
| OA5 | .000 | .000 | .000 | .000 | .000 | 1.302 | .000 | .000 | .000 |
| CS1 | .000 | .000 | .000 | .000 | .000 | .000 | .000 | 1.017 | .000 |
| CS2 | .000 | .000 | .000 | .000 | .000 | .000 | .000 | .991 | .000 |
| CS3 | .000 | .000 | .000 | .000 | .000 | .000 | .000 | 1.028 | .000 |
| CS4 | .000 | .000 | .000 | .000 | .000 | .000 | .000 | 1.019 | .000 |
| CS5 | .000 | .000 | .000 | .000 | .000 | .000 | .000 | 1.025 | .000 |
| CS6 | .000 | .000 | .000 | .000 | .000 | .000 | .000 | 1.036 | .000 |
| CS7 | .000 | .000 | .000 | .000 | .000 | .000 | .000 | 1.000 | .000 |
| CS8 | .000 | .000 | .000 | .000 | .000 | .000 | .000 | 1.023 | .000 |
| CS9 | .000 | .000 | .000 | .000 | .000 | .000 | .000 | 1.079 | .000 |
| CS10 | .000 | .000 | .000 | .000 | .000 | .000 | .000 | 1.040 | .000 |
| CS11 | .000 | .000 | .000 | .000 | .000 | .000 | .000 | 1.054 | .000 |
| CS12 | .000 | .000 | .000 | .000 | .000 | .000 | .000 | 1.284 | .000 |
| CS13 | .000 | .000 | .000 | .000 | .000 | .000 | .000 | 1.204 | .000 |
| TS1 | .000 | .000 | .000 | .000 | .000 | .000 | .813 | .000 | .000 |
| TS2 | .000 | .000 | .000 | .000 | .000 | .000 | .904 | .000 | .000 |
| TS3 | .000 | .000 | .000 | .000 | .000 | .000 | .895 | .000 | .000 |
| TS4 | .000 | .000 | .000 | .000 | .000 | .000 | .882 | .000 | .000 |
| TS5 | .000 | .000 | .000 | .000 | .000 | .000 | .900 | .000 | .000 |
| TS6 | .000 | .000 | .000 | .000 | .000 | .000 | .787 | .000 | .000 |
| TS7 | .000 | .000 | .000 | .000 | .000 | .000 | 1.000 | .000 | .000 |
| EB1 | .000 | .000 | 1.138 | .000 | .000 | .000 | .000 | .000 | .000 |
| EB2 | .000 | .000 | 1.085 | .000 | .000 | .000 | .000 | .000 | .000 |
| EB3 | .000 | .000 | .972 | .000 | .000 | .000 | .000 | .000 | .000 |
| EB4 | .000 | .000 | 1.103 | .000 | .000 | .000 | .000 | .000 | .000 |
| EB5 | .000 | .000 | 1.000 | .000 | .000 | .000 | .000 | .000 | .000 |
| EX3 | .000 | .000 | .000 | .000 | .000 | .000 | .000 | .000 | 1.052 |
| EX2 | .000 | .000 | .000 | .000 | .000 | .000 | .000 | .000 | 1.114 |
| EX1 | .000 | .000 | .000 | .000 | .000 | .000 | .000 | .000 | 1.000 |

##### Standardized Direct Effects (Group number 1 - Default model)

|  | Perceive\_SchoolClimate | Perseverance | Exercise\_Benefits | Enduring\_Enthusiasm | Unrelenting\_Efforts | Opportunities\_forAutonomy | Teacher\_Support | Classmate\_Support | Exercise\_Behaviour |
| --- | --- | --- | --- | --- | --- | --- | --- | --- | --- |
| Perseverance | .176 | .000 | .000 | .000 | .000 | .000 | .000 | .000 | .000 |
| Exercise\_Benefits | .265 | .000 | .000 | .000 | .000 | .000 | .000 | .000 | .000 |
| Enduring\_Enthusiasm | .000 | .842 | .000 | .000 | .000 | .000 | .000 | .000 | .000 |
| Unrelenting\_Efforts | .000 | .820 | .000 | .000 | .000 | .000 | .000 | .000 | .000 |
| Opportunities\_forAutonomy | .845 | .000 | .000 | .000 | .000 | .000 | .000 | .000 | .000 |
| Teacher\_Support | .550 | .000 | .000 | .000 | .000 | .000 | .000 | .000 | .000 |
| Classmate\_Support | .671 | .000 | .000 | .000 | .000 | .000 | .000 | .000 | .000 |
| Exercise\_Behaviour | .169 | .261 | .274 | .000 | .000 | .000 | .000 | .000 | .000 |
| EE6 | .000 | .000 | .000 | .781 | .000 | .000 | .000 | .000 | .000 |
| EE5 | .000 | .000 | .000 | .860 | .000 | .000 | .000 | .000 | .000 |
| EE4 | .000 | .000 | .000 | .854 | .000 | .000 | .000 | .000 | .000 |
| EE3 | .000 | .000 | .000 | .863 | .000 | .000 | .000 | .000 | .000 |
| EE2 | .000 | .000 | .000 | .846 | .000 | .000 | .000 | .000 | .000 |
| EE1 | .000 | .000 | .000 | .835 | .000 | .000 | .000 | .000 | .000 |
| UE6 | .000 | .000 | .000 | .000 | .741 | .000 | .000 | .000 | .000 |
| UE5 | .000 | .000 | .000 | .000 | .845 | .000 | .000 | .000 | .000 |
| UE4 | .000 | .000 | .000 | .000 | .854 | .000 | .000 | .000 | .000 |
| UE3 | .000 | .000 | .000 | .000 | .855 | .000 | .000 | .000 | .000 |
| UE2 | .000 | .000 | .000 | .000 | .818 | .000 | .000 | .000 | .000 |
| UE1 | .000 | .000 | .000 | .000 | .793 | .000 | .000 | .000 | .000 |
| OA1 | .000 | .000 | .000 | .000 | .000 | .836 | .000 | .000 | .000 |
| OA2 | .000 | .000 | .000 | .000 | .000 | .744 | .000 | .000 | .000 |
| OA3 | .000 | .000 | .000 | .000 | .000 | .773 | .000 | .000 | .000 |
| OA4 | .000 | .000 | .000 | .000 | .000 | .841 | .000 | .000 | .000 |
| OA5 | .000 | .000 | .000 | .000 | .000 | .793 | .000 | .000 | .000 |
| CS1 | .000 | .000 | .000 | .000 | .000 | .000 | .000 | .905 | .000 |
| CS2 | .000 | .000 | .000 | .000 | .000 | .000 | .000 | .888 | .000 |
| CS3 | .000 | .000 | .000 | .000 | .000 | .000 | .000 | .856 | .000 |
| CS4 | .000 | .000 | .000 | .000 | .000 | .000 | .000 | .923 | .000 |
| CS5 | .000 | .000 | .000 | .000 | .000 | .000 | .000 | .900 | .000 |
| CS6 | .000 | .000 | .000 | .000 | .000 | .000 | .000 | .913 | .000 |
| CS7 | .000 | .000 | .000 | .000 | .000 | .000 | .000 | .911 | .000 |
| CS8 | .000 | .000 | .000 | .000 | .000 | .000 | .000 | .905 | .000 |
| CS9 | .000 | .000 | .000 | .000 | .000 | .000 | .000 | .918 | .000 |
| CS10 | .000 | .000 | .000 | .000 | .000 | .000 | .000 | .911 | .000 |
| CS11 | .000 | .000 | .000 | .000 | .000 | .000 | .000 | .915 | .000 |
| CS12 | .000 | .000 | .000 | .000 | .000 | .000 | .000 | .861 | .000 |
| CS13 | .000 | .000 | .000 | .000 | .000 | .000 | .000 | .834 | .000 |
| TS1 | .000 | .000 | .000 | .000 | .000 | .000 | .900 | .000 | .000 |
| TS2 | .000 | .000 | .000 | .000 | .000 | .000 | .937 | .000 | .000 |
| TS3 | .000 | .000 | .000 | .000 | .000 | .000 | .946 | .000 | .000 |
| TS4 | .000 | .000 | .000 | .000 | .000 | .000 | .915 | .000 | .000 |
| TS5 | .000 | .000 | .000 | .000 | .000 | .000 | .920 | .000 | .000 |
| TS6 | .000 | .000 | .000 | .000 | .000 | .000 | .863 | .000 | .000 |
| TS7 | .000 | .000 | .000 | .000 | .000 | .000 | .850 | .000 | .000 |
| EB1 | .000 | .000 | .605 | .000 | .000 | .000 | .000 | .000 | .000 |
| EB2 | .000 | .000 | .599 | .000 | .000 | .000 | .000 | .000 | .000 |
| EB3 | .000 | .000 | .531 | .000 | .000 | .000 | .000 | .000 | .000 |
| EB4 | .000 | .000 | .598 | .000 | .000 | .000 | .000 | .000 | .000 |
| EB5 | .000 | .000 | .504 | .000 | .000 | .000 | .000 | .000 | .000 |
| EX3 | .000 | .000 | .000 | .000 | .000 | .000 | .000 | .000 | .643 |
| EX2 | .000 | .000 | .000 | .000 | .000 | .000 | .000 | .000 | .727 |
| EX1 | .000 | .000 | .000 | .000 | .000 | .000 | .000 | .000 | .645 |

##### Indirect Effects (Group number 1 - Default model)

|  | Perceive\_SchoolClimate | Perseverance | Exercise\_Benefits | Enduring\_Enthusiasm | Unrelenting\_Efforts | Opportunities\_forAutonomy | Teacher\_Support | Classmate\_Support | Exercise\_Behaviour |
| --- | --- | --- | --- | --- | --- | --- | --- | --- | --- |
| Perseverance | .000 | .000 | .000 | .000 | .000 | .000 | .000 | .000 | .000 |
| Exercise\_Benefits | .000 | .000 | .000 | .000 | .000 | .000 | .000 | .000 | .000 |
| Enduring\_Enthusiasm | .410 | .000 | .000 | .000 | .000 | .000 | .000 | .000 | .000 |
| Unrelenting\_Efforts | .348 | .000 | .000 | .000 | .000 | .000 | .000 | .000 | .000 |
| Opportunities\_forAutonomy | .000 | .000 | .000 | .000 | .000 | .000 | .000 | .000 | .000 |
| Teacher\_Support | .000 | .000 | .000 | .000 | .000 | .000 | .000 | .000 | .000 |
| Classmate\_Support | .000 | .000 | .000 | .000 | .000 | .000 | .000 | .000 | .000 |
| Exercise\_Behaviour | .231 | .000 | .000 | .000 | .000 | .000 | .000 | .000 | .000 |
| EE6 | .394 | 1.132 | .000 | .000 | .000 | .000 | .000 | .000 | .000 |
| EE5 | .419 | 1.202 | .000 | .000 | .000 | .000 | .000 | .000 | .000 |
| EE4 | .408 | 1.172 | .000 | .000 | .000 | .000 | .000 | .000 | .000 |
| EE3 | .425 | 1.221 | .000 | .000 | .000 | .000 | .000 | .000 | .000 |
| EE2 | .401 | 1.153 | .000 | .000 | .000 | .000 | .000 | .000 | .000 |
| EE1 | .410 | 1.178 | .000 | .000 | .000 | .000 | .000 | .000 | .000 |
| UE6 | .378 | 1.087 | .000 | .000 | .000 | .000 | .000 | .000 | .000 |
| UE5 | .370 | 1.061 | .000 | .000 | .000 | .000 | .000 | .000 | .000 |
| UE4 | .392 | 1.125 | .000 | .000 | .000 | .000 | .000 | .000 | .000 |
| UE3 | .391 | 1.124 | .000 | .000 | .000 | .000 | .000 | .000 | .000 |
| UE2 | .349 | 1.004 | .000 | .000 | .000 | .000 | .000 | .000 | .000 |
| UE1 | .348 | 1.000 | .000 | .000 | .000 | .000 | .000 | .000 | .000 |
| OA1 | 1.252 | .000 | .000 | .000 | .000 | .000 | .000 | .000 | .000 |
| OA2 | 1.183 | .000 | .000 | .000 | .000 | .000 | .000 | .000 | .000 |
| OA3 | 1.241 | .000 | .000 | .000 | .000 | .000 | .000 | .000 | .000 |
| OA4 | 1.209 | .000 | .000 | .000 | .000 | .000 | .000 | .000 | .000 |
| OA5 | 1.616 | .000 | .000 | .000 | .000 | .000 | .000 | .000 | .000 |
| CS1 | 1.017 | .000 | .000 | .000 | .000 | .000 | .000 | .000 | .000 |
| CS2 | .991 | .000 | .000 | .000 | .000 | .000 | .000 | .000 | .000 |
| CS3 | 1.028 | .000 | .000 | .000 | .000 | .000 | .000 | .000 | .000 |
| CS4 | 1.019 | .000 | .000 | .000 | .000 | .000 | .000 | .000 | .000 |
| CS5 | 1.025 | .000 | .000 | .000 | .000 | .000 | .000 | .000 | .000 |
| CS6 | 1.036 | .000 | .000 | .000 | .000 | .000 | .000 | .000 | .000 |
| CS7 | 1.000 | .000 | .000 | .000 | .000 | .000 | .000 | .000 | .000 |
| CS8 | 1.023 | .000 | .000 | .000 | .000 | .000 | .000 | .000 | .000 |
| CS9 | 1.079 | .000 | .000 | .000 | .000 | .000 | .000 | .000 | .000 |
| CS10 | 1.040 | .000 | .000 | .000 | .000 | .000 | .000 | .000 | .000 |
| CS11 | 1.054 | .000 | .000 | .000 | .000 | .000 | .000 | .000 | .000 |
| CS12 | 1.284 | .000 | .000 | .000 | .000 | .000 | .000 | .000 | .000 |
| CS13 | 1.204 | .000 | .000 | .000 | .000 | .000 | .000 | .000 | .000 |
| TS1 | .909 | .000 | .000 | .000 | .000 | .000 | .000 | .000 | .000 |
| TS2 | 1.011 | .000 | .000 | .000 | .000 | .000 | .000 | .000 | .000 |
| TS3 | 1.000 | .000 | .000 | .000 | .000 | .000 | .000 | .000 | .000 |
| TS4 | .986 | .000 | .000 | .000 | .000 | .000 | .000 | .000 | .000 |
| TS5 | 1.007 | .000 | .000 | .000 | .000 | .000 | .000 | .000 | .000 |
| TS6 | .879 | .000 | .000 | .000 | .000 | .000 | .000 | .000 | .000 |
| TS7 | 1.118 | .000 | .000 | .000 | .000 | .000 | .000 | .000 | .000 |
| EB1 | .314 | .000 | .000 | .000 | .000 | .000 | .000 | .000 | .000 |
| EB2 | .299 | .000 | .000 | .000 | .000 | .000 | .000 | .000 | .000 |
| EB3 | .268 | .000 | .000 | .000 | .000 | .000 | .000 | .000 | .000 |
| EB4 | .304 | .000 | .000 | .000 | .000 | .000 | .000 | .000 | .000 |
| EB5 | .276 | .000 | .000 | .000 | .000 | .000 | .000 | .000 | .000 |
| EX3 | .589 | .270 | .538 | .000 | .000 | .000 | .000 | .000 | .000 |
| EX2 | .624 | .286 | .570 | .000 | .000 | .000 | .000 | .000 | .000 |
| EX1 | .560 | .257 | .512 | .000 | .000 | .000 | .000 | .000 | .000 |

##### Standardized Indirect Effects (Group number 1 - Default model)

|  | Perceive\_SchoolClimate | Perseverance | Exercise\_Benefits | Enduring\_Enthusiasm | Unrelenting\_Efforts | Opportunities\_forAutonomy | Teacher\_Support | Classmate\_Support | Exercise\_Behaviour |
| --- | --- | --- | --- | --- | --- | --- | --- | --- | --- |
| Perseverance | .000 | .000 | .000 | .000 | .000 | .000 | .000 | .000 | .000 |
| Exercise\_Benefits | .000 | .000 | .000 | .000 | .000 | .000 | .000 | .000 | .000 |
| Enduring\_Enthusiasm | .148 | .000 | .000 | .000 | .000 | .000 | .000 | .000 | .000 |
| Unrelenting\_Efforts | .144 | .000 | .000 | .000 | .000 | .000 | .000 | .000 | .000 |
| Opportunities\_forAutonomy | .000 | .000 | .000 | .000 | .000 | .000 | .000 | .000 | .000 |
| Teacher\_Support | .000 | .000 | .000 | .000 | .000 | .000 | .000 | .000 | .000 |
| Classmate\_Support | .000 | .000 | .000 | .000 | .000 | .000 | .000 | .000 | .000 |
| Exercise\_Behaviour | .118 | .000 | .000 | .000 | .000 | .000 | .000 | .000 | .000 |
| EE6 | .116 | .658 | .000 | .000 | .000 | .000 | .000 | .000 | .000 |
| EE5 | .127 | .724 | .000 | .000 | .000 | .000 | .000 | .000 | .000 |
| EE4 | .126 | .719 | .000 | .000 | .000 | .000 | .000 | .000 | .000 |
| EE3 | .128 | .727 | .000 | .000 | .000 | .000 | .000 | .000 | .000 |
| EE2 | .125 | .713 | .000 | .000 | .000 | .000 | .000 | .000 | .000 |
| EE1 | .124 | .703 | .000 | .000 | .000 | .000 | .000 | .000 | .000 |
| UE6 | .107 | .608 | .000 | .000 | .000 | .000 | .000 | .000 | .000 |
| UE5 | .122 | .693 | .000 | .000 | .000 | .000 | .000 | .000 | .000 |
| UE4 | .123 | .700 | .000 | .000 | .000 | .000 | .000 | .000 | .000 |
| UE3 | .123 | .701 | .000 | .000 | .000 | .000 | .000 | .000 | .000 |
| UE2 | .118 | .671 | .000 | .000 | .000 | .000 | .000 | .000 | .000 |
| UE1 | .114 | .651 | .000 | .000 | .000 | .000 | .000 | .000 | .000 |
| OA1 | .706 | .000 | .000 | .000 | .000 | .000 | .000 | .000 | .000 |
| OA2 | .628 | .000 | .000 | .000 | .000 | .000 | .000 | .000 | .000 |
| OA3 | .653 | .000 | .000 | .000 | .000 | .000 | .000 | .000 | .000 |
| OA4 | .710 | .000 | .000 | .000 | .000 | .000 | .000 | .000 | .000 |
| OA5 | .670 | .000 | .000 | .000 | .000 | .000 | .000 | .000 | .000 |
| CS1 | .607 | .000 | .000 | .000 | .000 | .000 | .000 | .000 | .000 |
| CS2 | .595 | .000 | .000 | .000 | .000 | .000 | .000 | .000 | .000 |
| CS3 | .574 | .000 | .000 | .000 | .000 | .000 | .000 | .000 | .000 |
| CS4 | .619 | .000 | .000 | .000 | .000 | .000 | .000 | .000 | .000 |
| CS5 | .604 | .000 | .000 | .000 | .000 | .000 | .000 | .000 | .000 |
| CS6 | .612 | .000 | .000 | .000 | .000 | .000 | .000 | .000 | .000 |
| CS7 | .611 | .000 | .000 | .000 | .000 | .000 | .000 | .000 | .000 |
| CS8 | .607 | .000 | .000 | .000 | .000 | .000 | .000 | .000 | .000 |
| CS9 | .616 | .000 | .000 | .000 | .000 | .000 | .000 | .000 | .000 |
| CS10 | .611 | .000 | .000 | .000 | .000 | .000 | .000 | .000 | .000 |
| CS11 | .614 | .000 | .000 | .000 | .000 | .000 | .000 | .000 | .000 |
| CS12 | .578 | .000 | .000 | .000 | .000 | .000 | .000 | .000 | .000 |
| CS13 | .559 | .000 | .000 | .000 | .000 | .000 | .000 | .000 | .000 |
| TS1 | .495 | .000 | .000 | .000 | .000 | .000 | .000 | .000 | .000 |
| TS2 | .516 | .000 | .000 | .000 | .000 | .000 | .000 | .000 | .000 |
| TS3 | .520 | .000 | .000 | .000 | .000 | .000 | .000 | .000 | .000 |
| TS4 | .503 | .000 | .000 | .000 | .000 | .000 | .000 | .000 | .000 |
| TS5 | .506 | .000 | .000 | .000 | .000 | .000 | .000 | .000 | .000 |
| TS6 | .475 | .000 | .000 | .000 | .000 | .000 | .000 | .000 | .000 |
| TS7 | .468 | .000 | .000 | .000 | .000 | .000 | .000 | .000 | .000 |
| EB1 | .160 | .000 | .000 | .000 | .000 | .000 | .000 | .000 | .000 |
| EB2 | .159 | .000 | .000 | .000 | .000 | .000 | .000 | .000 | .000 |
| EB3 | .141 | .000 | .000 | .000 | .000 | .000 | .000 | .000 | .000 |
| EB4 | .158 | .000 | .000 | .000 | .000 | .000 | .000 | .000 | .000 |
| EB5 | .133 | .000 | .000 | .000 | .000 | .000 | .000 | .000 | .000 |
| EX3 | .185 | .168 | .176 | .000 | .000 | .000 | .000 | .000 | .000 |
| EX2 | .209 | .190 | .199 | .000 | .000 | .000 | .000 | .000 | .000 |
| EX1 | .186 | .169 | .177 | .000 | .000 | .000 | .000 | .000 | .000 |

##### Bootstrap (Group number 1 - Default model)

##### Bootstrap standard errors (Group number 1 - Default model)

##### Scalar Estimates (Group number 1 - Default model)

##### Regression Weights: (Group number 1 - Default model)

| Parameter | | | SE | SE-SE | Mean | Bias | SE-Bias |
| --- | --- | --- | --- | --- | --- | --- | --- |
| Perseverance | <--- | Perceive\_SchoolClimate | .124 | .001 | .349 | .001 | .002 |
| Exercise\_Benefits | <--- | Perceive\_SchoolClimate | .088 | .001 | .280 | .004 | .001 |
| Exercise\_Behaviour | <--- | Perceive\_SchoolClimate | .133 | .001 | .338 | .008 | .002 |
| Classmate\_Support | <--- | Perceive\_SchoolClimate | .000 | .000 | 1.000 | .000 | .000 |
| Teacher\_Support | <--- | Perceive\_SchoolClimate | .168 | .002 | 1.126 | .008 | .002 |
| Opportunities\_forAutonomy | <--- | Perceive\_SchoolClimate | .158 | .002 | 1.246 | .005 | .002 |
| Unrelenting\_Efforts | <--- | Perseverance | .000 | .000 | 1.000 | .000 | .000 |
| Exercise\_Behaviour | <--- | Exercise\_Benefits | .144 | .001 | .516 | .005 | .002 |
| Exercise\_Behaviour | <--- | Perseverance | .063 | .001 | .260 | .003 | .001 |
| Enduring\_Enthusiasm | <--- | Perseverance | .219 | .002 | 1.201 | .022 | .003 |
| EX1 | <--- | Exercise\_Behaviour | .000 | .000 | 1.000 | .000 | .000 |
| EX2 | <--- | Exercise\_Behaviour | .098 | .001 | 1.116 | .002 | .001 |
| EX3 | <--- | Exercise\_Behaviour | .095 | .001 | 1.054 | .002 | .001 |
| EB5 | <--- | Exercise\_Benefits | .000 | .000 | 1.000 | .000 | .000 |
| EB4 | <--- | Exercise\_Benefits | .123 | .001 | 1.111 | .008 | .002 |
| EB3 | <--- | Exercise\_Benefits | .120 | .001 | .980 | .008 | .002 |
| EB2 | <--- | Exercise\_Benefits | .135 | .001 | 1.092 | .008 | .002 |
| EB1 | <--- | Exercise\_Benefits | .140 | .001 | 1.146 | .008 | .002 |
| TS7 | <--- | Teacher\_Support | .000 | .000 | 1.000 | .000 | .000 |
| TS6 | <--- | Teacher\_Support | .040 | .000 | .784 | -.002 | .001 |
| TS5 | <--- | Teacher\_Support | .031 | .000 | .900 | -.001 | .000 |
| TS4 | <--- | Teacher\_Support | .032 | .000 | .881 | -.001 | .000 |
| TS3 | <--- | Teacher\_Support | .029 | .000 | .894 | -.001 | .000 |
| TS2 | <--- | Teacher\_Support | .030 | .000 | .903 | -.001 | .000 |
| TS1 | <--- | Teacher\_Support | .036 | .000 | .812 | -.002 | .001 |
| CS11 | <--- | Classmate\_Support | .046 | .000 | 1.055 | .001 | .001 |
| CS10 | <--- | Classmate\_Support | .033 | .000 | 1.042 | .001 | .000 |
| CS9 | <--- | Classmate\_Support | .033 | .000 | 1.080 | .001 | .000 |
| CS8 | <--- | Classmate\_Support | .043 | .000 | 1.023 | .000 | .001 |
| CS7 | <--- | Classmate\_Support | .000 | .000 | 1.000 | .000 | .000 |
| CS6 | <--- | Classmate\_Support | .038 | .000 | 1.037 | .001 | .001 |
| CS5 | <--- | Classmate\_Support | .041 | .000 | 1.026 | .001 | .001 |
| CS4 | <--- | Classmate\_Support | .038 | .000 | 1.020 | .001 | .001 |
| CS3 | <--- | Classmate\_Support | .045 | .000 | 1.029 | .001 | .001 |
| OA5 | <--- | Opportunities\_forAutonomy | .077 | .001 | 1.309 | .007 | .001 |
| OA4 | <--- | Opportunities\_forAutonomy | .055 | .001 | .976 | .002 | .001 |
| OA3 | <--- | Opportunities\_forAutonomy | .000 | .000 | 1.000 | .000 | .000 |
| OA2 | <--- | Opportunities\_forAutonomy | .047 | .000 | .954 | .001 | .001 |
| OA1 | <--- | Opportunities\_forAutonomy | .054 | .001 | 1.011 | .002 | .001 |
| UE1 | <--- | Unrelenting\_Efforts | .000 | .000 | 1.000 | .000 | .000 |
| UE2 | <--- | Unrelenting\_Efforts | .037 | .000 | 1.003 | .000 | .001 |
| UE3 | <--- | Unrelenting\_Efforts | .041 | .000 | 1.124 | .000 | .001 |
| UE4 | <--- | Unrelenting\_Efforts | .041 | .000 | 1.125 | .001 | .001 |
| UE5 | <--- | Unrelenting\_Efforts | .038 | .000 | 1.062 | .000 | .001 |
| UE6 | <--- | Unrelenting\_Efforts | .051 | .001 | 1.088 | .001 | .001 |
| EE1 | <--- | Enduring\_Enthusiasm | .000 | .000 | 1.000 | .000 | .000 |
| EE2 | <--- | Enduring\_Enthusiasm | .030 | .000 | .978 | -.001 | .000 |
| EE3 | <--- | Enduring\_Enthusiasm | .032 | .000 | 1.036 | -.001 | .000 |
| EE4 | <--- | Enduring\_Enthusiasm | .033 | .000 | .995 | .000 | .000 |
| EE5 | <--- | Enduring\_Enthusiasm | .029 | .000 | 1.020 | .000 | .000 |
| EE6 | <--- | Enduring\_Enthusiasm | .036 | .000 | .961 | .000 | .001 |
| CS1 | <--- | Classmate\_Support | .042 | .000 | 1.018 | .000 | .001 |
| CS13 | <--- | Classmate\_Support | .059 | .001 | 1.207 | .004 | .001 |
| CS12 | <--- | Classmate\_Support | .064 | .001 | 1.290 | .006 | .001 |
| CS2 | <--- | Classmate\_Support | .039 | .000 | .992 | .001 | .001 |

##### Standardized Regression Weights: (Group number 1 - Default model)

| Parameter | | | SE | SE-SE | Mean | Bias | SE-Bias |
| --- | --- | --- | --- | --- | --- | --- | --- |
| Perseverance | <--- | Perceive\_SchoolClimate | .053 | .001 | .173 | -.003 | .001 |
| Exercise\_Benefits | <--- | Perceive\_SchoolClimate | .069 | .001 | .265 | .000 | .001 |
| Exercise\_Behaviour | <--- | Perceive\_SchoolClimate | .061 | .001 | .171 | .002 | .001 |
| Classmate\_Support | <--- | Perceive\_SchoolClimate | .052 | .001 | .672 | .001 | .001 |
| Teacher\_Support | <--- | Perceive\_SchoolClimate | .063 | .001 | .549 | -.001 | .001 |
| Opportunities\_forAutonomy | <--- | Perceive\_SchoolClimate | .057 | .001 | .842 | -.002 | .001 |
| Unrelenting\_Efforts | <--- | Perseverance | .073 | .001 | .822 | .001 | .001 |
| Exercise\_Behaviour | <--- | Exercise\_Benefits | .065 | .001 | .272 | -.001 | .001 |
| Exercise\_Behaviour | <--- | Perseverance | .058 | .001 | .263 | .002 | .001 |
| Enduring\_Enthusiasm | <--- | Perseverance | .075 | .001 | .846 | .004 | .001 |
| EX1 | <--- | Exercise\_Behaviour | .039 | .000 | .645 | .000 | .001 |
| EX2 | <--- | Exercise\_Behaviour | .041 | .000 | .726 | -.001 | .001 |
| EX3 | <--- | Exercise\_Behaviour | .039 | .000 | .642 | -.001 | .001 |
| EB5 | <--- | Exercise\_Benefits | .047 | .000 | .503 | -.001 | .001 |
| EB4 | <--- | Exercise\_Benefits | .047 | .000 | .597 | -.001 | .001 |
| EB3 | <--- | Exercise\_Benefits | .052 | .001 | .531 | .000 | .001 |
| EB2 | <--- | Exercise\_Benefits | .049 | .000 | .597 | -.002 | .001 |
| EB1 | <--- | Exercise\_Benefits | .048 | .000 | .603 | -.002 | .001 |
| TS7 | <--- | Teacher\_Support | .019 | .000 | .851 | .000 | .000 |
| TS6 | <--- | Teacher\_Support | .021 | .000 | .861 | -.002 | .000 |
| TS5 | <--- | Teacher\_Support | .015 | .000 | .920 | .000 | .000 |
| TS4 | <--- | Teacher\_Support | .016 | .000 | .914 | .000 | .000 |
| TS3 | <--- | Teacher\_Support | .012 | .000 | .945 | .000 | .000 |
| TS2 | <--- | Teacher\_Support | .012 | .000 | .937 | .000 | .000 |
| TS1 | <--- | Teacher\_Support | .017 | .000 | .899 | -.001 | .000 |
| CS11 | <--- | Classmate\_Support | .015 | .000 | .915 | .000 | .000 |
| CS10 | <--- | Classmate\_Support | .015 | .000 | .910 | .000 | .000 |
| CS9 | <--- | Classmate\_Support | .014 | .000 | .917 | -.001 | .000 |
| CS8 | <--- | Classmate\_Support | .016 | .000 | .903 | -.001 | .000 |
| CS7 | <--- | Classmate\_Support | .016 | .000 | .910 | -.001 | .000 |
| CS6 | <--- | Classmate\_Support | .015 | .000 | .912 | -.001 | .000 |
| CS5 | <--- | Classmate\_Support | .017 | .000 | .900 | .000 | .000 |
| CS4 | <--- | Classmate\_Support | .015 | .000 | .922 | .000 | .000 |
| CS3 | <--- | Classmate\_Support | .025 | .000 | .855 | -.001 | .000 |
| OA5 | <--- | Opportunities\_forAutonomy | .021 | .000 | .793 | .000 | .000 |
| OA4 | <--- | Opportunities\_forAutonomy | .029 | .000 | .840 | -.001 | .000 |
| OA3 | <--- | Opportunities\_forAutonomy | .031 | .000 | .771 | -.002 | .000 |
| OA2 | <--- | Opportunities\_forAutonomy | .033 | .000 | .742 | -.002 | .000 |
| OA1 | <--- | Opportunities\_forAutonomy | .026 | .000 | .835 | -.001 | .000 |
| UE1 | <--- | Unrelenting\_Efforts | .019 | .000 | .793 | .000 | .000 |
| UE2 | <--- | Unrelenting\_Efforts | .017 | .000 | .818 | .000 | .000 |
| UE3 | <--- | Unrelenting\_Efforts | .015 | .000 | .855 | .000 | .000 |
| UE4 | <--- | Unrelenting\_Efforts | .015 | .000 | .854 | .000 | .000 |
| UE5 | <--- | Unrelenting\_Efforts | .015 | .000 | .845 | .000 | .000 |
| UE6 | <--- | Unrelenting\_Efforts | .031 | .000 | .741 | .000 | .000 |
| EE1 | <--- | Enduring\_Enthusiasm | .015 | .000 | .835 | .000 | .000 |
| EE2 | <--- | Enduring\_Enthusiasm | .015 | .000 | .846 | .000 | .000 |
| EE3 | <--- | Enduring\_Enthusiasm | .014 | .000 | .863 | .000 | .000 |
| EE4 | <--- | Enduring\_Enthusiasm | .015 | .000 | .854 | .000 | .000 |
| EE5 | <--- | Enduring\_Enthusiasm | .014 | .000 | .859 | .000 | .000 |
| EE6 | <--- | Enduring\_Enthusiasm | .022 | .000 | .781 | .000 | .000 |
| CS1 | <--- | Classmate\_Support | .016 | .000 | .904 | -.001 | .000 |
| CS13 | <--- | Classmate\_Support | .019 | .000 | .834 | .000 | .000 |
| CS12 | <--- | Classmate\_Support | .013 | .000 | .861 | .000 | .000 |
| CS2 | <--- | Classmate\_Support | .018 | .000 | .887 | -.001 | .000 |

##### Variances: (Group number 1 - Default model)

| Parameter | | | SE | SE-SE | Mean | Bias | SE-Bias |
| --- | --- | --- | --- | --- | --- | --- | --- |
| Perceive\_SchoolClimate |  |  | .029 | .000 | .140 | .001 | .000 |
| e3 |  |  | .030 | .000 | .140 | .000 | .000 |
| e1 |  |  | .109 | .001 | .534 | .006 | .002 |
| e6 |  |  | .051 | .001 | .397 | -.002 | .001 |
| e7 |  |  | .028 | .000 | .168 | -.002 | .000 |
| e8 |  |  | .031 | .000 | .086 | .000 | .000 |
| e4 |  |  | .097 | .001 | .258 | -.007 | .001 |
| e5 |  |  | .138 | .001 | .296 | -.013 | .002 |
| e2 |  |  | .060 | .001 | .407 | -.005 | .001 |
| e52 |  |  | .062 | .001 | .734 | -.003 | .001 |
| e53 |  |  | .072 | .001 | .579 | -.002 | .001 |
| e54 |  |  | .069 | .001 | .825 | -.003 | .001 |
| e51 |  |  | .029 | .000 | .441 | -.002 | .000 |
| e50 |  |  | .027 | .000 | .327 | -.002 | .000 |
| e49 |  |  | .026 | .000 | .359 | -.003 | .000 |
| e48 |  |  | .027 | .000 | .315 | -.001 | .000 |
| e47 |  |  | .029 | .000 | .337 | -.001 | .000 |
| e15 |  |  | .032 | .000 | .219 | -.001 | .000 |
| e14 |  |  | .015 | .000 | .121 | .000 | .000 |
| e13 |  |  | .015 | .000 | .084 | -.001 | .000 |
| e12 |  |  | .016 | .000 | .087 | .000 | .000 |
| e11 |  |  | .012 | .000 | .054 | .000 | .000 |
| e10 |  |  | .012 | .000 | .064 | -.001 | .000 |
| e9 |  |  | .013 | .000 | .088 | -.001 | .000 |
| e29 |  |  | .025 | .000 | .195 | -.001 | .000 |
| e28 |  |  | .017 | .000 | .176 | -.001 | .000 |
| e27 |  |  | .011 | .000 | .066 | .000 | .000 |
| e26 |  |  | .010 | .000 | .068 | .000 | .000 |
| e25 |  |  | .011 | .000 | .067 | .000 | .000 |
| e24 |  |  | .011 | .000 | .072 | .000 | .000 |
| e23 |  |  | .010 | .000 | .063 | .000 | .000 |
| e22 |  |  | .010 | .000 | .066 | .000 | .000 |
| e21 |  |  | .011 | .000 | .075 | -.001 | .000 |
| e20 |  |  | .009 | .000 | .056 | .000 | .000 |
| e19 |  |  | .020 | .000 | .119 | .000 | .000 |
| e18 |  |  | .011 | .000 | .081 | -.001 | .000 |
| e16 |  |  | .010 | .000 | .070 | .000 | .000 |
| e34 |  |  | .037 | .000 | .299 | -.001 | .001 |
| e33 |  |  | .017 | .000 | .117 | -.001 | .000 |
| e32 |  |  | .022 | .000 | .202 | .000 | .000 |
| e31 |  |  | .020 | .000 | .219 | -.001 | .000 |
| e30 |  |  | .016 | .000 | .130 | -.001 | .000 |
| e35 |  |  | .038 | .000 | .474 | -.002 | .001 |
| e36 |  |  | .032 | .000 | .402 | -.001 | .000 |
| e37 |  |  | .036 | .000 | .375 | -.001 | .001 |
| e38 |  |  | .035 | .000 | .379 | -.001 | .001 |
| e39 |  |  | .030 | .000 | .363 | -.001 | .000 |
| e40 |  |  | .091 | .001 | .783 | -.003 | .001 |
| e41 |  |  | .036 | .000 | .460 | -.002 | .001 |
| e42 |  |  | .034 | .000 | .402 | -.001 | .000 |
| e43 |  |  | .036 | .000 | .391 | -.001 | .001 |
| e44 |  |  | .036 | .000 | .391 | -.001 | .001 |
| e45 |  |  | .036 | .000 | .391 | -.001 | .001 |
| e46 |  |  | .055 | .001 | .625 | -.002 | .001 |

##### Squared Multiple Correlations: (Group number 1 - Default model)

| Parameter | | | SE | SE-SE | Mean | Bias | SE-Bias |
| --- | --- | --- | --- | --- | --- | --- | --- |
| Perseverance |  |  | .018 | .000 | .033 | .002 | .000 |
| Exercise\_Benefits |  |  | .038 | .000 | .075 | .005 | .001 |
| Enduring\_Enthusiasm |  |  | .129 | .001 | .722 | .012 | .002 |
| Unrelenting\_Efforts |  |  | .120 | .001 | .681 | .008 | .002 |
| Opportunities\_forAutonomy |  |  | .096 | .001 | .713 | -.001 | .001 |
| Teacher\_Support |  |  | .069 | .001 | .306 | .003 | .001 |
| Classmate\_Support |  |  | .070 | .001 | .454 | .004 | .001 |
| Exercise\_Behaviour |  |  | .047 | .000 | .230 | .011 | .001 |
| EE6 |  |  | .034 | .000 | .611 | .000 | .000 |
| EE5 |  |  | .024 | .000 | .739 | .000 | .000 |
| EE4 |  |  | .026 | .000 | .729 | .000 | .000 |
| EE3 |  |  | .025 | .000 | .745 | .000 | .000 |
| EE2 |  |  | .026 | .000 | .716 | .000 | .000 |
| EE1 |  |  | .025 | .000 | .698 | .001 | .000 |
| UE6 |  |  | .045 | .000 | .550 | .001 | .001 |
| UE5 |  |  | .025 | .000 | .714 | .000 | .000 |
| UE4 |  |  | .026 | .000 | .729 | .000 | .000 |
| UE3 |  |  | .026 | .000 | .731 | .000 | .000 |
| UE2 |  |  | .028 | .000 | .669 | .000 | .000 |
| UE1 |  |  | .031 | .000 | .630 | .001 | .000 |
| OA1 |  |  | .043 | .000 | .698 | -.001 | .001 |
| OA2 |  |  | .049 | .000 | .552 | -.002 | .001 |
| OA3 |  |  | .047 | .000 | .595 | -.002 | .001 |
| OA4 |  |  | .048 | .000 | .707 | .000 | .001 |
| OA5 |  |  | .033 | .000 | .630 | .001 | .000 |
| CS1 |  |  | .029 | .000 | .818 | -.001 | .000 |
| CS2 |  |  | .032 | .000 | .787 | -.001 | .000 |
| CS3 |  |  | .043 | .000 | .732 | -.001 | .001 |
| CS4 |  |  | .027 | .000 | .851 | -.001 | .000 |
| CS5 |  |  | .030 | .000 | .810 | .000 | .000 |
| CS6 |  |  | .027 | .000 | .832 | -.001 | .000 |
| CS7 |  |  | .029 | .000 | .828 | -.001 | .000 |
| CS8 |  |  | .029 | .000 | .817 | -.002 | .000 |
| CS9 |  |  | .026 | .000 | .842 | -.001 | .000 |
| CS10 |  |  | .027 | .000 | .829 | -.001 | .000 |
| CS11 |  |  | .027 | .000 | .837 | -.001 | .000 |
| CS12 |  |  | .023 | .000 | .742 | .001 | .000 |
| CS13 |  |  | .032 | .000 | .696 | .000 | .000 |
| TS1 |  |  | .031 | .000 | .809 | -.001 | .000 |
| TS2 |  |  | .023 | .000 | .878 | .000 | .000 |
| TS3 |  |  | .023 | .000 | .894 | .000 | .000 |
| TS4 |  |  | .029 | .000 | .836 | -.001 | .000 |
| TS5 |  |  | .027 | .000 | .846 | .000 | .000 |
| TS6 |  |  | .036 | .000 | .742 | -.002 | .001 |
| TS7 |  |  | .032 | .000 | .724 | .001 | .000 |
| EB1 |  |  | .057 | .001 | .366 | .000 | .001 |
| EB2 |  |  | .058 | .001 | .359 | .000 | .001 |
| EB3 |  |  | .055 | .001 | .285 | .003 | .001 |
| EB4 |  |  | .056 | .001 | .359 | .001 | .001 |
| EB5 |  |  | .047 | .000 | .255 | .002 | .001 |
| EX3 |  |  | .050 | .000 | .413 | .000 | .001 |
| EX2 |  |  | .059 | .001 | .529 | .000 | .001 |
| EX1 |  |  | .050 | .000 | .418 | .001 | .001 |

##### User-defined estimands: (Group number 1 - Default model)

| Parameter | | | SE | SE-SE | Mean | Bias | SE-Bias |
| --- | --- | --- | --- | --- | --- | --- | --- |
| Estimand 1 |  |  | .039 | .000 | .090 | .001 | .001 |
| Estimand 2 |  |  | .056 | .001 | .143 | .002 | .001 |
| Estimand 3 |  |  | .133 | .001 | .338 | .008 | .002 |
| Estimand 4 |  |  | .065 | .001 | -.052 | -.001 | .001 |
| Estimand 5 |  |  | .150 | .002 | .571 | .011 | .002 |

##### Matrices (Group number 1 - Default model)

##### Total Effects - Standard Errors (Group number 1 - Default model)

|  | Perceive\_SchoolClimate | Perseverance | Exercise\_Benefits | Enduring\_Enthusiasm | Unrelenting\_Efforts | Opportunities\_forAutonomy | Teacher\_Support | Classmate\_Support | Exercise\_Behaviour |
| --- | --- | --- | --- | --- | --- | --- | --- | --- | --- |
| Perseverance | .124 | .000 | .000 | .000 | .000 | .000 | .000 | .000 | .000 |
| Exercise\_Benefits | .088 | .000 | .000 | .000 | .000 | .000 | .000 | .000 | .000 |
| Enduring\_Enthusiasm | .147 | .219 | .000 | .000 | .000 | .000 | .000 | .000 | .000 |
| Unrelenting\_Efforts | .124 | .000 | .000 | .000 | .000 | .000 | .000 | .000 | .000 |
| Opportunities\_forAutonomy | .158 | .000 | .000 | .000 | .000 | .000 | .000 | .000 | .000 |
| Teacher\_Support | .168 | .000 | .000 | .000 | .000 | .000 | .000 | .000 | .000 |
| Classmate\_Support | .000 | .000 | .000 | .000 | .000 | .000 | .000 | .000 | .000 |
| Exercise\_Behaviour | .150 | .063 | .144 | .000 | .000 | .000 | .000 | .000 | .000 |
| EE6 | .143 | .213 | .000 | .036 | .000 | .000 | .000 | .000 | .000 |
| EE5 | .151 | .225 | .000 | .029 | .000 | .000 | .000 | .000 | .000 |
| EE4 | .147 | .219 | .000 | .033 | .000 | .000 | .000 | .000 | .000 |
| EE3 | .152 | .227 | .000 | .032 | .000 | .000 | .000 | .000 | .000 |
| EE2 | .145 | .212 | .000 | .030 | .000 | .000 | .000 | .000 | .000 |
| EE1 | .147 | .219 | .000 | .000 | .000 | .000 | .000 | .000 | .000 |
| UE6 | .135 | .051 | .000 | .000 | .051 | .000 | .000 | .000 | .000 |
| UE5 | .132 | .038 | .000 | .000 | .038 | .000 | .000 | .000 | .000 |
| UE4 | .139 | .041 | .000 | .000 | .041 | .000 | .000 | .000 | .000 |
| UE3 | .139 | .041 | .000 | .000 | .041 | .000 | .000 | .000 | .000 |
| UE2 | .124 | .037 | .000 | .000 | .037 | .000 | .000 | .000 | .000 |
| UE1 | .124 | .000 | .000 | .000 | .000 | .000 | .000 | .000 | .000 |
| OA1 | .168 | .000 | .000 | .000 | .000 | .054 | .000 | .000 | .000 |
| OA2 | .157 | .000 | .000 | .000 | .000 | .047 | .000 | .000 | .000 |
| OA3 | .158 | .000 | .000 | .000 | .000 | .000 | .000 | .000 | .000 |
| OA4 | .158 | .000 | .000 | .000 | .000 | .055 | .000 | .000 | .000 |
| OA5 | .202 | .000 | .000 | .000 | .000 | .077 | .000 | .000 | .000 |
| CS1 | .042 | .000 | .000 | .000 | .000 | .000 | .000 | .042 | .000 |
| CS2 | .039 | .000 | .000 | .000 | .000 | .000 | .000 | .039 | .000 |
| CS3 | .045 | .000 | .000 | .000 | .000 | .000 | .000 | .045 | .000 |
| CS4 | .038 | .000 | .000 | .000 | .000 | .000 | .000 | .038 | .000 |
| CS5 | .041 | .000 | .000 | .000 | .000 | .000 | .000 | .041 | .000 |
| CS6 | .038 | .000 | .000 | .000 | .000 | .000 | .000 | .038 | .000 |
| CS7 | .000 | .000 | .000 | .000 | .000 | .000 | .000 | .000 | .000 |
| CS8 | .043 | .000 | .000 | .000 | .000 | .000 | .000 | .043 | .000 |
| CS9 | .033 | .000 | .000 | .000 | .000 | .000 | .000 | .033 | .000 |
| CS10 | .033 | .000 | .000 | .000 | .000 | .000 | .000 | .033 | .000 |
| CS11 | .046 | .000 | .000 | .000 | .000 | .000 | .000 | .046 | .000 |
| CS12 | .064 | .000 | .000 | .000 | .000 | .000 | .000 | .064 | .000 |
| CS13 | .059 | .000 | .000 | .000 | .000 | .000 | .000 | .059 | .000 |
| TS1 | .142 | .000 | .000 | .000 | .000 | .000 | .036 | .000 | .000 |
| TS2 | .154 | .000 | .000 | .000 | .000 | .000 | .030 | .000 | .000 |
| TS3 | .152 | .000 | .000 | .000 | .000 | .000 | .029 | .000 | .000 |
| TS4 | .150 | .000 | .000 | .000 | .000 | .000 | .032 | .000 | .000 |
| TS5 | .155 | .000 | .000 | .000 | .000 | .000 | .031 | .000 | .000 |
| TS6 | .138 | .000 | .000 | .000 | .000 | .000 | .040 | .000 | .000 |
| TS7 | .168 | .000 | .000 | .000 | .000 | .000 | .000 | .000 | .000 |
| EB1 | .097 | .000 | .140 | .000 | .000 | .000 | .000 | .000 | .000 |
| EB2 | .090 | .000 | .135 | .000 | .000 | .000 | .000 | .000 | .000 |
| EB3 | .087 | .000 | .120 | .000 | .000 | .000 | .000 | .000 | .000 |
| EB4 | .093 | .000 | .123 | .000 | .000 | .000 | .000 | .000 | .000 |
| EB5 | .088 | .000 | .000 | .000 | .000 | .000 | .000 | .000 | .000 |
| EX3 | .151 | .066 | .154 | .000 | .000 | .000 | .000 | .000 | .095 |
| EX2 | .156 | .072 | .154 | .000 | .000 | .000 | .000 | .000 | .098 |
| EX1 | .150 | .063 | .144 | .000 | .000 | .000 | .000 | .000 | .000 |

##### Standardized Total Effects - Standard Errors (Group number 1 - Default model)

|  | Perceive\_SchoolClimate | Perseverance | Exercise\_Benefits | Enduring\_Enthusiasm | Unrelenting\_Efforts | Opportunities\_forAutonomy | Teacher\_Support | Classmate\_Support | Exercise\_Behaviour |
| --- | --- | --- | --- | --- | --- | --- | --- | --- | --- |
| Perseverance | .053 | .000 | .000 | .000 | .000 | .000 | .000 | .000 | .000 |
| Exercise\_Benefits | .069 | .000 | .000 | .000 | .000 | .000 | .000 | .000 | .000 |
| Enduring\_Enthusiasm | .047 | .075 | .000 | .000 | .000 | .000 | .000 | .000 | .000 |
| Unrelenting\_Efforts | .045 | .073 | .000 | .000 | .000 | .000 | .000 | .000 | .000 |
| Opportunities\_forAutonomy | .057 | .000 | .000 | .000 | .000 | .000 | .000 | .000 | .000 |
| Teacher\_Support | .063 | .000 | .000 | .000 | .000 | .000 | .000 | .000 | .000 |
| Classmate\_Support | .052 | .000 | .000 | .000 | .000 | .000 | .000 | .000 | .000 |
| Exercise\_Behaviour | .061 | .058 | .065 | .000 | .000 | .000 | .000 | .000 | .000 |
| EE6 | .037 | .062 | .000 | .022 | .000 | .000 | .000 | .000 | .000 |
| EE5 | .040 | .066 | .000 | .014 | .000 | .000 | .000 | .000 | .000 |
| EE4 | .040 | .065 | .000 | .015 | .000 | .000 | .000 | .000 | .000 |
| EE3 | .040 | .066 | .000 | .014 | .000 | .000 | .000 | .000 | .000 |
| EE2 | .040 | .064 | .000 | .015 | .000 | .000 | .000 | .000 | .000 |
| EE1 | .039 | .065 | .000 | .015 | .000 | .000 | .000 | .000 | .000 |
| UE6 | .034 | .059 | .000 | .000 | .031 | .000 | .000 | .000 | .000 |
| UE5 | .038 | .064 | .000 | .000 | .015 | .000 | .000 | .000 | .000 |
| UE4 | .039 | .064 | .000 | .000 | .015 | .000 | .000 | .000 | .000 |
| UE3 | .039 | .064 | .000 | .000 | .015 | .000 | .000 | .000 | .000 |
| UE2 | .037 | .062 | .000 | .000 | .017 | .000 | .000 | .000 | .000 |
| UE1 | .036 | .060 | .000 | .000 | .019 | .000 | .000 | .000 | .000 |
| OA1 | .052 | .000 | .000 | .000 | .000 | .026 | .000 | .000 | .000 |
| OA2 | .052 | .000 | .000 | .000 | .000 | .033 | .000 | .000 | .000 |
| OA3 | .053 | .000 | .000 | .000 | .000 | .031 | .000 | .000 | .000 |
| OA4 | .052 | .000 | .000 | .000 | .000 | .029 | .000 | .000 | .000 |
| OA5 | .047 | .000 | .000 | .000 | .000 | .021 | .000 | .000 | .000 |
| CS1 | .049 | .000 | .000 | .000 | .000 | .000 | .000 | .016 | .000 |
| CS2 | .048 | .000 | .000 | .000 | .000 | .000 | .000 | .018 | .000 |
| CS3 | .049 | .000 | .000 | .000 | .000 | .000 | .000 | .025 | .000 |
| CS4 | .050 | .000 | .000 | .000 | .000 | .000 | .000 | .015 | .000 |
| CS5 | .049 | .000 | .000 | .000 | .000 | .000 | .000 | .017 | .000 |
| CS6 | .049 | .000 | .000 | .000 | .000 | .000 | .000 | .015 | .000 |
| CS7 | .049 | .000 | .000 | .000 | .000 | .000 | .000 | .016 | .000 |
| CS8 | .048 | .000 | .000 | .000 | .000 | .000 | .000 | .016 | .000 |
| CS9 | .049 | .000 | .000 | .000 | .000 | .000 | .000 | .014 | .000 |
| CS10 | .049 | .000 | .000 | .000 | .000 | .000 | .000 | .015 | .000 |
| CS11 | .049 | .000 | .000 | .000 | .000 | .000 | .000 | .015 | .000 |
| CS12 | .047 | .000 | .000 | .000 | .000 | .000 | .000 | .013 | .000 |
| CS13 | .047 | .000 | .000 | .000 | .000 | .000 | .000 | .019 | .000 |
| TS1 | .057 | .000 | .000 | .000 | .000 | .000 | .017 | .000 | .000 |
| TS2 | .060 | .000 | .000 | .000 | .000 | .000 | .012 | .000 | .000 |
| TS3 | .060 | .000 | .000 | .000 | .000 | .000 | .012 | .000 | .000 |
| TS4 | .059 | .000 | .000 | .000 | .000 | .000 | .016 | .000 | .000 |
| TS5 | .059 | .000 | .000 | .000 | .000 | .000 | .015 | .000 | .000 |
| TS6 | .055 | .000 | .000 | .000 | .000 | .000 | .021 | .000 | .000 |
| TS7 | .055 | .000 | .000 | .000 | .000 | .000 | .019 | .000 | .000 |
| EB1 | .044 | .000 | .048 | .000 | .000 | .000 | .000 | .000 | .000 |
| EB2 | .041 | .000 | .049 | .000 | .000 | .000 | .000 | .000 | .000 |
| EB3 | .040 | .000 | .052 | .000 | .000 | .000 | .000 | .000 | .000 |
| EB4 | .042 | .000 | .047 | .000 | .000 | .000 | .000 | .000 | .000 |
| EB5 | .037 | .000 | .047 | .000 | .000 | .000 | .000 | .000 | .000 |
| EX3 | .040 | .039 | .043 | .000 | .000 | .000 | .000 | .000 | .039 |
| EX2 | .044 | .045 | .046 | .000 | .000 | .000 | .000 | .000 | .041 |
| EX1 | .042 | .040 | .043 | .000 | .000 | .000 | .000 | .000 | .039 |

##### Direct Effects - Standard Errors (Group number 1 - Default model)

|  | Perceive\_SchoolClimate | Perseverance | Exercise\_Benefits | Enduring\_Enthusiasm | Unrelenting\_Efforts | Opportunities\_forAutonomy | Teacher\_Support | Classmate\_Support | Exercise\_Behaviour |
| --- | --- | --- | --- | --- | --- | --- | --- | --- | --- |
| Perseverance | .124 | .000 | .000 | .000 | .000 | .000 | .000 | .000 | .000 |
| Exercise\_Benefits | .088 | .000 | .000 | .000 | .000 | .000 | .000 | .000 | .000 |
| Enduring\_Enthusiasm | .000 | .219 | .000 | .000 | .000 | .000 | .000 | .000 | .000 |
| Unrelenting\_Efforts | .000 | .000 | .000 | .000 | .000 | .000 | .000 | .000 | .000 |
| Opportunities\_forAutonomy | .158 | .000 | .000 | .000 | .000 | .000 | .000 | .000 | .000 |
| Teacher\_Support | .168 | .000 | .000 | .000 | .000 | .000 | .000 | .000 | .000 |
| Classmate\_Support | .000 | .000 | .000 | .000 | .000 | .000 | .000 | .000 | .000 |
| Exercise\_Behaviour | .133 | .063 | .144 | .000 | .000 | .000 | .000 | .000 | .000 |
| EE6 | .000 | .000 | .000 | .036 | .000 | .000 | .000 | .000 | .000 |
| EE5 | .000 | .000 | .000 | .029 | .000 | .000 | .000 | .000 | .000 |
| EE4 | .000 | .000 | .000 | .033 | .000 | .000 | .000 | .000 | .000 |
| EE3 | .000 | .000 | .000 | .032 | .000 | .000 | .000 | .000 | .000 |
| EE2 | .000 | .000 | .000 | .030 | .000 | .000 | .000 | .000 | .000 |
| EE1 | .000 | .000 | .000 | .000 | .000 | .000 | .000 | .000 | .000 |
| UE6 | .000 | .000 | .000 | .000 | .051 | .000 | .000 | .000 | .000 |
| UE5 | .000 | .000 | .000 | .000 | .038 | .000 | .000 | .000 | .000 |
| UE4 | .000 | .000 | .000 | .000 | .041 | .000 | .000 | .000 | .000 |
| UE3 | .000 | .000 | .000 | .000 | .041 | .000 | .000 | .000 | .000 |
| UE2 | .000 | .000 | .000 | .000 | .037 | .000 | .000 | .000 | .000 |
| UE1 | .000 | .000 | .000 | .000 | .000 | .000 | .000 | .000 | .000 |
| OA1 | .000 | .000 | .000 | .000 | .000 | .054 | .000 | .000 | .000 |
| OA2 | .000 | .000 | .000 | .000 | .000 | .047 | .000 | .000 | .000 |
| OA3 | .000 | .000 | .000 | .000 | .000 | .000 | .000 | .000 | .000 |
| OA4 | .000 | .000 | .000 | .000 | .000 | .055 | .000 | .000 | .000 |
| OA5 | .000 | .000 | .000 | .000 | .000 | .077 | .000 | .000 | .000 |
| CS1 | .000 | .000 | .000 | .000 | .000 | .000 | .000 | .042 | .000 |
| CS2 | .000 | .000 | .000 | .000 | .000 | .000 | .000 | .039 | .000 |
| CS3 | .000 | .000 | .000 | .000 | .000 | .000 | .000 | .045 | .000 |
| CS4 | .000 | .000 | .000 | .000 | .000 | .000 | .000 | .038 | .000 |
| CS5 | .000 | .000 | .000 | .000 | .000 | .000 | .000 | .041 | .000 |
| CS6 | .000 | .000 | .000 | .000 | .000 | .000 | .000 | .038 | .000 |
| CS7 | .000 | .000 | .000 | .000 | .000 | .000 | .000 | .000 | .000 |
| CS8 | .000 | .000 | .000 | .000 | .000 | .000 | .000 | .043 | .000 |
| CS9 | .000 | .000 | .000 | .000 | .000 | .000 | .000 | .033 | .000 |
| CS10 | .000 | .000 | .000 | .000 | .000 | .000 | .000 | .033 | .000 |
| CS11 | .000 | .000 | .000 | .000 | .000 | .000 | .000 | .046 | .000 |
| CS12 | .000 | .000 | .000 | .000 | .000 | .000 | .000 | .064 | .000 |
| CS13 | .000 | .000 | .000 | .000 | .000 | .000 | .000 | .059 | .000 |
| TS1 | .000 | .000 | .000 | .000 | .000 | .000 | .036 | .000 | .000 |
| TS2 | .000 | .000 | .000 | .000 | .000 | .000 | .030 | .000 | .000 |
| TS3 | .000 | .000 | .000 | .000 | .000 | .000 | .029 | .000 | .000 |
| TS4 | .000 | .000 | .000 | .000 | .000 | .000 | .032 | .000 | .000 |
| TS5 | .000 | .000 | .000 | .000 | .000 | .000 | .031 | .000 | .000 |
| TS6 | .000 | .000 | .000 | .000 | .000 | .000 | .040 | .000 | .000 |
| TS7 | .000 | .000 | .000 | .000 | .000 | .000 | .000 | .000 | .000 |
| EB1 | .000 | .000 | .140 | .000 | .000 | .000 | .000 | .000 | .000 |
| EB2 | .000 | .000 | .135 | .000 | .000 | .000 | .000 | .000 | .000 |
| EB3 | .000 | .000 | .120 | .000 | .000 | .000 | .000 | .000 | .000 |
| EB4 | .000 | .000 | .123 | .000 | .000 | .000 | .000 | .000 | .000 |
| EB5 | .000 | .000 | .000 | .000 | .000 | .000 | .000 | .000 | .000 |
| EX3 | .000 | .000 | .000 | .000 | .000 | .000 | .000 | .000 | .095 |
| EX2 | .000 | .000 | .000 | .000 | .000 | .000 | .000 | .000 | .098 |
| EX1 | .000 | .000 | .000 | .000 | .000 | .000 | .000 | .000 | .000 |

##### Standardized Direct Effects - Standard Errors (Group number 1 - Default model)

|  | Perceive\_SchoolClimate | Perseverance | Exercise\_Benefits | Enduring\_Enthusiasm | Unrelenting\_Efforts | Opportunities\_forAutonomy | Teacher\_Support | Classmate\_Support | Exercise\_Behaviour |
| --- | --- | --- | --- | --- | --- | --- | --- | --- | --- |
| Perseverance | .053 | .000 | .000 | .000 | .000 | .000 | .000 | .000 | .000 |
| Exercise\_Benefits | .069 | .000 | .000 | .000 | .000 | .000 | .000 | .000 | .000 |
| Enduring\_Enthusiasm | .000 | .075 | .000 | .000 | .000 | .000 | .000 | .000 | .000 |
| Unrelenting\_Efforts | .000 | .073 | .000 | .000 | .000 | .000 | .000 | .000 | .000 |
| Opportunities\_forAutonomy | .057 | .000 | .000 | .000 | .000 | .000 | .000 | .000 | .000 |
| Teacher\_Support | .063 | .000 | .000 | .000 | .000 | .000 | .000 | .000 | .000 |
| Classmate\_Support | .052 | .000 | .000 | .000 | .000 | .000 | .000 | .000 | .000 |
| Exercise\_Behaviour | .061 | .058 | .065 | .000 | .000 | .000 | .000 | .000 | .000 |
| EE6 | .000 | .000 | .000 | .022 | .000 | .000 | .000 | .000 | .000 |
| EE5 | .000 | .000 | .000 | .014 | .000 | .000 | .000 | .000 | .000 |
| EE4 | .000 | .000 | .000 | .015 | .000 | .000 | .000 | .000 | .000 |
| EE3 | .000 | .000 | .000 | .014 | .000 | .000 | .000 | .000 | .000 |
| EE2 | .000 | .000 | .000 | .015 | .000 | .000 | .000 | .000 | .000 |
| EE1 | .000 | .000 | .000 | .015 | .000 | .000 | .000 | .000 | .000 |
| UE6 | .000 | .000 | .000 | .000 | .031 | .000 | .000 | .000 | .000 |
| UE5 | .000 | .000 | .000 | .000 | .015 | .000 | .000 | .000 | .000 |
| UE4 | .000 | .000 | .000 | .000 | .015 | .000 | .000 | .000 | .000 |
| UE3 | .000 | .000 | .000 | .000 | .015 | .000 | .000 | .000 | .000 |
| UE2 | .000 | .000 | .000 | .000 | .017 | .000 | .000 | .000 | .000 |
| UE1 | .000 | .000 | .000 | .000 | .019 | .000 | .000 | .000 | .000 |
| OA1 | .000 | .000 | .000 | .000 | .000 | .026 | .000 | .000 | .000 |
| OA2 | .000 | .000 | .000 | .000 | .000 | .033 | .000 | .000 | .000 |
| OA3 | .000 | .000 | .000 | .000 | .000 | .031 | .000 | .000 | .000 |
| OA4 | .000 | .000 | .000 | .000 | .000 | .029 | .000 | .000 | .000 |
| OA5 | .000 | .000 | .000 | .000 | .000 | .021 | .000 | .000 | .000 |
| CS1 | .000 | .000 | .000 | .000 | .000 | .000 | .000 | .016 | .000 |
| CS2 | .000 | .000 | .000 | .000 | .000 | .000 | .000 | .018 | .000 |
| CS3 | .000 | .000 | .000 | .000 | .000 | .000 | .000 | .025 | .000 |
| CS4 | .000 | .000 | .000 | .000 | .000 | .000 | .000 | .015 | .000 |
| CS5 | .000 | .000 | .000 | .000 | .000 | .000 | .000 | .017 | .000 |
| CS6 | .000 | .000 | .000 | .000 | .000 | .000 | .000 | .015 | .000 |
| CS7 | .000 | .000 | .000 | .000 | .000 | .000 | .000 | .016 | .000 |
| CS8 | .000 | .000 | .000 | .000 | .000 | .000 | .000 | .016 | .000 |
| CS9 | .000 | .000 | .000 | .000 | .000 | .000 | .000 | .014 | .000 |
| CS10 | .000 | .000 | .000 | .000 | .000 | .000 | .000 | .015 | .000 |
| CS11 | .000 | .000 | .000 | .000 | .000 | .000 | .000 | .015 | .000 |
| CS12 | .000 | .000 | .000 | .000 | .000 | .000 | .000 | .013 | .000 |
| CS13 | .000 | .000 | .000 | .000 | .000 | .000 | .000 | .019 | .000 |
| TS1 | .000 | .000 | .000 | .000 | .000 | .000 | .017 | .000 | .000 |
| TS2 | .000 | .000 | .000 | .000 | .000 | .000 | .012 | .000 | .000 |
| TS3 | .000 | .000 | .000 | .000 | .000 | .000 | .012 | .000 | .000 |
| TS4 | .000 | .000 | .000 | .000 | .000 | .000 | .016 | .000 | .000 |
| TS5 | .000 | .000 | .000 | .000 | .000 | .000 | .015 | .000 | .000 |
| TS6 | .000 | .000 | .000 | .000 | .000 | .000 | .021 | .000 | .000 |
| TS7 | .000 | .000 | .000 | .000 | .000 | .000 | .019 | .000 | .000 |
| EB1 | .000 | .000 | .048 | .000 | .000 | .000 | .000 | .000 | .000 |
| EB2 | .000 | .000 | .049 | .000 | .000 | .000 | .000 | .000 | .000 |
| EB3 | .000 | .000 | .052 | .000 | .000 | .000 | .000 | .000 | .000 |
| EB4 | .000 | .000 | .047 | .000 | .000 | .000 | .000 | .000 | .000 |
| EB5 | .000 | .000 | .047 | .000 | .000 | .000 | .000 | .000 | .000 |
| EX3 | .000 | .000 | .000 | .000 | .000 | .000 | .000 | .000 | .039 |
| EX2 | .000 | .000 | .000 | .000 | .000 | .000 | .000 | .000 | .041 |
| EX1 | .000 | .000 | .000 | .000 | .000 | .000 | .000 | .000 | .039 |

##### Indirect Effects - Standard Errors (Group number 1 - Default model)

|  | Perceive\_SchoolClimate | Perseverance | Exercise\_Benefits | Enduring\_Enthusiasm | Unrelenting\_Efforts | Opportunities\_forAutonomy | Teacher\_Support | Classmate\_Support | Exercise\_Behaviour |
| --- | --- | --- | --- | --- | --- | --- | --- | --- | --- |
| Perseverance | .000 | .000 | .000 | .000 | .000 | .000 | .000 | .000 | .000 |
| Exercise\_Benefits | .000 | .000 | .000 | .000 | .000 | .000 | .000 | .000 | .000 |
| Enduring\_Enthusiasm | .147 | .000 | .000 | .000 | .000 | .000 | .000 | .000 | .000 |
| Unrelenting\_Efforts | .124 | .000 | .000 | .000 | .000 | .000 | .000 | .000 | .000 |
| Opportunities\_forAutonomy | .000 | .000 | .000 | .000 | .000 | .000 | .000 | .000 | .000 |
| Teacher\_Support | .000 | .000 | .000 | .000 | .000 | .000 | .000 | .000 | .000 |
| Classmate\_Support | .000 | .000 | .000 | .000 | .000 | .000 | .000 | .000 | .000 |
| Exercise\_Behaviour | .071 | .000 | .000 | .000 | .000 | .000 | .000 | .000 | .000 |
| EE6 | .143 | .213 | .000 | .000 | .000 | .000 | .000 | .000 | .000 |
| EE5 | .151 | .225 | .000 | .000 | .000 | .000 | .000 | .000 | .000 |
| EE4 | .147 | .219 | .000 | .000 | .000 | .000 | .000 | .000 | .000 |
| EE3 | .152 | .227 | .000 | .000 | .000 | .000 | .000 | .000 | .000 |
| EE2 | .145 | .212 | .000 | .000 | .000 | .000 | .000 | .000 | .000 |
| EE1 | .147 | .219 | .000 | .000 | .000 | .000 | .000 | .000 | .000 |
| UE6 | .135 | .051 | .000 | .000 | .000 | .000 | .000 | .000 | .000 |
| UE5 | .132 | .038 | .000 | .000 | .000 | .000 | .000 | .000 | .000 |
| UE4 | .139 | .041 | .000 | .000 | .000 | .000 | .000 | .000 | .000 |
| UE3 | .139 | .041 | .000 | .000 | .000 | .000 | .000 | .000 | .000 |
| UE2 | .124 | .037 | .000 | .000 | .000 | .000 | .000 | .000 | .000 |
| UE1 | .124 | .000 | .000 | .000 | .000 | .000 | .000 | .000 | .000 |
| OA1 | .168 | .000 | .000 | .000 | .000 | .000 | .000 | .000 | .000 |
| OA2 | .157 | .000 | .000 | .000 | .000 | .000 | .000 | .000 | .000 |
| OA3 | .158 | .000 | .000 | .000 | .000 | .000 | .000 | .000 | .000 |
| OA4 | .158 | .000 | .000 | .000 | .000 | .000 | .000 | .000 | .000 |
| OA5 | .202 | .000 | .000 | .000 | .000 | .000 | .000 | .000 | .000 |
| CS1 | .042 | .000 | .000 | .000 | .000 | .000 | .000 | .000 | .000 |
| CS2 | .039 | .000 | .000 | .000 | .000 | .000 | .000 | .000 | .000 |
| CS3 | .045 | .000 | .000 | .000 | .000 | .000 | .000 | .000 | .000 |
| CS4 | .038 | .000 | .000 | .000 | .000 | .000 | .000 | .000 | .000 |
| CS5 | .041 | .000 | .000 | .000 | .000 | .000 | .000 | .000 | .000 |
| CS6 | .038 | .000 | .000 | .000 | .000 | .000 | .000 | .000 | .000 |
| CS7 | .000 | .000 | .000 | .000 | .000 | .000 | .000 | .000 | .000 |
| CS8 | .043 | .000 | .000 | .000 | .000 | .000 | .000 | .000 | .000 |
| CS9 | .033 | .000 | .000 | .000 | .000 | .000 | .000 | .000 | .000 |
| CS10 | .033 | .000 | .000 | .000 | .000 | .000 | .000 | .000 | .000 |
| CS11 | .046 | .000 | .000 | .000 | .000 | .000 | .000 | .000 | .000 |
| CS12 | .064 | .000 | .000 | .000 | .000 | .000 | .000 | .000 | .000 |
| CS13 | .059 | .000 | .000 | .000 | .000 | .000 | .000 | .000 | .000 |
| TS1 | .142 | .000 | .000 | .000 | .000 | .000 | .000 | .000 | .000 |
| TS2 | .154 | .000 | .000 | .000 | .000 | .000 | .000 | .000 | .000 |
| TS3 | .152 | .000 | .000 | .000 | .000 | .000 | .000 | .000 | .000 |
| TS4 | .150 | .000 | .000 | .000 | .000 | .000 | .000 | .000 | .000 |
| TS5 | .155 | .000 | .000 | .000 | .000 | .000 | .000 | .000 | .000 |
| TS6 | .138 | .000 | .000 | .000 | .000 | .000 | .000 | .000 | .000 |
| TS7 | .168 | .000 | .000 | .000 | .000 | .000 | .000 | .000 | .000 |
| EB1 | .097 | .000 | .000 | .000 | .000 | .000 | .000 | .000 | .000 |
| EB2 | .090 | .000 | .000 | .000 | .000 | .000 | .000 | .000 | .000 |
| EB3 | .087 | .000 | .000 | .000 | .000 | .000 | .000 | .000 | .000 |
| EB4 | .093 | .000 | .000 | .000 | .000 | .000 | .000 | .000 | .000 |
| EB5 | .088 | .000 | .000 | .000 | .000 | .000 | .000 | .000 | .000 |
| EX3 | .151 | .066 | .154 | .000 | .000 | .000 | .000 | .000 | .000 |
| EX2 | .156 | .072 | .154 | .000 | .000 | .000 | .000 | .000 | .000 |
| EX1 | .150 | .063 | .144 | .000 | .000 | .000 | .000 | .000 | .000 |

##### Standardized Indirect Effects - Standard Errors (Group number 1 - Default model)

|  | Perceive\_SchoolClimate | Perseverance | Exercise\_Benefits | Enduring\_Enthusiasm | Unrelenting\_Efforts | Opportunities\_forAutonomy | Teacher\_Support | Classmate\_Support | Exercise\_Behaviour |
| --- | --- | --- | --- | --- | --- | --- | --- | --- | --- |
| Perseverance | .000 | .000 | .000 | .000 | .000 | .000 | .000 | .000 | .000 |
| Exercise\_Benefits | .000 | .000 | .000 | .000 | .000 | .000 | .000 | .000 | .000 |
| Enduring\_Enthusiasm | .047 | .000 | .000 | .000 | .000 | .000 | .000 | .000 | .000 |
| Unrelenting\_Efforts | .045 | .000 | .000 | .000 | .000 | .000 | .000 | .000 | .000 |
| Opportunities\_forAutonomy | .000 | .000 | .000 | .000 | .000 | .000 | .000 | .000 | .000 |
| Teacher\_Support | .000 | .000 | .000 | .000 | .000 | .000 | .000 | .000 | .000 |
| Classmate\_Support | .000 | .000 | .000 | .000 | .000 | .000 | .000 | .000 | .000 |
| Exercise\_Behaviour | .030 | .000 | .000 | .000 | .000 | .000 | .000 | .000 | .000 |
| EE6 | .037 | .062 | .000 | .000 | .000 | .000 | .000 | .000 | .000 |
| EE5 | .040 | .066 | .000 | .000 | .000 | .000 | .000 | .000 | .000 |
| EE4 | .040 | .065 | .000 | .000 | .000 | .000 | .000 | .000 | .000 |
| EE3 | .040 | .066 | .000 | .000 | .000 | .000 | .000 | .000 | .000 |
| EE2 | .040 | .064 | .000 | .000 | .000 | .000 | .000 | .000 | .000 |
| EE1 | .039 | .065 | .000 | .000 | .000 | .000 | .000 | .000 | .000 |
| UE6 | .034 | .059 | .000 | .000 | .000 | .000 | .000 | .000 | .000 |
| UE5 | .038 | .064 | .000 | .000 | .000 | .000 | .000 | .000 | .000 |
| UE4 | .039 | .064 | .000 | .000 | .000 | .000 | .000 | .000 | .000 |
| UE3 | .039 | .064 | .000 | .000 | .000 | .000 | .000 | .000 | .000 |
| UE2 | .037 | .062 | .000 | .000 | .000 | .000 | .000 | .000 | .000 |
| UE1 | .036 | .060 | .000 | .000 | .000 | .000 | .000 | .000 | .000 |
| OA1 | .052 | .000 | .000 | .000 | .000 | .000 | .000 | .000 | .000 |
| OA2 | .052 | .000 | .000 | .000 | .000 | .000 | .000 | .000 | .000 |
| OA3 | .053 | .000 | .000 | .000 | .000 | .000 | .000 | .000 | .000 |
| OA4 | .052 | .000 | .000 | .000 | .000 | .000 | .000 | .000 | .000 |
| OA5 | .047 | .000 | .000 | .000 | .000 | .000 | .000 | .000 | .000 |
| CS1 | .049 | .000 | .000 | .000 | .000 | .000 | .000 | .000 | .000 |
| CS2 | .048 | .000 | .000 | .000 | .000 | .000 | .000 | .000 | .000 |
| CS3 | .049 | .000 | .000 | .000 | .000 | .000 | .000 | .000 | .000 |
| CS4 | .050 | .000 | .000 | .000 | .000 | .000 | .000 | .000 | .000 |
| CS5 | .049 | .000 | .000 | .000 | .000 | .000 | .000 | .000 | .000 |
| CS6 | .049 | .000 | .000 | .000 | .000 | .000 | .000 | .000 | .000 |
| CS7 | .049 | .000 | .000 | .000 | .000 | .000 | .000 | .000 | .000 |
| CS8 | .048 | .000 | .000 | .000 | .000 | .000 | .000 | .000 | .000 |
| CS9 | .049 | .000 | .000 | .000 | .000 | .000 | .000 | .000 | .000 |
| CS10 | .049 | .000 | .000 | .000 | .000 | .000 | .000 | .000 | .000 |
| CS11 | .049 | .000 | .000 | .000 | .000 | .000 | .000 | .000 | .000 |
| CS12 | .047 | .000 | .000 | .000 | .000 | .000 | .000 | .000 | .000 |
| CS13 | .047 | .000 | .000 | .000 | .000 | .000 | .000 | .000 | .000 |
| TS1 | .057 | .000 | .000 | .000 | .000 | .000 | .000 | .000 | .000 |
| TS2 | .060 | .000 | .000 | .000 | .000 | .000 | .000 | .000 | .000 |
| TS3 | .060 | .000 | .000 | .000 | .000 | .000 | .000 | .000 | .000 |
| TS4 | .059 | .000 | .000 | .000 | .000 | .000 | .000 | .000 | .000 |
| TS5 | .059 | .000 | .000 | .000 | .000 | .000 | .000 | .000 | .000 |
| TS6 | .055 | .000 | .000 | .000 | .000 | .000 | .000 | .000 | .000 |
| TS7 | .055 | .000 | .000 | .000 | .000 | .000 | .000 | .000 | .000 |
| EB1 | .044 | .000 | .000 | .000 | .000 | .000 | .000 | .000 | .000 |
| EB2 | .041 | .000 | .000 | .000 | .000 | .000 | .000 | .000 | .000 |
| EB3 | .040 | .000 | .000 | .000 | .000 | .000 | .000 | .000 | .000 |
| EB4 | .042 | .000 | .000 | .000 | .000 | .000 | .000 | .000 | .000 |
| EB5 | .037 | .000 | .000 | .000 | .000 | .000 | .000 | .000 | .000 |
| EX3 | .040 | .039 | .043 | .000 | .000 | .000 | .000 | .000 | .000 |
| EX2 | .044 | .045 | .046 | .000 | .000 | .000 | .000 | .000 | .000 |
| EX1 | .042 | .040 | .043 | .000 | .000 | .000 | .000 | .000 | .000 |

##### Bootstrap Confidence (Group number 1 - Default model)

##### Percentile method (Group number 1 - Default model)

##### 95% confidence intervals (percentile method)

##### Scalar Estimates (Group number 1 - Default model)

##### Regression Weights: (Group number 1 - Default model)

| Parameter | | | Estimate | Lower | Upper | P |
| --- | --- | --- | --- | --- | --- | --- |
| Perseverance | <--- | Perceive\_SchoolClimate | .348 | .123 | .603 | .003 |
| Exercise\_Benefits | <--- | Perceive\_SchoolClimate | .276 | .126 | .476 | .000 |
| Exercise\_Behaviour | <--- | Perceive\_SchoolClimate | .330 | .101 | .632 | .004 |
| Classmate\_Support | <--- | Perceive\_SchoolClimate | 1.000 | 1.000 | 1.000 | ... |
| Teacher\_Support | <--- | Perceive\_SchoolClimate | 1.118 | .808 | 1.467 | .000 |
| Opportunities\_forAutonomy | <--- | Perceive\_SchoolClimate | 1.241 | .968 | 1.585 | .000 |
| Unrelenting\_Efforts | <--- | Perseverance | 1.000 | 1.000 | 1.000 | ... |
| Exercise\_Behaviour | <--- | Exercise\_Benefits | .512 | .267 | .836 | .000 |
| Exercise\_Behaviour | <--- | Perseverance | .257 | .139 | .390 | .000 |
| Enduring\_Enthusiasm | <--- | Perseverance | 1.178 | .843 | 1.698 | .000 |
| EX1 | <--- | Exercise\_Behaviour | 1.000 | 1.000 | 1.000 | ... |
| EX2 | <--- | Exercise\_Behaviour | 1.114 | .932 | 1.318 | .000 |
| EX3 | <--- | Exercise\_Behaviour | 1.052 | .877 | 1.254 | .000 |
| EB5 | <--- | Exercise\_Benefits | 1.000 | 1.000 | 1.000 | ... |
| EB4 | <--- | Exercise\_Benefits | 1.103 | .900 | 1.393 | .000 |
| EB3 | <--- | Exercise\_Benefits | .972 | .766 | 1.239 | .000 |
| EB2 | <--- | Exercise\_Benefits | 1.085 | .858 | 1.380 | .000 |
| EB1 | <--- | Exercise\_Benefits | 1.138 | .909 | 1.463 | .000 |
| TS7 | <--- | Teacher\_Support | 1.000 | 1.000 | 1.000 | ... |
| TS6 | <--- | Teacher\_Support | .787 | .702 | .858 | .000 |
| TS5 | <--- | Teacher\_Support | .900 | .836 | .959 | .000 |
| TS4 | <--- | Teacher\_Support | .882 | .814 | .942 | .000 |
| TS3 | <--- | Teacher\_Support | .895 | .835 | .949 | .000 |
| TS2 | <--- | Teacher\_Support | .904 | .842 | .962 | .000 |
| TS1 | <--- | Teacher\_Support | .813 | .739 | .877 | .000 |
| CS11 | <--- | Classmate\_Support | 1.054 | .971 | 1.152 | .000 |
| CS10 | <--- | Classmate\_Support | 1.040 | .981 | 1.110 | .000 |
| CS9 | <--- | Classmate\_Support | 1.079 | 1.018 | 1.147 | .000 |
| CS8 | <--- | Classmate\_Support | 1.023 | .941 | 1.109 | .000 |
| CS7 | <--- | Classmate\_Support | 1.000 | 1.000 | 1.000 | ... |
| CS6 | <--- | Classmate\_Support | 1.036 | .967 | 1.115 | .000 |
| CS5 | <--- | Classmate\_Support | 1.025 | .949 | 1.113 | .000 |
| CS4 | <--- | Classmate\_Support | 1.019 | .949 | 1.098 | .000 |
| CS3 | <--- | Classmate\_Support | 1.028 | .947 | 1.125 | .000 |
| OA5 | <--- | Opportunities\_forAutonomy | 1.302 | 1.178 | 1.477 | .000 |
| OA4 | <--- | Opportunities\_forAutonomy | .974 | .867 | 1.084 | .000 |
| OA3 | <--- | Opportunities\_forAutonomy | 1.000 | 1.000 | 1.000 | ... |
| OA2 | <--- | Opportunities\_forAutonomy | .953 | .862 | 1.046 | .000 |
| OA1 | <--- | Opportunities\_forAutonomy | 1.009 | .907 | 1.118 | .000 |
| UE1 | <--- | Unrelenting\_Efforts | 1.000 | 1.000 | 1.000 | ... |
| UE2 | <--- | Unrelenting\_Efforts | 1.004 | .933 | 1.078 | .000 |
| UE3 | <--- | Unrelenting\_Efforts | 1.124 | 1.048 | 1.208 | .000 |
| UE4 | <--- | Unrelenting\_Efforts | 1.125 | 1.048 | 1.210 | .000 |
| UE5 | <--- | Unrelenting\_Efforts | 1.061 | .991 | 1.141 | .000 |
| UE6 | <--- | Unrelenting\_Efforts | 1.087 | .989 | 1.188 | .000 |
| EE1 | <--- | Enduring\_Enthusiasm | 1.000 | 1.000 | 1.000 | ... |
| EE2 | <--- | Enduring\_Enthusiasm | .978 | .918 | 1.038 | .000 |
| EE3 | <--- | Enduring\_Enthusiasm | 1.036 | .976 | 1.099 | .000 |
| EE4 | <--- | Enduring\_Enthusiasm | .995 | .933 | 1.061 | .000 |
| EE5 | <--- | Enduring\_Enthusiasm | 1.020 | .965 | 1.079 | .000 |
| EE6 | <--- | Enduring\_Enthusiasm | .961 | .893 | 1.031 | .000 |
| CS1 | <--- | Classmate\_Support | 1.017 | .939 | 1.104 | .000 |
| CS13 | <--- | Classmate\_Support | 1.204 | 1.102 | 1.334 | .000 |
| CS12 | <--- | Classmate\_Support | 1.284 | 1.178 | 1.428 | .000 |
| CS2 | <--- | Classmate\_Support | .991 | .917 | 1.073 | .000 |

##### Standardized Regression Weights: (Group number 1 - Default model)

| Parameter | | | Estimate | Lower | Upper | P |
| --- | --- | --- | --- | --- | --- | --- |
| Perseverance | <--- | Perceive\_SchoolClimate | .176 | .066 | .270 | .003 |
| Exercise\_Benefits | <--- | Perceive\_SchoolClimate | .265 | .131 | .403 | .000 |
| Exercise\_Behaviour | <--- | Perceive\_SchoolClimate | .169 | .054 | .295 | .004 |
| Classmate\_Support | <--- | Perceive\_SchoolClimate | .671 | .565 | .773 | .000 |
| Teacher\_Support | <--- | Perceive\_SchoolClimate | .550 | .423 | .670 | .000 |
| Opportunities\_forAutonomy | <--- | Perceive\_SchoolClimate | .845 | .727 | .950 | .000 |
| Unrelenting\_Efforts | <--- | Perseverance | .820 | .685 | .967 | .000 |
| Exercise\_Behaviour | <--- | Exercise\_Benefits | .274 | .149 | .399 | .000 |
| Exercise\_Behaviour | <--- | Perseverance | .261 | .147 | .375 | .000 |
| Enduring\_Enthusiasm | <--- | Perseverance | .842 | .708 | 1.002 | .000 |
| EX1 | <--- | Exercise\_Behaviour | .645 | .568 | .719 | .000 |
| EX2 | <--- | Exercise\_Behaviour | .727 | .641 | .805 | .000 |
| EX3 | <--- | Exercise\_Behaviour | .643 | .564 | .716 | .000 |
| EB5 | <--- | Exercise\_Benefits | .504 | .406 | .591 | .000 |
| EB4 | <--- | Exercise\_Benefits | .598 | .500 | .682 | .000 |
| EB3 | <--- | Exercise\_Benefits | .531 | .424 | .625 | .000 |
| EB2 | <--- | Exercise\_Benefits | .599 | .493 | .685 | .000 |
| EB1 | <--- | Exercise\_Benefits | .605 | .500 | .691 | .000 |
| TS7 | <--- | Teacher\_Support | .850 | .814 | .886 | .000 |
| TS6 | <--- | Teacher\_Support | .863 | .817 | .899 | .000 |
| TS5 | <--- | Teacher\_Support | .920 | .889 | .946 | .000 |
| TS4 | <--- | Teacher\_Support | .915 | .881 | .943 | .000 |
| TS3 | <--- | Teacher\_Support | .946 | .920 | .968 | .000 |
| TS2 | <--- | Teacher\_Support | .937 | .911 | .959 | .000 |
| TS1 | <--- | Teacher\_Support | .900 | .862 | .930 | .000 |
| CS11 | <--- | Classmate\_Support | .915 | .885 | .942 | .000 |
| CS10 | <--- | Classmate\_Support | .911 | .879 | .938 | .000 |
| CS9 | <--- | Classmate\_Support | .918 | .888 | .943 | .000 |
| CS8 | <--- | Classmate\_Support | .905 | .870 | .933 | .000 |
| CS7 | <--- | Classmate\_Support | .911 | .876 | .939 | .000 |
| CS6 | <--- | Classmate\_Support | .913 | .880 | .938 | .000 |
| CS5 | <--- | Classmate\_Support | .900 | .865 | .930 | .000 |
| CS4 | <--- | Classmate\_Support | .923 | .891 | .949 | .000 |
| CS3 | <--- | Classmate\_Support | .856 | .801 | .899 | .000 |
| OA5 | <--- | Opportunities\_forAutonomy | .793 | .752 | .833 | .000 |
| OA4 | <--- | Opportunities\_forAutonomy | .841 | .778 | .891 | .000 |
| OA3 | <--- | Opportunities\_forAutonomy | .773 | .707 | .827 | .000 |
| OA2 | <--- | Opportunities\_forAutonomy | .744 | .673 | .802 | .000 |
| OA1 | <--- | Opportunities\_forAutonomy | .836 | .781 | .881 | .000 |
| UE1 | <--- | Unrelenting\_Efforts | .793 | .754 | .830 | .000 |
| UE2 | <--- | Unrelenting\_Efforts | .818 | .783 | .850 | .000 |
| UE3 | <--- | Unrelenting\_Efforts | .855 | .823 | .884 | .000 |
| UE4 | <--- | Unrelenting\_Efforts | .854 | .823 | .883 | .000 |
| UE5 | <--- | Unrelenting\_Efforts | .845 | .815 | .873 | .000 |
| UE6 | <--- | Unrelenting\_Efforts | .741 | .676 | .798 | .000 |
| EE1 | <--- | Enduring\_Enthusiasm | .835 | .804 | .863 | .000 |
| EE2 | <--- | Enduring\_Enthusiasm | .846 | .815 | .875 | .000 |
| EE3 | <--- | Enduring\_Enthusiasm | .863 | .833 | .889 | .000 |
| EE4 | <--- | Enduring\_Enthusiasm | .854 | .821 | .882 | .000 |
| EE5 | <--- | Enduring\_Enthusiasm | .860 | .830 | .886 | .000 |
| EE6 | <--- | Enduring\_Enthusiasm | .781 | .736 | .822 | .000 |
| CS1 | <--- | Classmate\_Support | .905 | .871 | .933 | .000 |
| CS13 | <--- | Classmate\_Support | .834 | .795 | .870 | .000 |
| CS12 | <--- | Classmate\_Support | .861 | .834 | .886 | .000 |
| CS2 | <--- | Classmate\_Support | .888 | .849 | .919 | .000 |

##### Variances: (Group number 1 - Default model)

| Parameter | | | Estimate | Lower | Upper | P |
| --- | --- | --- | --- | --- | --- | --- |
| Perceive\_SchoolClimate |  |  | .139 | .087 | .202 | .000 |
| e3 |  |  | .140 | .085 | .203 | .000 |
| e1 |  |  | .528 | .347 | .768 | .000 |
| e6 |  |  | .399 | .297 | .498 | .000 |
| e7 |  |  | .170 | .115 | .229 | .000 |
| e8 |  |  | .086 | .027 | .148 | .006 |
| e4 |  |  | .265 | .052 | .436 | .020 |
| e5 |  |  | .309 | -.005 | .532 | .052 |
| e2 |  |  | .411 | .296 | .532 | .000 |
| e52 |  |  | .737 | .609 | .856 | .000 |
| e53 |  |  | .581 | .439 | .720 | .000 |
| e54 |  |  | .828 | .691 | .960 | .000 |
| e51 |  |  | .443 | .385 | .496 | .000 |
| e50 |  |  | .329 | .275 | .379 | .000 |
| e49 |  |  | .362 | .308 | .409 | .000 |
| e48 |  |  | .317 | .265 | .368 | .000 |
| e47 |  |  | .338 | .280 | .393 | .000 |
| e15 |  |  | .220 | .158 | .282 | .000 |
| e14 |  |  | .122 | .094 | .151 | .000 |
| e13 |  |  | .085 | .056 | .114 | .000 |
| e12 |  |  | .087 | .058 | .119 | .000 |
| e11 |  |  | .054 | .032 | .078 | .000 |
| e10 |  |  | .065 | .042 | .089 | .000 |
| e9 |  |  | .089 | .064 | .115 | .000 |
| e29 |  |  | .196 | .148 | .244 | .000 |
| e28 |  |  | .177 | .144 | .211 | .000 |
| e27 |  |  | .067 | .046 | .088 | .000 |
| e26 |  |  | .069 | .048 | .089 | .000 |
| e25 |  |  | .067 | .046 | .089 | .000 |
| e24 |  |  | .072 | .052 | .094 | .000 |
| e23 |  |  | .064 | .045 | .084 | .000 |
| e22 |  |  | .066 | .048 | .086 | .000 |
| e21 |  |  | .076 | .054 | .098 | .000 |
| e20 |  |  | .056 | .038 | .074 | .000 |
| e19 |  |  | .119 | .084 | .162 | .000 |
| e18 |  |  | .082 | .060 | .103 | .000 |
| e16 |  |  | .071 | .051 | .091 | .000 |
| e34 |  |  | .300 | .229 | .372 | .000 |
| e33 |  |  | .118 | .084 | .151 | .000 |
| e32 |  |  | .202 | .161 | .246 | .000 |
| e31 |  |  | .220 | .180 | .260 | .000 |
| e30 |  |  | .131 | .099 | .164 | .000 |
| e35 |  |  | .476 | .403 | .552 | .000 |
| e36 |  |  | .403 | .341 | .465 | .000 |
| e37 |  |  | .376 | .305 | .447 | .000 |
| e38 |  |  | .381 | .311 | .450 | .000 |
| e39 |  |  | .364 | .305 | .423 | .000 |
| e40 |  |  | .785 | .621 | .973 | .000 |
| e41 |  |  | .463 | .393 | .533 | .000 |
| e42 |  |  | .404 | .338 | .470 | .000 |
| e43 |  |  | .392 | .322 | .464 | .000 |
| e44 |  |  | .392 | .322 | .464 | .000 |
| e45 |  |  | .392 | .323 | .462 | .000 |
| e46 |  |  | .627 | .519 | .738 | .000 |

##### Squared Multiple Correlations: (Group number 1 - Default model)

| Parameter | | | Estimate | Lower | Upper | P |
| --- | --- | --- | --- | --- | --- | --- |
| Perseverance |  |  | .031 | .004 | .073 | .000 |
| Exercise\_Benefits |  |  | .070 | .017 | .162 | .000 |
| Enduring\_Enthusiasm |  |  | .710 | .502 | 1.005 | .000 |
| Unrelenting\_Efforts |  |  | .673 | .469 | .935 | .000 |
| Opportunities\_forAutonomy |  |  | .713 | .529 | .902 | .000 |
| Teacher\_Support |  |  | .303 | .179 | .449 | .000 |
| Classmate\_Support |  |  | .450 | .319 | .598 | .000 |
| Exercise\_Behaviour |  |  | .218 | .143 | .328 | .000 |
| EE6 |  |  | .610 | .542 | .676 | .000 |
| EE5 |  |  | .739 | .688 | .785 | .000 |
| EE4 |  |  | .729 | .674 | .778 | .000 |
| EE3 |  |  | .745 | .694 | .791 | .000 |
| EE2 |  |  | .716 | .665 | .765 | .000 |
| EE1 |  |  | .697 | .647 | .745 | .000 |
| UE6 |  |  | .549 | .458 | .636 | .000 |
| UE5 |  |  | .714 | .664 | .762 | .000 |
| UE4 |  |  | .729 | .677 | .779 | .000 |
| UE3 |  |  | .731 | .677 | .782 | .000 |
| UE2 |  |  | .669 | .612 | .722 | .000 |
| UE1 |  |  | .629 | .568 | .688 | .000 |
| OA1 |  |  | .699 | .610 | .777 | .000 |
| OA2 |  |  | .553 | .453 | .643 | .000 |
| OA3 |  |  | .597 | .500 | .684 | .000 |
| OA4 |  |  | .707 | .605 | .794 | .000 |
| OA5 |  |  | .629 | .565 | .694 | .000 |
| CS1 |  |  | .819 | .758 | .871 | .000 |
| CS2 |  |  | .788 | .721 | .845 | .000 |
| CS3 |  |  | .732 | .641 | .808 | .000 |
| CS4 |  |  | .851 | .794 | .901 | .000 |
| CS5 |  |  | .810 | .749 | .865 | .000 |
| CS6 |  |  | .833 | .775 | .881 | .000 |
| CS7 |  |  | .829 | .768 | .881 | .000 |
| CS8 |  |  | .818 | .757 | .871 | .000 |
| CS9 |  |  | .842 | .789 | .889 | .000 |
| CS10 |  |  | .830 | .773 | .879 | .000 |
| CS11 |  |  | .837 | .783 | .887 | .000 |
| CS12 |  |  | .742 | .696 | .785 | .000 |
| CS13 |  |  | .695 | .632 | .757 | .000 |
| TS1 |  |  | .810 | .742 | .866 | .000 |
| TS2 |  |  | .878 | .829 | .920 | .000 |
| TS3 |  |  | .894 | .846 | .937 | .000 |
| TS4 |  |  | .836 | .776 | .889 | .000 |
| TS5 |  |  | .845 | .790 | .896 | .000 |
| TS6 |  |  | .744 | .667 | .808 | .000 |
| TS7 |  |  | .723 | .662 | .785 | .000 |
| EB1 |  |  | .366 | .250 | .477 | .000 |
| EB2 |  |  | .359 | .243 | .470 | .000 |
| EB3 |  |  | .282 | .180 | .391 | .000 |
| EB4 |  |  | .358 | .250 | .466 | .000 |
| EB5 |  |  | .254 | .165 | .349 | .000 |
| EX3 |  |  | .413 | .318 | .512 | .000 |
| EX2 |  |  | .529 | .411 | .648 | .000 |
| EX1 |  |  | .416 | .322 | .517 | .000 |

##### User-defined estimands: (Group number 1 - Default model)

| Parameter | | | Estimate | Lower | Upper | P |
| --- | --- | --- | --- | --- | --- | --- |
| Estimand 1 |  |  | .089 | .028 | .179 | .003 |
| Estimand 2 |  |  | .141 | .053 | .271 | .000 |
| Estimand 3 |  |  | .330 | .101 | .632 | .004 |
| Estimand 4 |  |  | -.052 | -.190 | .068 | .399 |
| Estimand 5 |  |  | .560 | .311 | .896 | .000 |

##### Matrices (Group number 1 - Default model)

##### Total Effects (Group number 1 - Default model)

##### Total Effects - Lower Bounds (PC) (Group number 1 - Default model)

|  | Perceive\_SchoolClimate | Perseverance | Exercise\_Benefits | Enduring\_Enthusiasm | Unrelenting\_Efforts | Opportunities\_forAutonomy | Teacher\_Support | Classmate\_Support | Exercise\_Behaviour |
| --- | --- | --- | --- | --- | --- | --- | --- | --- | --- |
| Perseverance | .123 | .000 | .000 | .000 | .000 | .000 | .000 | .000 | .000 |
| Exercise\_Benefits | .126 | .000 | .000 | .000 | .000 | .000 | .000 | .000 | .000 |
| Enduring\_Enthusiasm | .144 | .843 | .000 | .000 | .000 | .000 | .000 | .000 | .000 |
| Unrelenting\_Efforts | .123 | 1.000 | .000 | .000 | .000 | .000 | .000 | .000 | .000 |
| Opportunities\_forAutonomy | .968 | .000 | .000 | .000 | .000 | .000 | .000 | .000 | .000 |
| Teacher\_Support | .808 | .000 | .000 | .000 | .000 | .000 | .000 | .000 | .000 |
| Classmate\_Support | 1.000 | .000 | .000 | .000 | .000 | .000 | .000 | .000 | .000 |
| Exercise\_Behaviour | .311 | .139 | .267 | .000 | .000 | .000 | .000 | .000 | .000 |
| EE6 | .137 | .805 | .000 | .893 | .000 | .000 | .000 | .000 | .000 |
| EE5 | .144 | .859 | .000 | .965 | .000 | .000 | .000 | .000 | .000 |
| EE4 | .144 | .835 | .000 | .933 | .000 | .000 | .000 | .000 | .000 |
| EE3 | .150 | .874 | .000 | .976 | .000 | .000 | .000 | .000 | .000 |
| EE2 | .139 | .828 | .000 | .918 | .000 | .000 | .000 | .000 | .000 |
| EE1 | .144 | .843 | .000 | 1.000 | .000 | .000 | .000 | .000 | .000 |
| UE6 | .136 | .989 | .000 | .000 | .989 | .000 | .000 | .000 | .000 |
| UE5 | .129 | .991 | .000 | .000 | .991 | .000 | .000 | .000 | .000 |
| UE4 | .139 | 1.048 | .000 | .000 | 1.048 | .000 | .000 | .000 | .000 |
| UE3 | .138 | 1.048 | .000 | .000 | 1.048 | .000 | .000 | .000 | .000 |
| UE2 | .123 | .933 | .000 | .000 | .933 | .000 | .000 | .000 | .000 |
| UE1 | .123 | 1.000 | .000 | .000 | 1.000 | .000 | .000 | .000 | .000 |
| OA1 | .972 | .000 | .000 | .000 | .000 | .907 | .000 | .000 | .000 |
| OA2 | .916 | .000 | .000 | .000 | .000 | .862 | .000 | .000 | .000 |
| OA3 | .968 | .000 | .000 | .000 | .000 | 1.000 | .000 | .000 | .000 |
| OA4 | .941 | .000 | .000 | .000 | .000 | .867 | .000 | .000 | .000 |
| OA5 | 1.278 | .000 | .000 | .000 | .000 | 1.178 | .000 | .000 | .000 |
| CS1 | .939 | .000 | .000 | .000 | .000 | .000 | .000 | .939 | .000 |
| CS2 | .917 | .000 | .000 | .000 | .000 | .000 | .000 | .917 | .000 |
| CS3 | .947 | .000 | .000 | .000 | .000 | .000 | .000 | .947 | .000 |
| CS4 | .949 | .000 | .000 | .000 | .000 | .000 | .000 | .949 | .000 |
| CS5 | .949 | .000 | .000 | .000 | .000 | .000 | .000 | .949 | .000 |
| CS6 | .967 | .000 | .000 | .000 | .000 | .000 | .000 | .967 | .000 |
| CS7 | 1.000 | .000 | .000 | .000 | .000 | .000 | .000 | 1.000 | .000 |
| CS8 | .941 | .000 | .000 | .000 | .000 | .000 | .000 | .941 | .000 |
| CS9 | 1.018 | .000 | .000 | .000 | .000 | .000 | .000 | 1.018 | .000 |
| CS10 | .981 | .000 | .000 | .000 | .000 | .000 | .000 | .981 | .000 |
| CS11 | .971 | .000 | .000 | .000 | .000 | .000 | .000 | .971 | .000 |
| CS12 | 1.178 | .000 | .000 | .000 | .000 | .000 | .000 | 1.178 | .000 |
| CS13 | 1.102 | .000 | .000 | .000 | .000 | .000 | .000 | 1.102 | .000 |
| TS1 | .652 | .000 | .000 | .000 | .000 | .000 | .739 | .000 | .000 |
| TS2 | .728 | .000 | .000 | .000 | .000 | .000 | .842 | .000 | .000 |
| TS3 | .726 | .000 | .000 | .000 | .000 | .000 | .835 | .000 | .000 |
| TS4 | .713 | .000 | .000 | .000 | .000 | .000 | .814 | .000 | .000 |
| TS5 | .724 | .000 | .000 | .000 | .000 | .000 | .836 | .000 | .000 |
| TS6 | .629 | .000 | .000 | .000 | .000 | .000 | .702 | .000 | .000 |
| TS7 | .808 | .000 | .000 | .000 | .000 | .000 | 1.000 | .000 | .000 |
| EB1 | .143 | .000 | .909 | .000 | .000 | .000 | .000 | .000 | .000 |
| EB2 | .141 | .000 | .858 | .000 | .000 | .000 | .000 | .000 | .000 |
| EB3 | .123 | .000 | .766 | .000 | .000 | .000 | .000 | .000 | .000 |
| EB4 | .143 | .000 | .900 | .000 | .000 | .000 | .000 | .000 | .000 |
| EB5 | .126 | .000 | 1.000 | .000 | .000 | .000 | .000 | .000 | .000 |
| EX3 | .333 | .147 | .279 | .000 | .000 | .000 | .000 | .000 | .877 |
| EX2 | .364 | .154 | .305 | .000 | .000 | .000 | .000 | .000 | .932 |
| EX1 | .311 | .139 | .267 | .000 | .000 | .000 | .000 | .000 | 1.000 |

##### Total Effects - Upper Bounds (PC) (Group number 1 - Default model)

|  | Perceive\_SchoolClimate | Perseverance | Exercise\_Benefits | Enduring\_Enthusiasm | Unrelenting\_Efforts | Opportunities\_forAutonomy | Teacher\_Support | Classmate\_Support | Exercise\_Behaviour |
| --- | --- | --- | --- | --- | --- | --- | --- | --- | --- |
| Perseverance | .603 | .000 | .000 | .000 | .000 | .000 | .000 | .000 | .000 |
| Exercise\_Benefits | .476 | .000 | .000 | .000 | .000 | .000 | .000 | .000 | .000 |
| Enduring\_Enthusiasm | .710 | 1.698 | .000 | .000 | .000 | .000 | .000 | .000 | .000 |
| Unrelenting\_Efforts | .603 | 1.000 | .000 | .000 | .000 | .000 | .000 | .000 | .000 |
| Opportunities\_forAutonomy | 1.585 | .000 | .000 | .000 | .000 | .000 | .000 | .000 | .000 |
| Teacher\_Support | 1.467 | .000 | .000 | .000 | .000 | .000 | .000 | .000 | .000 |
| Classmate\_Support | 1.000 | .000 | .000 | .000 | .000 | .000 | .000 | .000 | .000 |
| Exercise\_Behaviour | .896 | .390 | .836 | .000 | .000 | .000 | .000 | .000 | .000 |
| EE6 | .688 | 1.621 | .000 | 1.031 | .000 | .000 | .000 | .000 | .000 |
| EE5 | .734 | 1.723 | .000 | 1.079 | .000 | .000 | .000 | .000 | .000 |
| EE4 | .715 | 1.676 | .000 | 1.061 | .000 | .000 | .000 | .000 | .000 |
| EE3 | .739 | 1.750 | .000 | 1.099 | .000 | .000 | .000 | .000 | .000 |
| EE2 | .698 | 1.650 | .000 | 1.038 | .000 | .000 | .000 | .000 | .000 |
| EE1 | .710 | 1.698 | .000 | 1.000 | .000 | .000 | .000 | .000 | .000 |
| UE6 | .658 | 1.188 | .000 | .000 | 1.188 | .000 | .000 | .000 | .000 |
| UE5 | .637 | 1.141 | .000 | .000 | 1.141 | .000 | .000 | .000 | .000 |
| UE4 | .678 | 1.210 | .000 | .000 | 1.210 | .000 | .000 | .000 | .000 |
| UE3 | .678 | 1.208 | .000 | .000 | 1.208 | .000 | .000 | .000 | .000 |
| UE2 | .602 | 1.078 | .000 | .000 | 1.078 | .000 | .000 | .000 | .000 |
| UE1 | .603 | 1.000 | .000 | .000 | 1.000 | .000 | .000 | .000 | .000 |
| OA1 | 1.618 | .000 | .000 | .000 | .000 | 1.118 | .000 | .000 | .000 |
| OA2 | 1.521 | .000 | .000 | .000 | .000 | 1.046 | .000 | .000 | .000 |
| OA3 | 1.585 | .000 | .000 | .000 | .000 | 1.000 | .000 | .000 | .000 |
| OA4 | 1.550 | .000 | .000 | .000 | .000 | 1.084 | .000 | .000 | .000 |
| OA5 | 2.079 | .000 | .000 | .000 | .000 | 1.477 | .000 | .000 | .000 |
| CS1 | 1.104 | .000 | .000 | .000 | .000 | .000 | .000 | 1.104 | .000 |
| CS2 | 1.073 | .000 | .000 | .000 | .000 | .000 | .000 | 1.073 | .000 |
| CS3 | 1.125 | .000 | .000 | .000 | .000 | .000 | .000 | 1.125 | .000 |
| CS4 | 1.098 | .000 | .000 | .000 | .000 | .000 | .000 | 1.098 | .000 |
| CS5 | 1.113 | .000 | .000 | .000 | .000 | .000 | .000 | 1.113 | .000 |
| CS6 | 1.115 | .000 | .000 | .000 | .000 | .000 | .000 | 1.115 | .000 |
| CS7 | 1.000 | .000 | .000 | .000 | .000 | .000 | .000 | 1.000 | .000 |
| CS8 | 1.109 | .000 | .000 | .000 | .000 | .000 | .000 | 1.109 | .000 |
| CS9 | 1.147 | .000 | .000 | .000 | .000 | .000 | .000 | 1.147 | .000 |
| CS10 | 1.110 | .000 | .000 | .000 | .000 | .000 | .000 | 1.110 | .000 |
| CS11 | 1.152 | .000 | .000 | .000 | .000 | .000 | .000 | 1.152 | .000 |
| CS12 | 1.428 | .000 | .000 | .000 | .000 | .000 | .000 | 1.428 | .000 |
| CS13 | 1.334 | .000 | .000 | .000 | .000 | .000 | .000 | 1.334 | .000 |
| TS1 | 1.205 | .000 | .000 | .000 | .000 | .000 | .877 | .000 | .000 |
| TS2 | 1.333 | .000 | .000 | .000 | .000 | .000 | .962 | .000 | .000 |
| TS3 | 1.324 | .000 | .000 | .000 | .000 | .000 | .949 | .000 | .000 |
| TS4 | 1.299 | .000 | .000 | .000 | .000 | .000 | .942 | .000 | .000 |
| TS5 | 1.332 | .000 | .000 | .000 | .000 | .000 | .959 | .000 | .000 |
| TS6 | 1.168 | .000 | .000 | .000 | .000 | .000 | .858 | .000 | .000 |
| TS7 | 1.467 | .000 | .000 | .000 | .000 | .000 | 1.000 | .000 | .000 |
| EB1 | .527 | .000 | 1.463 | .000 | .000 | .000 | .000 | .000 | .000 |
| EB2 | .496 | .000 | 1.380 | .000 | .000 | .000 | .000 | .000 | .000 |
| EB3 | .468 | .000 | 1.239 | .000 | .000 | .000 | .000 | .000 | .000 |
| EB4 | .507 | .000 | 1.393 | .000 | .000 | .000 | .000 | .000 | .000 |
| EB5 | .476 | .000 | 1.000 | .000 | .000 | .000 | .000 | .000 | .000 |
| EX3 | .924 | .405 | .895 | .000 | .000 | .000 | .000 | .000 | 1.254 |
| EX2 | .970 | .437 | .907 | .000 | .000 | .000 | .000 | .000 | 1.318 |
| EX1 | .896 | .390 | .836 | .000 | .000 | .000 | .000 | .000 | 1.000 |

##### Total Effects - Two Tailed Significance (PC) (Group number 1 - Default model)

|  | Perceive\_SchoolClimate | Perseverance | Exercise\_Benefits | Enduring\_Enthusiasm | Unrelenting\_Efforts | Opportunities\_forAutonomy | Teacher\_Support | Classmate\_Support | Exercise\_Behaviour |
| --- | --- | --- | --- | --- | --- | --- | --- | --- | --- |
| Perseverance | .003 | ... | ... | ... | ... | ... | ... | ... | ... |
| Exercise\_Benefits | .000 | ... | ... | ... | ... | ... | ... | ... | ... |
| Enduring\_Enthusiasm | .003 | .000 | ... | ... | ... | ... | ... | ... | ... |
| Unrelenting\_Efforts | .003 | ... | ... | ... | ... | ... | ... | ... | ... |
| Opportunities\_forAutonomy | .000 | ... | ... | ... | ... | ... | ... | ... | ... |
| Teacher\_Support | .000 | ... | ... | ... | ... | ... | ... | ... | ... |
| Classmate\_Support | ... | ... | ... | ... | ... | ... | ... | ... | ... |
| Exercise\_Behaviour | .000 | .000 | .000 | ... | ... | ... | ... | ... | ... |
| EE6 | .003 | .000 | ... | .000 | ... | ... | ... | ... | ... |
| EE5 | .003 | .000 | ... | .000 | ... | ... | ... | ... | ... |
| EE4 | .003 | .000 | ... | .000 | ... | ... | ... | ... | ... |
| EE3 | .003 | .000 | ... | .000 | ... | ... | ... | ... | ... |
| EE2 | .003 | .000 | ... | .000 | ... | ... | ... | ... | ... |
| EE1 | .003 | .000 | ... | ... | ... | ... | ... | ... | ... |
| UE6 | .003 | .000 | ... | ... | .000 | ... | ... | ... | ... |
| UE5 | .003 | .000 | ... | ... | .000 | ... | ... | ... | ... |
| UE4 | .003 | .000 | ... | ... | .000 | ... | ... | ... | ... |
| UE3 | .003 | .000 | ... | ... | .000 | ... | ... | ... | ... |
| UE2 | .003 | .000 | ... | ... | .000 | ... | ... | ... | ... |
| UE1 | .003 | ... | ... | ... | ... | ... | ... | ... | ... |
| OA1 | .000 | ... | ... | ... | ... | .000 | ... | ... | ... |
| OA2 | .000 | ... | ... | ... | ... | .000 | ... | ... | ... |
| OA3 | .000 | ... | ... | ... | ... | ... | ... | ... | ... |
| OA4 | .000 | ... | ... | ... | ... | .000 | ... | ... | ... |
| OA5 | .000 | ... | ... | ... | ... | .000 | ... | ... | ... |
| CS1 | .000 | ... | ... | ... | ... | ... | ... | .000 | ... |
| CS2 | .000 | ... | ... | ... | ... | ... | ... | .000 | ... |
| CS3 | .000 | ... | ... | ... | ... | ... | ... | .000 | ... |
| CS4 | .000 | ... | ... | ... | ... | ... | ... | .000 | ... |
| CS5 | .000 | ... | ... | ... | ... | ... | ... | .000 | ... |
| CS6 | .000 | ... | ... | ... | ... | ... | ... | .000 | ... |
| CS7 | ... | ... | ... | ... | ... | ... | ... | ... | ... |
| CS8 | .000 | ... | ... | ... | ... | ... | ... | .000 | ... |
| CS9 | .000 | ... | ... | ... | ... | ... | ... | .000 | ... |
| CS10 | .000 | ... | ... | ... | ... | ... | ... | .000 | ... |
| CS11 | .000 | ... | ... | ... | ... | ... | ... | .000 | ... |
| CS12 | .000 | ... | ... | ... | ... | ... | ... | .000 | ... |
| CS13 | .000 | ... | ... | ... | ... | ... | ... | .000 | ... |
| TS1 | .000 | ... | ... | ... | ... | ... | .000 | ... | ... |
| TS2 | .000 | ... | ... | ... | ... | ... | .000 | ... | ... |
| TS3 | .000 | ... | ... | ... | ... | ... | .000 | ... | ... |
| TS4 | .000 | ... | ... | ... | ... | ... | .000 | ... | ... |
| TS5 | .000 | ... | ... | ... | ... | ... | .000 | ... | ... |
| TS6 | .000 | ... | ... | ... | ... | ... | .000 | ... | ... |
| TS7 | .000 | ... | ... | ... | ... | ... | ... | ... | ... |
| EB1 | .000 | ... | .000 | ... | ... | ... | ... | ... | ... |
| EB2 | .000 | ... | .000 | ... | ... | ... | ... | ... | ... |
| EB3 | .000 | ... | .000 | ... | ... | ... | ... | ... | ... |
| EB4 | .000 | ... | .000 | ... | ... | ... | ... | ... | ... |
| EB5 | .000 | ... | ... | ... | ... | ... | ... | ... | ... |
| EX3 | .000 | .000 | .000 | ... | ... | ... | ... | ... | .000 |
| EX2 | .000 | .000 | .000 | ... | ... | ... | ... | ... | .000 |
| EX1 | .000 | .000 | .000 | ... | ... | ... | ... | ... | ... |

##### Standardized Total Effects (Group number 1 - Default model)

##### Standardized Total Effects - Lower Bounds (PC) (Group number 1 - Default model)

|  | Perceive\_SchoolClimate | Perseverance | Exercise\_Benefits | Enduring\_Enthusiasm | Unrelenting\_Efforts | Opportunities\_forAutonomy | Teacher\_Support | Classmate\_Support | Exercise\_Behaviour |
| --- | --- | --- | --- | --- | --- | --- | --- | --- | --- |
| Perseverance | .066 | .000 | .000 | .000 | .000 | .000 | .000 | .000 | .000 |
| Exercise\_Benefits | .131 | .000 | .000 | .000 | .000 | .000 | .000 | .000 | .000 |
| Enduring\_Enthusiasm | .054 | .708 | .000 | .000 | .000 | .000 | .000 | .000 | .000 |
| Unrelenting\_Efforts | .054 | .685 | .000 | .000 | .000 | .000 | .000 | .000 | .000 |
| Opportunities\_forAutonomy | .727 | .000 | .000 | .000 | .000 | .000 | .000 | .000 | .000 |
| Teacher\_Support | .423 | .000 | .000 | .000 | .000 | .000 | .000 | .000 | .000 |
| Classmate\_Support | .565 | .000 | .000 | .000 | .000 | .000 | .000 | .000 | .000 |
| Exercise\_Behaviour | .170 | .147 | .149 | .000 | .000 | .000 | .000 | .000 | .000 |
| EE6 | .043 | .545 | .000 | .736 | .000 | .000 | .000 | .000 | .000 |
| EE5 | .047 | .604 | .000 | .830 | .000 | .000 | .000 | .000 | .000 |
| EE4 | .047 | .602 | .000 | .821 | .000 | .000 | .000 | .000 | .000 |
| EE3 | .047 | .608 | .000 | .833 | .000 | .000 | .000 | .000 | .000 |
| EE2 | .046 | .597 | .000 | .815 | .000 | .000 | .000 | .000 | .000 |
| EE1 | .045 | .587 | .000 | .804 | .000 | .000 | .000 | .000 | .000 |
| UE6 | .040 | .497 | .000 | .000 | .676 | .000 | .000 | .000 | .000 |
| UE5 | .045 | .573 | .000 | .000 | .815 | .000 | .000 | .000 | .000 |
| UE4 | .046 | .580 | .000 | .000 | .823 | .000 | .000 | .000 | .000 |
| UE3 | .045 | .580 | .000 | .000 | .823 | .000 | .000 | .000 | .000 |
| UE2 | .044 | .555 | .000 | .000 | .783 | .000 | .000 | .000 | .000 |
| UE1 | .043 | .536 | .000 | .000 | .754 | .000 | .000 | .000 | .000 |
| OA1 | .601 | .000 | .000 | .000 | .000 | .781 | .000 | .000 | .000 |
| OA2 | .521 | .000 | .000 | .000 | .000 | .673 | .000 | .000 | .000 |
| OA3 | .544 | .000 | .000 | .000 | .000 | .707 | .000 | .000 | .000 |
| OA4 | .605 | .000 | .000 | .000 | .000 | .778 | .000 | .000 | .000 |
| OA5 | .574 | .000 | .000 | .000 | .000 | .752 | .000 | .000 | .000 |
| CS1 | .508 | .000 | .000 | .000 | .000 | .000 | .000 | .871 | .000 |
| CS2 | .498 | .000 | .000 | .000 | .000 | .000 | .000 | .849 | .000 |
| CS3 | .476 | .000 | .000 | .000 | .000 | .000 | .000 | .801 | .000 |
| CS4 | .518 | .000 | .000 | .000 | .000 | .000 | .000 | .891 | .000 |
| CS5 | .507 | .000 | .000 | .000 | .000 | .000 | .000 | .865 | .000 |
| CS6 | .514 | .000 | .000 | .000 | .000 | .000 | .000 | .880 | .000 |
| CS7 | .511 | .000 | .000 | .000 | .000 | .000 | .000 | .876 | .000 |
| CS8 | .509 | .000 | .000 | .000 | .000 | .000 | .000 | .870 | .000 |
| CS9 | .517 | .000 | .000 | .000 | .000 | .000 | .000 | .888 | .000 |
| CS10 | .514 | .000 | .000 | .000 | .000 | .000 | .000 | .879 | .000 |
| CS11 | .515 | .000 | .000 | .000 | .000 | .000 | .000 | .885 | .000 |
| CS12 | .483 | .000 | .000 | .000 | .000 | .000 | .000 | .834 | .000 |
| CS13 | .468 | .000 | .000 | .000 | .000 | .000 | .000 | .795 | .000 |
| TS1 | .382 | .000 | .000 | .000 | .000 | .000 | .862 | .000 | .000 |
| TS2 | .398 | .000 | .000 | .000 | .000 | .000 | .911 | .000 | .000 |
| TS3 | .400 | .000 | .000 | .000 | .000 | .000 | .920 | .000 | .000 |
| TS4 | .385 | .000 | .000 | .000 | .000 | .000 | .881 | .000 | .000 |
| TS5 | .389 | .000 | .000 | .000 | .000 | .000 | .889 | .000 | .000 |
| TS6 | .364 | .000 | .000 | .000 | .000 | .000 | .817 | .000 | .000 |
| TS7 | .358 | .000 | .000 | .000 | .000 | .000 | .814 | .000 | .000 |
| EB1 | .076 | .000 | .500 | .000 | .000 | .000 | .000 | .000 | .000 |
| EB2 | .079 | .000 | .493 | .000 | .000 | .000 | .000 | .000 | .000 |
| EB3 | .067 | .000 | .424 | .000 | .000 | .000 | .000 | .000 | .000 |
| EB4 | .077 | .000 | .500 | .000 | .000 | .000 | .000 | .000 | .000 |
| EB5 | .064 | .000 | .406 | .000 | .000 | .000 | .000 | .000 | .000 |
| EX3 | .109 | .094 | .094 | .000 | .000 | .000 | .000 | .000 | .564 |
| EX2 | .124 | .104 | .110 | .000 | .000 | .000 | .000 | .000 | .641 |
| EX1 | .107 | .092 | .096 | .000 | .000 | .000 | .000 | .000 | .568 |

##### Standardized Total Effects - Upper Bounds (PC) (Group number 1 - Default model)

|  | Perceive\_SchoolClimate | Perseverance | Exercise\_Benefits | Enduring\_Enthusiasm | Unrelenting\_Efforts | Opportunities\_forAutonomy | Teacher\_Support | Classmate\_Support | Exercise\_Behaviour |
| --- | --- | --- | --- | --- | --- | --- | --- | --- | --- |
| Perseverance | .270 | .000 | .000 | .000 | .000 | .000 | .000 | .000 | .000 |
| Exercise\_Benefits | .403 | .000 | .000 | .000 | .000 | .000 | .000 | .000 | .000 |
| Enduring\_Enthusiasm | .237 | 1.002 | .000 | .000 | .000 | .000 | .000 | .000 | .000 |
| Unrelenting\_Efforts | .226 | .967 | .000 | .000 | .000 | .000 | .000 | .000 | .000 |
| Opportunities\_forAutonomy | .950 | .000 | .000 | .000 | .000 | .000 | .000 | .000 | .000 |
| Teacher\_Support | .670 | .000 | .000 | .000 | .000 | .000 | .000 | .000 | .000 |
| Classmate\_Support | .773 | .000 | .000 | .000 | .000 | .000 | .000 | .000 | .000 |
| Exercise\_Behaviour | .410 | .375 | .399 | .000 | .000 | .000 | .000 | .000 | .000 |
| EE6 | .186 | .795 | .000 | .822 | .000 | .000 | .000 | .000 | .000 |
| EE5 | .205 | .866 | .000 | .886 | .000 | .000 | .000 | .000 | .000 |
| EE4 | .203 | .858 | .000 | .882 | .000 | .000 | .000 | .000 | .000 |
| EE3 | .205 | .871 | .000 | .889 | .000 | .000 | .000 | .000 | .000 |
| EE2 | .202 | .849 | .000 | .875 | .000 | .000 | .000 | .000 | .000 |
| EE1 | .198 | .841 | .000 | .863 | .000 | .000 | .000 | .000 | .000 |
| UE6 | .168 | .729 | .000 | .000 | .798 | .000 | .000 | .000 | .000 |
| UE5 | .192 | .822 | .000 | .000 | .873 | .000 | .000 | .000 | .000 |
| UE4 | .195 | .829 | .000 | .000 | .883 | .000 | .000 | .000 | .000 |
| UE3 | .194 | .827 | .000 | .000 | .884 | .000 | .000 | .000 | .000 |
| UE2 | .185 | .792 | .000 | .000 | .850 | .000 | .000 | .000 | .000 |
| UE1 | .181 | .772 | .000 | .000 | .830 | .000 | .000 | .000 | .000 |
| OA1 | .801 | .000 | .000 | .000 | .000 | .881 | .000 | .000 | .000 |
| OA2 | .724 | .000 | .000 | .000 | .000 | .802 | .000 | .000 | .000 |
| OA3 | .751 | .000 | .000 | .000 | .000 | .827 | .000 | .000 | .000 |
| OA4 | .808 | .000 | .000 | .000 | .000 | .891 | .000 | .000 | .000 |
| OA5 | .758 | .000 | .000 | .000 | .000 | .833 | .000 | .000 | .000 |
| CS1 | .701 | .000 | .000 | .000 | .000 | .000 | .000 | .933 | .000 |
| CS2 | .689 | .000 | .000 | .000 | .000 | .000 | .000 | .919 | .000 |
| CS3 | .670 | .000 | .000 | .000 | .000 | .000 | .000 | .899 | .000 |
| CS4 | .715 | .000 | .000 | .000 | .000 | .000 | .000 | .949 | .000 |
| CS5 | .699 | .000 | .000 | .000 | .000 | .000 | .000 | .930 | .000 |
| CS6 | .706 | .000 | .000 | .000 | .000 | .000 | .000 | .938 | .000 |
| CS7 | .706 | .000 | .000 | .000 | .000 | .000 | .000 | .939 | .000 |
| CS8 | .701 | .000 | .000 | .000 | .000 | .000 | .000 | .933 | .000 |
| CS9 | .711 | .000 | .000 | .000 | .000 | .000 | .000 | .943 | .000 |
| CS10 | .705 | .000 | .000 | .000 | .000 | .000 | .000 | .938 | .000 |
| CS11 | .706 | .000 | .000 | .000 | .000 | .000 | .000 | .942 | .000 |
| CS12 | .669 | .000 | .000 | .000 | .000 | .000 | .000 | .886 | .000 |
| CS13 | .651 | .000 | .000 | .000 | .000 | .000 | .000 | .870 | .000 |
| TS1 | .605 | .000 | .000 | .000 | .000 | .000 | .930 | .000 | .000 |
| TS2 | .630 | .000 | .000 | .000 | .000 | .000 | .959 | .000 | .000 |
| TS3 | .636 | .000 | .000 | .000 | .000 | .000 | .968 | .000 | .000 |
| TS4 | .616 | .000 | .000 | .000 | .000 | .000 | .943 | .000 | .000 |
| TS5 | .618 | .000 | .000 | .000 | .000 | .000 | .946 | .000 | .000 |
| TS6 | .580 | .000 | .000 | .000 | .000 | .000 | .899 | .000 | .000 |
| TS7 | .572 | .000 | .000 | .000 | .000 | .000 | .886 | .000 | .000 |
| EB1 | .250 | .000 | .691 | .000 | .000 | .000 | .000 | .000 | .000 |
| EB2 | .240 | .000 | .685 | .000 | .000 | .000 | .000 | .000 | .000 |
| EB3 | .225 | .000 | .625 | .000 | .000 | .000 | .000 | .000 | .000 |
| EB4 | .243 | .000 | .682 | .000 | .000 | .000 | .000 | .000 | .000 |
| EB5 | .210 | .000 | .591 | .000 | .000 | .000 | .000 | .000 | .000 |
| EX3 | .266 | .246 | .261 | .000 | .000 | .000 | .000 | .000 | .716 |
| EX2 | .296 | .280 | .288 | .000 | .000 | .000 | .000 | .000 | .805 |
| EX1 | .273 | .248 | .262 | .000 | .000 | .000 | .000 | .000 | .719 |

##### Standardized Total Effects - Two Tailed Significance (PC) (Group number 1 - Default model)

|  | Perceive\_SchoolClimate | Perseverance | Exercise\_Benefits | Enduring\_Enthusiasm | Unrelenting\_Efforts | Opportunities\_forAutonomy | Teacher\_Support | Classmate\_Support | Exercise\_Behaviour |
| --- | --- | --- | --- | --- | --- | --- | --- | --- | --- |
| Perseverance | .003 | ... | ... | ... | ... | ... | ... | ... | ... |
| Exercise\_Benefits | .000 | ... | ... | ... | ... | ... | ... | ... | ... |
| Enduring\_Enthusiasm | .003 | .000 | ... | ... | ... | ... | ... | ... | ... |
| Unrelenting\_Efforts | .003 | .000 | ... | ... | ... | ... | ... | ... | ... |
| Opportunities\_forAutonomy | .000 | ... | ... | ... | ... | ... | ... | ... | ... |
| Teacher\_Support | .000 | ... | ... | ... | ... | ... | ... | ... | ... |
| Classmate\_Support | .000 | ... | ... | ... | ... | ... | ... | ... | ... |
| Exercise\_Behaviour | .000 | .000 | .000 | ... | ... | ... | ... | ... | ... |
| EE6 | .003 | .000 | ... | .000 | ... | ... | ... | ... | ... |
| EE5 | .003 | .000 | ... | .000 | ... | ... | ... | ... | ... |
| EE4 | .003 | .000 | ... | .000 | ... | ... | ... | ... | ... |
| EE3 | .003 | .000 | ... | .000 | ... | ... | ... | ... | ... |
| EE2 | .003 | .000 | ... | .000 | ... | ... | ... | ... | ... |
| EE1 | .003 | .000 | ... | .000 | ... | ... | ... | ... | ... |
| UE6 | .003 | .000 | ... | ... | .000 | ... | ... | ... | ... |
| UE5 | .003 | .000 | ... | ... | .000 | ... | ... | ... | ... |
| UE4 | .003 | .000 | ... | ... | .000 | ... | ... | ... | ... |
| UE3 | .003 | .000 | ... | ... | .000 | ... | ... | ... | ... |
| UE2 | .003 | .000 | ... | ... | .000 | ... | ... | ... | ... |
| UE1 | .003 | .000 | ... | ... | .000 | ... | ... | ... | ... |
| OA1 | .000 | ... | ... | ... | ... | .000 | ... | ... | ... |
| OA2 | .000 | ... | ... | ... | ... | .000 | ... | ... | ... |
| OA3 | .000 | ... | ... | ... | ... | .000 | ... | ... | ... |
| OA4 | .000 | ... | ... | ... | ... | .000 | ... | ... | ... |
| OA5 | .000 | ... | ... | ... | ... | .000 | ... | ... | ... |
| CS1 | .000 | ... | ... | ... | ... | ... | ... | .000 | ... |
| CS2 | .000 | ... | ... | ... | ... | ... | ... | .000 | ... |
| CS3 | .000 | ... | ... | ... | ... | ... | ... | .000 | ... |
| CS4 | .000 | ... | ... | ... | ... | ... | ... | .000 | ... |
| CS5 | .000 | ... | ... | ... | ... | ... | ... | .000 | ... |
| CS6 | .000 | ... | ... | ... | ... | ... | ... | .000 | ... |
| CS7 | .000 | ... | ... | ... | ... | ... | ... | .000 | ... |
| CS8 | .000 | ... | ... | ... | ... | ... | ... | .000 | ... |
| CS9 | .000 | ... | ... | ... | ... | ... | ... | .000 | ... |
| CS10 | .000 | ... | ... | ... | ... | ... | ... | .000 | ... |
| CS11 | .000 | ... | ... | ... | ... | ... | ... | .000 | ... |
| CS12 | .000 | ... | ... | ... | ... | ... | ... | .000 | ... |
| CS13 | .000 | ... | ... | ... | ... | ... | ... | .000 | ... |
| TS1 | .000 | ... | ... | ... | ... | ... | .000 | ... | ... |
| TS2 | .000 | ... | ... | ... | ... | ... | .000 | ... | ... |
| TS3 | .000 | ... | ... | ... | ... | ... | .000 | ... | ... |
| TS4 | .000 | ... | ... | ... | ... | ... | .000 | ... | ... |
| TS5 | .000 | ... | ... | ... | ... | ... | .000 | ... | ... |
| TS6 | .000 | ... | ... | ... | ... | ... | .000 | ... | ... |
| TS7 | .000 | ... | ... | ... | ... | ... | .000 | ... | ... |
| EB1 | .000 | ... | .000 | ... | ... | ... | ... | ... | ... |
| EB2 | .000 | ... | .000 | ... | ... | ... | ... | ... | ... |
| EB3 | .000 | ... | .000 | ... | ... | ... | ... | ... | ... |
| EB4 | .000 | ... | .000 | ... | ... | ... | ... | ... | ... |
| EB5 | .000 | ... | .000 | ... | ... | ... | ... | ... | ... |
| EX3 | .000 | .000 | .000 | ... | ... | ... | ... | ... | .000 |
| EX2 | .000 | .000 | .000 | ... | ... | ... | ... | ... | .000 |
| EX1 | .000 | .000 | .000 | ... | ... | ... | ... | ... | .000 |

##### Direct Effects (Group number 1 - Default model)

##### Direct Effects - Lower Bounds (PC) (Group number 1 - Default model)

|  | Perceive\_SchoolClimate | Perseverance | Exercise\_Benefits | Enduring\_Enthusiasm | Unrelenting\_Efforts | Opportunities\_forAutonomy | Teacher\_Support | Classmate\_Support | Exercise\_Behaviour |
| --- | --- | --- | --- | --- | --- | --- | --- | --- | --- |
| Perseverance | .123 | .000 | .000 | .000 | .000 | .000 | .000 | .000 | .000 |
| Exercise\_Benefits | .126 | .000 | .000 | .000 | .000 | .000 | .000 | .000 | .000 |
| Enduring\_Enthusiasm | .000 | .843 | .000 | .000 | .000 | .000 | .000 | .000 | .000 |
| Unrelenting\_Efforts | .000 | 1.000 | .000 | .000 | .000 | .000 | .000 | .000 | .000 |
| Opportunities\_forAutonomy | .968 | .000 | .000 | .000 | .000 | .000 | .000 | .000 | .000 |
| Teacher\_Support | .808 | .000 | .000 | .000 | .000 | .000 | .000 | .000 | .000 |
| Classmate\_Support | 1.000 | .000 | .000 | .000 | .000 | .000 | .000 | .000 | .000 |
| Exercise\_Behaviour | .101 | .139 | .267 | .000 | .000 | .000 | .000 | .000 | .000 |
| EE6 | .000 | .000 | .000 | .893 | .000 | .000 | .000 | .000 | .000 |
| EE5 | .000 | .000 | .000 | .965 | .000 | .000 | .000 | .000 | .000 |
| EE4 | .000 | .000 | .000 | .933 | .000 | .000 | .000 | .000 | .000 |
| EE3 | .000 | .000 | .000 | .976 | .000 | .000 | .000 | .000 | .000 |
| EE2 | .000 | .000 | .000 | .918 | .000 | .000 | .000 | .000 | .000 |
| EE1 | .000 | .000 | .000 | 1.000 | .000 | .000 | .000 | .000 | .000 |
| UE6 | .000 | .000 | .000 | .000 | .989 | .000 | .000 | .000 | .000 |
| UE5 | .000 | .000 | .000 | .000 | .991 | .000 | .000 | .000 | .000 |
| UE4 | .000 | .000 | .000 | .000 | 1.048 | .000 | .000 | .000 | .000 |
| UE3 | .000 | .000 | .000 | .000 | 1.048 | .000 | .000 | .000 | .000 |
| UE2 | .000 | .000 | .000 | .000 | .933 | .000 | .000 | .000 | .000 |
| UE1 | .000 | .000 | .000 | .000 | 1.000 | .000 | .000 | .000 | .000 |
| OA1 | .000 | .000 | .000 | .000 | .000 | .907 | .000 | .000 | .000 |
| OA2 | .000 | .000 | .000 | .000 | .000 | .862 | .000 | .000 | .000 |
| OA3 | .000 | .000 | .000 | .000 | .000 | 1.000 | .000 | .000 | .000 |
| OA4 | .000 | .000 | .000 | .000 | .000 | .867 | .000 | .000 | .000 |
| OA5 | .000 | .000 | .000 | .000 | .000 | 1.178 | .000 | .000 | .000 |
| CS1 | .000 | .000 | .000 | .000 | .000 | .000 | .000 | .939 | .000 |
| CS2 | .000 | .000 | .000 | .000 | .000 | .000 | .000 | .917 | .000 |
| CS3 | .000 | .000 | .000 | .000 | .000 | .000 | .000 | .947 | .000 |
| CS4 | .000 | .000 | .000 | .000 | .000 | .000 | .000 | .949 | .000 |
| CS5 | .000 | .000 | .000 | .000 | .000 | .000 | .000 | .949 | .000 |
| CS6 | .000 | .000 | .000 | .000 | .000 | .000 | .000 | .967 | .000 |
| CS7 | .000 | .000 | .000 | .000 | .000 | .000 | .000 | 1.000 | .000 |
| CS8 | .000 | .000 | .000 | .000 | .000 | .000 | .000 | .941 | .000 |
| CS9 | .000 | .000 | .000 | .000 | .000 | .000 | .000 | 1.018 | .000 |
| CS10 | .000 | .000 | .000 | .000 | .000 | .000 | .000 | .981 | .000 |
| CS11 | .000 | .000 | .000 | .000 | .000 | .000 | .000 | .971 | .000 |
| CS12 | .000 | .000 | .000 | .000 | .000 | .000 | .000 | 1.178 | .000 |
| CS13 | .000 | .000 | .000 | .000 | .000 | .000 | .000 | 1.102 | .000 |
| TS1 | .000 | .000 | .000 | .000 | .000 | .000 | .739 | .000 | .000 |
| TS2 | .000 | .000 | .000 | .000 | .000 | .000 | .842 | .000 | .000 |
| TS3 | .000 | .000 | .000 | .000 | .000 | .000 | .835 | .000 | .000 |
| TS4 | .000 | .000 | .000 | .000 | .000 | .000 | .814 | .000 | .000 |
| TS5 | .000 | .000 | .000 | .000 | .000 | .000 | .836 | .000 | .000 |
| TS6 | .000 | .000 | .000 | .000 | .000 | .000 | .702 | .000 | .000 |
| TS7 | .000 | .000 | .000 | .000 | .000 | .000 | 1.000 | .000 | .000 |
| EB1 | .000 | .000 | .909 | .000 | .000 | .000 | .000 | .000 | .000 |
| EB2 | .000 | .000 | .858 | .000 | .000 | .000 | .000 | .000 | .000 |
| EB3 | .000 | .000 | .766 | .000 | .000 | .000 | .000 | .000 | .000 |
| EB4 | .000 | .000 | .900 | .000 | .000 | .000 | .000 | .000 | .000 |
| EB5 | .000 | .000 | 1.000 | .000 | .000 | .000 | .000 | .000 | .000 |
| EX3 | .000 | .000 | .000 | .000 | .000 | .000 | .000 | .000 | .877 |
| EX2 | .000 | .000 | .000 | .000 | .000 | .000 | .000 | .000 | .932 |
| EX1 | .000 | .000 | .000 | .000 | .000 | .000 | .000 | .000 | 1.000 |

##### Direct Effects - Upper Bounds (PC) (Group number 1 - Default model)

|  | Perceive\_SchoolClimate | Perseverance | Exercise\_Benefits | Enduring\_Enthusiasm | Unrelenting\_Efforts | Opportunities\_forAutonomy | Teacher\_Support | Classmate\_Support | Exercise\_Behaviour |
| --- | --- | --- | --- | --- | --- | --- | --- | --- | --- |
| Perseverance | .603 | .000 | .000 | .000 | .000 | .000 | .000 | .000 | .000 |
| Exercise\_Benefits | .476 | .000 | .000 | .000 | .000 | .000 | .000 | .000 | .000 |
| Enduring\_Enthusiasm | .000 | 1.698 | .000 | .000 | .000 | .000 | .000 | .000 | .000 |
| Unrelenting\_Efforts | .000 | 1.000 | .000 | .000 | .000 | .000 | .000 | .000 | .000 |
| Opportunities\_forAutonomy | 1.585 | .000 | .000 | .000 | .000 | .000 | .000 | .000 | .000 |
| Teacher\_Support | 1.467 | .000 | .000 | .000 | .000 | .000 | .000 | .000 | .000 |
| Classmate\_Support | 1.000 | .000 | .000 | .000 | .000 | .000 | .000 | .000 | .000 |
| Exercise\_Behaviour | .632 | .390 | .836 | .000 | .000 | .000 | .000 | .000 | .000 |
| EE6 | .000 | .000 | .000 | 1.031 | .000 | .000 | .000 | .000 | .000 |
| EE5 | .000 | .000 | .000 | 1.079 | .000 | .000 | .000 | .000 | .000 |
| EE4 | .000 | .000 | .000 | 1.061 | .000 | .000 | .000 | .000 | .000 |
| EE3 | .000 | .000 | .000 | 1.099 | .000 | .000 | .000 | .000 | .000 |
| EE2 | .000 | .000 | .000 | 1.038 | .000 | .000 | .000 | .000 | .000 |
| EE1 | .000 | .000 | .000 | 1.000 | .000 | .000 | .000 | .000 | .000 |
| UE6 | .000 | .000 | .000 | .000 | 1.188 | .000 | .000 | .000 | .000 |
| UE5 | .000 | .000 | .000 | .000 | 1.141 | .000 | .000 | .000 | .000 |
| UE4 | .000 | .000 | .000 | .000 | 1.210 | .000 | .000 | .000 | .000 |
| UE3 | .000 | .000 | .000 | .000 | 1.208 | .000 | .000 | .000 | .000 |
| UE2 | .000 | .000 | .000 | .000 | 1.078 | .000 | .000 | .000 | .000 |
| UE1 | .000 | .000 | .000 | .000 | 1.000 | .000 | .000 | .000 | .000 |
| OA1 | .000 | .000 | .000 | .000 | .000 | 1.118 | .000 | .000 | .000 |
| OA2 | .000 | .000 | .000 | .000 | .000 | 1.046 | .000 | .000 | .000 |
| OA3 | .000 | .000 | .000 | .000 | .000 | 1.000 | .000 | .000 | .000 |
| OA4 | .000 | .000 | .000 | .000 | .000 | 1.084 | .000 | .000 | .000 |
| OA5 | .000 | .000 | .000 | .000 | .000 | 1.477 | .000 | .000 | .000 |
| CS1 | .000 | .000 | .000 | .000 | .000 | .000 | .000 | 1.104 | .000 |
| CS2 | .000 | .000 | .000 | .000 | .000 | .000 | .000 | 1.073 | .000 |
| CS3 | .000 | .000 | .000 | .000 | .000 | .000 | .000 | 1.125 | .000 |
| CS4 | .000 | .000 | .000 | .000 | .000 | .000 | .000 | 1.098 | .000 |
| CS5 | .000 | .000 | .000 | .000 | .000 | .000 | .000 | 1.113 | .000 |
| CS6 | .000 | .000 | .000 | .000 | .000 | .000 | .000 | 1.115 | .000 |
| CS7 | .000 | .000 | .000 | .000 | .000 | .000 | .000 | 1.000 | .000 |
| CS8 | .000 | .000 | .000 | .000 | .000 | .000 | .000 | 1.109 | .000 |
| CS9 | .000 | .000 | .000 | .000 | .000 | .000 | .000 | 1.147 | .000 |
| CS10 | .000 | .000 | .000 | .000 | .000 | .000 | .000 | 1.110 | .000 |
| CS11 | .000 | .000 | .000 | .000 | .000 | .000 | .000 | 1.152 | .000 |
| CS12 | .000 | .000 | .000 | .000 | .000 | .000 | .000 | 1.428 | .000 |
| CS13 | .000 | .000 | .000 | .000 | .000 | .000 | .000 | 1.334 | .000 |
| TS1 | .000 | .000 | .000 | .000 | .000 | .000 | .877 | .000 | .000 |
| TS2 | .000 | .000 | .000 | .000 | .000 | .000 | .962 | .000 | .000 |
| TS3 | .000 | .000 | .000 | .000 | .000 | .000 | .949 | .000 | .000 |
| TS4 | .000 | .000 | .000 | .000 | .000 | .000 | .942 | .000 | .000 |
| TS5 | .000 | .000 | .000 | .000 | .000 | .000 | .959 | .000 | .000 |
| TS6 | .000 | .000 | .000 | .000 | .000 | .000 | .858 | .000 | .000 |
| TS7 | .000 | .000 | .000 | .000 | .000 | .000 | 1.000 | .000 | .000 |
| EB1 | .000 | .000 | 1.463 | .000 | .000 | .000 | .000 | .000 | .000 |
| EB2 | .000 | .000 | 1.380 | .000 | .000 | .000 | .000 | .000 | .000 |
| EB3 | .000 | .000 | 1.239 | .000 | .000 | .000 | .000 | .000 | .000 |
| EB4 | .000 | .000 | 1.393 | .000 | .000 | .000 | .000 | .000 | .000 |
| EB5 | .000 | .000 | 1.000 | .000 | .000 | .000 | .000 | .000 | .000 |
| EX3 | .000 | .000 | .000 | .000 | .000 | .000 | .000 | .000 | 1.254 |
| EX2 | .000 | .000 | .000 | .000 | .000 | .000 | .000 | .000 | 1.318 |
| EX1 | .000 | .000 | .000 | .000 | .000 | .000 | .000 | .000 | 1.000 |

##### Direct Effects - Two Tailed Significance (PC) (Group number 1 - Default model)

|  | Perceive\_SchoolClimate | Perseverance | Exercise\_Benefits | Enduring\_Enthusiasm | Unrelenting\_Efforts | Opportunities\_forAutonomy | Teacher\_Support | Classmate\_Support | Exercise\_Behaviour |
| --- | --- | --- | --- | --- | --- | --- | --- | --- | --- |
| Perseverance | .003 | ... | ... | ... | ... | ... | ... | ... | ... |
| Exercise\_Benefits | .000 | ... | ... | ... | ... | ... | ... | ... | ... |
| Enduring\_Enthusiasm | ... | .000 | ... | ... | ... | ... | ... | ... | ... |
| Unrelenting\_Efforts | ... | ... | ... | ... | ... | ... | ... | ... | ... |
| Opportunities\_forAutonomy | .000 | ... | ... | ... | ... | ... | ... | ... | ... |
| Teacher\_Support | .000 | ... | ... | ... | ... | ... | ... | ... | ... |
| Classmate\_Support | ... | ... | ... | ... | ... | ... | ... | ... | ... |
| Exercise\_Behaviour | .004 | .000 | .000 | ... | ... | ... | ... | ... | ... |
| EE6 | ... | ... | ... | .000 | ... | ... | ... | ... | ... |
| EE5 | ... | ... | ... | .000 | ... | ... | ... | ... | ... |
| EE4 | ... | ... | ... | .000 | ... | ... | ... | ... | ... |
| EE3 | ... | ... | ... | .000 | ... | ... | ... | ... | ... |
| EE2 | ... | ... | ... | .000 | ... | ... | ... | ... | ... |
| EE1 | ... | ... | ... | ... | ... | ... | ... | ... | ... |
| UE6 | ... | ... | ... | ... | .000 | ... | ... | ... | ... |
| UE5 | ... | ... | ... | ... | .000 | ... | ... | ... | ... |
| UE4 | ... | ... | ... | ... | .000 | ... | ... | ... | ... |
| UE3 | ... | ... | ... | ... | .000 | ... | ... | ... | ... |
| UE2 | ... | ... | ... | ... | .000 | ... | ... | ... | ... |
| UE1 | ... | ... | ... | ... | ... | ... | ... | ... | ... |
| OA1 | ... | ... | ... | ... | ... | .000 | ... | ... | ... |
| OA2 | ... | ... | ... | ... | ... | .000 | ... | ... | ... |
| OA3 | ... | ... | ... | ... | ... | ... | ... | ... | ... |
| OA4 | ... | ... | ... | ... | ... | .000 | ... | ... | ... |
| OA5 | ... | ... | ... | ... | ... | .000 | ... | ... | ... |
| CS1 | ... | ... | ... | ... | ... | ... | ... | .000 | ... |
| CS2 | ... | ... | ... | ... | ... | ... | ... | .000 | ... |
| CS3 | ... | ... | ... | ... | ... | ... | ... | .000 | ... |
| CS4 | ... | ... | ... | ... | ... | ... | ... | .000 | ... |
| CS5 | ... | ... | ... | ... | ... | ... | ... | .000 | ... |
| CS6 | ... | ... | ... | ... | ... | ... | ... | .000 | ... |
| CS7 | ... | ... | ... | ... | ... | ... | ... | ... | ... |
| CS8 | ... | ... | ... | ... | ... | ... | ... | .000 | ... |
| CS9 | ... | ... | ... | ... | ... | ... | ... | .000 | ... |
| CS10 | ... | ... | ... | ... | ... | ... | ... | .000 | ... |
| CS11 | ... | ... | ... | ... | ... | ... | ... | .000 | ... |
| CS12 | ... | ... | ... | ... | ... | ... | ... | .000 | ... |
| CS13 | ... | ... | ... | ... | ... | ... | ... | .000 | ... |
| TS1 | ... | ... | ... | ... | ... | ... | .000 | ... | ... |
| TS2 | ... | ... | ... | ... | ... | ... | .000 | ... | ... |
| TS3 | ... | ... | ... | ... | ... | ... | .000 | ... | ... |
| TS4 | ... | ... | ... | ... | ... | ... | .000 | ... | ... |
| TS5 | ... | ... | ... | ... | ... | ... | .000 | ... | ... |
| TS6 | ... | ... | ... | ... | ... | ... | .000 | ... | ... |
| TS7 | ... | ... | ... | ... | ... | ... | ... | ... | ... |
| EB1 | ... | ... | .000 | ... | ... | ... | ... | ... | ... |
| EB2 | ... | ... | .000 | ... | ... | ... | ... | ... | ... |
| EB3 | ... | ... | .000 | ... | ... | ... | ... | ... | ... |
| EB4 | ... | ... | .000 | ... | ... | ... | ... | ... | ... |
| EB5 | ... | ... | ... | ... | ... | ... | ... | ... | ... |
| EX3 | ... | ... | ... | ... | ... | ... | ... | ... | .000 |
| EX2 | ... | ... | ... | ... | ... | ... | ... | ... | .000 |
| EX1 | ... | ... | ... | ... | ... | ... | ... | ... | ... |

##### Standardized Direct Effects (Group number 1 - Default model)

##### Standardized Direct Effects - Lower Bounds (PC) (Group number 1 - Default model)

|  | Perceive\_SchoolClimate | Perseverance | Exercise\_Benefits | Enduring\_Enthusiasm | Unrelenting\_Efforts | Opportunities\_forAutonomy | Teacher\_Support | Classmate\_Support | Exercise\_Behaviour |
| --- | --- | --- | --- | --- | --- | --- | --- | --- | --- |
| Perseverance | .066 | .000 | .000 | .000 | .000 | .000 | .000 | .000 | .000 |
| Exercise\_Benefits | .131 | .000 | .000 | .000 | .000 | .000 | .000 | .000 | .000 |
| Enduring\_Enthusiasm | .000 | .708 | .000 | .000 | .000 | .000 | .000 | .000 | .000 |
| Unrelenting\_Efforts | .000 | .685 | .000 | .000 | .000 | .000 | .000 | .000 | .000 |
| Opportunities\_forAutonomy | .727 | .000 | .000 | .000 | .000 | .000 | .000 | .000 | .000 |
| Teacher\_Support | .423 | .000 | .000 | .000 | .000 | .000 | .000 | .000 | .000 |
| Classmate\_Support | .565 | .000 | .000 | .000 | .000 | .000 | .000 | .000 | .000 |
| Exercise\_Behaviour | .054 | .147 | .149 | .000 | .000 | .000 | .000 | .000 | .000 |
| EE6 | .000 | .000 | .000 | .736 | .000 | .000 | .000 | .000 | .000 |
| EE5 | .000 | .000 | .000 | .830 | .000 | .000 | .000 | .000 | .000 |
| EE4 | .000 | .000 | .000 | .821 | .000 | .000 | .000 | .000 | .000 |
| EE3 | .000 | .000 | .000 | .833 | .000 | .000 | .000 | .000 | .000 |
| EE2 | .000 | .000 | .000 | .815 | .000 | .000 | .000 | .000 | .000 |
| EE1 | .000 | .000 | .000 | .804 | .000 | .000 | .000 | .000 | .000 |
| UE6 | .000 | .000 | .000 | .000 | .676 | .000 | .000 | .000 | .000 |
| UE5 | .000 | .000 | .000 | .000 | .815 | .000 | .000 | .000 | .000 |
| UE4 | .000 | .000 | .000 | .000 | .823 | .000 | .000 | .000 | .000 |
| UE3 | .000 | .000 | .000 | .000 | .823 | .000 | .000 | .000 | .000 |
| UE2 | .000 | .000 | .000 | .000 | .783 | .000 | .000 | .000 | .000 |
| UE1 | .000 | .000 | .000 | .000 | .754 | .000 | .000 | .000 | .000 |
| OA1 | .000 | .000 | .000 | .000 | .000 | .781 | .000 | .000 | .000 |
| OA2 | .000 | .000 | .000 | .000 | .000 | .673 | .000 | .000 | .000 |
| OA3 | .000 | .000 | .000 | .000 | .000 | .707 | .000 | .000 | .000 |
| OA4 | .000 | .000 | .000 | .000 | .000 | .778 | .000 | .000 | .000 |
| OA5 | .000 | .000 | .000 | .000 | .000 | .752 | .000 | .000 | .000 |
| CS1 | .000 | .000 | .000 | .000 | .000 | .000 | .000 | .871 | .000 |
| CS2 | .000 | .000 | .000 | .000 | .000 | .000 | .000 | .849 | .000 |
| CS3 | .000 | .000 | .000 | .000 | .000 | .000 | .000 | .801 | .000 |
| CS4 | .000 | .000 | .000 | .000 | .000 | .000 | .000 | .891 | .000 |
| CS5 | .000 | .000 | .000 | .000 | .000 | .000 | .000 | .865 | .000 |
| CS6 | .000 | .000 | .000 | .000 | .000 | .000 | .000 | .880 | .000 |
| CS7 | .000 | .000 | .000 | .000 | .000 | .000 | .000 | .876 | .000 |
| CS8 | .000 | .000 | .000 | .000 | .000 | .000 | .000 | .870 | .000 |
| CS9 | .000 | .000 | .000 | .000 | .000 | .000 | .000 | .888 | .000 |
| CS10 | .000 | .000 | .000 | .000 | .000 | .000 | .000 | .879 | .000 |
| CS11 | .000 | .000 | .000 | .000 | .000 | .000 | .000 | .885 | .000 |
| CS12 | .000 | .000 | .000 | .000 | .000 | .000 | .000 | .834 | .000 |
| CS13 | .000 | .000 | .000 | .000 | .000 | .000 | .000 | .795 | .000 |
| TS1 | .000 | .000 | .000 | .000 | .000 | .000 | .862 | .000 | .000 |
| TS2 | .000 | .000 | .000 | .000 | .000 | .000 | .911 | .000 | .000 |
| TS3 | .000 | .000 | .000 | .000 | .000 | .000 | .920 | .000 | .000 |
| TS4 | .000 | .000 | .000 | .000 | .000 | .000 | .881 | .000 | .000 |
| TS5 | .000 | .000 | .000 | .000 | .000 | .000 | .889 | .000 | .000 |
| TS6 | .000 | .000 | .000 | .000 | .000 | .000 | .817 | .000 | .000 |
| TS7 | .000 | .000 | .000 | .000 | .000 | .000 | .814 | .000 | .000 |
| EB1 | .000 | .000 | .500 | .000 | .000 | .000 | .000 | .000 | .000 |
| EB2 | .000 | .000 | .493 | .000 | .000 | .000 | .000 | .000 | .000 |
| EB3 | .000 | .000 | .424 | .000 | .000 | .000 | .000 | .000 | .000 |
| EB4 | .000 | .000 | .500 | .000 | .000 | .000 | .000 | .000 | .000 |
| EB5 | .000 | .000 | .406 | .000 | .000 | .000 | .000 | .000 | .000 |
| EX3 | .000 | .000 | .000 | .000 | .000 | .000 | .000 | .000 | .564 |
| EX2 | .000 | .000 | .000 | .000 | .000 | .000 | .000 | .000 | .641 |
| EX1 | .000 | .000 | .000 | .000 | .000 | .000 | .000 | .000 | .568 |

##### Standardized Direct Effects - Upper Bounds (PC) (Group number 1 - Default model)

|  | Perceive\_SchoolClimate | Perseverance | Exercise\_Benefits | Enduring\_Enthusiasm | Unrelenting\_Efforts | Opportunities\_forAutonomy | Teacher\_Support | Classmate\_Support | Exercise\_Behaviour |
| --- | --- | --- | --- | --- | --- | --- | --- | --- | --- |
| Perseverance | .270 | .000 | .000 | .000 | .000 | .000 | .000 | .000 | .000 |
| Exercise\_Benefits | .403 | .000 | .000 | .000 | .000 | .000 | .000 | .000 | .000 |
| Enduring\_Enthusiasm | .000 | 1.002 | .000 | .000 | .000 | .000 | .000 | .000 | .000 |
| Unrelenting\_Efforts | .000 | .967 | .000 | .000 | .000 | .000 | .000 | .000 | .000 |
| Opportunities\_forAutonomy | .950 | .000 | .000 | .000 | .000 | .000 | .000 | .000 | .000 |
| Teacher\_Support | .670 | .000 | .000 | .000 | .000 | .000 | .000 | .000 | .000 |
| Classmate\_Support | .773 | .000 | .000 | .000 | .000 | .000 | .000 | .000 | .000 |
| Exercise\_Behaviour | .295 | .375 | .399 | .000 | .000 | .000 | .000 | .000 | .000 |
| EE6 | .000 | .000 | .000 | .822 | .000 | .000 | .000 | .000 | .000 |
| EE5 | .000 | .000 | .000 | .886 | .000 | .000 | .000 | .000 | .000 |
| EE4 | .000 | .000 | .000 | .882 | .000 | .000 | .000 | .000 | .000 |
| EE3 | .000 | .000 | .000 | .889 | .000 | .000 | .000 | .000 | .000 |
| EE2 | .000 | .000 | .000 | .875 | .000 | .000 | .000 | .000 | .000 |
| EE1 | .000 | .000 | .000 | .863 | .000 | .000 | .000 | .000 | .000 |
| UE6 | .000 | .000 | .000 | .000 | .798 | .000 | .000 | .000 | .000 |
| UE5 | .000 | .000 | .000 | .000 | .873 | .000 | .000 | .000 | .000 |
| UE4 | .000 | .000 | .000 | .000 | .883 | .000 | .000 | .000 | .000 |
| UE3 | .000 | .000 | .000 | .000 | .884 | .000 | .000 | .000 | .000 |
| UE2 | .000 | .000 | .000 | .000 | .850 | .000 | .000 | .000 | .000 |
| UE1 | .000 | .000 | .000 | .000 | .830 | .000 | .000 | .000 | .000 |
| OA1 | .000 | .000 | .000 | .000 | .000 | .881 | .000 | .000 | .000 |
| OA2 | .000 | .000 | .000 | .000 | .000 | .802 | .000 | .000 | .000 |
| OA3 | .000 | .000 | .000 | .000 | .000 | .827 | .000 | .000 | .000 |
| OA4 | .000 | .000 | .000 | .000 | .000 | .891 | .000 | .000 | .000 |
| OA5 | .000 | .000 | .000 | .000 | .000 | .833 | .000 | .000 | .000 |
| CS1 | .000 | .000 | .000 | .000 | .000 | .000 | .000 | .933 | .000 |
| CS2 | .000 | .000 | .000 | .000 | .000 | .000 | .000 | .919 | .000 |
| CS3 | .000 | .000 | .000 | .000 | .000 | .000 | .000 | .899 | .000 |
| CS4 | .000 | .000 | .000 | .000 | .000 | .000 | .000 | .949 | .000 |
| CS5 | .000 | .000 | .000 | .000 | .000 | .000 | .000 | .930 | .000 |
| CS6 | .000 | .000 | .000 | .000 | .000 | .000 | .000 | .938 | .000 |
| CS7 | .000 | .000 | .000 | .000 | .000 | .000 | .000 | .939 | .000 |
| CS8 | .000 | .000 | .000 | .000 | .000 | .000 | .000 | .933 | .000 |
| CS9 | .000 | .000 | .000 | .000 | .000 | .000 | .000 | .943 | .000 |
| CS10 | .000 | .000 | .000 | .000 | .000 | .000 | .000 | .938 | .000 |
| CS11 | .000 | .000 | .000 | .000 | .000 | .000 | .000 | .942 | .000 |
| CS12 | .000 | .000 | .000 | .000 | .000 | .000 | .000 | .886 | .000 |
| CS13 | .000 | .000 | .000 | .000 | .000 | .000 | .000 | .870 | .000 |
| TS1 | .000 | .000 | .000 | .000 | .000 | .000 | .930 | .000 | .000 |
| TS2 | .000 | .000 | .000 | .000 | .000 | .000 | .959 | .000 | .000 |
| TS3 | .000 | .000 | .000 | .000 | .000 | .000 | .968 | .000 | .000 |
| TS4 | .000 | .000 | .000 | .000 | .000 | .000 | .943 | .000 | .000 |
| TS5 | .000 | .000 | .000 | .000 | .000 | .000 | .946 | .000 | .000 |
| TS6 | .000 | .000 | .000 | .000 | .000 | .000 | .899 | .000 | .000 |
| TS7 | .000 | .000 | .000 | .000 | .000 | .000 | .886 | .000 | .000 |
| EB1 | .000 | .000 | .691 | .000 | .000 | .000 | .000 | .000 | .000 |
| EB2 | .000 | .000 | .685 | .000 | .000 | .000 | .000 | .000 | .000 |
| EB3 | .000 | .000 | .625 | .000 | .000 | .000 | .000 | .000 | .000 |
| EB4 | .000 | .000 | .682 | .000 | .000 | .000 | .000 | .000 | .000 |
| EB5 | .000 | .000 | .591 | .000 | .000 | .000 | .000 | .000 | .000 |
| EX3 | .000 | .000 | .000 | .000 | .000 | .000 | .000 | .000 | .716 |
| EX2 | .000 | .000 | .000 | .000 | .000 | .000 | .000 | .000 | .805 |
| EX1 | .000 | .000 | .000 | .000 | .000 | .000 | .000 | .000 | .719 |

##### Standardized Direct Effects - Two Tailed Significance (PC) (Group number 1 - Default model)

|  | Perceive\_SchoolClimate | Perseverance | Exercise\_Benefits | Enduring\_Enthusiasm | Unrelenting\_Efforts | Opportunities\_forAutonomy | Teacher\_Support | Classmate\_Support | Exercise\_Behaviour |
| --- | --- | --- | --- | --- | --- | --- | --- | --- | --- |
| Perseverance | .003 | ... | ... | ... | ... | ... | ... | ... | ... |
| Exercise\_Benefits | .000 | ... | ... | ... | ... | ... | ... | ... | ... |
| Enduring\_Enthusiasm | ... | .000 | ... | ... | ... | ... | ... | ... | ... |
| Unrelenting\_Efforts | ... | .000 | ... | ... | ... | ... | ... | ... | ... |
| Opportunities\_forAutonomy | .000 | ... | ... | ... | ... | ... | ... | ... | ... |
| Teacher\_Support | .000 | ... | ... | ... | ... | ... | ... | ... | ... |
| Classmate\_Support | .000 | ... | ... | ... | ... | ... | ... | ... | ... |
| Exercise\_Behaviour | .004 | .000 | .000 | ... | ... | ... | ... | ... | ... |
| EE6 | ... | ... | ... | .000 | ... | ... | ... | ... | ... |
| EE5 | ... | ... | ... | .000 | ... | ... | ... | ... | ... |
| EE4 | ... | ... | ... | .000 | ... | ... | ... | ... | ... |
| EE3 | ... | ... | ... | .000 | ... | ... | ... | ... | ... |
| EE2 | ... | ... | ... | .000 | ... | ... | ... | ... | ... |
| EE1 | ... | ... | ... | .000 | ... | ... | ... | ... | ... |
| UE6 | ... | ... | ... | ... | .000 | ... | ... | ... | ... |
| UE5 | ... | ... | ... | ... | .000 | ... | ... | ... | ... |
| UE4 | ... | ... | ... | ... | .000 | ... | ... | ... | ... |
| UE3 | ... | ... | ... | ... | .000 | ... | ... | ... | ... |
| UE2 | ... | ... | ... | ... | .000 | ... | ... | ... | ... |
| UE1 | ... | ... | ... | ... | .000 | ... | ... | ... | ... |
| OA1 | ... | ... | ... | ... | ... | .000 | ... | ... | ... |
| OA2 | ... | ... | ... | ... | ... | .000 | ... | ... | ... |
| OA3 | ... | ... | ... | ... | ... | .000 | ... | ... | ... |
| OA4 | ... | ... | ... | ... | ... | .000 | ... | ... | ... |
| OA5 | ... | ... | ... | ... | ... | .000 | ... | ... | ... |
| CS1 | ... | ... | ... | ... | ... | ... | ... | .000 | ... |
| CS2 | ... | ... | ... | ... | ... | ... | ... | .000 | ... |
| CS3 | ... | ... | ... | ... | ... | ... | ... | .000 | ... |
| CS4 | ... | ... | ... | ... | ... | ... | ... | .000 | ... |
| CS5 | ... | ... | ... | ... | ... | ... | ... | .000 | ... |
| CS6 | ... | ... | ... | ... | ... | ... | ... | .000 | ... |
| CS7 | ... | ... | ... | ... | ... | ... | ... | .000 | ... |
| CS8 | ... | ... | ... | ... | ... | ... | ... | .000 | ... |
| CS9 | ... | ... | ... | ... | ... | ... | ... | .000 | ... |
| CS10 | ... | ... | ... | ... | ... | ... | ... | .000 | ... |
| CS11 | ... | ... | ... | ... | ... | ... | ... | .000 | ... |
| CS12 | ... | ... | ... | ... | ... | ... | ... | .000 | ... |
| CS13 | ... | ... | ... | ... | ... | ... | ... | .000 | ... |
| TS1 | ... | ... | ... | ... | ... | ... | .000 | ... | ... |
| TS2 | ... | ... | ... | ... | ... | ... | .000 | ... | ... |
| TS3 | ... | ... | ... | ... | ... | ... | .000 | ... | ... |
| TS4 | ... | ... | ... | ... | ... | ... | .000 | ... | ... |
| TS5 | ... | ... | ... | ... | ... | ... | .000 | ... | ... |
| TS6 | ... | ... | ... | ... | ... | ... | .000 | ... | ... |
| TS7 | ... | ... | ... | ... | ... | ... | .000 | ... | ... |
| EB1 | ... | ... | .000 | ... | ... | ... | ... | ... | ... |
| EB2 | ... | ... | .000 | ... | ... | ... | ... | ... | ... |
| EB3 | ... | ... | .000 | ... | ... | ... | ... | ... | ... |
| EB4 | ... | ... | .000 | ... | ... | ... | ... | ... | ... |
| EB5 | ... | ... | .000 | ... | ... | ... | ... | ... | ... |
| EX3 | ... | ... | ... | ... | ... | ... | ... | ... | .000 |
| EX2 | ... | ... | ... | ... | ... | ... | ... | ... | .000 |
| EX1 | ... | ... | ... | ... | ... | ... | ... | ... | .000 |

##### Indirect Effects (Group number 1 - Default model)

##### Indirect Effects - Lower Bounds (PC) (Group number 1 - Default model)

|  | Perceive\_SchoolClimate | Perseverance | Exercise\_Benefits | Enduring\_Enthusiasm | Unrelenting\_Efforts | Opportunities\_forAutonomy | Teacher\_Support | Classmate\_Support | Exercise\_Behaviour |
| --- | --- | --- | --- | --- | --- | --- | --- | --- | --- |
| Perseverance | .000 | .000 | .000 | .000 | .000 | .000 | .000 | .000 | .000 |
| Exercise\_Benefits | .000 | .000 | .000 | .000 | .000 | .000 | .000 | .000 | .000 |
| Enduring\_Enthusiasm | .144 | .000 | .000 | .000 | .000 | .000 | .000 | .000 | .000 |
| Unrelenting\_Efforts | .123 | .000 | .000 | .000 | .000 | .000 | .000 | .000 | .000 |
| Opportunities\_forAutonomy | .000 | .000 | .000 | .000 | .000 | .000 | .000 | .000 | .000 |
| Teacher\_Support | .000 | .000 | .000 | .000 | .000 | .000 | .000 | .000 | .000 |
| Classmate\_Support | .000 | .000 | .000 | .000 | .000 | .000 | .000 | .000 | .000 |
| Exercise\_Behaviour | .115 | .000 | .000 | .000 | .000 | .000 | .000 | .000 | .000 |
| EE6 | .137 | .805 | .000 | .000 | .000 | .000 | .000 | .000 | .000 |
| EE5 | .144 | .859 | .000 | .000 | .000 | .000 | .000 | .000 | .000 |
| EE4 | .144 | .835 | .000 | .000 | .000 | .000 | .000 | .000 | .000 |
| EE3 | .150 | .874 | .000 | .000 | .000 | .000 | .000 | .000 | .000 |
| EE2 | .139 | .828 | .000 | .000 | .000 | .000 | .000 | .000 | .000 |
| EE1 | .144 | .843 | .000 | .000 | .000 | .000 | .000 | .000 | .000 |
| UE6 | .136 | .989 | .000 | .000 | .000 | .000 | .000 | .000 | .000 |
| UE5 | .129 | .991 | .000 | .000 | .000 | .000 | .000 | .000 | .000 |
| UE4 | .139 | 1.048 | .000 | .000 | .000 | .000 | .000 | .000 | .000 |
| UE3 | .138 | 1.048 | .000 | .000 | .000 | .000 | .000 | .000 | .000 |
| UE2 | .123 | .933 | .000 | .000 | .000 | .000 | .000 | .000 | .000 |
| UE1 | .123 | 1.000 | .000 | .000 | .000 | .000 | .000 | .000 | .000 |
| OA1 | .972 | .000 | .000 | .000 | .000 | .000 | .000 | .000 | .000 |
| OA2 | .916 | .000 | .000 | .000 | .000 | .000 | .000 | .000 | .000 |
| OA3 | .968 | .000 | .000 | .000 | .000 | .000 | .000 | .000 | .000 |
| OA4 | .941 | .000 | .000 | .000 | .000 | .000 | .000 | .000 | .000 |
| OA5 | 1.278 | .000 | .000 | .000 | .000 | .000 | .000 | .000 | .000 |
| CS1 | .939 | .000 | .000 | .000 | .000 | .000 | .000 | .000 | .000 |
| CS2 | .917 | .000 | .000 | .000 | .000 | .000 | .000 | .000 | .000 |
| CS3 | .947 | .000 | .000 | .000 | .000 | .000 | .000 | .000 | .000 |
| CS4 | .949 | .000 | .000 | .000 | .000 | .000 | .000 | .000 | .000 |
| CS5 | .949 | .000 | .000 | .000 | .000 | .000 | .000 | .000 | .000 |
| CS6 | .967 | .000 | .000 | .000 | .000 | .000 | .000 | .000 | .000 |
| CS7 | 1.000 | .000 | .000 | .000 | .000 | .000 | .000 | .000 | .000 |
| CS8 | .941 | .000 | .000 | .000 | .000 | .000 | .000 | .000 | .000 |
| CS9 | 1.018 | .000 | .000 | .000 | .000 | .000 | .000 | .000 | .000 |
| CS10 | .981 | .000 | .000 | .000 | .000 | .000 | .000 | .000 | .000 |
| CS11 | .971 | .000 | .000 | .000 | .000 | .000 | .000 | .000 | .000 |
| CS12 | 1.178 | .000 | .000 | .000 | .000 | .000 | .000 | .000 | .000 |
| CS13 | 1.102 | .000 | .000 | .000 | .000 | .000 | .000 | .000 | .000 |
| TS1 | .652 | .000 | .000 | .000 | .000 | .000 | .000 | .000 | .000 |
| TS2 | .728 | .000 | .000 | .000 | .000 | .000 | .000 | .000 | .000 |
| TS3 | .726 | .000 | .000 | .000 | .000 | .000 | .000 | .000 | .000 |
| TS4 | .713 | .000 | .000 | .000 | .000 | .000 | .000 | .000 | .000 |
| TS5 | .724 | .000 | .000 | .000 | .000 | .000 | .000 | .000 | .000 |
| TS6 | .629 | .000 | .000 | .000 | .000 | .000 | .000 | .000 | .000 |
| TS7 | .808 | .000 | .000 | .000 | .000 | .000 | .000 | .000 | .000 |
| EB1 | .143 | .000 | .000 | .000 | .000 | .000 | .000 | .000 | .000 |
| EB2 | .141 | .000 | .000 | .000 | .000 | .000 | .000 | .000 | .000 |
| EB3 | .123 | .000 | .000 | .000 | .000 | .000 | .000 | .000 | .000 |
| EB4 | .143 | .000 | .000 | .000 | .000 | .000 | .000 | .000 | .000 |
| EB5 | .126 | .000 | .000 | .000 | .000 | .000 | .000 | .000 | .000 |
| EX3 | .333 | .147 | .279 | .000 | .000 | .000 | .000 | .000 | .000 |
| EX2 | .364 | .154 | .305 | .000 | .000 | .000 | .000 | .000 | .000 |
| EX1 | .311 | .139 | .267 | .000 | .000 | .000 | .000 | .000 | .000 |

##### Indirect Effects - Upper Bounds (PC) (Group number 1 - Default model)

|  | Perceive\_SchoolClimate | Perseverance | Exercise\_Benefits | Enduring\_Enthusiasm | Unrelenting\_Efforts | Opportunities\_forAutonomy | Teacher\_Support | Classmate\_Support | Exercise\_Behaviour |
| --- | --- | --- | --- | --- | --- | --- | --- | --- | --- |
| Perseverance | .000 | .000 | .000 | .000 | .000 | .000 | .000 | .000 | .000 |
| Exercise\_Benefits | .000 | .000 | .000 | .000 | .000 | .000 | .000 | .000 | .000 |
| Enduring\_Enthusiasm | .710 | .000 | .000 | .000 | .000 | .000 | .000 | .000 | .000 |
| Unrelenting\_Efforts | .603 | .000 | .000 | .000 | .000 | .000 | .000 | .000 | .000 |
| Opportunities\_forAutonomy | .000 | .000 | .000 | .000 | .000 | .000 | .000 | .000 | .000 |
| Teacher\_Support | .000 | .000 | .000 | .000 | .000 | .000 | .000 | .000 | .000 |
| Classmate\_Support | .000 | .000 | .000 | .000 | .000 | .000 | .000 | .000 | .000 |
| Exercise\_Behaviour | .391 | .000 | .000 | .000 | .000 | .000 | .000 | .000 | .000 |
| EE6 | .688 | 1.621 | .000 | .000 | .000 | .000 | .000 | .000 | .000 |
| EE5 | .734 | 1.723 | .000 | .000 | .000 | .000 | .000 | .000 | .000 |
| EE4 | .715 | 1.676 | .000 | .000 | .000 | .000 | .000 | .000 | .000 |
| EE3 | .739 | 1.750 | .000 | .000 | .000 | .000 | .000 | .000 | .000 |
| EE2 | .698 | 1.650 | .000 | .000 | .000 | .000 | .000 | .000 | .000 |
| EE1 | .710 | 1.698 | .000 | .000 | .000 | .000 | .000 | .000 | .000 |
| UE6 | .658 | 1.188 | .000 | .000 | .000 | .000 | .000 | .000 | .000 |
| UE5 | .637 | 1.141 | .000 | .000 | .000 | .000 | .000 | .000 | .000 |
| UE4 | .678 | 1.210 | .000 | .000 | .000 | .000 | .000 | .000 | .000 |
| UE3 | .678 | 1.208 | .000 | .000 | .000 | .000 | .000 | .000 | .000 |
| UE2 | .602 | 1.078 | .000 | .000 | .000 | .000 | .000 | .000 | .000 |
| UE1 | .603 | 1.000 | .000 | .000 | .000 | .000 | .000 | .000 | .000 |
| OA1 | 1.618 | .000 | .000 | .000 | .000 | .000 | .000 | .000 | .000 |
| OA2 | 1.521 | .000 | .000 | .000 | .000 | .000 | .000 | .000 | .000 |
| OA3 | 1.585 | .000 | .000 | .000 | .000 | .000 | .000 | .000 | .000 |
| OA4 | 1.550 | .000 | .000 | .000 | .000 | .000 | .000 | .000 | .000 |
| OA5 | 2.079 | .000 | .000 | .000 | .000 | .000 | .000 | .000 | .000 |
| CS1 | 1.104 | .000 | .000 | .000 | .000 | .000 | .000 | .000 | .000 |
| CS2 | 1.073 | .000 | .000 | .000 | .000 | .000 | .000 | .000 | .000 |
| CS3 | 1.125 | .000 | .000 | .000 | .000 | .000 | .000 | .000 | .000 |
| CS4 | 1.098 | .000 | .000 | .000 | .000 | .000 | .000 | .000 | .000 |
| CS5 | 1.113 | .000 | .000 | .000 | .000 | .000 | .000 | .000 | .000 |
| CS6 | 1.115 | .000 | .000 | .000 | .000 | .000 | .000 | .000 | .000 |
| CS7 | 1.000 | .000 | .000 | .000 | .000 | .000 | .000 | .000 | .000 |
| CS8 | 1.109 | .000 | .000 | .000 | .000 | .000 | .000 | .000 | .000 |
| CS9 | 1.147 | .000 | .000 | .000 | .000 | .000 | .000 | .000 | .000 |
| CS10 | 1.110 | .000 | .000 | .000 | .000 | .000 | .000 | .000 | .000 |
| CS11 | 1.152 | .000 | .000 | .000 | .000 | .000 | .000 | .000 | .000 |
| CS12 | 1.428 | .000 | .000 | .000 | .000 | .000 | .000 | .000 | .000 |
| CS13 | 1.334 | .000 | .000 | .000 | .000 | .000 | .000 | .000 | .000 |
| TS1 | 1.205 | .000 | .000 | .000 | .000 | .000 | .000 | .000 | .000 |
| TS2 | 1.333 | .000 | .000 | .000 | .000 | .000 | .000 | .000 | .000 |
| TS3 | 1.324 | .000 | .000 | .000 | .000 | .000 | .000 | .000 | .000 |
| TS4 | 1.299 | .000 | .000 | .000 | .000 | .000 | .000 | .000 | .000 |
| TS5 | 1.332 | .000 | .000 | .000 | .000 | .000 | .000 | .000 | .000 |
| TS6 | 1.168 | .000 | .000 | .000 | .000 | .000 | .000 | .000 | .000 |
| TS7 | 1.467 | .000 | .000 | .000 | .000 | .000 | .000 | .000 | .000 |
| EB1 | .527 | .000 | .000 | .000 | .000 | .000 | .000 | .000 | .000 |
| EB2 | .496 | .000 | .000 | .000 | .000 | .000 | .000 | .000 | .000 |
| EB3 | .468 | .000 | .000 | .000 | .000 | .000 | .000 | .000 | .000 |
| EB4 | .507 | .000 | .000 | .000 | .000 | .000 | .000 | .000 | .000 |
| EB5 | .476 | .000 | .000 | .000 | .000 | .000 | .000 | .000 | .000 |
| EX3 | .924 | .405 | .895 | .000 | .000 | .000 | .000 | .000 | .000 |
| EX2 | .970 | .437 | .907 | .000 | .000 | .000 | .000 | .000 | .000 |
| EX1 | .896 | .390 | .836 | .000 | .000 | .000 | .000 | .000 | .000 |

##### Indirect Effects - Two Tailed Significance (PC) (Group number 1 - Default model)

|  | Perceive\_SchoolClimate | Perseverance | Exercise\_Benefits | Enduring\_Enthusiasm | Unrelenting\_Efforts | Opportunities\_forAutonomy | Teacher\_Support | Classmate\_Support | Exercise\_Behaviour |
| --- | --- | --- | --- | --- | --- | --- | --- | --- | --- |
| Perseverance | ... | ... | ... | ... | ... | ... | ... | ... | ... |
| Exercise\_Benefits | ... | ... | ... | ... | ... | ... | ... | ... | ... |
| Enduring\_Enthusiasm | .003 | ... | ... | ... | ... | ... | ... | ... | ... |
| Unrelenting\_Efforts | .003 | ... | ... | ... | ... | ... | ... | ... | ... |
| Opportunities\_forAutonomy | ... | ... | ... | ... | ... | ... | ... | ... | ... |
| Teacher\_Support | ... | ... | ... | ... | ... | ... | ... | ... | ... |
| Classmate\_Support | ... | ... | ... | ... | ... | ... | ... | ... | ... |
| Exercise\_Behaviour | .000 | ... | ... | ... | ... | ... | ... | ... | ... |
| EE6 | .003 | .000 | ... | ... | ... | ... | ... | ... | ... |
| EE5 | .003 | .000 | ... | ... | ... | ... | ... | ... | ... |
| EE4 | .003 | .000 | ... | ... | ... | ... | ... | ... | ... |
| EE3 | .003 | .000 | ... | ... | ... | ... | ... | ... | ... |
| EE2 | .003 | .000 | ... | ... | ... | ... | ... | ... | ... |
| EE1 | .003 | .000 | ... | ... | ... | ... | ... | ... | ... |
| UE6 | .003 | .000 | ... | ... | ... | ... | ... | ... | ... |
| UE5 | .003 | .000 | ... | ... | ... | ... | ... | ... | ... |
| UE4 | .003 | .000 | ... | ... | ... | ... | ... | ... | ... |
| UE3 | .003 | .000 | ... | ... | ... | ... | ... | ... | ... |
| UE2 | .003 | .000 | ... | ... | ... | ... | ... | ... | ... |
| UE1 | .003 | ... | ... | ... | ... | ... | ... | ... | ... |
| OA1 | .000 | ... | ... | ... | ... | ... | ... | ... | ... |
| OA2 | .000 | ... | ... | ... | ... | ... | ... | ... | ... |
| OA3 | .000 | ... | ... | ... | ... | ... | ... | ... | ... |
| OA4 | .000 | ... | ... | ... | ... | ... | ... | ... | ... |
| OA5 | .000 | ... | ... | ... | ... | ... | ... | ... | ... |
| CS1 | .000 | ... | ... | ... | ... | ... | ... | ... | ... |
| CS2 | .000 | ... | ... | ... | ... | ... | ... | ... | ... |
| CS3 | .000 | ... | ... | ... | ... | ... | ... | ... | ... |
| CS4 | .000 | ... | ... | ... | ... | ... | ... | ... | ... |
| CS5 | .000 | ... | ... | ... | ... | ... | ... | ... | ... |
| CS6 | .000 | ... | ... | ... | ... | ... | ... | ... | ... |
| CS7 | ... | ... | ... | ... | ... | ... | ... | ... | ... |
| CS8 | .000 | ... | ... | ... | ... | ... | ... | ... | ... |
| CS9 | .000 | ... | ... | ... | ... | ... | ... | ... | ... |
| CS10 | .000 | ... | ... | ... | ... | ... | ... | ... | ... |
| CS11 | .000 | ... | ... | ... | ... | ... | ... | ... | ... |
| CS12 | .000 | ... | ... | ... | ... | ... | ... | ... | ... |
| CS13 | .000 | ... | ... | ... | ... | ... | ... | ... | ... |
| TS1 | .000 | ... | ... | ... | ... | ... | ... | ... | ... |
| TS2 | .000 | ... | ... | ... | ... | ... | ... | ... | ... |
| TS3 | .000 | ... | ... | ... | ... | ... | ... | ... | ... |
| TS4 | .000 | ... | ... | ... | ... | ... | ... | ... | ... |
| TS5 | .000 | ... | ... | ... | ... | ... | ... | ... | ... |
| TS6 | .000 | ... | ... | ... | ... | ... | ... | ... | ... |
| TS7 | .000 | ... | ... | ... | ... | ... | ... | ... | ... |
| EB1 | .000 | ... | ... | ... | ... | ... | ... | ... | ... |
| EB2 | .000 | ... | ... | ... | ... | ... | ... | ... | ... |
| EB3 | .000 | ... | ... | ... | ... | ... | ... | ... | ... |
| EB4 | .000 | ... | ... | ... | ... | ... | ... | ... | ... |
| EB5 | .000 | ... | ... | ... | ... | ... | ... | ... | ... |
| EX3 | .000 | .000 | .000 | ... | ... | ... | ... | ... | ... |
| EX2 | .000 | .000 | .000 | ... | ... | ... | ... | ... | ... |
| EX1 | .000 | .000 | .000 | ... | ... | ... | ... | ... | ... |

##### Standardized Indirect Effects (Group number 1 - Default model)

##### Standardized Indirect Effects - Lower Bounds (PC) (Group number 1 - Default model)

|  | Perceive\_SchoolClimate | Perseverance | Exercise\_Benefits | Enduring\_Enthusiasm | Unrelenting\_Efforts | Opportunities\_forAutonomy | Teacher\_Support | Classmate\_Support | Exercise\_Behaviour |
| --- | --- | --- | --- | --- | --- | --- | --- | --- | --- |
| Perseverance | .000 | .000 | .000 | .000 | .000 | .000 | .000 | .000 | .000 |
| Exercise\_Benefits | .000 | .000 | .000 | .000 | .000 | .000 | .000 | .000 | .000 |
| Enduring\_Enthusiasm | .054 | .000 | .000 | .000 | .000 | .000 | .000 | .000 | .000 |
| Unrelenting\_Efforts | .054 | .000 | .000 | .000 | .000 | .000 | .000 | .000 | .000 |
| Opportunities\_forAutonomy | .000 | .000 | .000 | .000 | .000 | .000 | .000 | .000 | .000 |
| Teacher\_Support | .000 | .000 | .000 | .000 | .000 | .000 | .000 | .000 | .000 |
| Classmate\_Support | .000 | .000 | .000 | .000 | .000 | .000 | .000 | .000 | .000 |
| Exercise\_Behaviour | .064 | .000 | .000 | .000 | .000 | .000 | .000 | .000 | .000 |
| EE6 | .043 | .545 | .000 | .000 | .000 | .000 | .000 | .000 | .000 |
| EE5 | .047 | .604 | .000 | .000 | .000 | .000 | .000 | .000 | .000 |
| EE4 | .047 | .602 | .000 | .000 | .000 | .000 | .000 | .000 | .000 |
| EE3 | .047 | .608 | .000 | .000 | .000 | .000 | .000 | .000 | .000 |
| EE2 | .046 | .597 | .000 | .000 | .000 | .000 | .000 | .000 | .000 |
| EE1 | .045 | .587 | .000 | .000 | .000 | .000 | .000 | .000 | .000 |
| UE6 | .040 | .497 | .000 | .000 | .000 | .000 | .000 | .000 | .000 |
| UE5 | .045 | .573 | .000 | .000 | .000 | .000 | .000 | .000 | .000 |
| UE4 | .046 | .580 | .000 | .000 | .000 | .000 | .000 | .000 | .000 |
| UE3 | .045 | .580 | .000 | .000 | .000 | .000 | .000 | .000 | .000 |
| UE2 | .044 | .555 | .000 | .000 | .000 | .000 | .000 | .000 | .000 |
| UE1 | .043 | .536 | .000 | .000 | .000 | .000 | .000 | .000 | .000 |
| OA1 | .601 | .000 | .000 | .000 | .000 | .000 | .000 | .000 | .000 |
| OA2 | .521 | .000 | .000 | .000 | .000 | .000 | .000 | .000 | .000 |
| OA3 | .544 | .000 | .000 | .000 | .000 | .000 | .000 | .000 | .000 |
| OA4 | .605 | .000 | .000 | .000 | .000 | .000 | .000 | .000 | .000 |
| OA5 | .574 | .000 | .000 | .000 | .000 | .000 | .000 | .000 | .000 |
| CS1 | .508 | .000 | .000 | .000 | .000 | .000 | .000 | .000 | .000 |
| CS2 | .498 | .000 | .000 | .000 | .000 | .000 | .000 | .000 | .000 |
| CS3 | .476 | .000 | .000 | .000 | .000 | .000 | .000 | .000 | .000 |
| CS4 | .518 | .000 | .000 | .000 | .000 | .000 | .000 | .000 | .000 |
| CS5 | .507 | .000 | .000 | .000 | .000 | .000 | .000 | .000 | .000 |
| CS6 | .514 | .000 | .000 | .000 | .000 | .000 | .000 | .000 | .000 |
| CS7 | .511 | .000 | .000 | .000 | .000 | .000 | .000 | .000 | .000 |
| CS8 | .509 | .000 | .000 | .000 | .000 | .000 | .000 | .000 | .000 |
| CS9 | .517 | .000 | .000 | .000 | .000 | .000 | .000 | .000 | .000 |
| CS10 | .514 | .000 | .000 | .000 | .000 | .000 | .000 | .000 | .000 |
| CS11 | .515 | .000 | .000 | .000 | .000 | .000 | .000 | .000 | .000 |
| CS12 | .483 | .000 | .000 | .000 | .000 | .000 | .000 | .000 | .000 |
| CS13 | .468 | .000 | .000 | .000 | .000 | .000 | .000 | .000 | .000 |
| TS1 | .382 | .000 | .000 | .000 | .000 | .000 | .000 | .000 | .000 |
| TS2 | .398 | .000 | .000 | .000 | .000 | .000 | .000 | .000 | .000 |
| TS3 | .400 | .000 | .000 | .000 | .000 | .000 | .000 | .000 | .000 |
| TS4 | .385 | .000 | .000 | .000 | .000 | .000 | .000 | .000 | .000 |
| TS5 | .389 | .000 | .000 | .000 | .000 | .000 | .000 | .000 | .000 |
| TS6 | .364 | .000 | .000 | .000 | .000 | .000 | .000 | .000 | .000 |
| TS7 | .358 | .000 | .000 | .000 | .000 | .000 | .000 | .000 | .000 |
| EB1 | .076 | .000 | .000 | .000 | .000 | .000 | .000 | .000 | .000 |
| EB2 | .079 | .000 | .000 | .000 | .000 | .000 | .000 | .000 | .000 |
| EB3 | .067 | .000 | .000 | .000 | .000 | .000 | .000 | .000 | .000 |
| EB4 | .077 | .000 | .000 | .000 | .000 | .000 | .000 | .000 | .000 |
| EB5 | .064 | .000 | .000 | .000 | .000 | .000 | .000 | .000 | .000 |
| EX3 | .109 | .094 | .094 | .000 | .000 | .000 | .000 | .000 | .000 |
| EX2 | .124 | .104 | .110 | .000 | .000 | .000 | .000 | .000 | .000 |
| EX1 | .107 | .092 | .096 | .000 | .000 | .000 | .000 | .000 | .000 |

##### Standardized Indirect Effects - Upper Bounds (PC) (Group number 1 - Default model)

|  | Perceive\_SchoolClimate | Perseverance | Exercise\_Benefits | Enduring\_Enthusiasm | Unrelenting\_Efforts | Opportunities\_forAutonomy | Teacher\_Support | Classmate\_Support | Exercise\_Behaviour |
| --- | --- | --- | --- | --- | --- | --- | --- | --- | --- |
| Perseverance | .000 | .000 | .000 | .000 | .000 | .000 | .000 | .000 | .000 |
| Exercise\_Benefits | .000 | .000 | .000 | .000 | .000 | .000 | .000 | .000 | .000 |
| Enduring\_Enthusiasm | .237 | .000 | .000 | .000 | .000 | .000 | .000 | .000 | .000 |
| Unrelenting\_Efforts | .226 | .000 | .000 | .000 | .000 | .000 | .000 | .000 | .000 |
| Opportunities\_forAutonomy | .000 | .000 | .000 | .000 | .000 | .000 | .000 | .000 | .000 |
| Teacher\_Support | .000 | .000 | .000 | .000 | .000 | .000 | .000 | .000 | .000 |
| Classmate\_Support | .000 | .000 | .000 | .000 | .000 | .000 | .000 | .000 | .000 |
| Exercise\_Behaviour | .181 | .000 | .000 | .000 | .000 | .000 | .000 | .000 | .000 |
| EE6 | .186 | .795 | .000 | .000 | .000 | .000 | .000 | .000 | .000 |
| EE5 | .205 | .866 | .000 | .000 | .000 | .000 | .000 | .000 | .000 |
| EE4 | .203 | .858 | .000 | .000 | .000 | .000 | .000 | .000 | .000 |
| EE3 | .205 | .871 | .000 | .000 | .000 | .000 | .000 | .000 | .000 |
| EE2 | .202 | .849 | .000 | .000 | .000 | .000 | .000 | .000 | .000 |
| EE1 | .198 | .841 | .000 | .000 | .000 | .000 | .000 | .000 | .000 |
| UE6 | .168 | .729 | .000 | .000 | .000 | .000 | .000 | .000 | .000 |
| UE5 | .192 | .822 | .000 | .000 | .000 | .000 | .000 | .000 | .000 |
| UE4 | .195 | .829 | .000 | .000 | .000 | .000 | .000 | .000 | .000 |
| UE3 | .194 | .827 | .000 | .000 | .000 | .000 | .000 | .000 | .000 |
| UE2 | .185 | .792 | .000 | .000 | .000 | .000 | .000 | .000 | .000 |
| UE1 | .181 | .772 | .000 | .000 | .000 | .000 | .000 | .000 | .000 |
| OA1 | .801 | .000 | .000 | .000 | .000 | .000 | .000 | .000 | .000 |
| OA2 | .724 | .000 | .000 | .000 | .000 | .000 | .000 | .000 | .000 |
| OA3 | .751 | .000 | .000 | .000 | .000 | .000 | .000 | .000 | .000 |
| OA4 | .808 | .000 | .000 | .000 | .000 | .000 | .000 | .000 | .000 |
| OA5 | .758 | .000 | .000 | .000 | .000 | .000 | .000 | .000 | .000 |
| CS1 | .701 | .000 | .000 | .000 | .000 | .000 | .000 | .000 | .000 |
| CS2 | .689 | .000 | .000 | .000 | .000 | .000 | .000 | .000 | .000 |
| CS3 | .670 | .000 | .000 | .000 | .000 | .000 | .000 | .000 | .000 |
| CS4 | .715 | .000 | .000 | .000 | .000 | .000 | .000 | .000 | .000 |
| CS5 | .699 | .000 | .000 | .000 | .000 | .000 | .000 | .000 | .000 |
| CS6 | .706 | .000 | .000 | .000 | .000 | .000 | .000 | .000 | .000 |
| CS7 | .706 | .000 | .000 | .000 | .000 | .000 | .000 | .000 | .000 |
| CS8 | .701 | .000 | .000 | .000 | .000 | .000 | .000 | .000 | .000 |
| CS9 | .711 | .000 | .000 | .000 | .000 | .000 | .000 | .000 | .000 |
| CS10 | .705 | .000 | .000 | .000 | .000 | .000 | .000 | .000 | .000 |
| CS11 | .706 | .000 | .000 | .000 | .000 | .000 | .000 | .000 | .000 |
| CS12 | .669 | .000 | .000 | .000 | .000 | .000 | .000 | .000 | .000 |
| CS13 | .651 | .000 | .000 | .000 | .000 | .000 | .000 | .000 | .000 |
| TS1 | .605 | .000 | .000 | .000 | .000 | .000 | .000 | .000 | .000 |
| TS2 | .630 | .000 | .000 | .000 | .000 | .000 | .000 | .000 | .000 |
| TS3 | .636 | .000 | .000 | .000 | .000 | .000 | .000 | .000 | .000 |
| TS4 | .616 | .000 | .000 | .000 | .000 | .000 | .000 | .000 | .000 |
| TS5 | .618 | .000 | .000 | .000 | .000 | .000 | .000 | .000 | .000 |
| TS6 | .580 | .000 | .000 | .000 | .000 | .000 | .000 | .000 | .000 |
| TS7 | .572 | .000 | .000 | .000 | .000 | .000 | .000 | .000 | .000 |
| EB1 | .250 | .000 | .000 | .000 | .000 | .000 | .000 | .000 | .000 |
| EB2 | .240 | .000 | .000 | .000 | .000 | .000 | .000 | .000 | .000 |
| EB3 | .225 | .000 | .000 | .000 | .000 | .000 | .000 | .000 | .000 |
| EB4 | .243 | .000 | .000 | .000 | .000 | .000 | .000 | .000 | .000 |
| EB5 | .210 | .000 | .000 | .000 | .000 | .000 | .000 | .000 | .000 |
| EX3 | .266 | .246 | .261 | .000 | .000 | .000 | .000 | .000 | .000 |
| EX2 | .296 | .280 | .288 | .000 | .000 | .000 | .000 | .000 | .000 |
| EX1 | .273 | .248 | .262 | .000 | .000 | .000 | .000 | .000 | .000 |

##### Standardized Indirect Effects - Two Tailed Significance (PC) (Group number 1 - Default model)

|  | Perceive\_SchoolClimate | Perseverance | Exercise\_Benefits | Enduring\_Enthusiasm | Unrelenting\_Efforts | Opportunities\_forAutonomy | Teacher\_Support | Classmate\_Support | Exercise\_Behaviour |
| --- | --- | --- | --- | --- | --- | --- | --- | --- | --- |
| Perseverance | ... | ... | ... | ... | ... | ... | ... | ... | ... |
| Exercise\_Benefits | ... | ... | ... | ... | ... | ... | ... | ... | ... |
| Enduring\_Enthusiasm | .003 | ... | ... | ... | ... | ... | ... | ... | ... |
| Unrelenting\_Efforts | .003 | ... | ... | ... | ... | ... | ... | ... | ... |
| Opportunities\_forAutonomy | ... | ... | ... | ... | ... | ... | ... | ... | ... |
| Teacher\_Support | ... | ... | ... | ... | ... | ... | ... | ... | ... |
| Classmate\_Support | ... | ... | ... | ... | ... | ... | ... | ... | ... |
| Exercise\_Behaviour | .000 | ... | ... | ... | ... | ... | ... | ... | ... |
| EE6 | .003 | .000 | ... | ... | ... | ... | ... | ... | ... |
| EE5 | .003 | .000 | ... | ... | ... | ... | ... | ... | ... |
| EE4 | .003 | .000 | ... | ... | ... | ... | ... | ... | ... |
| EE3 | .003 | .000 | ... | ... | ... | ... | ... | ... | ... |
| EE2 | .003 | .000 | ... | ... | ... | ... | ... | ... | ... |
| EE1 | .003 | .000 | ... | ... | ... | ... | ... | ... | ... |
| UE6 | .003 | .000 | ... | ... | ... | ... | ... | ... | ... |
| UE5 | .003 | .000 | ... | ... | ... | ... | ... | ... | ... |
| UE4 | .003 | .000 | ... | ... | ... | ... | ... | ... | ... |
| UE3 | .003 | .000 | ... | ... | ... | ... | ... | ... | ... |
| UE2 | .003 | .000 | ... | ... | ... | ... | ... | ... | ... |
| UE1 | .003 | .000 | ... | ... | ... | ... | ... | ... | ... |
| OA1 | .000 | ... | ... | ... | ... | ... | ... | ... | ... |
| OA2 | .000 | ... | ... | ... | ... | ... | ... | ... | ... |
| OA3 | .000 | ... | ... | ... | ... | ... | ... | ... | ... |
| OA4 | .000 | ... | ... | ... | ... | ... | ... | ... | ... |
| OA5 | .000 | ... | ... | ... | ... | ... | ... | ... | ... |
| CS1 | .000 | ... | ... | ... | ... | ... | ... | ... | ... |
| CS2 | .000 | ... | ... | ... | ... | ... | ... | ... | ... |
| CS3 | .000 | ... | ... | ... | ... | ... | ... | ... | ... |
| CS4 | .000 | ... | ... | ... | ... | ... | ... | ... | ... |
| CS5 | .000 | ... | ... | ... | ... | ... | ... | ... | ... |
| CS6 | .000 | ... | ... | ... | ... | ... | ... | ... | ... |
| CS7 | .000 | ... | ... | ... | ... | ... | ... | ... | ... |
| CS8 | .000 | ... | ... | ... | ... | ... | ... | ... | ... |
| CS9 | .000 | ... | ... | ... | ... | ... | ... | ... | ... |
| CS10 | .000 | ... | ... | ... | ... | ... | ... | ... | ... |
| CS11 | .000 | ... | ... | ... | ... | ... | ... | ... | ... |
| CS12 | .000 | ... | ... | ... | ... | ... | ... | ... | ... |
| CS13 | .000 | ... | ... | ... | ... | ... | ... | ... | ... |
| TS1 | .000 | ... | ... | ... | ... | ... | ... | ... | ... |
| TS2 | .000 | ... | ... | ... | ... | ... | ... | ... | ... |
| TS3 | .000 | ... | ... | ... | ... | ... | ... | ... | ... |
| TS4 | .000 | ... | ... | ... | ... | ... | ... | ... | ... |
| TS5 | .000 | ... | ... | ... | ... | ... | ... | ... | ... |
| TS6 | .000 | ... | ... | ... | ... | ... | ... | ... | ... |
| TS7 | .000 | ... | ... | ... | ... | ... | ... | ... | ... |
| EB1 | .000 | ... | ... | ... | ... | ... | ... | ... | ... |
| EB2 | .000 | ... | ... | ... | ... | ... | ... | ... | ... |
| EB3 | .000 | ... | ... | ... | ... | ... | ... | ... | ... |
| EB4 | .000 | ... | ... | ... | ... | ... | ... | ... | ... |
| EB5 | .000 | ... | ... | ... | ... | ... | ... | ... | ... |
| EX3 | .000 | .000 | .000 | ... | ... | ... | ... | ... | ... |
| EX2 | .000 | .000 | .000 | ... | ... | ... | ... | ... | ... |
| EX1 | .000 | .000 | .000 | ... | ... | ... | ... | ... | ... |

##### Bias-corrected percentile method (Group number 1 - Default model)

##### 95% confidence intervals (bias-corrected percentile method)

##### Scalar Estimates (Group number 1 - Default model)

##### Regression Weights: (Group number 1 - Default model)

| Parameter | | | Estimate | Lower | Upper | P |
| --- | --- | --- | --- | --- | --- | --- |
| Perseverance | <--- | Perceive\_SchoolClimate | .348 | .125 | .612 | .003 |
| Exercise\_Benefits | <--- | Perceive\_SchoolClimate | .276 | .134 | .488 | .000 |
| Exercise\_Behaviour | <--- | Perceive\_SchoolClimate | .330 | .107 | .639 | .004 |
| Classmate\_Support | <--- | Perceive\_SchoolClimate | 1.000 | 1.000 | 1.000 | ... |
| Teacher\_Support | <--- | Perceive\_SchoolClimate | 1.118 | .808 | 1.466 | .000 |
| Opportunities\_forAutonomy | <--- | Perceive\_SchoolClimate | 1.241 | .978 | 1.602 | .000 |
| Unrelenting\_Efforts | <--- | Perseverance | 1.000 | 1.000 | 1.000 | ... |
| Exercise\_Behaviour | <--- | Exercise\_Benefits | .512 | .273 | .849 | .000 |
| Exercise\_Behaviour | <--- | Perseverance | .257 | .135 | .387 | .000 |
| Enduring\_Enthusiasm | <--- | Perseverance | 1.178 | .844 | 1.700 | .000 |
| EX1 | <--- | Exercise\_Behaviour | 1.000 | 1.000 | 1.000 | ... |
| EX2 | <--- | Exercise\_Behaviour | 1.114 | .939 | 1.325 | .000 |
| EX3 | <--- | Exercise\_Behaviour | 1.052 | .878 | 1.255 | .000 |
| EB5 | <--- | Exercise\_Benefits | 1.000 | 1.000 | 1.000 | ... |
| EB4 | <--- | Exercise\_Benefits | 1.103 | .904 | 1.399 | .000 |
| EB3 | <--- | Exercise\_Benefits | .972 | .768 | 1.242 | .000 |
| EB2 | <--- | Exercise\_Benefits | 1.085 | .861 | 1.386 | .000 |
| EB1 | <--- | Exercise\_Benefits | 1.138 | .913 | 1.473 | .000 |
| TS7 | <--- | Teacher\_Support | 1.000 | 1.000 | 1.000 | ... |
| TS6 | <--- | Teacher\_Support | .787 | .704 | .860 | .000 |
| TS5 | <--- | Teacher\_Support | .900 | .834 | .958 | .000 |
| TS4 | <--- | Teacher\_Support | .882 | .814 | .942 | .000 |
| TS3 | <--- | Teacher\_Support | .895 | .835 | .950 | .000 |
| TS2 | <--- | Teacher\_Support | .904 | .842 | .962 | .000 |
| TS1 | <--- | Teacher\_Support | .813 | .739 | .877 | .000 |
| CS11 | <--- | Classmate\_Support | 1.054 | .972 | 1.155 | .000 |
| CS10 | <--- | Classmate\_Support | 1.040 | .979 | 1.108 | .000 |
| CS9 | <--- | Classmate\_Support | 1.079 | 1.018 | 1.147 | .000 |
| CS8 | <--- | Classmate\_Support | 1.023 | .944 | 1.113 | .000 |
| CS7 | <--- | Classmate\_Support | 1.000 | 1.000 | 1.000 | ... |
| CS6 | <--- | Classmate\_Support | 1.036 | .968 | 1.116 | .000 |
| CS5 | <--- | Classmate\_Support | 1.025 | .949 | 1.113 | .000 |
| CS4 | <--- | Classmate\_Support | 1.019 | .950 | 1.100 | .000 |
| CS3 | <--- | Classmate\_Support | 1.028 | .950 | 1.128 | .000 |
| OA5 | <--- | Opportunities\_forAutonomy | 1.302 | 1.178 | 1.478 | .000 |
| OA4 | <--- | Opportunities\_forAutonomy | .974 | .865 | 1.082 | .000 |
| OA3 | <--- | Opportunities\_forAutonomy | 1.000 | 1.000 | 1.000 | ... |
| OA2 | <--- | Opportunities\_forAutonomy | .953 | .862 | 1.046 | .000 |
| OA1 | <--- | Opportunities\_forAutonomy | 1.009 | .905 | 1.115 | .000 |
| UE1 | <--- | Unrelenting\_Efforts | 1.000 | 1.000 | 1.000 | ... |
| UE2 | <--- | Unrelenting\_Efforts | 1.004 | .936 | 1.080 | .000 |
| UE3 | <--- | Unrelenting\_Efforts | 1.124 | 1.049 | 1.210 | .000 |
| UE4 | <--- | Unrelenting\_Efforts | 1.125 | 1.053 | 1.216 | .000 |
| UE5 | <--- | Unrelenting\_Efforts | 1.061 | .993 | 1.143 | .000 |
| UE6 | <--- | Unrelenting\_Efforts | 1.087 | .987 | 1.186 | .000 |
| EE1 | <--- | Enduring\_Enthusiasm | 1.000 | 1.000 | 1.000 | ... |
| EE2 | <--- | Enduring\_Enthusiasm | .978 | .920 | 1.040 | .000 |
| EE3 | <--- | Enduring\_Enthusiasm | 1.036 | .978 | 1.103 | .000 |
| EE4 | <--- | Enduring\_Enthusiasm | .995 | .934 | 1.062 | .000 |
| EE5 | <--- | Enduring\_Enthusiasm | 1.020 | .966 | 1.081 | .000 |
| EE6 | <--- | Enduring\_Enthusiasm | .961 | .893 | 1.031 | .000 |
| CS1 | <--- | Classmate\_Support | 1.017 | .941 | 1.107 | .000 |
| CS13 | <--- | Classmate\_Support | 1.204 | 1.102 | 1.334 | .000 |
| CS12 | <--- | Classmate\_Support | 1.284 | 1.176 | 1.424 | .000 |
| CS2 | <--- | Classmate\_Support | .991 | .918 | 1.074 | .000 |

##### Standardized Regression Weights: (Group number 1 - Default model)

| Parameter | | | Estimate | Lower | Upper | P |
| --- | --- | --- | --- | --- | --- | --- |
| Perseverance | <--- | Perceive\_SchoolClimate | .176 | .069 | .273 | .002 |
| Exercise\_Benefits | <--- | Perceive\_SchoolClimate | .265 | .132 | .404 | .000 |
| Exercise\_Behaviour | <--- | Perceive\_SchoolClimate | .169 | .054 | .295 | .004 |
| Classmate\_Support | <--- | Perceive\_SchoolClimate | .671 | .560 | .768 | .001 |
| Teacher\_Support | <--- | Perceive\_SchoolClimate | .550 | .423 | .670 | .000 |
| Opportunities\_forAutonomy | <--- | Perceive\_SchoolClimate | .845 | .730 | .954 | .000 |
| Unrelenting\_Efforts | <--- | Perseverance | .820 | .684 | .967 | .000 |
| Exercise\_Behaviour | <--- | Exercise\_Benefits | .274 | .153 | .405 | .000 |
| Exercise\_Behaviour | <--- | Perseverance | .261 | .141 | .370 | .001 |
| Enduring\_Enthusiasm | <--- | Perseverance | .842 | .707 | .998 | .000 |
| EX1 | <--- | Exercise\_Behaviour | .645 | .566 | .718 | .000 |
| EX2 | <--- | Exercise\_Behaviour | .727 | .641 | .805 | .000 |
| EX3 | <--- | Exercise\_Behaviour | .643 | .565 | .717 | .000 |
| EB5 | <--- | Exercise\_Benefits | .504 | .405 | .590 | .000 |
| EB4 | <--- | Exercise\_Benefits | .598 | .497 | .681 | .000 |
| EB3 | <--- | Exercise\_Benefits | .531 | .420 | .623 | .001 |
| EB2 | <--- | Exercise\_Benefits | .599 | .489 | .682 | .001 |
| EB1 | <--- | Exercise\_Benefits | .605 | .499 | .690 | .000 |
| TS7 | <--- | Teacher\_Support | .850 | .812 | .885 | .001 |
| TS6 | <--- | Teacher\_Support | .863 | .817 | .899 | .000 |
| TS5 | <--- | Teacher\_Support | .920 | .887 | .945 | .001 |
| TS4 | <--- | Teacher\_Support | .915 | .880 | .943 | .000 |
| TS3 | <--- | Teacher\_Support | .946 | .919 | .967 | .000 |
| TS2 | <--- | Teacher\_Support | .937 | .909 | .958 | .001 |
| TS1 | <--- | Teacher\_Support | .900 | .860 | .930 | .000 |
| CS11 | <--- | Classmate\_Support | .915 | .884 | .942 | .000 |
| CS10 | <--- | Classmate\_Support | .911 | .879 | .938 | .000 |
| CS9 | <--- | Classmate\_Support | .918 | .888 | .943 | .000 |
| CS8 | <--- | Classmate\_Support | .905 | .871 | .933 | .000 |
| CS7 | <--- | Classmate\_Support | .911 | .876 | .939 | .000 |
| CS6 | <--- | Classmate\_Support | .913 | .880 | .938 | .000 |
| CS5 | <--- | Classmate\_Support | .900 | .864 | .929 | .000 |
| CS4 | <--- | Classmate\_Support | .923 | .890 | .949 | .000 |
| CS3 | <--- | Classmate\_Support | .856 | .799 | .898 | .001 |
| OA5 | <--- | Opportunities\_forAutonomy | .793 | .751 | .832 | .000 |
| OA4 | <--- | Opportunities\_forAutonomy | .841 | .773 | .888 | .001 |
| OA3 | <--- | Opportunities\_forAutonomy | .773 | .709 | .828 | .000 |
| OA2 | <--- | Opportunities\_forAutonomy | .744 | .673 | .802 | .000 |
| OA1 | <--- | Opportunities\_forAutonomy | .836 | .780 | .881 | .000 |
| UE1 | <--- | Unrelenting\_Efforts | .793 | .752 | .828 | .001 |
| UE2 | <--- | Unrelenting\_Efforts | .818 | .782 | .849 | .000 |
| UE3 | <--- | Unrelenting\_Efforts | .855 | .822 | .883 | .001 |
| UE4 | <--- | Unrelenting\_Efforts | .854 | .822 | .882 | .000 |
| UE5 | <--- | Unrelenting\_Efforts | .845 | .814 | .872 | .001 |
| UE6 | <--- | Unrelenting\_Efforts | .741 | .672 | .793 | .001 |
| EE1 | <--- | Enduring\_Enthusiasm | .835 | .802 | .861 | .001 |
| EE2 | <--- | Enduring\_Enthusiasm | .846 | .815 | .874 | .000 |
| EE3 | <--- | Enduring\_Enthusiasm | .863 | .833 | .889 | .000 |
| EE4 | <--- | Enduring\_Enthusiasm | .854 | .820 | .881 | .001 |
| EE5 | <--- | Enduring\_Enthusiasm | .860 | .829 | .885 | .001 |
| EE6 | <--- | Enduring\_Enthusiasm | .781 | .735 | .821 | .001 |
| CS1 | <--- | Classmate\_Support | .905 | .871 | .933 | .000 |
| CS13 | <--- | Classmate\_Support | .834 | .793 | .869 | .001 |
| CS12 | <--- | Classmate\_Support | .861 | .832 | .884 | .001 |
| CS2 | <--- | Classmate\_Support | .888 | .849 | .918 | .000 |

##### Variances: (Group number 1 - Default model)

| Parameter | | | Estimate | Lower | Upper | P |
| --- | --- | --- | --- | --- | --- | --- |
| Perceive\_SchoolClimate |  |  | .139 | .088 | .203 | .000 |
| e3 |  |  | .140 | .085 | .204 | .000 |
| e1 |  |  | .528 | .350 | .776 | .000 |
| e6 |  |  | .399 | .303 | .507 | .000 |
| e7 |  |  | .170 | .122 | .236 | .000 |
| e8 |  |  | .086 | .028 | .149 | .005 |
| e4 |  |  | .265 | .056 | .440 | .018 |
| e5 |  |  | .309 | -.001 | .535 | .050 |
| e2 |  |  | .411 | .308 | .544 | .000 |
| e52 |  |  | .737 | .614 | .860 | .000 |
| e53 |  |  | .581 | .440 | .723 | .000 |
| e54 |  |  | .828 | .693 | .962 | .000 |
| e51 |  |  | .443 | .389 | .500 | .000 |
| e50 |  |  | .329 | .278 | .384 | .000 |
| e49 |  |  | .362 | .314 | .414 | .000 |
| e48 |  |  | .317 | .267 | .372 | .000 |
| e47 |  |  | .338 | .282 | .395 | .000 |
| e15 |  |  | .220 | .162 | .286 | .000 |
| e14 |  |  | .122 | .095 | .153 | .000 |
| e13 |  |  | .085 | .059 | .117 | .000 |
| e12 |  |  | .087 | .060 | .121 | .000 |
| e11 |  |  | .054 | .033 | .079 | .000 |
| e10 |  |  | .065 | .043 | .092 | .000 |
| e9 |  |  | .089 | .066 | .117 | .000 |
| e29 |  |  | .196 | .151 | .248 | .000 |
| e28 |  |  | .177 | .146 | .214 | .000 |
| e27 |  |  | .067 | .047 | .090 | .000 |
| e26 |  |  | .069 | .050 | .091 | .000 |
| e25 |  |  | .067 | .046 | .090 | .000 |
| e24 |  |  | .072 | .052 | .094 | .000 |
| e23 |  |  | .064 | .046 | .085 | .000 |
| e22 |  |  | .066 | .048 | .087 | .000 |
| e21 |  |  | .076 | .055 | .099 | .000 |
| e20 |  |  | .056 | .039 | .076 | .000 |
| e19 |  |  | .119 | .086 | .166 | .000 |
| e18 |  |  | .082 | .062 | .105 | .000 |
| e16 |  |  | .071 | .052 | .092 | .000 |
| e34 |  |  | .300 | .231 | .374 | .000 |
| e33 |  |  | .118 | .087 | .154 | .000 |
| e32 |  |  | .202 | .163 | .250 | .000 |
| e31 |  |  | .220 | .182 | .261 | .000 |
| e30 |  |  | .131 | .101 | .166 | .000 |
| e35 |  |  | .476 | .409 | .558 | .000 |
| e36 |  |  | .403 | .345 | .470 | .000 |
| e37 |  |  | .376 | .310 | .452 | .000 |
| e38 |  |  | .381 | .314 | .454 | .000 |
| e39 |  |  | .364 | .310 | .427 | .000 |
| e40 |  |  | .785 | .636 | .986 | .000 |
| e41 |  |  | .463 | .398 | .539 | .000 |
| e42 |  |  | .404 | .341 | .473 | .000 |
| e43 |  |  | .392 | .324 | .467 | .000 |
| e44 |  |  | .392 | .325 | .469 | .000 |
| e45 |  |  | .392 | .326 | .466 | .000 |
| e46 |  |  | .627 | .524 | .744 | .000 |

##### Squared Multiple Correlations: (Group number 1 - Default model)

| Parameter | | | Estimate | Lower | Upper | P |
| --- | --- | --- | --- | --- | --- | --- |
| Perseverance |  |  | .031 | .005 | .074 | .000 |
| Exercise\_Benefits |  |  | .070 | .017 | .163 | .000 |
| Enduring\_Enthusiasm |  |  | .710 | .499 | .996 | .000 |
| Unrelenting\_Efforts |  |  | .673 | .468 | .935 | .000 |
| Opportunities\_forAutonomy |  |  | .713 | .533 | .909 | .000 |
| Teacher\_Support |  |  | .303 | .179 | .449 | .000 |
| Classmate\_Support |  |  | .450 | .314 | .589 | .001 |
| Exercise\_Behaviour |  |  | .218 | .127 | .308 | .002 |
| EE6 |  |  | .610 | .540 | .673 | .001 |
| EE5 |  |  | .739 | .687 | .783 | .001 |
| EE4 |  |  | .729 | .672 | .776 | .001 |
| EE3 |  |  | .745 | .694 | .791 | .000 |
| EE2 |  |  | .716 | .664 | .764 | .000 |
| EE1 |  |  | .697 | .643 | .742 | .001 |
| UE6 |  |  | .549 | .452 | .629 | .001 |
| UE5 |  |  | .714 | .662 | .760 | .001 |
| UE4 |  |  | .729 | .676 | .779 | .000 |
| UE3 |  |  | .731 | .675 | .780 | .001 |
| UE2 |  |  | .669 | .612 | .722 | .000 |
| UE1 |  |  | .629 | .565 | .686 | .001 |
| OA1 |  |  | .699 | .608 | .776 | .000 |
| OA2 |  |  | .553 | .453 | .643 | .000 |
| OA3 |  |  | .597 | .503 | .686 | .000 |
| OA4 |  |  | .707 | .598 | .789 | .001 |
| OA5 |  |  | .629 | .564 | .692 | .000 |
| CS1 |  |  | .819 | .758 | .871 | .000 |
| CS2 |  |  | .788 | .720 | .844 | .000 |
| CS3 |  |  | .732 | .638 | .807 | .001 |
| CS4 |  |  | .851 | .793 | .900 | .000 |
| CS5 |  |  | .810 | .747 | .863 | .000 |
| CS6 |  |  | .833 | .774 | .880 | .000 |
| CS7 |  |  | .829 | .768 | .881 | .000 |
| CS8 |  |  | .818 | .758 | .871 | .000 |
| CS9 |  |  | .842 | .789 | .889 | .000 |
| CS10 |  |  | .830 | .773 | .879 | .000 |
| CS11 |  |  | .837 | .782 | .886 | .000 |
| CS12 |  |  | .742 | .691 | .782 | .001 |
| CS13 |  |  | .695 | .629 | .755 | .001 |
| TS1 |  |  | .810 | .740 | .864 | .000 |
| TS2 |  |  | .878 | .827 | .919 | .001 |
| TS3 |  |  | .894 | .844 | .936 | .000 |
| TS4 |  |  | .836 | .774 | .889 | .000 |
| TS5 |  |  | .845 | .786 | .893 | .001 |
| TS6 |  |  | .744 | .668 | .809 | .000 |
| TS7 |  |  | .723 | .660 | .783 | .001 |
| EB1 |  |  | .366 | .249 | .477 | .000 |
| EB2 |  |  | .359 | .239 | .465 | .001 |
| EB3 |  |  | .282 | .176 | .388 | .001 |
| EB4 |  |  | .358 | .247 | .463 | .000 |
| EB5 |  |  | .254 | .164 | .348 | .000 |
| EX3 |  |  | .413 | .320 | .514 | .000 |
| EX2 |  |  | .529 | .411 | .648 | .000 |
| EX1 |  |  | .416 | .320 | .516 | .000 |

##### User-defined estimands: (Group number 1 - Default model)

| Parameter | | | Estimate | Lower | Upper | P |
| --- | --- | --- | --- | --- | --- | --- |
| Estimand 1 |  |  | .089 | .032 | .191 | .002 |
| Estimand 2 |  |  | .141 | .060 | .287 | .000 |
| Estimand 3 |  |  | .330 | .107 | .639 | .004 |
| Estimand 4 |  |  | -.052 | -.198 | .064 | .348 |
| Estimand 5 |  |  | .560 | .312 | .898 | .000 |

##### Matrices (Group number 1 - Default model)

##### Total Effects (Group number 1 - Default model)

##### Total Effects - Lower Bounds (BC) (Group number 1 - Default model)

|  | Perceive\_SchoolClimate | Perseverance | Exercise\_Benefits | Enduring\_Enthusiasm | Unrelenting\_Efforts | Opportunities\_forAutonomy | Teacher\_Support | Classmate\_Support | Exercise\_Behaviour |
| --- | --- | --- | --- | --- | --- | --- | --- | --- | --- |
| Perseverance | .125 | .000 | .000 | .000 | .000 | .000 | .000 | .000 | .000 |
| Exercise\_Benefits | .134 | .000 | .000 | .000 | .000 | .000 | .000 | .000 | .000 |
| Enduring\_Enthusiasm | .147 | .844 | .000 | .000 | .000 | .000 | .000 | .000 | .000 |
| Unrelenting\_Efforts | .125 | 1.000 | .000 | .000 | .000 | .000 | .000 | .000 | .000 |
| Opportunities\_forAutonomy | .978 | .000 | .000 | .000 | .000 | .000 | .000 | .000 | .000 |
| Teacher\_Support | .808 | .000 | .000 | .000 | .000 | .000 | .000 | .000 | .000 |
| Classmate\_Support | 1.000 | .000 | .000 | .000 | .000 | .000 | .000 | .000 | .000 |
| Exercise\_Behaviour | .312 | .135 | .273 | .000 | .000 | .000 | .000 | .000 | .000 |
| EE6 | .142 | .803 | .000 | .893 | .000 | .000 | .000 | .000 | .000 |
| EE5 | .150 | .859 | .000 | .966 | .000 | .000 | .000 | .000 | .000 |
| EE4 | .148 | .834 | .000 | .934 | .000 | .000 | .000 | .000 | .000 |
| EE3 | .155 | .877 | .000 | .978 | .000 | .000 | .000 | .000 | .000 |
| EE2 | .143 | .830 | .000 | .920 | .000 | .000 | .000 | .000 | .000 |
| EE1 | .147 | .844 | .000 | 1.000 | .000 | .000 | .000 | .000 | .000 |
| UE6 | .141 | .987 | .000 | .000 | .987 | .000 | .000 | .000 | .000 |
| UE5 | .137 | .993 | .000 | .000 | .993 | .000 | .000 | .000 | .000 |
| UE4 | .141 | 1.053 | .000 | .000 | 1.053 | .000 | .000 | .000 | .000 |
| UE3 | .142 | 1.049 | .000 | .000 | 1.049 | .000 | .000 | .000 | .000 |
| UE2 | .129 | .936 | .000 | .000 | .936 | .000 | .000 | .000 | .000 |
| UE1 | .125 | 1.000 | .000 | .000 | 1.000 | .000 | .000 | .000 | .000 |
| OA1 | .983 | .000 | .000 | .000 | .000 | .905 | .000 | .000 | .000 |
| OA2 | .929 | .000 | .000 | .000 | .000 | .862 | .000 | .000 | .000 |
| OA3 | .978 | .000 | .000 | .000 | .000 | 1.000 | .000 | .000 | .000 |
| OA4 | .955 | .000 | .000 | .000 | .000 | .865 | .000 | .000 | .000 |
| OA5 | 1.286 | .000 | .000 | .000 | .000 | 1.178 | .000 | .000 | .000 |
| CS1 | .941 | .000 | .000 | .000 | .000 | .000 | .000 | .941 | .000 |
| CS2 | .918 | .000 | .000 | .000 | .000 | .000 | .000 | .918 | .000 |
| CS3 | .950 | .000 | .000 | .000 | .000 | .000 | .000 | .950 | .000 |
| CS4 | .950 | .000 | .000 | .000 | .000 | .000 | .000 | .950 | .000 |
| CS5 | .949 | .000 | .000 | .000 | .000 | .000 | .000 | .949 | .000 |
| CS6 | .968 | .000 | .000 | .000 | .000 | .000 | .000 | .968 | .000 |
| CS7 | 1.000 | .000 | .000 | .000 | .000 | .000 | .000 | 1.000 | .000 |
| CS8 | .944 | .000 | .000 | .000 | .000 | .000 | .000 | .944 | .000 |
| CS9 | 1.018 | .000 | .000 | .000 | .000 | .000 | .000 | 1.018 | .000 |
| CS10 | .979 | .000 | .000 | .000 | .000 | .000 | .000 | .979 | .000 |
| CS11 | .972 | .000 | .000 | .000 | .000 | .000 | .000 | .972 | .000 |
| CS12 | 1.176 | .000 | .000 | .000 | .000 | .000 | .000 | 1.176 | .000 |
| CS13 | 1.102 | .000 | .000 | .000 | .000 | .000 | .000 | 1.102 | .000 |
| TS1 | .655 | .000 | .000 | .000 | .000 | .000 | .739 | .000 | .000 |
| TS2 | .728 | .000 | .000 | .000 | .000 | .000 | .842 | .000 | .000 |
| TS3 | .727 | .000 | .000 | .000 | .000 | .000 | .835 | .000 | .000 |
| TS4 | .719 | .000 | .000 | .000 | .000 | .000 | .814 | .000 | .000 |
| TS5 | .724 | .000 | .000 | .000 | .000 | .000 | .834 | .000 | .000 |
| TS6 | .632 | .000 | .000 | .000 | .000 | .000 | .704 | .000 | .000 |
| TS7 | .808 | .000 | .000 | .000 | .000 | .000 | 1.000 | .000 | .000 |
| EB1 | .150 | .000 | .913 | .000 | .000 | .000 | .000 | .000 | .000 |
| EB2 | .148 | .000 | .861 | .000 | .000 | .000 | .000 | .000 | .000 |
| EB3 | .127 | .000 | .768 | .000 | .000 | .000 | .000 | .000 | .000 |
| EB4 | .146 | .000 | .904 | .000 | .000 | .000 | .000 | .000 | .000 |
| EB5 | .134 | .000 | 1.000 | .000 | .000 | .000 | .000 | .000 | .000 |
| EX3 | .339 | .146 | .287 | .000 | .000 | .000 | .000 | .000 | .878 |
| EX2 | .370 | .152 | .315 | .000 | .000 | .000 | .000 | .000 | .939 |
| EX1 | .312 | .135 | .273 | .000 | .000 | .000 | .000 | .000 | 1.000 |

##### Total Effects - Upper Bounds (BC) (Group number 1 - Default model)

|  | Perceive\_SchoolClimate | Perseverance | Exercise\_Benefits | Enduring\_Enthusiasm | Unrelenting\_Efforts | Opportunities\_forAutonomy | Teacher\_Support | Classmate\_Support | Exercise\_Behaviour |
| --- | --- | --- | --- | --- | --- | --- | --- | --- | --- |
| Perseverance | .612 | .000 | .000 | .000 | .000 | .000 | .000 | .000 | .000 |
| Exercise\_Benefits | .488 | .000 | .000 | .000 | .000 | .000 | .000 | .000 | .000 |
| Enduring\_Enthusiasm | .719 | 1.700 | .000 | .000 | .000 | .000 | .000 | .000 | .000 |
| Unrelenting\_Efforts | .612 | 1.000 | .000 | .000 | .000 | .000 | .000 | .000 | .000 |
| Opportunities\_forAutonomy | 1.602 | .000 | .000 | .000 | .000 | .000 | .000 | .000 | .000 |
| Teacher\_Support | 1.466 | .000 | .000 | .000 | .000 | .000 | .000 | .000 | .000 |
| Classmate\_Support | 1.000 | .000 | .000 | .000 | .000 | .000 | .000 | .000 | .000 |
| Exercise\_Behaviour | .898 | .387 | .849 | .000 | .000 | .000 | .000 | .000 | .000 |
| EE6 | .695 | 1.617 | .000 | 1.031 | .000 | .000 | .000 | .000 | .000 |
| EE5 | .739 | 1.723 | .000 | 1.081 | .000 | .000 | .000 | .000 | .000 |
| EE4 | .721 | 1.674 | .000 | 1.062 | .000 | .000 | .000 | .000 | .000 |
| EE3 | .751 | 1.758 | .000 | 1.103 | .000 | .000 | .000 | .000 | .000 |
| EE2 | .704 | 1.651 | .000 | 1.040 | .000 | .000 | .000 | .000 | .000 |
| EE1 | .719 | 1.700 | .000 | 1.000 | .000 | .000 | .000 | .000 | .000 |
| UE6 | .670 | 1.186 | .000 | .000 | 1.186 | .000 | .000 | .000 | .000 |
| UE5 | .654 | 1.143 | .000 | .000 | 1.143 | .000 | .000 | .000 | .000 |
| UE4 | .685 | 1.216 | .000 | .000 | 1.216 | .000 | .000 | .000 | .000 |
| UE3 | .688 | 1.210 | .000 | .000 | 1.210 | .000 | .000 | .000 | .000 |
| UE2 | .613 | 1.080 | .000 | .000 | 1.080 | .000 | .000 | .000 | .000 |
| UE1 | .612 | 1.000 | .000 | .000 | 1.000 | .000 | .000 | .000 | .000 |
| OA1 | 1.633 | .000 | .000 | .000 | .000 | 1.115 | .000 | .000 | .000 |
| OA2 | 1.550 | .000 | .000 | .000 | .000 | 1.046 | .000 | .000 | .000 |
| OA3 | 1.602 | .000 | .000 | .000 | .000 | 1.000 | .000 | .000 | .000 |
| OA4 | 1.577 | .000 | .000 | .000 | .000 | 1.082 | .000 | .000 | .000 |
| OA5 | 2.094 | .000 | .000 | .000 | .000 | 1.478 | .000 | .000 | .000 |
| CS1 | 1.107 | .000 | .000 | .000 | .000 | .000 | .000 | 1.107 | .000 |
| CS2 | 1.074 | .000 | .000 | .000 | .000 | .000 | .000 | 1.074 | .000 |
| CS3 | 1.128 | .000 | .000 | .000 | .000 | .000 | .000 | 1.128 | .000 |
| CS4 | 1.100 | .000 | .000 | .000 | .000 | .000 | .000 | 1.100 | .000 |
| CS5 | 1.113 | .000 | .000 | .000 | .000 | .000 | .000 | 1.113 | .000 |
| CS6 | 1.116 | .000 | .000 | .000 | .000 | .000 | .000 | 1.116 | .000 |
| CS7 | 1.000 | .000 | .000 | .000 | .000 | .000 | .000 | 1.000 | .000 |
| CS8 | 1.113 | .000 | .000 | .000 | .000 | .000 | .000 | 1.113 | .000 |
| CS9 | 1.147 | .000 | .000 | .000 | .000 | .000 | .000 | 1.147 | .000 |
| CS10 | 1.108 | .000 | .000 | .000 | .000 | .000 | .000 | 1.108 | .000 |
| CS11 | 1.155 | .000 | .000 | .000 | .000 | .000 | .000 | 1.155 | .000 |
| CS12 | 1.424 | .000 | .000 | .000 | .000 | .000 | .000 | 1.424 | .000 |
| CS13 | 1.334 | .000 | .000 | .000 | .000 | .000 | .000 | 1.334 | .000 |
| TS1 | 1.211 | .000 | .000 | .000 | .000 | .000 | .877 | .000 | .000 |
| TS2 | 1.333 | .000 | .000 | .000 | .000 | .000 | .962 | .000 | .000 |
| TS3 | 1.326 | .000 | .000 | .000 | .000 | .000 | .950 | .000 | .000 |
| TS4 | 1.307 | .000 | .000 | .000 | .000 | .000 | .942 | .000 | .000 |
| TS5 | 1.332 | .000 | .000 | .000 | .000 | .000 | .958 | .000 | .000 |
| TS6 | 1.177 | .000 | .000 | .000 | .000 | .000 | .860 | .000 | .000 |
| TS7 | 1.466 | .000 | .000 | .000 | .000 | .000 | 1.000 | .000 | .000 |
| EB1 | .536 | .000 | 1.473 | .000 | .000 | .000 | .000 | .000 | .000 |
| EB2 | .505 | .000 | 1.386 | .000 | .000 | .000 | .000 | .000 | .000 |
| EB3 | .479 | .000 | 1.242 | .000 | .000 | .000 | .000 | .000 | .000 |
| EB4 | .516 | .000 | 1.399 | .000 | .000 | .000 | .000 | .000 | .000 |
| EB5 | .488 | .000 | 1.000 | .000 | .000 | .000 | .000 | .000 | .000 |
| EX3 | .929 | .404 | .911 | .000 | .000 | .000 | .000 | .000 | 1.255 |
| EX2 | .980 | .436 | .929 | .000 | .000 | .000 | .000 | .000 | 1.325 |
| EX1 | .898 | .387 | .849 | .000 | .000 | .000 | .000 | .000 | 1.000 |

##### Total Effects - Two Tailed Significance (BC) (Group number 1 - Default model)

|  | Perceive\_SchoolClimate | Perseverance | Exercise\_Benefits | Enduring\_Enthusiasm | Unrelenting\_Efforts | Opportunities\_forAutonomy | Teacher\_Support | Classmate\_Support | Exercise\_Behaviour |
| --- | --- | --- | --- | --- | --- | --- | --- | --- | --- |
| Perseverance | .003 | ... | ... | ... | ... | ... | ... | ... | ... |
| Exercise\_Benefits | .000 | ... | ... | ... | ... | ... | ... | ... | ... |
| Enduring\_Enthusiasm | .003 | .000 | ... | ... | ... | ... | ... | ... | ... |
| Unrelenting\_Efforts | .003 | ... | ... | ... | ... | ... | ... | ... | ... |
| Opportunities\_forAutonomy | .000 | ... | ... | ... | ... | ... | ... | ... | ... |
| Teacher\_Support | .000 | ... | ... | ... | ... | ... | ... | ... | ... |
| Classmate\_Support | ... | ... | ... | ... | ... | ... | ... | ... | ... |
| Exercise\_Behaviour | .000 | .000 | .000 | ... | ... | ... | ... | ... | ... |
| EE6 | .003 | .000 | ... | .000 | ... | ... | ... | ... | ... |
| EE5 | .003 | .000 | ... | .000 | ... | ... | ... | ... | ... |
| EE4 | .003 | .000 | ... | .000 | ... | ... | ... | ... | ... |
| EE3 | .002 | .000 | ... | .000 | ... | ... | ... | ... | ... |
| EE2 | .003 | .000 | ... | .000 | ... | ... | ... | ... | ... |
| EE1 | .003 | .000 | ... | ... | ... | ... | ... | ... | ... |
| UE6 | .002 | .000 | ... | ... | .000 | ... | ... | ... | ... |
| UE5 | .002 | .000 | ... | ... | .000 | ... | ... | ... | ... |
| UE4 | .003 | .000 | ... | ... | .000 | ... | ... | ... | ... |
| UE3 | .002 | .000 | ... | ... | .000 | ... | ... | ... | ... |
| UE2 | .002 | .000 | ... | ... | .000 | ... | ... | ... | ... |
| UE1 | .003 | ... | ... | ... | ... | ... | ... | ... | ... |
| OA1 | .000 | ... | ... | ... | ... | .000 | ... | ... | ... |
| OA2 | .000 | ... | ... | ... | ... | .000 | ... | ... | ... |
| OA3 | .000 | ... | ... | ... | ... | ... | ... | ... | ... |
| OA4 | .000 | ... | ... | ... | ... | .000 | ... | ... | ... |
| OA5 | .000 | ... | ... | ... | ... | .000 | ... | ... | ... |
| CS1 | .000 | ... | ... | ... | ... | ... | ... | .000 | ... |
| CS2 | .000 | ... | ... | ... | ... | ... | ... | .000 | ... |
| CS3 | .000 | ... | ... | ... | ... | ... | ... | .000 | ... |
| CS4 | .000 | ... | ... | ... | ... | ... | ... | .000 | ... |
| CS5 | .000 | ... | ... | ... | ... | ... | ... | .000 | ... |
| CS6 | .000 | ... | ... | ... | ... | ... | ... | .000 | ... |
| CS7 | ... | ... | ... | ... | ... | ... | ... | ... | ... |
| CS8 | .000 | ... | ... | ... | ... | ... | ... | .000 | ... |
| CS9 | .000 | ... | ... | ... | ... | ... | ... | .000 | ... |
| CS10 | .000 | ... | ... | ... | ... | ... | ... | .000 | ... |
| CS11 | .000 | ... | ... | ... | ... | ... | ... | .000 | ... |
| CS12 | .000 | ... | ... | ... | ... | ... | ... | .000 | ... |
| CS13 | .000 | ... | ... | ... | ... | ... | ... | .000 | ... |
| TS1 | .000 | ... | ... | ... | ... | ... | .000 | ... | ... |
| TS2 | .000 | ... | ... | ... | ... | ... | .000 | ... | ... |
| TS3 | .000 | ... | ... | ... | ... | ... | .000 | ... | ... |
| TS4 | .000 | ... | ... | ... | ... | ... | .000 | ... | ... |
| TS5 | .000 | ... | ... | ... | ... | ... | .000 | ... | ... |
| TS6 | .000 | ... | ... | ... | ... | ... | .000 | ... | ... |
| TS7 | .000 | ... | ... | ... | ... | ... | ... | ... | ... |
| EB1 | .000 | ... | .000 | ... | ... | ... | ... | ... | ... |
| EB2 | .000 | ... | .000 | ... | ... | ... | ... | ... | ... |
| EB3 | .000 | ... | .000 | ... | ... | ... | ... | ... | ... |
| EB4 | .000 | ... | .000 | ... | ... | ... | ... | ... | ... |
| EB5 | .000 | ... | ... | ... | ... | ... | ... | ... | ... |
| EX3 | .000 | .000 | .000 | ... | ... | ... | ... | ... | .000 |
| EX2 | .000 | .000 | .000 | ... | ... | ... | ... | ... | .000 |
| EX1 | .000 | .000 | .000 | ... | ... | ... | ... | ... | ... |

##### Standardized Total Effects (Group number 1 - Default model)

##### Standardized Total Effects - Lower Bounds (BC) (Group number 1 - Default model)

|  | Perceive\_SchoolClimate | Perseverance | Exercise\_Benefits | Enduring\_Enthusiasm | Unrelenting\_Efforts | Opportunities\_forAutonomy | Teacher\_Support | Classmate\_Support | Exercise\_Behaviour |
| --- | --- | --- | --- | --- | --- | --- | --- | --- | --- |
| Perseverance | .069 | .000 | .000 | .000 | .000 | .000 | .000 | .000 | .000 |
| Exercise\_Benefits | .132 | .000 | .000 | .000 | .000 | .000 | .000 | .000 | .000 |
| Enduring\_Enthusiasm | .056 | .707 | .000 | .000 | .000 | .000 | .000 | .000 | .000 |
| Unrelenting\_Efforts | .056 | .684 | .000 | .000 | .000 | .000 | .000 | .000 | .000 |
| Opportunities\_forAutonomy | .730 | .000 | .000 | .000 | .000 | .000 | .000 | .000 | .000 |
| Teacher\_Support | .423 | .000 | .000 | .000 | .000 | .000 | .000 | .000 | .000 |
| Classmate\_Support | .560 | .000 | .000 | .000 | .000 | .000 | .000 | .000 | .000 |
| Exercise\_Behaviour | .168 | .141 | .153 | .000 | .000 | .000 | .000 | .000 | .000 |
| EE6 | .043 | .539 | .000 | .735 | .000 | .000 | .000 | .000 | .000 |
| EE5 | .048 | .600 | .000 | .829 | .000 | .000 | .000 | .000 | .000 |
| EE4 | .048 | .600 | .000 | .820 | .000 | .000 | .000 | .000 | .000 |
| EE3 | .049 | .605 | .000 | .833 | .000 | .000 | .000 | .000 | .000 |
| EE2 | .048 | .597 | .000 | .815 | .000 | .000 | .000 | .000 | .000 |
| EE1 | .047 | .586 | .000 | .802 | .000 | .000 | .000 | .000 | .000 |
| UE6 | .041 | .496 | .000 | .000 | .672 | .000 | .000 | .000 | .000 |
| UE5 | .047 | .573 | .000 | .000 | .814 | .000 | .000 | .000 | .000 |
| UE4 | .048 | .579 | .000 | .000 | .822 | .000 | .000 | .000 | .000 |
| UE3 | .048 | .578 | .000 | .000 | .822 | .000 | .000 | .000 | .000 |
| UE2 | .046 | .555 | .000 | .000 | .782 | .000 | .000 | .000 | .000 |
| UE1 | .044 | .532 | .000 | .000 | .752 | .000 | .000 | .000 | .000 |
| OA1 | .604 | .000 | .000 | .000 | .000 | .780 | .000 | .000 | .000 |
| OA2 | .526 | .000 | .000 | .000 | .000 | .673 | .000 | .000 | .000 |
| OA3 | .550 | .000 | .000 | .000 | .000 | .709 | .000 | .000 | .000 |
| OA4 | .607 | .000 | .000 | .000 | .000 | .773 | .000 | .000 | .000 |
| OA5 | .576 | .000 | .000 | .000 | .000 | .751 | .000 | .000 | .000 |
| CS1 | .506 | .000 | .000 | .000 | .000 | .000 | .000 | .871 | .000 |
| CS2 | .494 | .000 | .000 | .000 | .000 | .000 | .000 | .849 | .000 |
| CS3 | .471 | .000 | .000 | .000 | .000 | .000 | .000 | .799 | .000 |
| CS4 | .515 | .000 | .000 | .000 | .000 | .000 | .000 | .890 | .000 |
| CS5 | .503 | .000 | .000 | .000 | .000 | .000 | .000 | .864 | .000 |
| CS6 | .513 | .000 | .000 | .000 | .000 | .000 | .000 | .880 | .000 |
| CS7 | .509 | .000 | .000 | .000 | .000 | .000 | .000 | .876 | .000 |
| CS8 | .507 | .000 | .000 | .000 | .000 | .000 | .000 | .871 | .000 |
| CS9 | .514 | .000 | .000 | .000 | .000 | .000 | .000 | .888 | .000 |
| CS10 | .510 | .000 | .000 | .000 | .000 | .000 | .000 | .879 | .000 |
| CS11 | .512 | .000 | .000 | .000 | .000 | .000 | .000 | .884 | .000 |
| CS12 | .480 | .000 | .000 | .000 | .000 | .000 | .000 | .832 | .000 |
| CS13 | .466 | .000 | .000 | .000 | .000 | .000 | .000 | .793 | .000 |
| TS1 | .383 | .000 | .000 | .000 | .000 | .000 | .860 | .000 | .000 |
| TS2 | .398 | .000 | .000 | .000 | .000 | .000 | .909 | .000 | .000 |
| TS3 | .401 | .000 | .000 | .000 | .000 | .000 | .919 | .000 | .000 |
| TS4 | .385 | .000 | .000 | .000 | .000 | .000 | .880 | .000 | .000 |
| TS5 | .388 | .000 | .000 | .000 | .000 | .000 | .887 | .000 | .000 |
| TS6 | .367 | .000 | .000 | .000 | .000 | .000 | .817 | .000 | .000 |
| TS7 | .357 | .000 | .000 | .000 | .000 | .000 | .812 | .000 | .000 |
| EB1 | .080 | .000 | .499 | .000 | .000 | .000 | .000 | .000 | .000 |
| EB2 | .082 | .000 | .489 | .000 | .000 | .000 | .000 | .000 | .000 |
| EB3 | .070 | .000 | .420 | .000 | .000 | .000 | .000 | .000 | .000 |
| EB4 | .080 | .000 | .497 | .000 | .000 | .000 | .000 | .000 | .000 |
| EB5 | .067 | .000 | .405 | .000 | .000 | .000 | .000 | .000 | .000 |
| EX3 | .110 | .091 | .097 | .000 | .000 | .000 | .000 | .000 | .565 |
| EX2 | .123 | .102 | .113 | .000 | .000 | .000 | .000 | .000 | .641 |
| EX1 | .106 | .091 | .097 | .000 | .000 | .000 | .000 | .000 | .566 |

##### Standardized Total Effects - Upper Bounds (BC) (Group number 1 - Default model)

|  | Perceive\_SchoolClimate | Perseverance | Exercise\_Benefits | Enduring\_Enthusiasm | Unrelenting\_Efforts | Opportunities\_forAutonomy | Teacher\_Support | Classmate\_Support | Exercise\_Behaviour |
| --- | --- | --- | --- | --- | --- | --- | --- | --- | --- |
| Perseverance | .273 | .000 | .000 | .000 | .000 | .000 | .000 | .000 | .000 |
| Exercise\_Benefits | .404 | .000 | .000 | .000 | .000 | .000 | .000 | .000 | .000 |
| Enduring\_Enthusiasm | .238 | .998 | .000 | .000 | .000 | .000 | .000 | .000 | .000 |
| Unrelenting\_Efforts | .229 | .967 | .000 | .000 | .000 | .000 | .000 | .000 | .000 |
| Opportunities\_forAutonomy | .954 | .000 | .000 | .000 | .000 | .000 | .000 | .000 | .000 |
| Teacher\_Support | .670 | .000 | .000 | .000 | .000 | .000 | .000 | .000 | .000 |
| Classmate\_Support | .768 | .000 | .000 | .000 | .000 | .000 | .000 | .000 | .000 |
| Exercise\_Behaviour | .408 | .370 | .405 | .000 | .000 | .000 | .000 | .000 | .000 |
| EE6 | .188 | .786 | .000 | .821 | .000 | .000 | .000 | .000 | .000 |
| EE5 | .205 | .862 | .000 | .885 | .000 | .000 | .000 | .000 | .000 |
| EE4 | .204 | .856 | .000 | .881 | .000 | .000 | .000 | .000 | .000 |
| EE3 | .206 | .866 | .000 | .889 | .000 | .000 | .000 | .000 | .000 |
| EE2 | .202 | .849 | .000 | .874 | .000 | .000 | .000 | .000 | .000 |
| EE1 | .199 | .840 | .000 | .861 | .000 | .000 | .000 | .000 | .000 |
| UE6 | .170 | .728 | .000 | .000 | .793 | .000 | .000 | .000 | .000 |
| UE5 | .194 | .822 | .000 | .000 | .872 | .000 | .000 | .000 | .000 |
| UE4 | .196 | .829 | .000 | .000 | .882 | .000 | .000 | .000 | .000 |
| UE3 | .197 | .825 | .000 | .000 | .883 | .000 | .000 | .000 | .000 |
| UE2 | .188 | .792 | .000 | .000 | .849 | .000 | .000 | .000 | .000 |
| UE1 | .183 | .770 | .000 | .000 | .828 | .000 | .000 | .000 | .000 |
| OA1 | .804 | .000 | .000 | .000 | .000 | .881 | .000 | .000 | .000 |
| OA2 | .729 | .000 | .000 | .000 | .000 | .802 | .000 | .000 | .000 |
| OA3 | .754 | .000 | .000 | .000 | .000 | .828 | .000 | .000 | .000 |
| OA4 | .811 | .000 | .000 | .000 | .000 | .888 | .000 | .000 | .000 |
| OA5 | .759 | .000 | .000 | .000 | .000 | .832 | .000 | .000 | .000 |
| CS1 | .698 | .000 | .000 | .000 | .000 | .000 | .000 | .933 | .000 |
| CS2 | .686 | .000 | .000 | .000 | .000 | .000 | .000 | .918 | .000 |
| CS3 | .665 | .000 | .000 | .000 | .000 | .000 | .000 | .898 | .000 |
| CS4 | .711 | .000 | .000 | .000 | .000 | .000 | .000 | .949 | .000 |
| CS5 | .697 | .000 | .000 | .000 | .000 | .000 | .000 | .929 | .000 |
| CS6 | .705 | .000 | .000 | .000 | .000 | .000 | .000 | .938 | .000 |
| CS7 | .705 | .000 | .000 | .000 | .000 | .000 | .000 | .939 | .000 |
| CS8 | .698 | .000 | .000 | .000 | .000 | .000 | .000 | .933 | .000 |
| CS9 | .708 | .000 | .000 | .000 | .000 | .000 | .000 | .943 | .000 |
| CS10 | .701 | .000 | .000 | .000 | .000 | .000 | .000 | .938 | .000 |
| CS11 | .704 | .000 | .000 | .000 | .000 | .000 | .000 | .942 | .000 |
| CS12 | .666 | .000 | .000 | .000 | .000 | .000 | .000 | .884 | .000 |
| CS13 | .647 | .000 | .000 | .000 | .000 | .000 | .000 | .869 | .000 |
| TS1 | .606 | .000 | .000 | .000 | .000 | .000 | .930 | .000 | .000 |
| TS2 | .629 | .000 | .000 | .000 | .000 | .000 | .958 | .000 | .000 |
| TS3 | .636 | .000 | .000 | .000 | .000 | .000 | .967 | .000 | .000 |
| TS4 | .616 | .000 | .000 | .000 | .000 | .000 | .943 | .000 | .000 |
| TS5 | .617 | .000 | .000 | .000 | .000 | .000 | .945 | .000 | .000 |
| TS6 | .583 | .000 | .000 | .000 | .000 | .000 | .899 | .000 | .000 |
| TS7 | .570 | .000 | .000 | .000 | .000 | .000 | .885 | .000 | .000 |
| EB1 | .253 | .000 | .690 | .000 | .000 | .000 | .000 | .000 | .000 |
| EB2 | .244 | .000 | .682 | .000 | .000 | .000 | .000 | .000 | .000 |
| EB3 | .228 | .000 | .623 | .000 | .000 | .000 | .000 | .000 | .000 |
| EB4 | .246 | .000 | .681 | .000 | .000 | .000 | .000 | .000 | .000 |
| EB5 | .214 | .000 | .590 | .000 | .000 | .000 | .000 | .000 | .000 |
| EX3 | .266 | .245 | .266 | .000 | .000 | .000 | .000 | .000 | .717 |
| EX2 | .296 | .276 | .290 | .000 | .000 | .000 | .000 | .000 | .805 |
| EX1 | .273 | .247 | .264 | .000 | .000 | .000 | .000 | .000 | .718 |

##### Standardized Total Effects - Two Tailed Significance (BC) (Group number 1 - Default model)

|  | Perceive\_SchoolClimate | Perseverance | Exercise\_Benefits | Enduring\_Enthusiasm | Unrelenting\_Efforts | Opportunities\_forAutonomy | Teacher\_Support | Classmate\_Support | Exercise\_Behaviour |
| --- | --- | --- | --- | --- | --- | --- | --- | --- | --- |
| Perseverance | .002 | ... | ... | ... | ... | ... | ... | ... | ... |
| Exercise\_Benefits | .000 | ... | ... | ... | ... | ... | ... | ... | ... |
| Enduring\_Enthusiasm | .003 | .000 | ... | ... | ... | ... | ... | ... | ... |
| Unrelenting\_Efforts | .002 | .000 | ... | ... | ... | ... | ... | ... | ... |
| Opportunities\_forAutonomy | .000 | ... | ... | ... | ... | ... | ... | ... | ... |
| Teacher\_Support | .000 | ... | ... | ... | ... | ... | ... | ... | ... |
| Classmate\_Support | .001 | ... | ... | ... | ... | ... | ... | ... | ... |
| Exercise\_Behaviour | .000 | .001 | .000 | ... | ... | ... | ... | ... | ... |
| EE6 | .003 | .001 | ... | .001 | ... | ... | ... | ... | ... |
| EE5 | .003 | .000 | ... | .001 | ... | ... | ... | ... | ... |
| EE4 | .003 | .000 | ... | .001 | ... | ... | ... | ... | ... |
| EE3 | .003 | .000 | ... | .000 | ... | ... | ... | ... | ... |
| EE2 | .003 | .000 | ... | .000 | ... | ... | ... | ... | ... |
| EE1 | .003 | .000 | ... | .001 | ... | ... | ... | ... | ... |
| UE6 | .002 | .000 | ... | ... | .001 | ... | ... | ... | ... |
| UE5 | .002 | .000 | ... | ... | .001 | ... | ... | ... | ... |
| UE4 | .002 | .000 | ... | ... | .000 | ... | ... | ... | ... |
| UE3 | .002 | .000 | ... | ... | .001 | ... | ... | ... | ... |
| UE2 | .002 | .000 | ... | ... | .000 | ... | ... | ... | ... |
| UE1 | .002 | .000 | ... | ... | .001 | ... | ... | ... | ... |
| OA1 | .000 | ... | ... | ... | ... | .000 | ... | ... | ... |
| OA2 | .000 | ... | ... | ... | ... | .000 | ... | ... | ... |
| OA3 | .000 | ... | ... | ... | ... | .000 | ... | ... | ... |
| OA4 | .000 | ... | ... | ... | ... | .001 | ... | ... | ... |
| OA5 | .000 | ... | ... | ... | ... | .000 | ... | ... | ... |
| CS1 | .000 | ... | ... | ... | ... | ... | ... | .000 | ... |
| CS2 | .001 | ... | ... | ... | ... | ... | ... | .000 | ... |
| CS3 | .001 | ... | ... | ... | ... | ... | ... | .001 | ... |
| CS4 | .001 | ... | ... | ... | ... | ... | ... | .000 | ... |
| CS5 | .000 | ... | ... | ... | ... | ... | ... | .000 | ... |
| CS6 | .000 | ... | ... | ... | ... | ... | ... | .000 | ... |
| CS7 | .000 | ... | ... | ... | ... | ... | ... | .000 | ... |
| CS8 | .000 | ... | ... | ... | ... | ... | ... | .000 | ... |
| CS9 | .001 | ... | ... | ... | ... | ... | ... | .000 | ... |
| CS10 | .001 | ... | ... | ... | ... | ... | ... | .000 | ... |
| CS11 | .000 | ... | ... | ... | ... | ... | ... | .000 | ... |
| CS12 | .001 | ... | ... | ... | ... | ... | ... | .001 | ... |
| CS13 | .000 | ... | ... | ... | ... | ... | ... | .001 | ... |
| TS1 | .000 | ... | ... | ... | ... | ... | .000 | ... | ... |
| TS2 | .000 | ... | ... | ... | ... | ... | .001 | ... | ... |
| TS3 | .000 | ... | ... | ... | ... | ... | .000 | ... | ... |
| TS4 | .000 | ... | ... | ... | ... | ... | .000 | ... | ... |
| TS5 | .000 | ... | ... | ... | ... | ... | .001 | ... | ... |
| TS6 | .000 | ... | ... | ... | ... | ... | .000 | ... | ... |
| TS7 | .000 | ... | ... | ... | ... | ... | .001 | ... | ... |
| EB1 | .000 | ... | .000 | ... | ... | ... | ... | ... | ... |
| EB2 | .000 | ... | .001 | ... | ... | ... | ... | ... | ... |
| EB3 | .000 | ... | .001 | ... | ... | ... | ... | ... | ... |
| EB4 | .000 | ... | .000 | ... | ... | ... | ... | ... | ... |
| EB5 | .000 | ... | .000 | ... | ... | ... | ... | ... | ... |
| EX3 | .000 | .000 | .000 | ... | ... | ... | ... | ... | .000 |
| EX2 | .000 | .001 | .000 | ... | ... | ... | ... | ... | .000 |
| EX1 | .000 | .000 | .000 | ... | ... | ... | ... | ... | .000 |

##### Direct Effects (Group number 1 - Default model)

##### Direct Effects - Lower Bounds (BC) (Group number 1 - Default model)

|  | Perceive\_SchoolClimate | Perseverance | Exercise\_Benefits | Enduring\_Enthusiasm | Unrelenting\_Efforts | Opportunities\_forAutonomy | Teacher\_Support | Classmate\_Support | Exercise\_Behaviour |
| --- | --- | --- | --- | --- | --- | --- | --- | --- | --- |
| Perseverance | .125 | .000 | .000 | .000 | .000 | .000 | .000 | .000 | .000 |
| Exercise\_Benefits | .134 | .000 | .000 | .000 | .000 | .000 | .000 | .000 | .000 |
| Enduring\_Enthusiasm | .000 | .844 | .000 | .000 | .000 | .000 | .000 | .000 | .000 |
| Unrelenting\_Efforts | .000 | 1.000 | .000 | .000 | .000 | .000 | .000 | .000 | .000 |
| Opportunities\_forAutonomy | .978 | .000 | .000 | .000 | .000 | .000 | .000 | .000 | .000 |
| Teacher\_Support | .808 | .000 | .000 | .000 | .000 | .000 | .000 | .000 | .000 |
| Classmate\_Support | 1.000 | .000 | .000 | .000 | .000 | .000 | .000 | .000 | .000 |
| Exercise\_Behaviour | .107 | .135 | .273 | .000 | .000 | .000 | .000 | .000 | .000 |
| EE6 | .000 | .000 | .000 | .893 | .000 | .000 | .000 | .000 | .000 |
| EE5 | .000 | .000 | .000 | .966 | .000 | .000 | .000 | .000 | .000 |
| EE4 | .000 | .000 | .000 | .934 | .000 | .000 | .000 | .000 | .000 |
| EE3 | .000 | .000 | .000 | .978 | .000 | .000 | .000 | .000 | .000 |
| EE2 | .000 | .000 | .000 | .920 | .000 | .000 | .000 | .000 | .000 |
| EE1 | .000 | .000 | .000 | 1.000 | .000 | .000 | .000 | .000 | .000 |
| UE6 | .000 | .000 | .000 | .000 | .987 | .000 | .000 | .000 | .000 |
| UE5 | .000 | .000 | .000 | .000 | .993 | .000 | .000 | .000 | .000 |
| UE4 | .000 | .000 | .000 | .000 | 1.053 | .000 | .000 | .000 | .000 |
| UE3 | .000 | .000 | .000 | .000 | 1.049 | .000 | .000 | .000 | .000 |
| UE2 | .000 | .000 | .000 | .000 | .936 | .000 | .000 | .000 | .000 |
| UE1 | .000 | .000 | .000 | .000 | 1.000 | .000 | .000 | .000 | .000 |
| OA1 | .000 | .000 | .000 | .000 | .000 | .905 | .000 | .000 | .000 |
| OA2 | .000 | .000 | .000 | .000 | .000 | .862 | .000 | .000 | .000 |
| OA3 | .000 | .000 | .000 | .000 | .000 | 1.000 | .000 | .000 | .000 |
| OA4 | .000 | .000 | .000 | .000 | .000 | .865 | .000 | .000 | .000 |
| OA5 | .000 | .000 | .000 | .000 | .000 | 1.178 | .000 | .000 | .000 |
| CS1 | .000 | .000 | .000 | .000 | .000 | .000 | .000 | .941 | .000 |
| CS2 | .000 | .000 | .000 | .000 | .000 | .000 | .000 | .918 | .000 |
| CS3 | .000 | .000 | .000 | .000 | .000 | .000 | .000 | .950 | .000 |
| CS4 | .000 | .000 | .000 | .000 | .000 | .000 | .000 | .950 | .000 |
| CS5 | .000 | .000 | .000 | .000 | .000 | .000 | .000 | .949 | .000 |
| CS6 | .000 | .000 | .000 | .000 | .000 | .000 | .000 | .968 | .000 |
| CS7 | .000 | .000 | .000 | .000 | .000 | .000 | .000 | 1.000 | .000 |
| CS8 | .000 | .000 | .000 | .000 | .000 | .000 | .000 | .944 | .000 |
| CS9 | .000 | .000 | .000 | .000 | .000 | .000 | .000 | 1.018 | .000 |
| CS10 | .000 | .000 | .000 | .000 | .000 | .000 | .000 | .979 | .000 |
| CS11 | .000 | .000 | .000 | .000 | .000 | .000 | .000 | .972 | .000 |
| CS12 | .000 | .000 | .000 | .000 | .000 | .000 | .000 | 1.176 | .000 |
| CS13 | .000 | .000 | .000 | .000 | .000 | .000 | .000 | 1.102 | .000 |
| TS1 | .000 | .000 | .000 | .000 | .000 | .000 | .739 | .000 | .000 |
| TS2 | .000 | .000 | .000 | .000 | .000 | .000 | .842 | .000 | .000 |
| TS3 | .000 | .000 | .000 | .000 | .000 | .000 | .835 | .000 | .000 |
| TS4 | .000 | .000 | .000 | .000 | .000 | .000 | .814 | .000 | .000 |
| TS5 | .000 | .000 | .000 | .000 | .000 | .000 | .834 | .000 | .000 |
| TS6 | .000 | .000 | .000 | .000 | .000 | .000 | .704 | .000 | .000 |
| TS7 | .000 | .000 | .000 | .000 | .000 | .000 | 1.000 | .000 | .000 |
| EB1 | .000 | .000 | .913 | .000 | .000 | .000 | .000 | .000 | .000 |
| EB2 | .000 | .000 | .861 | .000 | .000 | .000 | .000 | .000 | .000 |
| EB3 | .000 | .000 | .768 | .000 | .000 | .000 | .000 | .000 | .000 |
| EB4 | .000 | .000 | .904 | .000 | .000 | .000 | .000 | .000 | .000 |
| EB5 | .000 | .000 | 1.000 | .000 | .000 | .000 | .000 | .000 | .000 |
| EX3 | .000 | .000 | .000 | .000 | .000 | .000 | .000 | .000 | .878 |
| EX2 | .000 | .000 | .000 | .000 | .000 | .000 | .000 | .000 | .939 |
| EX1 | .000 | .000 | .000 | .000 | .000 | .000 | .000 | .000 | 1.000 |

##### Direct Effects - Upper Bounds (BC) (Group number 1 - Default model)

|  | Perceive\_SchoolClimate | Perseverance | Exercise\_Benefits | Enduring\_Enthusiasm | Unrelenting\_Efforts | Opportunities\_forAutonomy | Teacher\_Support | Classmate\_Support | Exercise\_Behaviour |
| --- | --- | --- | --- | --- | --- | --- | --- | --- | --- |
| Perseverance | .612 | .000 | .000 | .000 | .000 | .000 | .000 | .000 | .000 |
| Exercise\_Benefits | .488 | .000 | .000 | .000 | .000 | .000 | .000 | .000 | .000 |
| Enduring\_Enthusiasm | .000 | 1.700 | .000 | .000 | .000 | .000 | .000 | .000 | .000 |
| Unrelenting\_Efforts | .000 | 1.000 | .000 | .000 | .000 | .000 | .000 | .000 | .000 |
| Opportunities\_forAutonomy | 1.602 | .000 | .000 | .000 | .000 | .000 | .000 | .000 | .000 |
| Teacher\_Support | 1.466 | .000 | .000 | .000 | .000 | .000 | .000 | .000 | .000 |
| Classmate\_Support | 1.000 | .000 | .000 | .000 | .000 | .000 | .000 | .000 | .000 |
| Exercise\_Behaviour | .639 | .387 | .849 | .000 | .000 | .000 | .000 | .000 | .000 |
| EE6 | .000 | .000 | .000 | 1.031 | .000 | .000 | .000 | .000 | .000 |
| EE5 | .000 | .000 | .000 | 1.081 | .000 | .000 | .000 | .000 | .000 |
| EE4 | .000 | .000 | .000 | 1.062 | .000 | .000 | .000 | .000 | .000 |
| EE3 | .000 | .000 | .000 | 1.103 | .000 | .000 | .000 | .000 | .000 |
| EE2 | .000 | .000 | .000 | 1.040 | .000 | .000 | .000 | .000 | .000 |
| EE1 | .000 | .000 | .000 | 1.000 | .000 | .000 | .000 | .000 | .000 |
| UE6 | .000 | .000 | .000 | .000 | 1.186 | .000 | .000 | .000 | .000 |
| UE5 | .000 | .000 | .000 | .000 | 1.143 | .000 | .000 | .000 | .000 |
| UE4 | .000 | .000 | .000 | .000 | 1.216 | .000 | .000 | .000 | .000 |
| UE3 | .000 | .000 | .000 | .000 | 1.210 | .000 | .000 | .000 | .000 |
| UE2 | .000 | .000 | .000 | .000 | 1.080 | .000 | .000 | .000 | .000 |
| UE1 | .000 | .000 | .000 | .000 | 1.000 | .000 | .000 | .000 | .000 |
| OA1 | .000 | .000 | .000 | .000 | .000 | 1.115 | .000 | .000 | .000 |
| OA2 | .000 | .000 | .000 | .000 | .000 | 1.046 | .000 | .000 | .000 |
| OA3 | .000 | .000 | .000 | .000 | .000 | 1.000 | .000 | .000 | .000 |
| OA4 | .000 | .000 | .000 | .000 | .000 | 1.082 | .000 | .000 | .000 |
| OA5 | .000 | .000 | .000 | .000 | .000 | 1.478 | .000 | .000 | .000 |
| CS1 | .000 | .000 | .000 | .000 | .000 | .000 | .000 | 1.107 | .000 |
| CS2 | .000 | .000 | .000 | .000 | .000 | .000 | .000 | 1.074 | .000 |
| CS3 | .000 | .000 | .000 | .000 | .000 | .000 | .000 | 1.128 | .000 |
| CS4 | .000 | .000 | .000 | .000 | .000 | .000 | .000 | 1.100 | .000 |
| CS5 | .000 | .000 | .000 | .000 | .000 | .000 | .000 | 1.113 | .000 |
| CS6 | .000 | .000 | .000 | .000 | .000 | .000 | .000 | 1.116 | .000 |
| CS7 | .000 | .000 | .000 | .000 | .000 | .000 | .000 | 1.000 | .000 |
| CS8 | .000 | .000 | .000 | .000 | .000 | .000 | .000 | 1.113 | .000 |
| CS9 | .000 | .000 | .000 | .000 | .000 | .000 | .000 | 1.147 | .000 |
| CS10 | .000 | .000 | .000 | .000 | .000 | .000 | .000 | 1.108 | .000 |
| CS11 | .000 | .000 | .000 | .000 | .000 | .000 | .000 | 1.155 | .000 |
| CS12 | .000 | .000 | .000 | .000 | .000 | .000 | .000 | 1.424 | .000 |
| CS13 | .000 | .000 | .000 | .000 | .000 | .000 | .000 | 1.334 | .000 |
| TS1 | .000 | .000 | .000 | .000 | .000 | .000 | .877 | .000 | .000 |
| TS2 | .000 | .000 | .000 | .000 | .000 | .000 | .962 | .000 | .000 |
| TS3 | .000 | .000 | .000 | .000 | .000 | .000 | .950 | .000 | .000 |
| TS4 | .000 | .000 | .000 | .000 | .000 | .000 | .942 | .000 | .000 |
| TS5 | .000 | .000 | .000 | .000 | .000 | .000 | .958 | .000 | .000 |
| TS6 | .000 | .000 | .000 | .000 | .000 | .000 | .860 | .000 | .000 |
| TS7 | .000 | .000 | .000 | .000 | .000 | .000 | 1.000 | .000 | .000 |
| EB1 | .000 | .000 | 1.473 | .000 | .000 | .000 | .000 | .000 | .000 |
| EB2 | .000 | .000 | 1.386 | .000 | .000 | .000 | .000 | .000 | .000 |
| EB3 | .000 | .000 | 1.242 | .000 | .000 | .000 | .000 | .000 | .000 |
| EB4 | .000 | .000 | 1.399 | .000 | .000 | .000 | .000 | .000 | .000 |
| EB5 | .000 | .000 | 1.000 | .000 | .000 | .000 | .000 | .000 | .000 |
| EX3 | .000 | .000 | .000 | .000 | .000 | .000 | .000 | .000 | 1.255 |
| EX2 | .000 | .000 | .000 | .000 | .000 | .000 | .000 | .000 | 1.325 |
| EX1 | .000 | .000 | .000 | .000 | .000 | .000 | .000 | .000 | 1.000 |

##### Direct Effects - Two Tailed Significance (BC) (Group number 1 - Default model)

|  | Perceive\_SchoolClimate | Perseverance | Exercise\_Benefits | Enduring\_Enthusiasm | Unrelenting\_Efforts | Opportunities\_forAutonomy | Teacher\_Support | Classmate\_Support | Exercise\_Behaviour |
| --- | --- | --- | --- | --- | --- | --- | --- | --- | --- |
| Perseverance | .003 | ... | ... | ... | ... | ... | ... | ... | ... |
| Exercise\_Benefits | .000 | ... | ... | ... | ... | ... | ... | ... | ... |
| Enduring\_Enthusiasm | ... | .000 | ... | ... | ... | ... | ... | ... | ... |
| Unrelenting\_Efforts | ... | ... | ... | ... | ... | ... | ... | ... | ... |
| Opportunities\_forAutonomy | .000 | ... | ... | ... | ... | ... | ... | ... | ... |
| Teacher\_Support | .000 | ... | ... | ... | ... | ... | ... | ... | ... |
| Classmate\_Support | ... | ... | ... | ... | ... | ... | ... | ... | ... |
| Exercise\_Behaviour | .004 | .000 | .000 | ... | ... | ... | ... | ... | ... |
| EE6 | ... | ... | ... | .000 | ... | ... | ... | ... | ... |
| EE5 | ... | ... | ... | .000 | ... | ... | ... | ... | ... |
| EE4 | ... | ... | ... | .000 | ... | ... | ... | ... | ... |
| EE3 | ... | ... | ... | .000 | ... | ... | ... | ... | ... |
| EE2 | ... | ... | ... | .000 | ... | ... | ... | ... | ... |
| EE1 | ... | ... | ... | ... | ... | ... | ... | ... | ... |
| UE6 | ... | ... | ... | ... | .000 | ... | ... | ... | ... |
| UE5 | ... | ... | ... | ... | .000 | ... | ... | ... | ... |
| UE4 | ... | ... | ... | ... | .000 | ... | ... | ... | ... |
| UE3 | ... | ... | ... | ... | .000 | ... | ... | ... | ... |
| UE2 | ... | ... | ... | ... | .000 | ... | ... | ... | ... |
| UE1 | ... | ... | ... | ... | ... | ... | ... | ... | ... |
| OA1 | ... | ... | ... | ... | ... | .000 | ... | ... | ... |
| OA2 | ... | ... | ... | ... | ... | .000 | ... | ... | ... |
| OA3 | ... | ... | ... | ... | ... | ... | ... | ... | ... |
| OA4 | ... | ... | ... | ... | ... | .000 | ... | ... | ... |
| OA5 | ... | ... | ... | ... | ... | .000 | ... | ... | ... |
| CS1 | ... | ... | ... | ... | ... | ... | ... | .000 | ... |
| CS2 | ... | ... | ... | ... | ... | ... | ... | .000 | ... |
| CS3 | ... | ... | ... | ... | ... | ... | ... | .000 | ... |
| CS4 | ... | ... | ... | ... | ... | ... | ... | .000 | ... |
| CS5 | ... | ... | ... | ... | ... | ... | ... | .000 | ... |
| CS6 | ... | ... | ... | ... | ... | ... | ... | .000 | ... |
| CS7 | ... | ... | ... | ... | ... | ... | ... | ... | ... |
| CS8 | ... | ... | ... | ... | ... | ... | ... | .000 | ... |
| CS9 | ... | ... | ... | ... | ... | ... | ... | .000 | ... |
| CS10 | ... | ... | ... | ... | ... | ... | ... | .000 | ... |
| CS11 | ... | ... | ... | ... | ... | ... | ... | .000 | ... |
| CS12 | ... | ... | ... | ... | ... | ... | ... | .000 | ... |
| CS13 | ... | ... | ... | ... | ... | ... | ... | .000 | ... |
| TS1 | ... | ... | ... | ... | ... | ... | .000 | ... | ... |
| TS2 | ... | ... | ... | ... | ... | ... | .000 | ... | ... |
| TS3 | ... | ... | ... | ... | ... | ... | .000 | ... | ... |
| TS4 | ... | ... | ... | ... | ... | ... | .000 | ... | ... |
| TS5 | ... | ... | ... | ... | ... | ... | .000 | ... | ... |
| TS6 | ... | ... | ... | ... | ... | ... | .000 | ... | ... |
| TS7 | ... | ... | ... | ... | ... | ... | ... | ... | ... |
| EB1 | ... | ... | .000 | ... | ... | ... | ... | ... | ... |
| EB2 | ... | ... | .000 | ... | ... | ... | ... | ... | ... |
| EB3 | ... | ... | .000 | ... | ... | ... | ... | ... | ... |
| EB4 | ... | ... | .000 | ... | ... | ... | ... | ... | ... |
| EB5 | ... | ... | ... | ... | ... | ... | ... | ... | ... |
| EX3 | ... | ... | ... | ... | ... | ... | ... | ... | .000 |
| EX2 | ... | ... | ... | ... | ... | ... | ... | ... | .000 |
| EX1 | ... | ... | ... | ... | ... | ... | ... | ... | ... |

##### Standardized Direct Effects (Group number 1 - Default model)

##### Standardized Direct Effects - Lower Bounds (BC) (Group number 1 - Default model)

|  | Perceive\_SchoolClimate | Perseverance | Exercise\_Benefits | Enduring\_Enthusiasm | Unrelenting\_Efforts | Opportunities\_forAutonomy | Teacher\_Support | Classmate\_Support | Exercise\_Behaviour |
| --- | --- | --- | --- | --- | --- | --- | --- | --- | --- |
| Perseverance | .069 | .000 | .000 | .000 | .000 | .000 | .000 | .000 | .000 |
| Exercise\_Benefits | .132 | .000 | .000 | .000 | .000 | .000 | .000 | .000 | .000 |
| Enduring\_Enthusiasm | .000 | .707 | .000 | .000 | .000 | .000 | .000 | .000 | .000 |
| Unrelenting\_Efforts | .000 | .684 | .000 | .000 | .000 | .000 | .000 | .000 | .000 |
| Opportunities\_forAutonomy | .730 | .000 | .000 | .000 | .000 | .000 | .000 | .000 | .000 |
| Teacher\_Support | .423 | .000 | .000 | .000 | .000 | .000 | .000 | .000 | .000 |
| Classmate\_Support | .560 | .000 | .000 | .000 | .000 | .000 | .000 | .000 | .000 |
| Exercise\_Behaviour | .054 | .141 | .153 | .000 | .000 | .000 | .000 | .000 | .000 |
| EE6 | .000 | .000 | .000 | .735 | .000 | .000 | .000 | .000 | .000 |
| EE5 | .000 | .000 | .000 | .829 | .000 | .000 | .000 | .000 | .000 |
| EE4 | .000 | .000 | .000 | .820 | .000 | .000 | .000 | .000 | .000 |
| EE3 | .000 | .000 | .000 | .833 | .000 | .000 | .000 | .000 | .000 |
| EE2 | .000 | .000 | .000 | .815 | .000 | .000 | .000 | .000 | .000 |
| EE1 | .000 | .000 | .000 | .802 | .000 | .000 | .000 | .000 | .000 |
| UE6 | .000 | .000 | .000 | .000 | .672 | .000 | .000 | .000 | .000 |
| UE5 | .000 | .000 | .000 | .000 | .814 | .000 | .000 | .000 | .000 |
| UE4 | .000 | .000 | .000 | .000 | .822 | .000 | .000 | .000 | .000 |
| UE3 | .000 | .000 | .000 | .000 | .822 | .000 | .000 | .000 | .000 |
| UE2 | .000 | .000 | .000 | .000 | .782 | .000 | .000 | .000 | .000 |
| UE1 | .000 | .000 | .000 | .000 | .752 | .000 | .000 | .000 | .000 |
| OA1 | .000 | .000 | .000 | .000 | .000 | .780 | .000 | .000 | .000 |
| OA2 | .000 | .000 | .000 | .000 | .000 | .673 | .000 | .000 | .000 |
| OA3 | .000 | .000 | .000 | .000 | .000 | .709 | .000 | .000 | .000 |
| OA4 | .000 | .000 | .000 | .000 | .000 | .773 | .000 | .000 | .000 |
| OA5 | .000 | .000 | .000 | .000 | .000 | .751 | .000 | .000 | .000 |
| CS1 | .000 | .000 | .000 | .000 | .000 | .000 | .000 | .871 | .000 |
| CS2 | .000 | .000 | .000 | .000 | .000 | .000 | .000 | .849 | .000 |
| CS3 | .000 | .000 | .000 | .000 | .000 | .000 | .000 | .799 | .000 |
| CS4 | .000 | .000 | .000 | .000 | .000 | .000 | .000 | .890 | .000 |
| CS5 | .000 | .000 | .000 | .000 | .000 | .000 | .000 | .864 | .000 |
| CS6 | .000 | .000 | .000 | .000 | .000 | .000 | .000 | .880 | .000 |
| CS7 | .000 | .000 | .000 | .000 | .000 | .000 | .000 | .876 | .000 |
| CS8 | .000 | .000 | .000 | .000 | .000 | .000 | .000 | .871 | .000 |
| CS9 | .000 | .000 | .000 | .000 | .000 | .000 | .000 | .888 | .000 |
| CS10 | .000 | .000 | .000 | .000 | .000 | .000 | .000 | .879 | .000 |
| CS11 | .000 | .000 | .000 | .000 | .000 | .000 | .000 | .884 | .000 |
| CS12 | .000 | .000 | .000 | .000 | .000 | .000 | .000 | .832 | .000 |
| CS13 | .000 | .000 | .000 | .000 | .000 | .000 | .000 | .793 | .000 |
| TS1 | .000 | .000 | .000 | .000 | .000 | .000 | .860 | .000 | .000 |
| TS2 | .000 | .000 | .000 | .000 | .000 | .000 | .909 | .000 | .000 |
| TS3 | .000 | .000 | .000 | .000 | .000 | .000 | .919 | .000 | .000 |
| TS4 | .000 | .000 | .000 | .000 | .000 | .000 | .880 | .000 | .000 |
| TS5 | .000 | .000 | .000 | .000 | .000 | .000 | .887 | .000 | .000 |
| TS6 | .000 | .000 | .000 | .000 | .000 | .000 | .817 | .000 | .000 |
| TS7 | .000 | .000 | .000 | .000 | .000 | .000 | .812 | .000 | .000 |
| EB1 | .000 | .000 | .499 | .000 | .000 | .000 | .000 | .000 | .000 |
| EB2 | .000 | .000 | .489 | .000 | .000 | .000 | .000 | .000 | .000 |
| EB3 | .000 | .000 | .420 | .000 | .000 | .000 | .000 | .000 | .000 |
| EB4 | .000 | .000 | .497 | .000 | .000 | .000 | .000 | .000 | .000 |
| EB5 | .000 | .000 | .405 | .000 | .000 | .000 | .000 | .000 | .000 |
| EX3 | .000 | .000 | .000 | .000 | .000 | .000 | .000 | .000 | .565 |
| EX2 | .000 | .000 | .000 | .000 | .000 | .000 | .000 | .000 | .641 |
| EX1 | .000 | .000 | .000 | .000 | .000 | .000 | .000 | .000 | .566 |

##### Standardized Direct Effects - Upper Bounds (BC) (Group number 1 - Default model)

|  | Perceive\_SchoolClimate | Perseverance | Exercise\_Benefits | Enduring\_Enthusiasm | Unrelenting\_Efforts | Opportunities\_forAutonomy | Teacher\_Support | Classmate\_Support | Exercise\_Behaviour |
| --- | --- | --- | --- | --- | --- | --- | --- | --- | --- |
| Perseverance | .273 | .000 | .000 | .000 | .000 | .000 | .000 | .000 | .000 |
| Exercise\_Benefits | .404 | .000 | .000 | .000 | .000 | .000 | .000 | .000 | .000 |
| Enduring\_Enthusiasm | .000 | .998 | .000 | .000 | .000 | .000 | .000 | .000 | .000 |
| Unrelenting\_Efforts | .000 | .967 | .000 | .000 | .000 | .000 | .000 | .000 | .000 |
| Opportunities\_forAutonomy | .954 | .000 | .000 | .000 | .000 | .000 | .000 | .000 | .000 |
| Teacher\_Support | .670 | .000 | .000 | .000 | .000 | .000 | .000 | .000 | .000 |
| Classmate\_Support | .768 | .000 | .000 | .000 | .000 | .000 | .000 | .000 | .000 |
| Exercise\_Behaviour | .295 | .370 | .405 | .000 | .000 | .000 | .000 | .000 | .000 |
| EE6 | .000 | .000 | .000 | .821 | .000 | .000 | .000 | .000 | .000 |
| EE5 | .000 | .000 | .000 | .885 | .000 | .000 | .000 | .000 | .000 |
| EE4 | .000 | .000 | .000 | .881 | .000 | .000 | .000 | .000 | .000 |
| EE3 | .000 | .000 | .000 | .889 | .000 | .000 | .000 | .000 | .000 |
| EE2 | .000 | .000 | .000 | .874 | .000 | .000 | .000 | .000 | .000 |
| EE1 | .000 | .000 | .000 | .861 | .000 | .000 | .000 | .000 | .000 |
| UE6 | .000 | .000 | .000 | .000 | .793 | .000 | .000 | .000 | .000 |
| UE5 | .000 | .000 | .000 | .000 | .872 | .000 | .000 | .000 | .000 |
| UE4 | .000 | .000 | .000 | .000 | .882 | .000 | .000 | .000 | .000 |
| UE3 | .000 | .000 | .000 | .000 | .883 | .000 | .000 | .000 | .000 |
| UE2 | .000 | .000 | .000 | .000 | .849 | .000 | .000 | .000 | .000 |
| UE1 | .000 | .000 | .000 | .000 | .828 | .000 | .000 | .000 | .000 |
| OA1 | .000 | .000 | .000 | .000 | .000 | .881 | .000 | .000 | .000 |
| OA2 | .000 | .000 | .000 | .000 | .000 | .802 | .000 | .000 | .000 |
| OA3 | .000 | .000 | .000 | .000 | .000 | .828 | .000 | .000 | .000 |
| OA4 | .000 | .000 | .000 | .000 | .000 | .888 | .000 | .000 | .000 |
| OA5 | .000 | .000 | .000 | .000 | .000 | .832 | .000 | .000 | .000 |
| CS1 | .000 | .000 | .000 | .000 | .000 | .000 | .000 | .933 | .000 |
| CS2 | .000 | .000 | .000 | .000 | .000 | .000 | .000 | .918 | .000 |
| CS3 | .000 | .000 | .000 | .000 | .000 | .000 | .000 | .898 | .000 |
| CS4 | .000 | .000 | .000 | .000 | .000 | .000 | .000 | .949 | .000 |
| CS5 | .000 | .000 | .000 | .000 | .000 | .000 | .000 | .929 | .000 |
| CS6 | .000 | .000 | .000 | .000 | .000 | .000 | .000 | .938 | .000 |
| CS7 | .000 | .000 | .000 | .000 | .000 | .000 | .000 | .939 | .000 |
| CS8 | .000 | .000 | .000 | .000 | .000 | .000 | .000 | .933 | .000 |
| CS9 | .000 | .000 | .000 | .000 | .000 | .000 | .000 | .943 | .000 |
| CS10 | .000 | .000 | .000 | .000 | .000 | .000 | .000 | .938 | .000 |
| CS11 | .000 | .000 | .000 | .000 | .000 | .000 | .000 | .942 | .000 |
| CS12 | .000 | .000 | .000 | .000 | .000 | .000 | .000 | .884 | .000 |
| CS13 | .000 | .000 | .000 | .000 | .000 | .000 | .000 | .869 | .000 |
| TS1 | .000 | .000 | .000 | .000 | .000 | .000 | .930 | .000 | .000 |
| TS2 | .000 | .000 | .000 | .000 | .000 | .000 | .958 | .000 | .000 |
| TS3 | .000 | .000 | .000 | .000 | .000 | .000 | .967 | .000 | .000 |
| TS4 | .000 | .000 | .000 | .000 | .000 | .000 | .943 | .000 | .000 |
| TS5 | .000 | .000 | .000 | .000 | .000 | .000 | .945 | .000 | .000 |
| TS6 | .000 | .000 | .000 | .000 | .000 | .000 | .899 | .000 | .000 |
| TS7 | .000 | .000 | .000 | .000 | .000 | .000 | .885 | .000 | .000 |
| EB1 | .000 | .000 | .690 | .000 | .000 | .000 | .000 | .000 | .000 |
| EB2 | .000 | .000 | .682 | .000 | .000 | .000 | .000 | .000 | .000 |
| EB3 | .000 | .000 | .623 | .000 | .000 | .000 | .000 | .000 | .000 |
| EB4 | .000 | .000 | .681 | .000 | .000 | .000 | .000 | .000 | .000 |
| EB5 | .000 | .000 | .590 | .000 | .000 | .000 | .000 | .000 | .000 |
| EX3 | .000 | .000 | .000 | .000 | .000 | .000 | .000 | .000 | .717 |
| EX2 | .000 | .000 | .000 | .000 | .000 | .000 | .000 | .000 | .805 |
| EX1 | .000 | .000 | .000 | .000 | .000 | .000 | .000 | .000 | .718 |

##### Standardized Direct Effects - Two Tailed Significance (BC) (Group number 1 - Default model)

|  | Perceive\_SchoolClimate | Perseverance | Exercise\_Benefits | Enduring\_Enthusiasm | Unrelenting\_Efforts | Opportunities\_forAutonomy | Teacher\_Support | Classmate\_Support | Exercise\_Behaviour |
| --- | --- | --- | --- | --- | --- | --- | --- | --- | --- |
| Perseverance | .002 | ... | ... | ... | ... | ... | ... | ... | ... |
| Exercise\_Benefits | .000 | ... | ... | ... | ... | ... | ... | ... | ... |
| Enduring\_Enthusiasm | ... | .000 | ... | ... | ... | ... | ... | ... | ... |
| Unrelenting\_Efforts | ... | .000 | ... | ... | ... | ... | ... | ... | ... |
| Opportunities\_forAutonomy | .000 | ... | ... | ... | ... | ... | ... | ... | ... |
| Teacher\_Support | .000 | ... | ... | ... | ... | ... | ... | ... | ... |
| Classmate\_Support | .001 | ... | ... | ... | ... | ... | ... | ... | ... |
| Exercise\_Behaviour | .004 | .001 | .000 | ... | ... | ... | ... | ... | ... |
| EE6 | ... | ... | ... | .001 | ... | ... | ... | ... | ... |
| EE5 | ... | ... | ... | .001 | ... | ... | ... | ... | ... |
| EE4 | ... | ... | ... | .001 | ... | ... | ... | ... | ... |
| EE3 | ... | ... | ... | .000 | ... | ... | ... | ... | ... |
| EE2 | ... | ... | ... | .000 | ... | ... | ... | ... | ... |
| EE1 | ... | ... | ... | .001 | ... | ... | ... | ... | ... |
| UE6 | ... | ... | ... | ... | .001 | ... | ... | ... | ... |
| UE5 | ... | ... | ... | ... | .001 | ... | ... | ... | ... |
| UE4 | ... | ... | ... | ... | .000 | ... | ... | ... | ... |
| UE3 | ... | ... | ... | ... | .001 | ... | ... | ... | ... |
| UE2 | ... | ... | ... | ... | .000 | ... | ... | ... | ... |
| UE1 | ... | ... | ... | ... | .001 | ... | ... | ... | ... |
| OA1 | ... | ... | ... | ... | ... | .000 | ... | ... | ... |
| OA2 | ... | ... | ... | ... | ... | .000 | ... | ... | ... |
| OA3 | ... | ... | ... | ... | ... | .000 | ... | ... | ... |
| OA4 | ... | ... | ... | ... | ... | .001 | ... | ... | ... |
| OA5 | ... | ... | ... | ... | ... | .000 | ... | ... | ... |
| CS1 | ... | ... | ... | ... | ... | ... | ... | .000 | ... |
| CS2 | ... | ... | ... | ... | ... | ... | ... | .000 | ... |
| CS3 | ... | ... | ... | ... | ... | ... | ... | .001 | ... |
| CS4 | ... | ... | ... | ... | ... | ... | ... | .000 | ... |
| CS5 | ... | ... | ... | ... | ... | ... | ... | .000 | ... |
| CS6 | ... | ... | ... | ... | ... | ... | ... | .000 | ... |
| CS7 | ... | ... | ... | ... | ... | ... | ... | .000 | ... |
| CS8 | ... | ... | ... | ... | ... | ... | ... | .000 | ... |
| CS9 | ... | ... | ... | ... | ... | ... | ... | .000 | ... |
| CS10 | ... | ... | ... | ... | ... | ... | ... | .000 | ... |
| CS11 | ... | ... | ... | ... | ... | ... | ... | .000 | ... |
| CS12 | ... | ... | ... | ... | ... | ... | ... | .001 | ... |
| CS13 | ... | ... | ... | ... | ... | ... | ... | .001 | ... |
| TS1 | ... | ... | ... | ... | ... | ... | .000 | ... | ... |
| TS2 | ... | ... | ... | ... | ... | ... | .001 | ... | ... |
| TS3 | ... | ... | ... | ... | ... | ... | .000 | ... | ... |
| TS4 | ... | ... | ... | ... | ... | ... | .000 | ... | ... |
| TS5 | ... | ... | ... | ... | ... | ... | .001 | ... | ... |
| TS6 | ... | ... | ... | ... | ... | ... | .000 | ... | ... |
| TS7 | ... | ... | ... | ... | ... | ... | .001 | ... | ... |
| EB1 | ... | ... | .000 | ... | ... | ... | ... | ... | ... |
| EB2 | ... | ... | .001 | ... | ... | ... | ... | ... | ... |
| EB3 | ... | ... | .001 | ... | ... | ... | ... | ... | ... |
| EB4 | ... | ... | .000 | ... | ... | ... | ... | ... | ... |
| EB5 | ... | ... | .000 | ... | ... | ... | ... | ... | ... |
| EX3 | ... | ... | ... | ... | ... | ... | ... | ... | .000 |
| EX2 | ... | ... | ... | ... | ... | ... | ... | ... | .000 |
| EX1 | ... | ... | ... | ... | ... | ... | ... | ... | .000 |

##### Indirect Effects (Group number 1 - Default model)

##### Indirect Effects - Lower Bounds (BC) (Group number 1 - Default model)

|  | Perceive\_SchoolClimate | Perseverance | Exercise\_Benefits | Enduring\_Enthusiasm | Unrelenting\_Efforts | Opportunities\_forAutonomy | Teacher\_Support | Classmate\_Support | Exercise\_Behaviour |
| --- | --- | --- | --- | --- | --- | --- | --- | --- | --- |
| Perseverance | .000 | .000 | .000 | .000 | .000 | .000 | .000 | .000 | .000 |
| Exercise\_Benefits | .000 | .000 | .000 | .000 | .000 | .000 | .000 | .000 | .000 |
| Enduring\_Enthusiasm | .147 | .000 | .000 | .000 | .000 | .000 | .000 | .000 | .000 |
| Unrelenting\_Efforts | .125 | .000 | .000 | .000 | .000 | .000 | .000 | .000 | .000 |
| Opportunities\_forAutonomy | .000 | .000 | .000 | .000 | .000 | .000 | .000 | .000 | .000 |
| Teacher\_Support | .000 | .000 | .000 | .000 | .000 | .000 | .000 | .000 | .000 |
| Classmate\_Support | .000 | .000 | .000 | .000 | .000 | .000 | .000 | .000 | .000 |
| Exercise\_Behaviour | .121 | .000 | .000 | .000 | .000 | .000 | .000 | .000 | .000 |
| EE6 | .142 | .803 | .000 | .000 | .000 | .000 | .000 | .000 | .000 |
| EE5 | .150 | .859 | .000 | .000 | .000 | .000 | .000 | .000 | .000 |
| EE4 | .148 | .834 | .000 | .000 | .000 | .000 | .000 | .000 | .000 |
| EE3 | .155 | .877 | .000 | .000 | .000 | .000 | .000 | .000 | .000 |
| EE2 | .143 | .830 | .000 | .000 | .000 | .000 | .000 | .000 | .000 |
| EE1 | .147 | .844 | .000 | .000 | .000 | .000 | .000 | .000 | .000 |
| UE6 | .141 | .987 | .000 | .000 | .000 | .000 | .000 | .000 | .000 |
| UE5 | .137 | .993 | .000 | .000 | .000 | .000 | .000 | .000 | .000 |
| UE4 | .141 | 1.053 | .000 | .000 | .000 | .000 | .000 | .000 | .000 |
| UE3 | .142 | 1.049 | .000 | .000 | .000 | .000 | .000 | .000 | .000 |
| UE2 | .129 | .936 | .000 | .000 | .000 | .000 | .000 | .000 | .000 |
| UE1 | .125 | 1.000 | .000 | .000 | .000 | .000 | .000 | .000 | .000 |
| OA1 | .983 | .000 | .000 | .000 | .000 | .000 | .000 | .000 | .000 |
| OA2 | .929 | .000 | .000 | .000 | .000 | .000 | .000 | .000 | .000 |
| OA3 | .978 | .000 | .000 | .000 | .000 | .000 | .000 | .000 | .000 |
| OA4 | .955 | .000 | .000 | .000 | .000 | .000 | .000 | .000 | .000 |
| OA5 | 1.286 | .000 | .000 | .000 | .000 | .000 | .000 | .000 | .000 |
| CS1 | .941 | .000 | .000 | .000 | .000 | .000 | .000 | .000 | .000 |
| CS2 | .918 | .000 | .000 | .000 | .000 | .000 | .000 | .000 | .000 |
| CS3 | .950 | .000 | .000 | .000 | .000 | .000 | .000 | .000 | .000 |
| CS4 | .950 | .000 | .000 | .000 | .000 | .000 | .000 | .000 | .000 |
| CS5 | .949 | .000 | .000 | .000 | .000 | .000 | .000 | .000 | .000 |
| CS6 | .968 | .000 | .000 | .000 | .000 | .000 | .000 | .000 | .000 |
| CS7 | 1.000 | .000 | .000 | .000 | .000 | .000 | .000 | .000 | .000 |
| CS8 | .944 | .000 | .000 | .000 | .000 | .000 | .000 | .000 | .000 |
| CS9 | 1.018 | .000 | .000 | .000 | .000 | .000 | .000 | .000 | .000 |
| CS10 | .979 | .000 | .000 | .000 | .000 | .000 | .000 | .000 | .000 |
| CS11 | .972 | .000 | .000 | .000 | .000 | .000 | .000 | .000 | .000 |
| CS12 | 1.176 | .000 | .000 | .000 | .000 | .000 | .000 | .000 | .000 |
| CS13 | 1.102 | .000 | .000 | .000 | .000 | .000 | .000 | .000 | .000 |
| TS1 | .655 | .000 | .000 | .000 | .000 | .000 | .000 | .000 | .000 |
| TS2 | .728 | .000 | .000 | .000 | .000 | .000 | .000 | .000 | .000 |
| TS3 | .727 | .000 | .000 | .000 | .000 | .000 | .000 | .000 | .000 |
| TS4 | .719 | .000 | .000 | .000 | .000 | .000 | .000 | .000 | .000 |
| TS5 | .724 | .000 | .000 | .000 | .000 | .000 | .000 | .000 | .000 |
| TS6 | .632 | .000 | .000 | .000 | .000 | .000 | .000 | .000 | .000 |
| TS7 | .808 | .000 | .000 | .000 | .000 | .000 | .000 | .000 | .000 |
| EB1 | .150 | .000 | .000 | .000 | .000 | .000 | .000 | .000 | .000 |
| EB2 | .148 | .000 | .000 | .000 | .000 | .000 | .000 | .000 | .000 |
| EB3 | .127 | .000 | .000 | .000 | .000 | .000 | .000 | .000 | .000 |
| EB4 | .146 | .000 | .000 | .000 | .000 | .000 | .000 | .000 | .000 |
| EB5 | .134 | .000 | .000 | .000 | .000 | .000 | .000 | .000 | .000 |
| EX3 | .339 | .146 | .287 | .000 | .000 | .000 | .000 | .000 | .000 |
| EX2 | .370 | .152 | .315 | .000 | .000 | .000 | .000 | .000 | .000 |
| EX1 | .312 | .135 | .273 | .000 | .000 | .000 | .000 | .000 | .000 |

##### Indirect Effects - Upper Bounds (BC) (Group number 1 - Default model)

|  | Perceive\_SchoolClimate | Perseverance | Exercise\_Benefits | Enduring\_Enthusiasm | Unrelenting\_Efforts | Opportunities\_forAutonomy | Teacher\_Support | Classmate\_Support | Exercise\_Behaviour |
| --- | --- | --- | --- | --- | --- | --- | --- | --- | --- |
| Perseverance | .000 | .000 | .000 | .000 | .000 | .000 | .000 | .000 | .000 |
| Exercise\_Benefits | .000 | .000 | .000 | .000 | .000 | .000 | .000 | .000 | .000 |
| Enduring\_Enthusiasm | .719 | .000 | .000 | .000 | .000 | .000 | .000 | .000 | .000 |
| Unrelenting\_Efforts | .612 | .000 | .000 | .000 | .000 | .000 | .000 | .000 | .000 |
| Opportunities\_forAutonomy | .000 | .000 | .000 | .000 | .000 | .000 | .000 | .000 | .000 |
| Teacher\_Support | .000 | .000 | .000 | .000 | .000 | .000 | .000 | .000 | .000 |
| Classmate\_Support | .000 | .000 | .000 | .000 | .000 | .000 | .000 | .000 | .000 |
| Exercise\_Behaviour | .409 | .000 | .000 | .000 | .000 | .000 | .000 | .000 | .000 |
| EE6 | .695 | 1.617 | .000 | .000 | .000 | .000 | .000 | .000 | .000 |
| EE5 | .739 | 1.723 | .000 | .000 | .000 | .000 | .000 | .000 | .000 |
| EE4 | .721 | 1.674 | .000 | .000 | .000 | .000 | .000 | .000 | .000 |
| EE3 | .751 | 1.758 | .000 | .000 | .000 | .000 | .000 | .000 | .000 |
| EE2 | .704 | 1.651 | .000 | .000 | .000 | .000 | .000 | .000 | .000 |
| EE1 | .719 | 1.700 | .000 | .000 | .000 | .000 | .000 | .000 | .000 |
| UE6 | .670 | 1.186 | .000 | .000 | .000 | .000 | .000 | .000 | .000 |
| UE5 | .654 | 1.143 | .000 | .000 | .000 | .000 | .000 | .000 | .000 |
| UE4 | .685 | 1.216 | .000 | .000 | .000 | .000 | .000 | .000 | .000 |
| UE3 | .688 | 1.210 | .000 | .000 | .000 | .000 | .000 | .000 | .000 |
| UE2 | .613 | 1.080 | .000 | .000 | .000 | .000 | .000 | .000 | .000 |
| UE1 | .612 | 1.000 | .000 | .000 | .000 | .000 | .000 | .000 | .000 |
| OA1 | 1.633 | .000 | .000 | .000 | .000 | .000 | .000 | .000 | .000 |
| OA2 | 1.550 | .000 | .000 | .000 | .000 | .000 | .000 | .000 | .000 |
| OA3 | 1.602 | .000 | .000 | .000 | .000 | .000 | .000 | .000 | .000 |
| OA4 | 1.577 | .000 | .000 | .000 | .000 | .000 | .000 | .000 | .000 |
| OA5 | 2.094 | .000 | .000 | .000 | .000 | .000 | .000 | .000 | .000 |
| CS1 | 1.107 | .000 | .000 | .000 | .000 | .000 | .000 | .000 | .000 |
| CS2 | 1.074 | .000 | .000 | .000 | .000 | .000 | .000 | .000 | .000 |
| CS3 | 1.128 | .000 | .000 | .000 | .000 | .000 | .000 | .000 | .000 |
| CS4 | 1.100 | .000 | .000 | .000 | .000 | .000 | .000 | .000 | .000 |
| CS5 | 1.113 | .000 | .000 | .000 | .000 | .000 | .000 | .000 | .000 |
| CS6 | 1.116 | .000 | .000 | .000 | .000 | .000 | .000 | .000 | .000 |
| CS7 | 1.000 | .000 | .000 | .000 | .000 | .000 | .000 | .000 | .000 |
| CS8 | 1.113 | .000 | .000 | .000 | .000 | .000 | .000 | .000 | .000 |
| CS9 | 1.147 | .000 | .000 | .000 | .000 | .000 | .000 | .000 | .000 |
| CS10 | 1.108 | .000 | .000 | .000 | .000 | .000 | .000 | .000 | .000 |
| CS11 | 1.155 | .000 | .000 | .000 | .000 | .000 | .000 | .000 | .000 |
| CS12 | 1.424 | .000 | .000 | .000 | .000 | .000 | .000 | .000 | .000 |
| CS13 | 1.334 | .000 | .000 | .000 | .000 | .000 | .000 | .000 | .000 |
| TS1 | 1.211 | .000 | .000 | .000 | .000 | .000 | .000 | .000 | .000 |
| TS2 | 1.333 | .000 | .000 | .000 | .000 | .000 | .000 | .000 | .000 |
| TS3 | 1.326 | .000 | .000 | .000 | .000 | .000 | .000 | .000 | .000 |
| TS4 | 1.307 | .000 | .000 | .000 | .000 | .000 | .000 | .000 | .000 |
| TS5 | 1.332 | .000 | .000 | .000 | .000 | .000 | .000 | .000 | .000 |
| TS6 | 1.177 | .000 | .000 | .000 | .000 | .000 | .000 | .000 | .000 |
| TS7 | 1.466 | .000 | .000 | .000 | .000 | .000 | .000 | .000 | .000 |
| EB1 | .536 | .000 | .000 | .000 | .000 | .000 | .000 | .000 | .000 |
| EB2 | .505 | .000 | .000 | .000 | .000 | .000 | .000 | .000 | .000 |
| EB3 | .479 | .000 | .000 | .000 | .000 | .000 | .000 | .000 | .000 |
| EB4 | .516 | .000 | .000 | .000 | .000 | .000 | .000 | .000 | .000 |
| EB5 | .488 | .000 | .000 | .000 | .000 | .000 | .000 | .000 | .000 |
| EX3 | .929 | .404 | .911 | .000 | .000 | .000 | .000 | .000 | .000 |
| EX2 | .980 | .436 | .929 | .000 | .000 | .000 | .000 | .000 | .000 |
| EX1 | .898 | .387 | .849 | .000 | .000 | .000 | .000 | .000 | .000 |

##### Indirect Effects - Two Tailed Significance (BC) (Group number 1 - Default model)

|  | Perceive\_SchoolClimate | Perseverance | Exercise\_Benefits | Enduring\_Enthusiasm | Unrelenting\_Efforts | Opportunities\_forAutonomy | Teacher\_Support | Classmate\_Support | Exercise\_Behaviour |
| --- | --- | --- | --- | --- | --- | --- | --- | --- | --- |
| Perseverance | ... | ... | ... | ... | ... | ... | ... | ... | ... |
| Exercise\_Benefits | ... | ... | ... | ... | ... | ... | ... | ... | ... |
| Enduring\_Enthusiasm | .003 | ... | ... | ... | ... | ... | ... | ... | ... |
| Unrelenting\_Efforts | .003 | ... | ... | ... | ... | ... | ... | ... | ... |
| Opportunities\_forAutonomy | ... | ... | ... | ... | ... | ... | ... | ... | ... |
| Teacher\_Support | ... | ... | ... | ... | ... | ... | ... | ... | ... |
| Classmate\_Support | ... | ... | ... | ... | ... | ... | ... | ... | ... |
| Exercise\_Behaviour | .000 | ... | ... | ... | ... | ... | ... | ... | ... |
| EE6 | .003 | .000 | ... | ... | ... | ... | ... | ... | ... |
| EE5 | .003 | .000 | ... | ... | ... | ... | ... | ... | ... |
| EE4 | .003 | .000 | ... | ... | ... | ... | ... | ... | ... |
| EE3 | .002 | .000 | ... | ... | ... | ... | ... | ... | ... |
| EE2 | .003 | .000 | ... | ... | ... | ... | ... | ... | ... |
| EE1 | .003 | .000 | ... | ... | ... | ... | ... | ... | ... |
| UE6 | .002 | .000 | ... | ... | ... | ... | ... | ... | ... |
| UE5 | .002 | .000 | ... | ... | ... | ... | ... | ... | ... |
| UE4 | .003 | .000 | ... | ... | ... | ... | ... | ... | ... |
| UE3 | .002 | .000 | ... | ... | ... | ... | ... | ... | ... |
| UE2 | .002 | .000 | ... | ... | ... | ... | ... | ... | ... |
| UE1 | .003 | ... | ... | ... | ... | ... | ... | ... | ... |
| OA1 | .000 | ... | ... | ... | ... | ... | ... | ... | ... |
| OA2 | .000 | ... | ... | ... | ... | ... | ... | ... | ... |
| OA3 | .000 | ... | ... | ... | ... | ... | ... | ... | ... |
| OA4 | .000 | ... | ... | ... | ... | ... | ... | ... | ... |
| OA5 | .000 | ... | ... | ... | ... | ... | ... | ... | ... |
| CS1 | .000 | ... | ... | ... | ... | ... | ... | ... | ... |
| CS2 | .000 | ... | ... | ... | ... | ... | ... | ... | ... |
| CS3 | .000 | ... | ... | ... | ... | ... | ... | ... | ... |
| CS4 | .000 | ... | ... | ... | ... | ... | ... | ... | ... |
| CS5 | .000 | ... | ... | ... | ... | ... | ... | ... | ... |
| CS6 | .000 | ... | ... | ... | ... | ... | ... | ... | ... |
| CS7 | ... | ... | ... | ... | ... | ... | ... | ... | ... |
| CS8 | .000 | ... | ... | ... | ... | ... | ... | ... | ... |
| CS9 | .000 | ... | ... | ... | ... | ... | ... | ... | ... |
| CS10 | .000 | ... | ... | ... | ... | ... | ... | ... | ... |
| CS11 | .000 | ... | ... | ... | ... | ... | ... | ... | ... |
| CS12 | .000 | ... | ... | ... | ... | ... | ... | ... | ... |
| CS13 | .000 | ... | ... | ... | ... | ... | ... | ... | ... |
| TS1 | .000 | ... | ... | ... | ... | ... | ... | ... | ... |
| TS2 | .000 | ... | ... | ... | ... | ... | ... | ... | ... |
| TS3 | .000 | ... | ... | ... | ... | ... | ... | ... | ... |
| TS4 | .000 | ... | ... | ... | ... | ... | ... | ... | ... |
| TS5 | .000 | ... | ... | ... | ... | ... | ... | ... | ... |
| TS6 | .000 | ... | ... | ... | ... | ... | ... | ... | ... |
| TS7 | .000 | ... | ... | ... | ... | ... | ... | ... | ... |
| EB1 | .000 | ... | ... | ... | ... | ... | ... | ... | ... |
| EB2 | .000 | ... | ... | ... | ... | ... | ... | ... | ... |
| EB3 | .000 | ... | ... | ... | ... | ... | ... | ... | ... |
| EB4 | .000 | ... | ... | ... | ... | ... | ... | ... | ... |
| EB5 | .000 | ... | ... | ... | ... | ... | ... | ... | ... |
| EX3 | .000 | .000 | .000 | ... | ... | ... | ... | ... | ... |
| EX2 | .000 | .000 | .000 | ... | ... | ... | ... | ... | ... |
| EX1 | .000 | .000 | .000 | ... | ... | ... | ... | ... | ... |

##### Standardized Indirect Effects (Group number 1 - Default model)

##### Standardized Indirect Effects - Lower Bounds (BC) (Group number 1 - Default model)

|  | Perceive\_SchoolClimate | Perseverance | Exercise\_Benefits | Enduring\_Enthusiasm | Unrelenting\_Efforts | Opportunities\_forAutonomy | Teacher\_Support | Classmate\_Support | Exercise\_Behaviour |
| --- | --- | --- | --- | --- | --- | --- | --- | --- | --- |
| Perseverance | .000 | .000 | .000 | .000 | .000 | .000 | .000 | .000 | .000 |
| Exercise\_Benefits | .000 | .000 | .000 | .000 | .000 | .000 | .000 | .000 | .000 |
| Enduring\_Enthusiasm | .056 | .000 | .000 | .000 | .000 | .000 | .000 | .000 | .000 |
| Unrelenting\_Efforts | .056 | .000 | .000 | .000 | .000 | .000 | .000 | .000 | .000 |
| Opportunities\_forAutonomy | .000 | .000 | .000 | .000 | .000 | .000 | .000 | .000 | .000 |
| Teacher\_Support | .000 | .000 | .000 | .000 | .000 | .000 | .000 | .000 | .000 |
| Classmate\_Support | .000 | .000 | .000 | .000 | .000 | .000 | .000 | .000 | .000 |
| Exercise\_Behaviour | .068 | .000 | .000 | .000 | .000 | .000 | .000 | .000 | .000 |
| EE6 | .043 | .539 | .000 | .000 | .000 | .000 | .000 | .000 | .000 |
| EE5 | .048 | .600 | .000 | .000 | .000 | .000 | .000 | .000 | .000 |
| EE4 | .048 | .600 | .000 | .000 | .000 | .000 | .000 | .000 | .000 |
| EE3 | .049 | .605 | .000 | .000 | .000 | .000 | .000 | .000 | .000 |
| EE2 | .048 | .597 | .000 | .000 | .000 | .000 | .000 | .000 | .000 |
| EE1 | .047 | .586 | .000 | .000 | .000 | .000 | .000 | .000 | .000 |
| UE6 | .041 | .496 | .000 | .000 | .000 | .000 | .000 | .000 | .000 |
| UE5 | .047 | .573 | .000 | .000 | .000 | .000 | .000 | .000 | .000 |
| UE4 | .048 | .579 | .000 | .000 | .000 | .000 | .000 | .000 | .000 |
| UE3 | .048 | .578 | .000 | .000 | .000 | .000 | .000 | .000 | .000 |
| UE2 | .046 | .555 | .000 | .000 | .000 | .000 | .000 | .000 | .000 |
| UE1 | .044 | .532 | .000 | .000 | .000 | .000 | .000 | .000 | .000 |
| OA1 | .604 | .000 | .000 | .000 | .000 | .000 | .000 | .000 | .000 |
| OA2 | .526 | .000 | .000 | .000 | .000 | .000 | .000 | .000 | .000 |
| OA3 | .550 | .000 | .000 | .000 | .000 | .000 | .000 | .000 | .000 |
| OA4 | .607 | .000 | .000 | .000 | .000 | .000 | .000 | .000 | .000 |
| OA5 | .576 | .000 | .000 | .000 | .000 | .000 | .000 | .000 | .000 |
| CS1 | .506 | .000 | .000 | .000 | .000 | .000 | .000 | .000 | .000 |
| CS2 | .494 | .000 | .000 | .000 | .000 | .000 | .000 | .000 | .000 |
| CS3 | .471 | .000 | .000 | .000 | .000 | .000 | .000 | .000 | .000 |
| CS4 | .515 | .000 | .000 | .000 | .000 | .000 | .000 | .000 | .000 |
| CS5 | .503 | .000 | .000 | .000 | .000 | .000 | .000 | .000 | .000 |
| CS6 | .513 | .000 | .000 | .000 | .000 | .000 | .000 | .000 | .000 |
| CS7 | .509 | .000 | .000 | .000 | .000 | .000 | .000 | .000 | .000 |
| CS8 | .507 | .000 | .000 | .000 | .000 | .000 | .000 | .000 | .000 |
| CS9 | .514 | .000 | .000 | .000 | .000 | .000 | .000 | .000 | .000 |
| CS10 | .510 | .000 | .000 | .000 | .000 | .000 | .000 | .000 | .000 |
| CS11 | .512 | .000 | .000 | .000 | .000 | .000 | .000 | .000 | .000 |
| CS12 | .480 | .000 | .000 | .000 | .000 | .000 | .000 | .000 | .000 |
| CS13 | .466 | .000 | .000 | .000 | .000 | .000 | .000 | .000 | .000 |
| TS1 | .383 | .000 | .000 | .000 | .000 | .000 | .000 | .000 | .000 |
| TS2 | .398 | .000 | .000 | .000 | .000 | .000 | .000 | .000 | .000 |
| TS3 | .401 | .000 | .000 | .000 | .000 | .000 | .000 | .000 | .000 |
| TS4 | .385 | .000 | .000 | .000 | .000 | .000 | .000 | .000 | .000 |
| TS5 | .388 | .000 | .000 | .000 | .000 | .000 | .000 | .000 | .000 |
| TS6 | .367 | .000 | .000 | .000 | .000 | .000 | .000 | .000 | .000 |
| TS7 | .357 | .000 | .000 | .000 | .000 | .000 | .000 | .000 | .000 |
| EB1 | .080 | .000 | .000 | .000 | .000 | .000 | .000 | .000 | .000 |
| EB2 | .082 | .000 | .000 | .000 | .000 | .000 | .000 | .000 | .000 |
| EB3 | .070 | .000 | .000 | .000 | .000 | .000 | .000 | .000 | .000 |
| EB4 | .080 | .000 | .000 | .000 | .000 | .000 | .000 | .000 | .000 |
| EB5 | .067 | .000 | .000 | .000 | .000 | .000 | .000 | .000 | .000 |
| EX3 | .110 | .091 | .097 | .000 | .000 | .000 | .000 | .000 | .000 |
| EX2 | .123 | .102 | .113 | .000 | .000 | .000 | .000 | .000 | .000 |
| EX1 | .106 | .091 | .097 | .000 | .000 | .000 | .000 | .000 | .000 |

##### Standardized Indirect Effects - Upper Bounds (BC) (Group number 1 - Default model)

|  | Perceive\_SchoolClimate | Perseverance | Exercise\_Benefits | Enduring\_Enthusiasm | Unrelenting\_Efforts | Opportunities\_forAutonomy | Teacher\_Support | Classmate\_Support | Exercise\_Behaviour |
| --- | --- | --- | --- | --- | --- | --- | --- | --- | --- |
| Perseverance | .000 | .000 | .000 | .000 | .000 | .000 | .000 | .000 | .000 |
| Exercise\_Benefits | .000 | .000 | .000 | .000 | .000 | .000 | .000 | .000 | .000 |
| Enduring\_Enthusiasm | .238 | .000 | .000 | .000 | .000 | .000 | .000 | .000 | .000 |
| Unrelenting\_Efforts | .229 | .000 | .000 | .000 | .000 | .000 | .000 | .000 | .000 |
| Opportunities\_forAutonomy | .000 | .000 | .000 | .000 | .000 | .000 | .000 | .000 | .000 |
| Teacher\_Support | .000 | .000 | .000 | .000 | .000 | .000 | .000 | .000 | .000 |
| Classmate\_Support | .000 | .000 | .000 | .000 | .000 | .000 | .000 | .000 | .000 |
| Exercise\_Behaviour | .191 | .000 | .000 | .000 | .000 | .000 | .000 | .000 | .000 |
| EE6 | .188 | .786 | .000 | .000 | .000 | .000 | .000 | .000 | .000 |
| EE5 | .205 | .862 | .000 | .000 | .000 | .000 | .000 | .000 | .000 |
| EE4 | .204 | .856 | .000 | .000 | .000 | .000 | .000 | .000 | .000 |
| EE3 | .206 | .866 | .000 | .000 | .000 | .000 | .000 | .000 | .000 |
| EE2 | .202 | .849 | .000 | .000 | .000 | .000 | .000 | .000 | .000 |
| EE1 | .199 | .840 | .000 | .000 | .000 | .000 | .000 | .000 | .000 |
| UE6 | .170 | .728 | .000 | .000 | .000 | .000 | .000 | .000 | .000 |
| UE5 | .194 | .822 | .000 | .000 | .000 | .000 | .000 | .000 | .000 |
| UE4 | .196 | .829 | .000 | .000 | .000 | .000 | .000 | .000 | .000 |
| UE3 | .197 | .825 | .000 | .000 | .000 | .000 | .000 | .000 | .000 |
| UE2 | .188 | .792 | .000 | .000 | .000 | .000 | .000 | .000 | .000 |
| UE1 | .183 | .770 | .000 | .000 | .000 | .000 | .000 | .000 | .000 |
| OA1 | .804 | .000 | .000 | .000 | .000 | .000 | .000 | .000 | .000 |
| OA2 | .729 | .000 | .000 | .000 | .000 | .000 | .000 | .000 | .000 |
| OA3 | .754 | .000 | .000 | .000 | .000 | .000 | .000 | .000 | .000 |
| OA4 | .811 | .000 | .000 | .000 | .000 | .000 | .000 | .000 | .000 |
| OA5 | .759 | .000 | .000 | .000 | .000 | .000 | .000 | .000 | .000 |
| CS1 | .698 | .000 | .000 | .000 | .000 | .000 | .000 | .000 | .000 |
| CS2 | .686 | .000 | .000 | .000 | .000 | .000 | .000 | .000 | .000 |
| CS3 | .665 | .000 | .000 | .000 | .000 | .000 | .000 | .000 | .000 |
| CS4 | .711 | .000 | .000 | .000 | .000 | .000 | .000 | .000 | .000 |
| CS5 | .697 | .000 | .000 | .000 | .000 | .000 | .000 | .000 | .000 |
| CS6 | .705 | .000 | .000 | .000 | .000 | .000 | .000 | .000 | .000 |
| CS7 | .705 | .000 | .000 | .000 | .000 | .000 | .000 | .000 | .000 |
| CS8 | .698 | .000 | .000 | .000 | .000 | .000 | .000 | .000 | .000 |
| CS9 | .708 | .000 | .000 | .000 | .000 | .000 | .000 | .000 | .000 |
| CS10 | .701 | .000 | .000 | .000 | .000 | .000 | .000 | .000 | .000 |
| CS11 | .704 | .000 | .000 | .000 | .000 | .000 | .000 | .000 | .000 |
| CS12 | .666 | .000 | .000 | .000 | .000 | .000 | .000 | .000 | .000 |
| CS13 | .647 | .000 | .000 | .000 | .000 | .000 | .000 | .000 | .000 |
| TS1 | .606 | .000 | .000 | .000 | .000 | .000 | .000 | .000 | .000 |
| TS2 | .629 | .000 | .000 | .000 | .000 | .000 | .000 | .000 | .000 |
| TS3 | .636 | .000 | .000 | .000 | .000 | .000 | .000 | .000 | .000 |
| TS4 | .616 | .000 | .000 | .000 | .000 | .000 | .000 | .000 | .000 |
| TS5 | .617 | .000 | .000 | .000 | .000 | .000 | .000 | .000 | .000 |
| TS6 | .583 | .000 | .000 | .000 | .000 | .000 | .000 | .000 | .000 |
| TS7 | .570 | .000 | .000 | .000 | .000 | .000 | .000 | .000 | .000 |
| EB1 | .253 | .000 | .000 | .000 | .000 | .000 | .000 | .000 | .000 |
| EB2 | .244 | .000 | .000 | .000 | .000 | .000 | .000 | .000 | .000 |
| EB3 | .228 | .000 | .000 | .000 | .000 | .000 | .000 | .000 | .000 |
| EB4 | .246 | .000 | .000 | .000 | .000 | .000 | .000 | .000 | .000 |
| EB5 | .214 | .000 | .000 | .000 | .000 | .000 | .000 | .000 | .000 |
| EX3 | .266 | .245 | .266 | .000 | .000 | .000 | .000 | .000 | .000 |
| EX2 | .296 | .276 | .290 | .000 | .000 | .000 | .000 | .000 | .000 |
| EX1 | .273 | .247 | .264 | .000 | .000 | .000 | .000 | .000 | .000 |

##### Standardized Indirect Effects - Two Tailed Significance (BC) (Group number 1 - Default model)

|  | Perceive\_SchoolClimate | Perseverance | Exercise\_Benefits | Enduring\_Enthusiasm | Unrelenting\_Efforts | Opportunities\_forAutonomy | Teacher\_Support | Classmate\_Support | Exercise\_Behaviour |
| --- | --- | --- | --- | --- | --- | --- | --- | --- | --- |
| Perseverance | ... | ... | ... | ... | ... | ... | ... | ... | ... |
| Exercise\_Benefits | ... | ... | ... | ... | ... | ... | ... | ... | ... |
| Enduring\_Enthusiasm | .003 | ... | ... | ... | ... | ... | ... | ... | ... |
| Unrelenting\_Efforts | .002 | ... | ... | ... | ... | ... | ... | ... | ... |
| Opportunities\_forAutonomy | ... | ... | ... | ... | ... | ... | ... | ... | ... |
| Teacher\_Support | ... | ... | ... | ... | ... | ... | ... | ... | ... |
| Classmate\_Support | ... | ... | ... | ... | ... | ... | ... | ... | ... |
| Exercise\_Behaviour | .000 | ... | ... | ... | ... | ... | ... | ... | ... |
| EE6 | .003 | .001 | ... | ... | ... | ... | ... | ... | ... |
| EE5 | .003 | .000 | ... | ... | ... | ... | ... | ... | ... |
| EE4 | .003 | .000 | ... | ... | ... | ... | ... | ... | ... |
| EE3 | .003 | .000 | ... | ... | ... | ... | ... | ... | ... |
| EE2 | .003 | .000 | ... | ... | ... | ... | ... | ... | ... |
| EE1 | .003 | .000 | ... | ... | ... | ... | ... | ... | ... |
| UE6 | .002 | .000 | ... | ... | ... | ... | ... | ... | ... |
| UE5 | .002 | .000 | ... | ... | ... | ... | ... | ... | ... |
| UE4 | .002 | .000 | ... | ... | ... | ... | ... | ... | ... |
| UE3 | .002 | .000 | ... | ... | ... | ... | ... | ... | ... |
| UE2 | .002 | .000 | ... | ... | ... | ... | ... | ... | ... |
| UE1 | .002 | .000 | ... | ... | ... | ... | ... | ... | ... |
| OA1 | .000 | ... | ... | ... | ... | ... | ... | ... | ... |
| OA2 | .000 | ... | ... | ... | ... | ... | ... | ... | ... |
| OA3 | .000 | ... | ... | ... | ... | ... | ... | ... | ... |
| OA4 | .000 | ... | ... | ... | ... | ... | ... | ... | ... |
| OA5 | .000 | ... | ... | ... | ... | ... | ... | ... | ... |
| CS1 | .000 | ... | ... | ... | ... | ... | ... | ... | ... |
| CS2 | .001 | ... | ... | ... | ... | ... | ... | ... | ... |
| CS3 | .001 | ... | ... | ... | ... | ... | ... | ... | ... |
| CS4 | .001 | ... | ... | ... | ... | ... | ... | ... | ... |
| CS5 | .000 | ... | ... | ... | ... | ... | ... | ... | ... |
| CS6 | .000 | ... | ... | ... | ... | ... | ... | ... | ... |
| CS7 | .000 | ... | ... | ... | ... | ... | ... | ... | ... |
| CS8 | .000 | ... | ... | ... | ... | ... | ... | ... | ... |
| CS9 | .001 | ... | ... | ... | ... | ... | ... | ... | ... |
| CS10 | .001 | ... | ... | ... | ... | ... | ... | ... | ... |
| CS11 | .000 | ... | ... | ... | ... | ... | ... | ... | ... |
| CS12 | .001 | ... | ... | ... | ... | ... | ... | ... | ... |
| CS13 | .000 | ... | ... | ... | ... | ... | ... | ... | ... |
| TS1 | .000 | ... | ... | ... | ... | ... | ... | ... | ... |
| TS2 | .000 | ... | ... | ... | ... | ... | ... | ... | ... |
| TS3 | .000 | ... | ... | ... | ... | ... | ... | ... | ... |
| TS4 | .000 | ... | ... | ... | ... | ... | ... | ... | ... |
| TS5 | .000 | ... | ... | ... | ... | ... | ... | ... | ... |
| TS6 | .000 | ... | ... | ... | ... | ... | ... | ... | ... |
| TS7 | .000 | ... | ... | ... | ... | ... | ... | ... | ... |
| EB1 | .000 | ... | ... | ... | ... | ... | ... | ... | ... |
| EB2 | .000 | ... | ... | ... | ... | ... | ... | ... | ... |
| EB3 | .000 | ... | ... | ... | ... | ... | ... | ... | ... |
| EB4 | .000 | ... | ... | ... | ... | ... | ... | ... | ... |
| EB5 | .000 | ... | ... | ... | ... | ... | ... | ... | ... |
| EX3 | .000 | .000 | .000 | ... | ... | ... | ... | ... | ... |
| EX2 | .000 | .001 | .000 | ... | ... | ... | ... | ... | ... |
| EX1 | .000 | .000 | .000 | ... | ... | ... | ... | ... | ... |

##### Minimization History (Default model)

| Iteration |  | Negative eigenvalues | Condition # | Smallest eigenvalue | Diameter | F | NTries | Ratio |
| --- | --- | --- | --- | --- | --- | --- | --- | --- |
| 0 | e | 27 |  | -2.054 | 9999.000 | 24037.546 | 0 | 9999.000 |
| 1 | e\* | 29 |  | -.378 | 6.420 | 12190.665 | 19 | .163 |
| 2 | e\* | 15 |  | -.374 | 2.037 | 7167.042 | 4 | .994 |
| 3 | e\* | 8 |  | -.123 | .423 | 6215.557 | 6 | .837 |
| 4 | e | 5 |  | -.148 | .954 | 4626.281 | 6 | .921 |
| 5 | e\* | 2 |  | -.129 | 2.164 | 2883.834 | 7 | .826 |
| 6 | e\* | 2 |  | -.579 | 1.611 | 2046.200 | 5 | .698 |
| 7 | e | 1 |  | -.016 | .719 | 1790.906 | 7 | .707 |
| 8 | e | 0 | 269.211 |  | .830 | 1687.850 | 5 | .901 |
| 9 | e | 0 | 2894.038 |  | .363 | 1665.988 | 1 | 1.002 |
| 10 | e | 0 | 776.745 |  | .203 | 1663.830 | 1 | 1.039 |
| 11 | e | 0 | 802.582 |  | .088 | 1663.641 | 1 | 1.009 |
| 12 | e | 0 | 816.354 |  | .006 | 1663.637 | 1 | 1.000 |
| 13 | e | 0 | 816.673 |  | .000 | 1663.637 | 1 | 1.002 |

##### Bootstrap (Default model)

##### Summary of Bootstrap Iterations (Default model)

##### (Default model)

| Iterations | Method 0 | Method 1 | Method 2 |
| --- | --- | --- | --- |
| 1 | 0 | 0 | 0 |
| 2 | 0 | 0 | 0 |
| 3 | 0 | 0 | 0 |
| 4 | 0 | 0 | 0 |
| 5 | 0 | 0 | 0 |
| 6 | 0 | 0 | 0 |
| 7 | 0 | 0 | 0 |
| 8 | 0 | 8 | 0 |
| 9 | 0 | 110 | 0 |
| 10 | 0 | 360 | 0 |
| 11 | 0 | 726 | 0 |
| 12 | 0 | 813 | 0 |
| 13 | 0 | 860 | 0 |
| 14 | 0 | 686 | 0 |
| 15 | 0 | 526 | 0 |
| 16 | 0 | 361 | 0 |
| 17 | 0 | 239 | 0 |
| 18 | 0 | 133 | 0 |
| 19 | 0 | 178 | 0 |
| Total | 0 | 5000 | 0 |

0 bootstrap samples were unused because of a singular covariance matrix.

0 bootstrap samples were unused because a solution was not found.

5000 usable bootstrap samples were obtained.

##### Bootstrap Distributions (Default model)

##### ML discrepancy (implied vs sample) (Default model)

|  |  |  |
| --- | --- | --- |
|  |  | |-------------------- |
|  | 2316.980 | |\* |
|  | 2432.538 | |\* |
|  | 2548.096 | |\*\* |
|  | 2663.655 | |\*\*\*\*\*\* |
|  | 2779.213 | |\*\*\*\*\*\*\*\*\*\*\*\* |
|  | 2894.772 | |\*\*\*\*\*\*\*\*\*\*\*\*\*\*\*\*\*\* |
|  | 3010.330 | |\*\*\*\*\*\*\*\*\*\*\*\*\*\*\*\*\*\*\*\* |
| N = 5000 | 3125.889 | |\*\*\*\*\*\*\*\*\*\*\*\*\*\*\*\* |
| Mean = 3017.368 | 3241.447 | |\*\*\*\*\*\*\*\*\*\*\* |
| S. e. = 3.018 | 3357.006 | |\*\*\*\*\*\* |
|  | 3472.564 | |\*\*\* |
|  | 3588.123 | |\* |
|  | 3703.681 | |\* |
|  | 3819.240 | |\* |
|  | 3934.798 | |\* |
|  |  | |-------------------- |

##### ML discrepancy (implied vs pop) (Default model)

|  |  |  |
| --- | --- | --- |
|  |  | |-------------------- |
|  | 1801.633 | |\* |
|  | 1876.142 | |\*\*\*\*\*\*\*\*\*\*\*\*\* |
|  | 1950.651 | |\*\*\*\*\*\*\*\*\*\*\*\*\*\*\*\*\*\*\*\* |
|  | 2025.161 | |\*\*\*\*\*\*\*\*\*\* |
|  | 2099.670 | |\*\*\*\* |
|  | 2174.179 | |\*\* |
|  | 2248.688 | |\* |
| N = 5000 | 2323.197 | |\* |
| Mean = 1963.345 | 2397.706 | |\* |
| S. e. = 1.190 | 2472.215 | |\* |
|  | 2546.725 | |\* |
|  | 2621.234 | |\* |
|  | 2695.743 | | |
|  | 2770.252 | | |
|  | 2844.761 | |\* |
|  |  | |-------------------- |

##### K-L overoptimism (unstabilized) (Default model)

|  |  |  |
| --- | --- | --- |
|  |  | |-------------------- |
|  | -2424.780 | |\* |
|  | -1859.502 | |\* |
|  | -1294.224 | |\*\*\* |
|  | -728.945 | |\*\*\*\*\*\*\*\* |
|  | -163.667 | |\*\*\*\*\*\*\*\*\*\*\*\*\*\*\*\* |
|  | 401.612 | |\*\*\*\*\*\*\*\*\*\*\*\*\*\*\*\*\*\*\*\* |
|  | 966.890 | |\*\*\*\*\*\*\*\*\*\*\*\*\*\*\*\*\*\* |
| N = 5000 | 1532.168 | |\*\*\*\*\*\*\*\*\*\*\* |
| Mean = 591.444 | 2097.447 | |\*\*\*\*\*\* |
| S. e. = 13.191 | 2662.725 | |\*\*\* |
|  | 3228.004 | |\* |
|  | 3793.282 | |\* |
|  | 4358.560 | |\* |
|  | 4923.839 | | |
|  | 5489.117 | |\* |
|  |  | |-------------------- |

##### K-L overoptimism (stabilized) (Default model)

|  |  |  |
| --- | --- | --- |
|  |  | |-------------------- |
|  | -2.353 | |\* |
|  | 176.947 | |\*\*\*\* |
|  | 356.248 | |\*\*\*\*\*\*\*\*\*\*\*\*\*\* |
|  | 535.549 | |\*\*\*\*\*\*\*\*\*\*\*\*\*\*\*\*\*\*\*\* |
|  | 714.849 | |\*\*\*\*\*\*\*\*\*\*\*\*\*\* |
|  | 894.150 | |\*\*\*\*\*\* |
|  | 1073.451 | |\*\*\* |
| N = 5000 | 1252.751 | |\* |
| Mean = 586.772 | 1432.052 | |\* |
| S. e. = 3.327 | 1611.353 | |\* |
|  | 1790.653 | |\* |
|  | 1969.954 | | |
|  | 2149.255 | | |
|  | 2328.555 | | |
|  | 2507.856 | |\* |
|  |  | |-------------------- |

##### Model Fit Summary

##### CMIN

| Model | NPAR | CMIN | DF | P | CMIN/DF |
| --- | --- | --- | --- | --- | --- |
| Default model | 100 | 1663.637 | 935 | .000 | 1.779 |
| Saturated model | 1035 | .000 | 0 |
| Independence model | 45 | 25224.407 | 990 | .000 | 25.479 |

##### RMR, GFI

| Model | RMR | GFI | AGFI | PGFI |
| --- | --- | --- | --- | --- |
| Default model | .036 | .887 | .875 | .802 |
| Saturated model | .000 | 1.000 |  |  |
| Independence model | .265 | .157 | .119 | .150 |

##### Baseline Comparisons

| Model | NFI Delta1 | RFI rho1 | IFI Delta2 | TLI rho2 | CFI |
| --- | --- | --- | --- | --- | --- |
| Default model | .934 | .930 | .970 | .968 | .970 |
| Saturated model | 1.000 |  | 1.000 |  | 1.000 |
| Independence model | .000 | .000 | .000 | .000 | .000 |

##### Parsimony-Adjusted Measures

| Model | PRATIO | PNFI | PCFI |
| --- | --- | --- | --- |
| Default model | .944 | .882 | .916 |
| Saturated model | .000 | .000 | .000 |
| Independence model | 1.000 | .000 | .000 |

##### NCP

| Model | NCP | LO 90 | HI 90 |
| --- | --- | --- | --- |
| Default model | 728.637 | 618.742 | 846.356 |
| Saturated model | .000 | .000 | .000 |
| Independence model | 24234.407 | 23720.269 | 24754.909 |

##### FMIN

| Model | FMIN | F0 | LO 90 | HI 90 |
| --- | --- | --- | --- | --- |
| Default model | 2.844 | 1.246 | 1.058 | 1.447 |
| Saturated model | .000 | .000 | .000 | .000 |
| Independence model | 43.119 | 41.426 | 40.547 | 42.316 |

##### RMSEA

| Model | RMSEA | LO 90 | HI 90 | PCLOSE |
| --- | --- | --- | --- | --- |
| Default model | .036 | .034 | .039 | 1.000 |
| Independence model | .205 | .202 | .207 | .000 |

##### AIC

| Model | AIC | BCC | BIC | CAIC |
| --- | --- | --- | --- | --- |
| Default model | 1863.637 | 1880.705 | 2300.969 | 2400.969 |
| Saturated model | 2070.000 | 2246.660 | 6596.386 | 7631.386 |
| Independence model | 25314.407 | 25322.088 | 25511.207 | 25556.207 |

##### ECVI

| Model | ECVI | LO 90 | HI 90 | MECVI |
| --- | --- | --- | --- | --- |
| Default model | 3.186 | 2.998 | 3.387 | 3.215 |
| Saturated model | 3.538 | 3.538 | 3.538 | 3.840 |
| Independence model | 43.272 | 42.394 | 44.162 | 43.286 |

##### HOELTER

| Model | HOELTER .05 | HOELTER .01 |
| --- | --- | --- |
| Default model | 355 | 366 |
| Independence model | 25 | 26 |

##### Execution time summary

|  |  |
| --- | --- |
| Minimization: | .094 |
| Miscellaneous: | 1.568 |
| Bootstrap: | 81.841 |
| Total: | 83.503 |
